# 7,8-Dihydro-8-oxo-1,*N*^6^-ethenoadenine: an exclusively Hoogsteen-paired thymine mimic in DNA that induces A→T transversions in *Escherichia coli*

**DOI:** 10.1093/nar/gkac148

**Published:** 2022-03-02

**Authors:** Andrey V Aralov, Nina Gubina, Cristina Cabrero, Vladimir B Tsvetkov, Anton V Turaev, Bogdan I Fedeles, Robert G Croy, Ekaterina A Isaakova, Denis Melnik, Svetlana Dukova, Dmitriy Y Ryazantsev, Alexei A Khrulev, Anna M Varizhuk, Carlos González, Timofei S Zatsepin, John M Essigmann

**Affiliations:** Shemyakin-Ovchinnikov Institute of Bioorganic Chemistry RAS, Moscow 117997, Russia; Department of Biological Engineering, Department of Chemistry and Center for Environmental Health Sciences, Massachusetts Institute of Technology, Cambridge, MA 02139, USA; Institute of Theoretical and Experimental Biophysics RAS, Pushchino 142290, Russia; Instituto de Química-Física Rocasolano (IQFR-CSIC), Madrid 28006, Spain; Federal Research and Clinical Center of Physical Chemical Medicine of Federal Medical Biological Agency, Moscow 119435, Russia; World-Class Research Center “Digital biodesign and personalized healthcare”, Sechenov First Moscow State Medical University, Moscow 119146, Russia; Federal Research and Clinical Center of Physical Chemical Medicine of Federal Medical Biological Agency, Moscow 119435, Russia; Department of Biological Engineering, Department of Chemistry and Center for Environmental Health Sciences, Massachusetts Institute of Technology, Cambridge, MA 02139, USA; Department of Biological Engineering, Department of Chemistry and Center for Environmental Health Sciences, Massachusetts Institute of Technology, Cambridge, MA 02139, USA; Federal Research and Clinical Center of Physical Chemical Medicine of Federal Medical Biological Agency, Moscow 119435, Russia; Center for Life Sciences, Skolkovo Institute of Science and Technology, Moscow 143026, Russia; Center for Life Sciences, Skolkovo Institute of Science and Technology, Moscow 143026, Russia; Shemyakin-Ovchinnikov Institute of Bioorganic Chemistry RAS, Moscow 117997, Russia; Shemyakin-Ovchinnikov Institute of Bioorganic Chemistry RAS, Moscow 117997, Russia; Federal Research and Clinical Center of Physical Chemical Medicine of Federal Medical Biological Agency, Moscow 119435, Russia; Moscow Institute of Physics and Technology, Dolgoprudny 141701, Russia; Instituto de Química-Física Rocasolano (IQFR-CSIC), Madrid 28006, Spain; Center for Life Sciences, Skolkovo Institute of Science and Technology, Moscow 143026, Russia; Chemistry Department, Lomonosov Moscow State University, Moscow 119992, Russia; Department of Biological Engineering, Department of Chemistry and Center for Environmental Health Sciences, Massachusetts Institute of Technology, Cambridge, MA 02139, USA

## Abstract

This work investigated the structural and biological properties of DNA containing 7,8-dihydro-8-oxo-1,*N*^6^-ethenoadenine (oxo-ϵA), a non-natural synthetic base that combines structural features of two naturally occurring DNA lesions (7,8-dihydro-8-oxoadenine and 1,*N*^6^-ethenoadenine). UV-, CD-, NMR spectroscopies and molecular modeling of DNA duplexes revealed that oxo-ϵA adopts the non-canonical syn conformation (χ = 65º) and fits very well among surrounding residues without inducing major distortions in local helical architecture. The adduct remarkably mimics the natural base thymine. When considered as an adenine-derived DNA lesion, oxo-ϵA was >99% mutagenic in living cells, causing predominantly A→T transversion mutations in *Escherichia coli*. The adduct in a single-stranded vector was not repaired by base excision repair enzymes (MutM and MutY glycosylases) or the AlkB dioxygenase and did not detectably affect the efficacy of DNA replication *in vivo*. When the biological and structural data are viewed together, it is likely that the nearly exclusive syn conformation and thymine mimicry of oxo-ϵA defines the selectivity of base pairing *in vitro* and *in vivo*, resulting in lesion pairing with A during replication. The base pairing properties of oxo-ϵA, its strong fluorescence and its invisibility to enzymatic repair systems *in vivo* are features that are sought in novel DNA-based probes and modulators of gene expression.

## Graphical Abstract

**Graphical Abstract:**
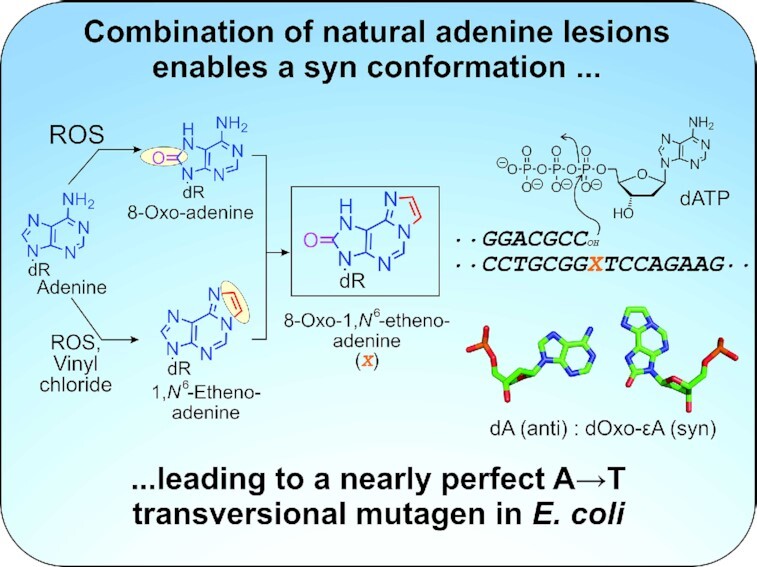
We designed 7,8-dihydro-8-oxo-1,N6-ethenoadenine (oxo-ϵA) with blocked Watson–Crick face and improved Hoogsteen H-pairing side to study the importance of rotamerism in DNA. Combination of two known natural chemical modifications of adenine resulted in directing of the nucleobase in syn conformation that functions as a close mimic of thymine. So, oxo-ϵA was >99% mutagenic, causing predominantly A→T transversion mutations in *E. coli*. Unexpectedly, oxo-ϵA did not affect replication rates and was not repaired in *E. coli*.

## INTRODUCTION

Modified nucleosides, nucleotides and nucleic acids are finding an expanding range of applications in basic research, biotechnology and drug development. Point chemical modifications of nucleobases or the sugar-phosphate backbone facilitate study of DNA–protein interactions (1), enable development of specific inhibitors (2), permit the tuning of duplex stability (3), improve selectivity of siRNA (4) or aptamers (5), and improve pharmaceutical properties of antisense oligonucleotides (6), siRNA (7) and mRNA vaccines (8). Modified bases can improve stability to nucleases, enhance immune responses and promote favourable pharmacokinetics and pharmacodynamics by blocking or enhancing interactions with specific proteins. The opportunity to program these interactions in a prescribed manner helps promote success in the development of oligonucleotide drugs (more than ten of which are already approved by the FDA) and mRNA vaccines for infectious diseases such as COVID-19. The design of such drugs has been informed by results of numerous studies on nuclease-resistant base modifications that create unnatural base pairing yet do not interfere with the machinery of DNA replication (9–11). As one example, isocytidine (isoC) : isoguanosine (isoG) was the first unnatural base pair used in technology (Branched DNA (bDNA) testing kit (Roche)) (12) to increase the thermodynamic stability of a DNA duplex (13). Later it was shown that incorporation of the unnatural base pairs 5-Me-isoC:isoG and A:2-thioT into double helices enhances their thermal stability and resistance to T7 exonuclease digestion (14).

In the present work, we studied a novel DNA lesion, 7,8-dihydro-8-oxo-1,*N*^6^-ethenoadenine (oxo-ϵA) by the tools of structural biology and mutagenesis to define its properties and potential for use in basic research and biotechnology. Oxo-ϵA combines the chemistry and structure of several well-known DNA modifications formed by reactive oxygen species (ROS), such as 7,8-dihydro-8-oxoguanine (oxoG) (15,16), Fapy-dG, uracil, 7,8-dihydro-8-oxoadenine (oxoA) (17) and others (18). In addition to direct genome damage, ROS can oxidize membrane and other lipids leading to the formation of alkyl bridge-adducts (e.g. etheno adducts) with DNA adenines, cytosines and guanines. Most of these lesions can be efficiently removed by base or nucleotide excision repair systems (BER and NER) or by direct oxidative reversal by AlkB family enzymes.

**Figure 1.**
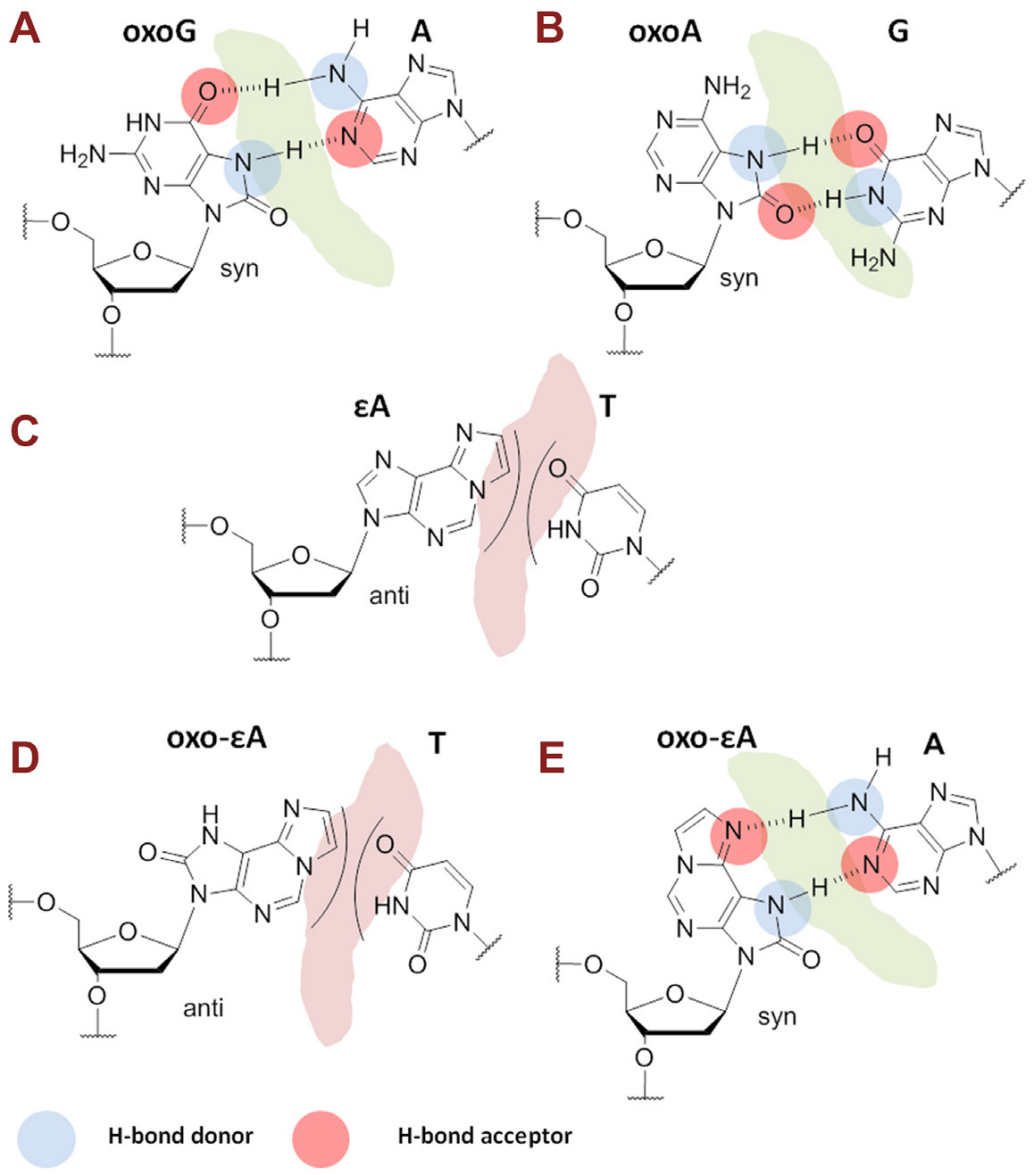
Proposed base pairing patterns of modified nucleobases in different conformations. *Syn*-8-oxoG:*anti*-A (**A**) and *syn*-8-oxoA:*anti*-G (**B**) can promote G→T and A→C transversion mutations, respectively. *Anti*-ϵA cannot pair with T (**C**). *Anti*-oxo-ϵA also cannot pair with T (**D**) and suggested base pairing of *syn*-oxo-ϵA:*anti*-A (**E**). Pairings (**E**) can promote the observed A→T transversion mutations.

Among the common oxidative DNA lesions, oxoG is one of the most prominent and its mutagenic properties have been well studied both *in vitro* and *in vivo* (15,16). Mutagenesis stemming from oxoG can be explained by the ability of the nucleobase to rotate between its syn and anti conformations about the glycosidic bond in the DNA helix (19,20). OxoG in the non-mutagenic anti conformation can form three Watson−Crick hydrogen bonds with cytosine, whereas when it rotates to the syn conformation, two Hoogsteen hydrogen bonds are possible for pairing with adenine (Figure 1A) (20–24). The syn-oxoG:A base pair resembles the cognate pyrimidine (T):purine (A) pair, which explains why oxoG formation in DNA leads to G→T transversion mutations (25).

A structurally similar oxidative DNA lesion is oxoA, which can form a stable Hoogsteen base pair in the syn conformation with an opposing guanine (Figure 1B) (26). The presence of the 8-oxo group in oxoA increases the population of the syn conformer in comparison to native adenine (27,28) and leads to A→C transversion mutations (26). The third natural oxidative stress-induced DNA lesion of relevance to the present work is 1,*N*^6^-ethenoadenine (ϵA), which is also mutagenic (29). This DNA adduct is formed following exposure to the carcinogen vinyl chloride through reaction with adenine of its 2-chloroacetaldehyde metabolite (30) or by the reaction of adenine with endogenous aldehyde by-products of lipid peroxidation (31). In the anti conformation ϵA is unable to pair through hydrogen bonds with any canonical base in DNA duplexes (Figure 1C). However, the presence of the 1,*N^6^*-etheno group can partially shift the syn-anti equilibrium towards the syn conformation of the glycosidic bond (32), resulting in the incorporation of A, C and G opposite ϵA (33–35). Taken together, the observations on oxoG, oxoA and ϵA suggest a common theme for their mechanism of mutagenesis: mutagenic pairing is achieved when the damaged base adopts a syn conformation, which enables the formation of Hoogsteen H-bonds with non-cognate native bases in the opposite DNA strand. H-Bonding is not the only driving force for base pairing (36,37), and hydrophobic and stacking interactions becomes particularly important in the design of unnatural base pairs (UBPs) (38). However, the structural and geometric similarity of all the aforementioned purine adducts and relevant pairing data make it possible to revise the importance of H-bonding in this context. Indeed, the constructed 2′-deoxy-7,8-dihydro-8-oxo-1,*N*^6^-ethenoadenosine modification should block canonical Watson–Crick base pairing (Figure 1D) and provide both an optimal Hoogsteen base pair geometry and a perfect hydrogen bonding pattern (one H-bond donor and one H-bond acceptor) (Figure 1E).

An adenine derivative featuring both an 1,*N*^6^-etheno bridge and an 8-oxo group of the above-described lesions is an unlikely occurrence *in vivo*, but it represents an excellent opportunity to evaluate the structural and biological consequences of rotamerism (the ability of a base to rotate about its glycosidic bond) coupled with a novel predefined H-bonding pattern.

For our applications, we sought a DNA modification that would be both mutagenic and insensitive to the pathways of adduct repair. Here we report the synthesis of oxo-ϵA phosphoramidite and its use to prepare modified oligodeoxynucleotides bearing the designed modification with varied flanking nucleotides. CD-spectroscopy and UV-melting analyses revealed that the oxo-ϵA modification placed opposite adenine in a DNA duplex did not significantly disturb helix geometry and stability in comparison to the cognate T:A base pair. The structure of the DNA duplex bearing oxo-ϵA was determined by 2D NMR techniques, indicating that oxo-ϵA opposite adenine displayed a syn conformation without significant local duplex deformations. The stability of the oxo-ϵA:A base pair was also evaluated by molecular modelling, which lends support to the empirical data.

To evaluate the base pairing properties of oxo-ϵA in living cells, the modification was introduced into a single-stranded M13mp7(L2) phage genome *via* ligation of oligonucleotides that site-specifically contain the modification. Subsequently, the modified phage genomes were replicated in *E. coli* strains proficient or deficient in DNA repair systems capable of acting on similar lesions (e.g. cells lacking MutM, MutY or AlkB), and phage progeny were recovered and their DNA analyzed by next-generation sequencing (NGS). We observed that oxo-ϵA is remarkably mutagenic, inducing almost exclusively A→T transversions in any nucleotide context in all genetic backgrounds evaluated. Thus, our *in vivo* data provide corroborating evidence that oxo-ϵA strongly prefers to pair with adenine during replication. Additionally, we found no indication that oxo-ϵA, under the conditions in which it was studied, was recognized and processed by any DNA repair system in *E. coli*. In sum, our study provides an in depth look at the impact on base-pairing specificity of a novel purine base containing a combination of common chemical DNA alterations that adopts a predominantly syn-pairing conformation in duplex DNA. As DNA–protein interactions play a pivotal role in transcriptional regulation of gene expression, oxo-ϵA can become a valuable tool for both *in vitro* and *in vivo* studies. Being a fluorescent thymine mimic, oxo-ϵA can help monitor changes of secondary structure in DNA-protein complexes and aptamers without risk of repair in the cell. This fluorophore, which is well tolerated in cells, is a prospective component of FRET systems, such as aptamer switches or FRET relay logic gates, in next-generation molecular sensors (39). Also, oxo-ϵA nucleobase can be combined with modified sugar-phosphate backbones that could be used in a wide range of RNA-based applications.

## MATERIALS AND METHODS

For a detailed description of the phosphoramidite and oligonucleotide synthesis, circular dichroism, UV-melting experiments, as well as molecular modelling and NMR spectroscopy, see Supporting Information.

### Primer-extension past oxo-ϵA

The specificity of dATP insertion opposite oxo-ϵA and subsequent primer elongation were studied on native (**ODN11**; see Supplementary Table S1 for oligodeoxynucleotide (ODN) structures) and modified (**ODN12**) templates by primer extension experiments using primer (**ODN13**) bearing Cy5 residue at 5′-end and four native dNTPs (Supplementary Table S1). The reactions were performed by mixing a preliminary annealed primer-template duplex (5 μM) in 1× corresponding reaction buffer, 750 μM each of dNTPs (with or without dATP), and 2 units of either *E. coli* DNA Polymerase I, Large fragment (Klenow fragment) or T4 DNA polymerase (SibEnzyme, Russia). Primer extension was carried out at 37°C for 4 h, with the analysis of a reaction mixture every hour. Formamide-containing dye was used to terminate the reaction and products were evaluated by 19% denaturing PAGE. The gels were scanned using a Typhoon RGB scanner (Amersham, UK). Bands with the products of the primer extension reaction were cut out, and extension products were eluted and analyzed by mass-spectrometry.

### Building control and lesion-containing M13Mp7(L2) genomes

The M13mp7(L2) genome was prepared as described in Delaney and Essigmann (40). Barcoded lesion-containing genomes were constructed based on a previously reported method (41) with some modifications. Briefly, the 16-mer with adenine or oxo-ϵA in the TXG context (X = test site), or a pool containing an equimolar mixture of sixteen 16-mers with adenine or oxo-ϵA in 16 contexts, were ligated to 18-mer containing a trinucleotide barcode unique for a single genome or for a pool of 16-mers. The 34-mer ligation product was then ligated to linearized M13mp7(L2) single-stranded bacteriophage genomic DNA. The ligated M13 genome was extracted with phenol/chloroform/isoamyl alcohol (25:24:1) (Invitrogen) and purified with QIAprep Spin Miniprep Kit (Qiagen) following the manufacturer's protocol.

### Replication of the phage genome construct in *E. coli*

Electrocompetent cells were prepared as described in Delaney and Essigmann (40). One hundred fmol of a single genome or 480 fmol of a pool (NAN or NXN) were electroporated into 100 μl of competent *E. coli* cells. Each electroporation was done in triplicate. All electroporations resulted in at least 5.3 × 10^4^ initial transformation events (infective centers), with most of the samples producing more than 10^5^ events. The progeny phage were amplified in SCS110 cells to dilute out the residual genomic DNA used for electroporation. Single-stranded DNA from the progeny phage preparation was isolated with the QIAprep Spin Miniprep Kit (Qiagen) following the manufacturer's instructions. A ∼1 kb sequence covering the region of interest was amplified in triplicate by PCR; the PCR product was purified with the QIAquick PCR Purification Kit (Qiagen).

### Library preparation and next-generation sequencing

Triplicates of purified 1kB PCR amplicons were pooled and submitted for next generation sequencing as three technical replicates for each biological replicate. Libraries were prepared using Nextera DNA Flex Library Prep (now called Illumina DNA Prep, Illumina), pooled together and submitted for sequencing on a MiSeq machine (Illumina).

### Next-generation sequencing data analysis

After evaluating the quality of reads and removing the adapters, read pairs were concatenated, and only fragments having a 34-mer insert (18-mer barcode and 16-mer lesion oligonucleotide) were selected for the further analysis. Then the reads were split into groups associated with lesions, and within each lesion-related group into subgroups associated with 16 trinucleotide contexts, where applicable, and mapped to the M13mp7(L2) genome containing TAG 18-mer and TAG 16-mer insertions.

The mutation rate was calculated from the piled-up reads for each base inside the region of interest (6244…6280 bases). The miscoding percentage was obtained by calculating the relative proportion of each native base (A, C, G or T) at the site previously occupied by the lesion. Statistical comparison among genotypes and among sequence contexts was performed using ANOVA with the Tukey post-hoc test; *P* < 0.05 was taken as the significance threshold.

## RESULTS

### Evaluation of context-dependent oxo-ϵA base paring within DNA duplexes by UV melting and CD spectroscopy

**Scheme 1.**
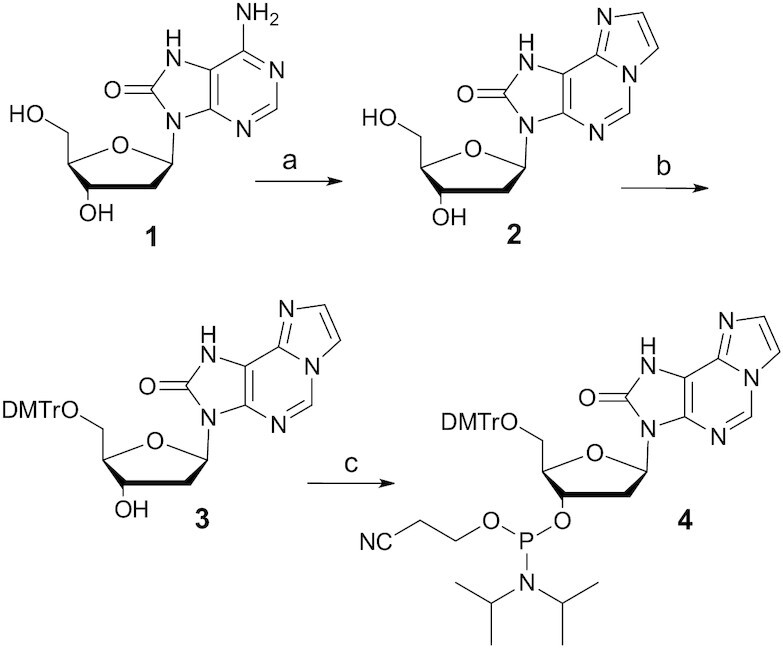
Synthesis of oxo-ϵA phosphoramidite. Reagents and conditions: (a) chloroacetaldehyde, aq. sodium acetate buffer, pH 4.6, 84%; (b) DMTr-Cl, pyridine, 79%; (c) NCCH_2_CH_2_OP(Cl)N-*i*Pr_2_, DIPEA, CH_2_Cl_2_, 81%.

Oligonucleotides were synthesized by using phosphoramidite chemistry, purified by HPLC and characterized by LC–MS. The phosphoramidite **4** was prepared in three steps (Scheme 1). Treatment of 2′-deoxy-7,8-dihydro-8-oxoadenosine **1** (42) with chloroacetaldehyde gave nucleoside derivative **2**, which was successively 5′-*O*-dimethoxytritylaled and 3′-*O*-phosphitylated. Detailed synthetic procedures and NMR/MS data are presented in Supporting Information (Materials and Methods).

**Figure 2.**
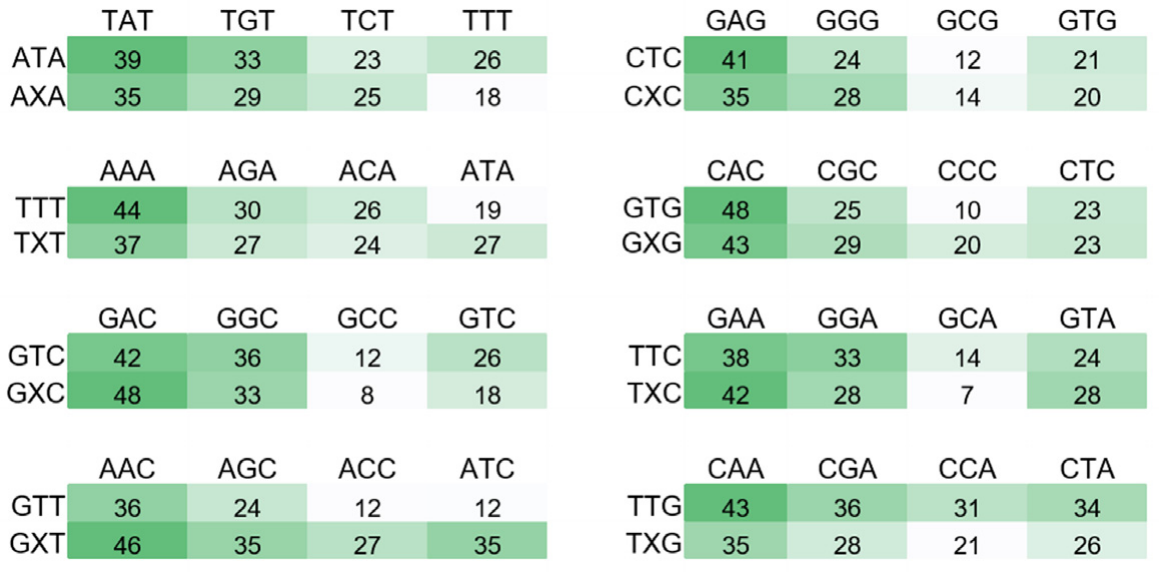
Heat maps summarizing relative thermal stabilities (*T*_m_± 2°C) of the duplexes formed by a native (5′-GCANTNTACG-3′) or modified (5′-GCANXNTACG-3′, X = oxo-ϵA) strand and a complement (5′-CGTANNNTGC-3′) (for detailed information, see Supplementary Table S1).

To assess the effects of oxo-ϵA on DNA duplex stability, we synthesized eight modified homologous decamers (ODN1–ODN8) with different central triplets (the triplets are schematically shown in Figure 2; for full sequences, see Supplementary Table S1). By using UV melting and CD spectroscopy, we evaluated the effect of oxo-ϵA on duplex stability in oligonucleotides in which the modification was situated in eight flanking base contexts, and where the modification was matched with all possible pairing partners.

Based on the T_m_ values derived from melting curves (Supplementary Figure S1), we obtained a ‘heat map’ of relative duplex stabilities (Figure 2) and calculated the modification-induced stabilization/destabilization of the duplex (Δ*T*_m_) (Supplementary Table S2, for thermodynamic parameters of duplex formation from single strands see Supplementary Table S3). According to these data, oxo-ϵA behaves similarly to thymine, the canonical base pairing partner of adenine. At the same time, oxo-ϵA modulates duplex stability in a flanking base-dependent manner and Δ*T*_m_ varies from –7 ± 2°C (TXT) to +10 ± 2°C (GXT) (Figure 2). The hybridization specificity is also context-dependent, with the lowest value observed for the GXT triplet. CD spectra of the duplexes with varied flanks (5′-NXN-3′; where X = oxo-ϵA and N = any base) are close to the 5′-NTN-3′ set and consistent with the typical CD signature of mixed-sequence dsDNA with negative and positive bands around 250 nm and 280 nm, respectively (Supplementary Figure S2). Taken together, the data lead to the conclusion that oxo-ϵA does not substantially alter duplex stability and geometry and likely forms an almost perfect base pair with adenine.

### NMR and molecular modelling of the DNA duplex containing oxo-ϵA support adoption of the syn conformation and formation of a stable base pair with the opposite adenine

**Figure 3.**
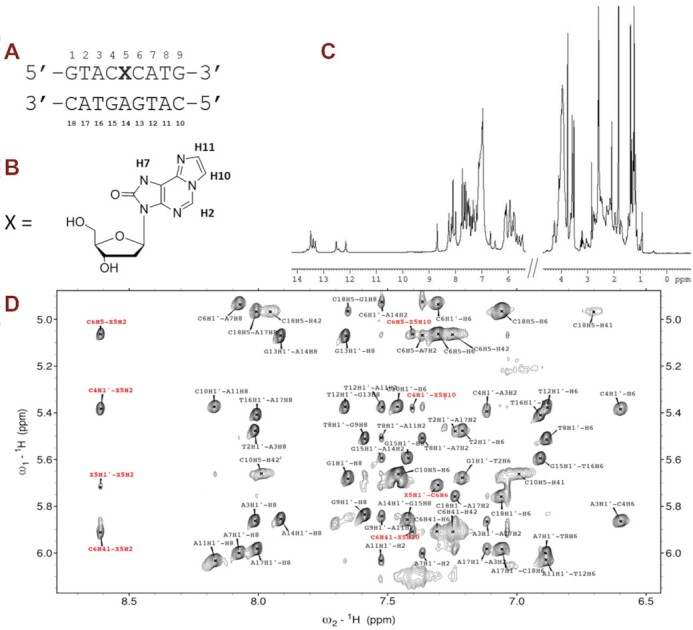
NMR analysis of oxo-ϵA in duplex DNA. (**A)** DNA duplex used in the NMR and MD studies; (**B**) H-atoms numbering for oxo-ϵA nucleoside; (**C**) 1D NMR spectrum; (**D**) H1′-aromatic region of the NOESY spectra (150 ms mixing time, *T* = 3 ºC (X5 proton resonances are marked in red).

**Figure 4.**
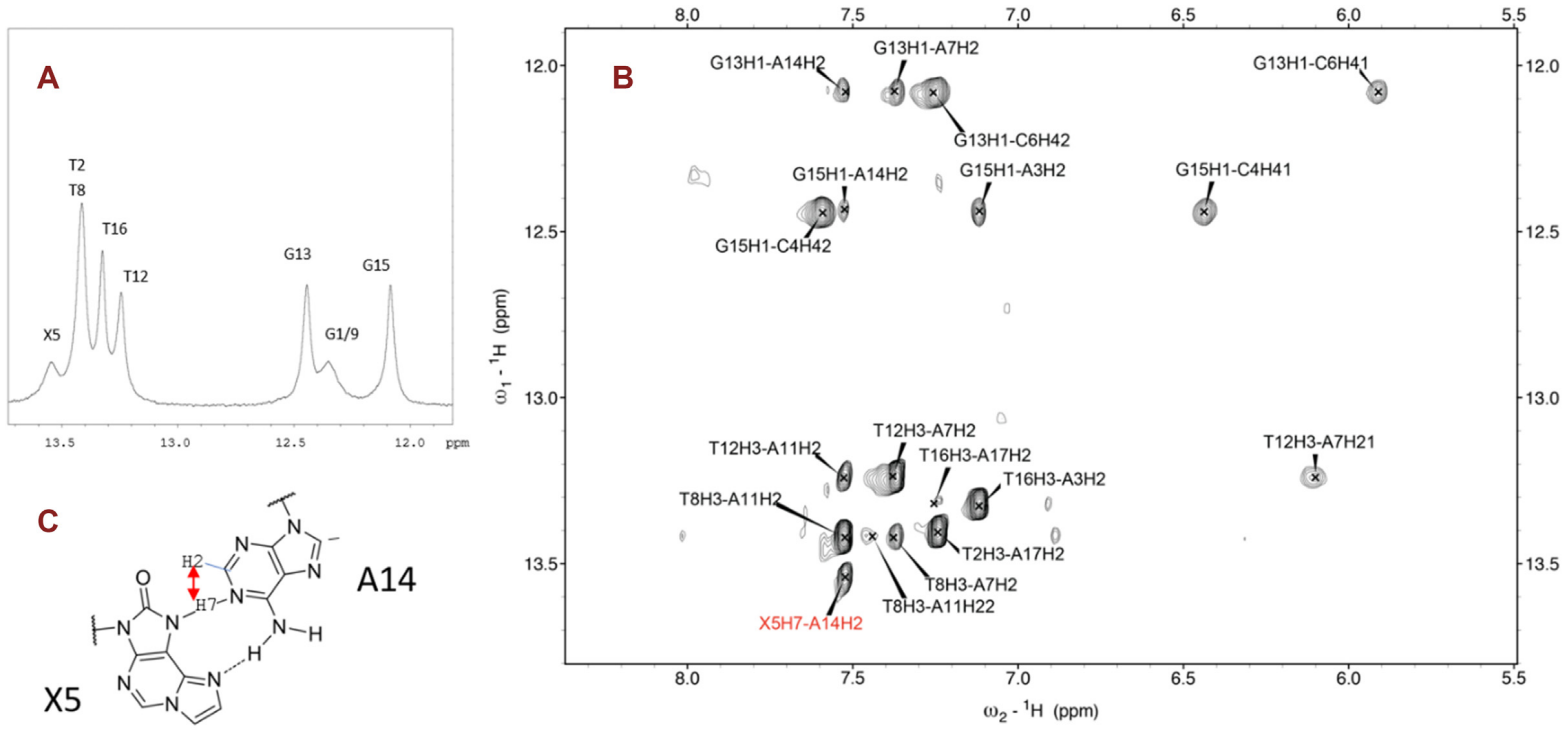
Exchangeable protons region of the NMR spectra (**A**); NOESY spectra (mixing time 50 ms, *T* = 3°C) (**B**). All non-terminal base pairs could be identified. H7 proton of X5 exhibits a clear NOE with H2A14 (labelled in red), confirming the proposed base paring X5A14 shown in (**C**).

To uncover the structural basis underlying the unusual selective pairing of oxo-ϵA with an opposing strand adenine, we performed NMR studies that were further refined using molecular simulations. We synthesized the 9-mer DNA duplex used previously to determine the ϵA conformation opposite T or G (32,43), but the central base pair was replaced with oxo-ϵA:adenine (**X**:A) (Figure 3A, B). The 1D NMR spectrum is shown in Figure 3C, and the detailed assignment of protons in Supplementary Table S4. Non-exchangeable protons were assigned using established techniques for right-handed, double-stranded nucleic acids using DQF-COSY, TOCSY and 2D NOESY spectra. The base-H1′ region is illustrated in Figure 3D. Three signals in the guanine imino protons region are observed in the NMR spectra (Figure 4A). Two of the signals could be assigned to G13 and G15 by following their connection to H5 of cytosines (H5C→HN4C→H1G) involved in G:C base pairs (Figure 4B). Most probably, the broad signal at 12.53 ppm (Figure 4A) corresponds to the terminal guanines (G1 and/or G9) which is further confirmed by the ^1^H-NMR melting experiment (Supplementary Figure S5). Thymine imino H3 protons were identified by their strong inter-strand cross-peaks with H2 of their base-paired adenines. NOE connectivities between imino protons confirmed the sequential assignment of all imino and most amino protons of non-terminal base pairs. The cross-peak patterns observed indicate that all bases are forming Watson–Crick base pairs. The intensities of intra-residual H1′–H6/H8 NOEs indicate that the glycosidic angles of all the non-modified bases, including A14, are in anti conformation (see Figure 3D). Many NOEs involving protons of oxo-ϵA residue could be detected (Supplementary Figures S3 and S4). Of particular importance are the NOEs H7X5-H2A14 (Figure 4B) and H11X5-H6A14 (Supplementary Figure S4), supporting the oxo-ϵA:A base pair shown in Figure 4C. The chemical shift of one of the A14 amino protons (9.62 ppm) is also indicative of being involved in a hydrogen bond.

**Figure 5.**
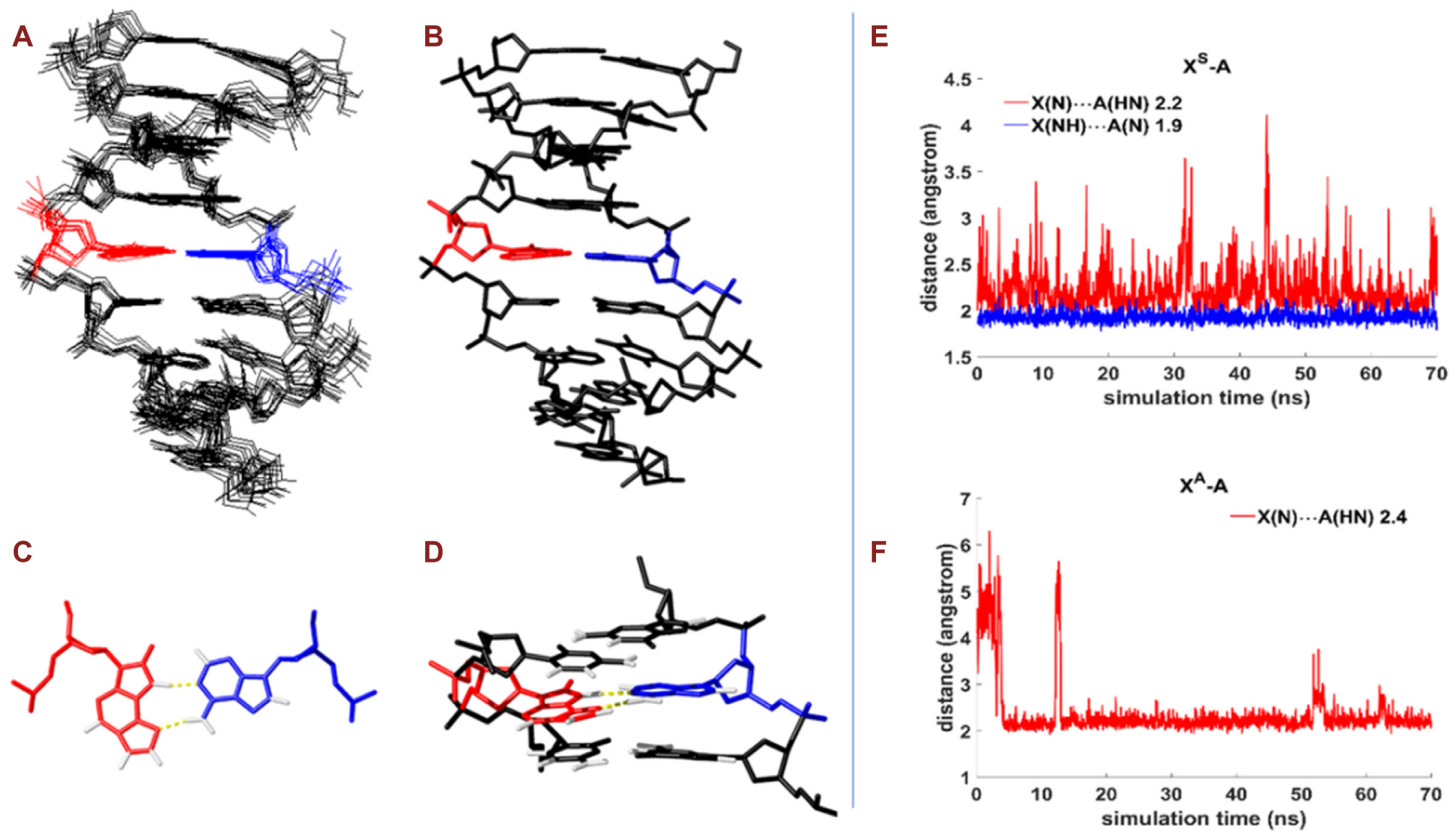
Ensemble of 10 resulting structures determined form NMR data (**A**) (7NBP). Averaged solution structure (**B**). Detail of the X5:A14 base pair (**C**), and their neighboring base pairs (**D**). X5 is shown in red, and A14 in cyan. The distance between hydrogen atom of H-bond donor and nitrogen of H-bond acceptor in X^S^–A (**E**) and X^A^-A (**F**) base pairs.

The solution structure of the modified duplex was determined on the basis of 196 experimental distance constraints derived from NOESY experiments. The structure is well-defined, with an RMSD for all heavy atoms below 0.7 Å. Statistical data are summarized in Supplementary Table S5 and the structure ensemble is shown in Figure 5A. Overall, the DNA structure is almost a standard B-form double helix. The modified oxo-ϵA base adopts a syn conformation (χ = 65º) and fits very well within the surrounding residues without inducing distortions in local DNA architecture (see Figure 5D). Short distances and appropriate geometries indicate the formation of two hydrogen bonds between oxo-ϵA (X5) and A14 (N4X5-H61A14 and N1A14-H2X5), as shown in Figure 5C. This structure is supported by numerous experimental NOEs involving oxo-ϵA (X5) protons, shown in Supplementary Table S6. Final structures are deposited in the PDB (7NBP) and their geometrical parameters are shown in Supplementary Table S7. The resulting structure is very similar to a standard B-form duplex with only very minor distortions. A comparison with a canonical B-duplex with an A:T base pair is shown in Supplementary Figure S6. Oxo-ϵA fits very well within the surrounding nucleobases. The minor changes in the backbone observed are sufficient to mitigate potential steric clashes due the syn conformation the oxo-ϵA residue.

Next, computational modeling was performed to evaluate the stability of the oxo-ϵA:adenine base pair. We compared syn (X^S^) and anti (X^A^) conformations of oxo-ϵA in the DNA duplex used for NMR studies. Distances between NH donors and N acceptors and initial and final conformations for both variants are shown in Figure 5E-F and Supplementary Figure S7. For the syn conformation of oxo-ϵA, two NH-N hydrogen bonds can exist with adenine, while only one H-bond is available in case of the anti orientation (Supplementary Figure S7, Supplementary Table S8). At the same time, the contribution of electrostatic interactions was higher for the syn variant (Supplementary Figure S8B). In the case of the anti conformation, the aromatic part of the X^A^:A pair does not perturb stacking with neighboring bases (Supplementary Figures S7 and S8C), but causes significant positive stress and deformations of the sugar-phosphate backbone (Supplementary Figure S8E). Thus, the calculated free energy is much lower for the syn conformation, mainly due to the Coulombic contributions and stress energies (Supplementary Figure S8A). As a result, we conclude that the anti conformation of oxo-ϵA is not likely to exist in a B-form DNA duplex. Taken together, the NMR and molecular dynamics simulation data are consistent with the UV melting and CD spectroscopy data and confirm that oxo-ϵA functions as a close mimic of thymine.

### *In vitro* bypass of oxo-ϵA by DNA polymerase I, Klenow fragment or T4 DNA polymerase

First, we carried out primer-extension experiments, using native DNA (**ODN11** with native T at the 7-position) or modified DNA template (**ODN12** with oxo-ϵA residue at the 7-position), a primer bearing 5′-terminal Cy5 residue (**ODN13**) and the large fragment of *E. coli* DNA polymerase I (Klenow fragment) or T4 DNA polymerase (44). For both polymerases, after a 4 h primer extension reaction under optimal conditions, an extension product was found only in the presence of all four nucleoside triphosphates (dNTPs), whereas in the absence of deoxyadenosine triphosphate (dATP), the reaction did not yield any measurable extension product (lanes 2 and 3, 4 and 5, respectively in Supplementary Figure S9, upper panel). Both polymerases were equally efficient in primer elongation on the unmodified template **ODN11**, and T4 DNA polymerase also effectively extended the primer on modified template **ODN12** within 1 h. In turn, we did not observe complete primer elongation with the Klenow fragment on modified template even after 4 h, suggesting that oxo-ϵA can be a partial replication blocker for some polymerases *in vitro*. MS analysis showed the presence of a fully elongated product for the both polymerases with the incorporation of a deoxyadenosine residue opposite oxo-ϵA, as well as certain intermediate products of the extension reaction (see Supplementary Figure S9, lower panel).

### Evaluation of oxo-ϵA base pairing properties in living cells

Oxo-ϵA is not known to be a naturally formed pre-mutagenic lesion, but studies of how it is processed *in vivo* are relevant for two reasons. First, oxo-ϵA applications in biotechnology would be limited if it were to be repaired inside cells. And second, as a fundamental issue, it was of interest to see if observed mutational outcomes would be in alignment with expectations from the spectroscopic, modelling and *in vitro* data that indicate a syn-oxo-ϵA:adenine base pair.

The spectroscopic data show the ability of oxo-ϵA to form a stable base pair with adenine. Here, we consider oxo-ϵA as a modification of the cognate adenine base, and thus, any base-pairing behavior deviating from that of normal adenine is considered mutagenic. In this context, we anticipated, based on the spectroscopy data, that oxo-ϵA was very likely to induce mutations. Because oxo-ϵA is a hybrid combining the structural features of oxoA and ϵA, we expected that the mutagenic properties of oxo-ϵA might reflect the mutagenic features of either one or both lesions. In *E. coli*, the repair of ϵA in single-stranded DNA is performed primarily by AlkB, a direct DNA damage reversal enzyme that removes the etheno bridge by oxidation and restores the canonical base, adenine, within the DNA sequence (45). Less is known about repair of oxoA *in vitro*, but the bacterial MUG enzyme (mismatch uracil glycosylase) is able to remove oxoA from duplexes, primarily when mispaired with C, but less able to repair the lesion in an oxoA:A pair (46). In eukaryotes, oxoA is repaired by the OGG1 glycosylase, a component of the base excision repair pathway (BER) (47); the TDG glycosylase also plays a role, but, as with the bacterial MUG, repair is dependent on the type of mismatch (46). With regard to oxoG, MutM, the bacterial homolog of human OGG1, removes oxoG paired with C, while the MutY glycosylase prevents the mutagenic outcome of oxoG by removing A from the oxoG:A pair (48,49). Given that both MutM and MutY have a broad substrate specificity (50), we decided to probe both repair pathways - direct reversal by AlkB, and MutM/MutY-mediated BER to examine the mutagenic properties of oxo-ϵA.

### Oxo-ϵA is not successfully repaired by the *E. coli* base excision repair machinery

**Figure 6.**
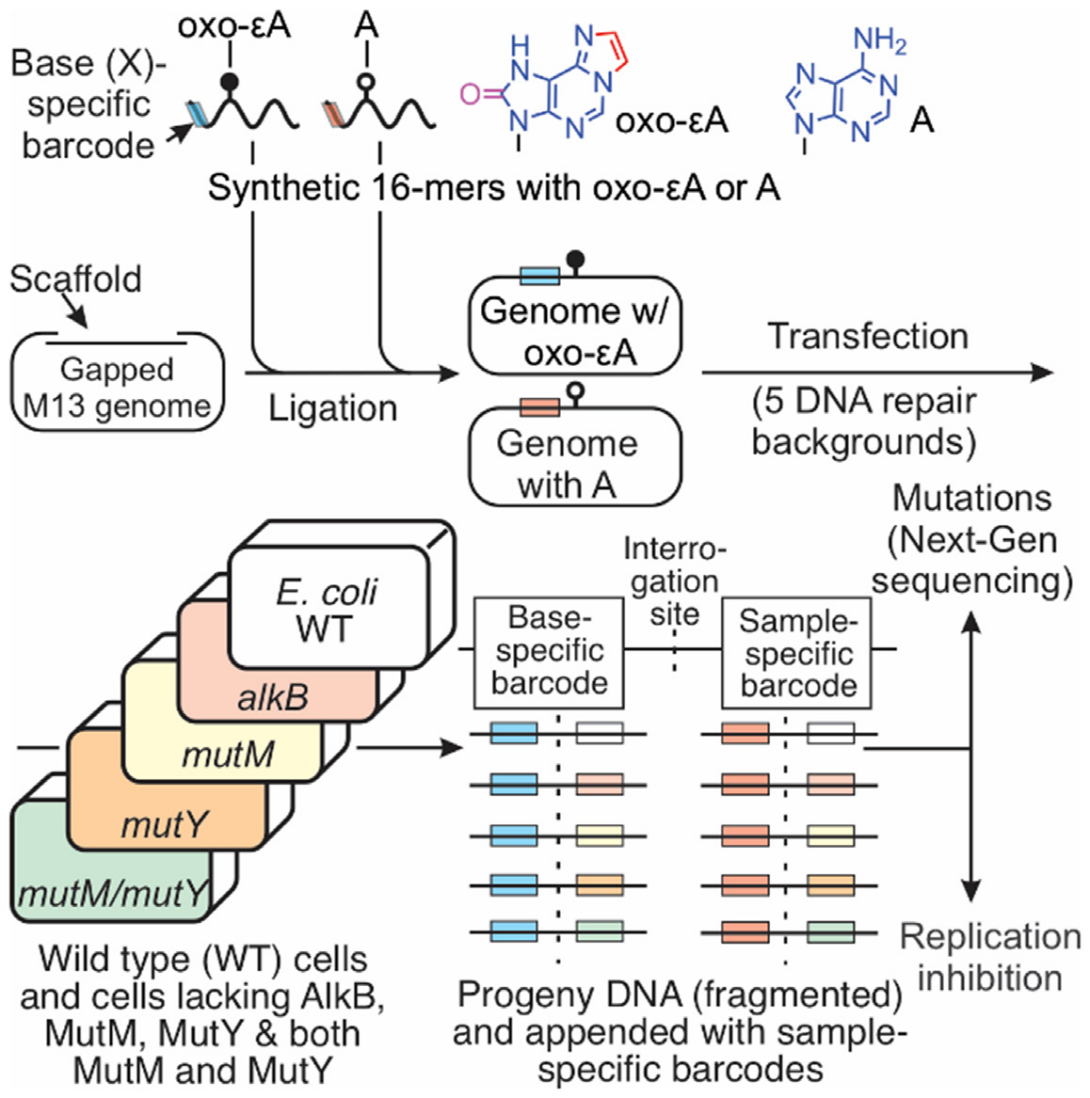
Schematic representation of the *in vivo* mutagenesis assay with NextGen sequencing. X is either A (adenine) or oxo-ϵA at the lesion site. The colored box to the left of the interrogated site symbolizes the lesion-specific trinucleotide barcode; the box to the right of the interrogated site represents a second barcode introduced during Illumina library preparation (indexing barcode), corresponding to each sample.

**Figure 7.**
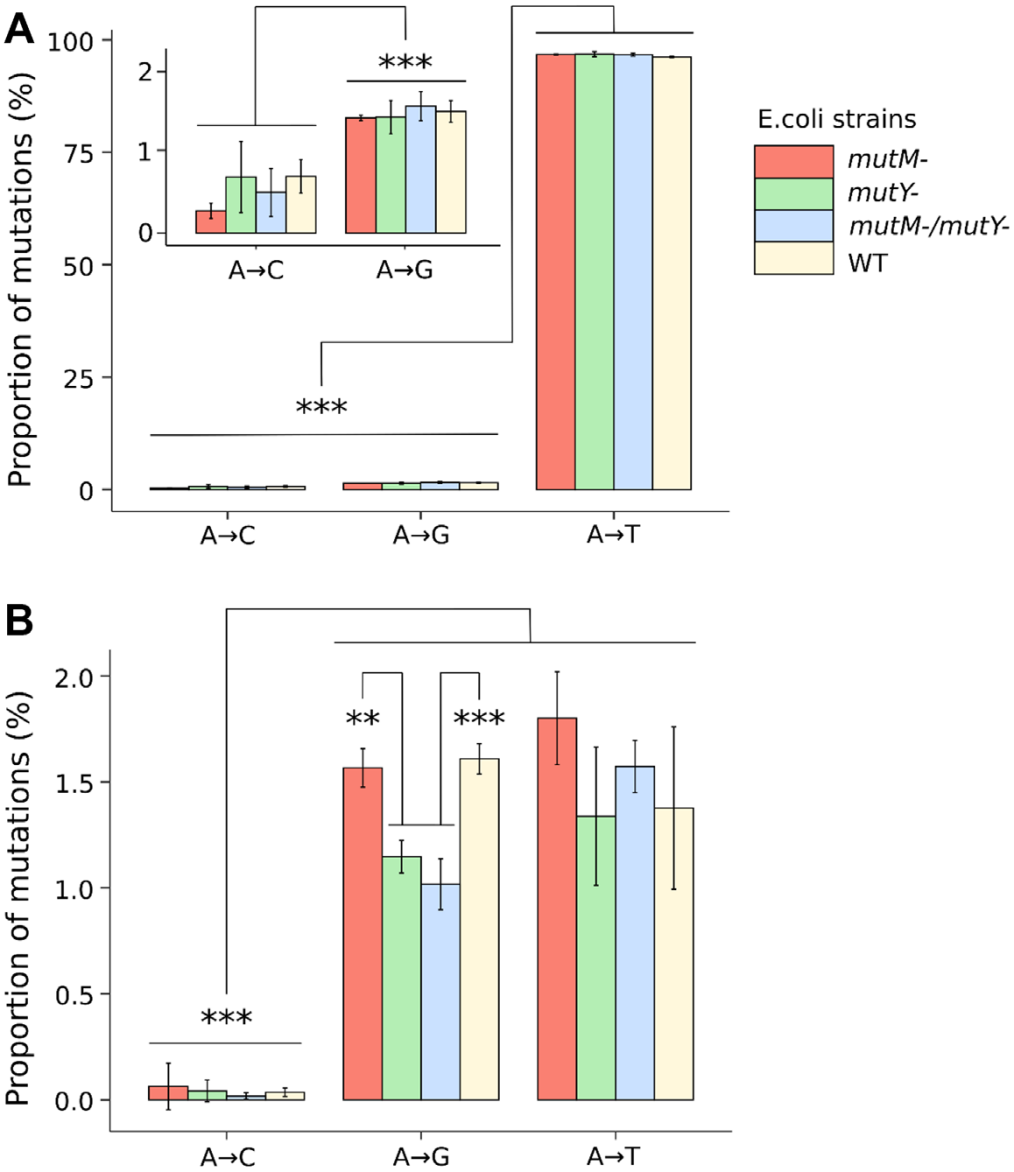
Nucleotide composition at the lesion site in M13 phage DNA after replication in *E. coli* cells either proficient or deficient in MutM, MutY or both (**A**) oxo-ϵA lesion (**B**) control adenine. X-axis denotes a type of mutation; y-axis indicates the relative quantity of a given base at the lesion site.

**Figure 8.**
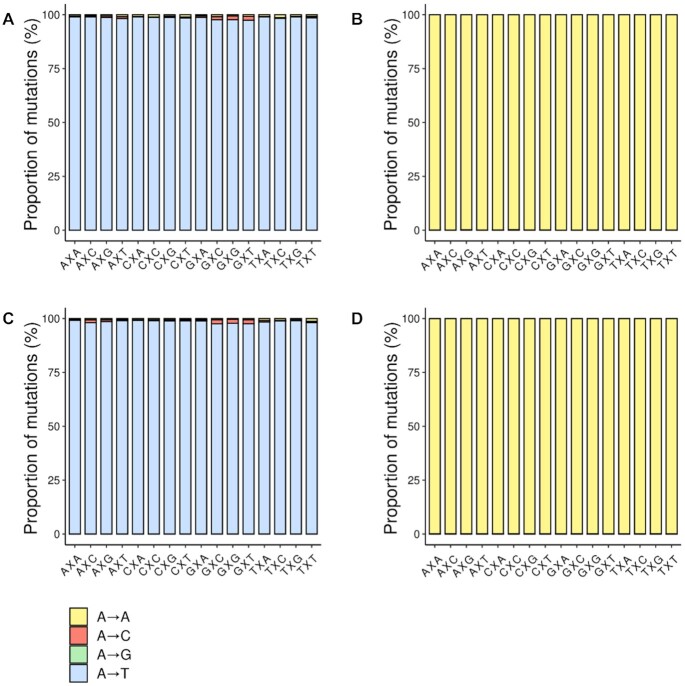
Sequence-context dependent mutagenic potential of oxo-ϵA in *E. coli* cells possessing and lacking the AlkB protein. (**A**) Mutagenesis of oxo-ϵA in the WT strain (*alkB^+^*). (**B**) Mutagenesis of the unmodified A control genome in the WT strain (*alkB^+^*). (**C**) Mutagenesis of oxo-ϵA in *alkB* (AlkB-deficient) cells. (**D**) Mutagenesis of the A control in the *alkB* strain. The x-axis shows the trinucleotide contexts in which the modification or control bases were evaluated. The y-axis denotes relative percentage composition of a base at the lesion site (with respect to the total coverage).

To assess mutagenic potential of oxo-ϵA in MutM/MutY-proficient and -deficient strains of *E. coli*, we took an advantage of the high-throughput sequencing technology developed in our lab whereby the mutagenic properties of a site-specific DNA lesion can be probed in parallel in multiple bacterial strains and/or sequence contexts using multiplexing and NGS (Figure 6) (41). In brief, M13 single-stranded vectors with either A or oxo-ϵA at a defined position in the 5′-TXG-3′ sequence context and a specific barcode sequence, are mixed in a 1:1 ratio and introduced by electroporation into wild type (WT) *E. coli* or MutM^–^, MutY^–^, MutM^–^/MutY^–^ strains. After *in vivo* replication, progeny DNA from each repair background was isolated, the 1 kb fragment around the lesion site was PCR amplified and fragmented to generate sequencing libraries, where each individual sample was given a second set of specific Illumina barcodes. This step allowed us to pool all samples and sequence them in a single run on an Illumina MiSeq instrument. NGS provides data on the mutagenicity of oxo-ϵA as a function of genetic background (the detailed procedure is described in Supporting Information). The percentage of mutations is given in Supplementary Table S9, and statistical comparison between genotypes using ANOVA with the Tukey post-hoc test is given in Supplementary Table S10. The results show that oxo-ϵA is ∼97% mutagenic, independent of the MutM/MutY status of the host cell in which the modification is replicated (Figure 7A), compared to the adenine control (Figure 7B). Notably, 95% of all mutations induced by oxo-ϵA are A→T transversions, in alignment with our predictions based on structural and physicochemical characteristics of oxo-ϵA in DNA. The results suggest that an oxo-ϵA:A mispairing in the TXG context is unlikely to be a substrate for the MutM glycosylase (Figure 7A). Unexpectedly, in the experiment with oxo-ϵA in 16 contexts, there are nearly 2% of oxo-ϵA→C mutations in the GXC, GXG and GXT contexts (Figures 8A and C, Supplementary Figures S11A and S11C), implying that in these cases oxo-ϵA could be paired with G and could become a substrate for MutM, as MutM can excise oxoG paired with G (51). However, even if MutM removes oxo-ϵA from oxo-ϵA:G pairs, in further repair reactions C will be incorporated opposite to G leading to the A→C mutation observed in the GXC, GXG and GXT contexts, and therefore, the potential activity of MutM against oxo-ϵA:G mispairing will be camouflaged.

The same situation seems to apply to MutY. Even if it recognizes oxo-ϵA:A pairings and successfully removes A from that pair, in the next rounds of repair, this A will be replaced by another A, and the mutagenic potential of oxo-ϵA will not be decreased. Therefore, we cannot completely exclude the opportunity of oxo-ϵA recognition by MutM or MutY, but their activity will be unnoticed due to the futile cycle of further incorporation of the complementary base, which will finally lead to nearly 100% mutagenesis of oxo-ϵA even in WT cells.

### Oxo-ϵA is not recognized by an *E. coli* direct reversal repair system, independent of the nucleotide context

The experiments above were conducted in a single sequence context around the lesion site, namely 5′-TXG-3′. Since mutagenesis and DNA repair can be context-dependent (52,53). It is possible that the choice of sequence context may partially account for the high mutation rate and low extent of repair observed. To address this issue, we set out to investigate the mutagenic properties of oxo-ϵA in all 16 possible 3-base sequence contexts, as well as to determine the contribution of the direct reversal repair enzyme AlkB, which is very effective at repairing ϵA. We constructed 32 single-stranded M13 vectors, each containing either adenine or oxo-ϵA in one of 16 trinucleotide contexts, and a trinucleotide barcode corresponding to adenine or oxo-ϵA (detailed description is given in Supporting Information). Sixteen adenine-containing genomes were mixed in an equimolar proportion into a control pool, and 16 oxo-ϵA-containing genomes were prepared as the experimental pool, and electroporated into AlkB-proficient and AlkB-deficient strains (HK81 and HK82, respectively). The subsequent steps, including phage replication, DNA isolation, PCR amplification, sequencing and data analysis, were performed as described above. The percentage of mutations is given in Supplementary Table S11 for AlkB deficient strain, and the results of statistical comparison between sequence contexts are provided in Supplementary Table S12. Consistent with the previous experiment, we found that oxo-ϵA is nearly 100% mutagenic in both the WT strain (Figure 8A, Supplementary Figure S11A) and the AlkB-deficient strain (Figure 8C, Supplementary Figure S11C). Moreover, high levels of miscoding are seen uniformly across all sequence contexts. The dominant mutation is the A→T transversion (98.5%), which is consistent with the hypothesis that oxo-ϵA in the syn conformation strictly pairs with dATP during replication. The control genome, featuring a normal A in all sequence contexts shows only a background level of mutations, well in line with the sensitivity and level of noise of the method (Figures 8B and D; Supplementary Figures S11B and D).

### Evaluation of oxo-ϵA genotoxicity in *E. coli*

Lesions such as ϵA are very toxic to replication, so it was of interest to determine the level of genotoxicity associated with replication of DNA containing oxo-ϵA. One established method of estimating the ability of the DNA lesion to inhibit replication is the competitive replication of adduct bypass (CRAB) assay based on mixing the lesion-bearing genome with a non-lesion competitor genome prior to transfection and calculating the replication blocking ability from the decrease in the lesion-to-competitor output signal ratio (40). More recently, we have shown that the same result can be inferred from comparing the number of sequenced fragments (i.e. sequencing reads) corresponding to the lesion-containing genomes and lesion-free control genomes; when starting with an equimolar mix of genomes, the ratio between the number of sequencing reads provides a direct estimate of the bypass efficiency, similarly to the traditional CRAB assay (41). We electroporated an equimolar mixture of adenine- and oxo-ϵA-bearing genomes into MutM/MutY-proficient and -deficient strains of *E. coli*, which allowed us to use the adenine-bearing genome as a competitor for oxo-ϵA. In these settings, a post-replicative ratio of lesion genome to control genome equal to 1 reflects 100% bypass. Distribution of the coverage at the lesion site (Supplementary Figure S10) showed no statistically significant difference between the number of reads corresponding to the adenine control genome and to the oxo-ϵA genome, indicating that oxo-ϵA does not impact the efficacy of replication in any of the *E. coli* strains analysed.

## DISCUSSION

We describe here a novel DNA modification, 7,8-dihydro-8-oxo-1,*N*^6^-ethenoadenine, which possesses a precise arrangement of H-bond donors and acceptors that enables pairing between its Hoogsteen face and an opposite adenine in the double helix. Oxo-ϵA, being a combination of two naturally occurring forms of DNA damage, oxoA and ϵA, was anticipated to be mutagenic due to the predicted ability to interact with adenine. Thorough evaluation of the influence of oxo-ϵA on DNA duplex thermal stability confirmed the hypothesis that predicted a thymine-like behaviour of oxo-ϵA (Figure 2). The melting temperature trend for oxo-ϵA:A within eight nucleotide neighboring contexts (AXA, TXT, CXC, GXG, GXC, GXT, TXC and TXG) was consistent with data on the T:A pair. Moreover, the p*K*_a_ of the N7 atom of the model oxo-ϵA derivative (9-ethyl-8-oxo-ϵA), which acts as an H-bond acceptor, is equal to 9.49 (54). This value is close to the pKa of the thymine N3 atom and, therefore, the modification mimics well enough thymine from the point of view of H-bond proton donating ability (55). In agreement with CD spectroscopy results, the NMR structure clearly shows that the oxo-ϵA:A base pair does not provoke major distortions in the local duplex structure. Additional evidence of the oxo-ϵA syn conformation was provided by molecular modelling showing that oxo-ϵA in the anti conformation should lead to significant deformations of the sugar-phosphate backbone. Thus, oxo-ϵA in the anti conformation is an unlikely occurrence in DNA duplex (Supplementary Figure S8A). Given its base-pairing preference, oxo-ϵA constitutes a purine that functions as a structural and functional thymine mimic in DNA duplexes.

Many DNA lesions are mutagenic because they undergo rotation about glycosidic bond and can interact during replication with non-classically complementary opposite-strand nucleobases. Furthermore, transient Hoogsteen base pairs exist even in canonical duplex DNA (56,57) and can contribute to specific DNA-protein interactions and formation of non-canonical DNA structures (e.g. G-quadruplexes). So, oxo-ϵA can be thought of as a limit case where the equilibrium is completely shifted towards a Hoogsteen base pair (Figure 1E). To test the hypothesis that a combination of chemical modifications of adenine can fix the syn conformation of the nucleobase, we studied its mutagenic properties. We found that oxo-ϵA in DNA overwhelmingly pairs with adenine during elongation *in vitro* and replication *in vivo*, which, when considering A as the reference base, leads to a predominant A→T mutational outcome. Our *in vivo* studies indicate that oxo-ϵA is extraordinarily mutagenic in *E. coli*, independent of the trinucleotide sequence context. While the chemical parents of oxo-ϵA (oxoA and ϵA) are individually vulnerable to repair by BER and AlkB, respectively, their combination in a single molecule is a powerful mutagen that cannot be repaired by these systems. Because some DNA lesions are repaired in a sequence context-dependent manner, we thought that the potent mutagenic properties and resistance to DNA repair might be attributable to the 3-base context in which the lesion was present in the genome. As one example, context-dependent DNA repair *in vivo* is observed with the methylated base, *O*^6^-methylguanine (52). We found that oxo-ϵA causes A→T transversions in all 16 possible trinucleotide contexts with almost equal efficacy in repair-proficient WT cells and in cells lacking AlkB (Figure 8). Thus, oxo-ϵA is a potent mutagen in all possible contexts with an inherent mutation frequency above 97%. Such strong mutagenicity together with low genotoxicity suggests that oxo-ϵA pairs with adenine as efficiently as thymine—the canonical adenine partner—and cannot be detected as DNA damage by *E. coli*. The lack of genotoxicity is remarkable and probably reflects the ability of the modification to masquerade as thymine, allowing facile polymerase bypass, as well as being invisible to repair systems.

**Figure 9.**
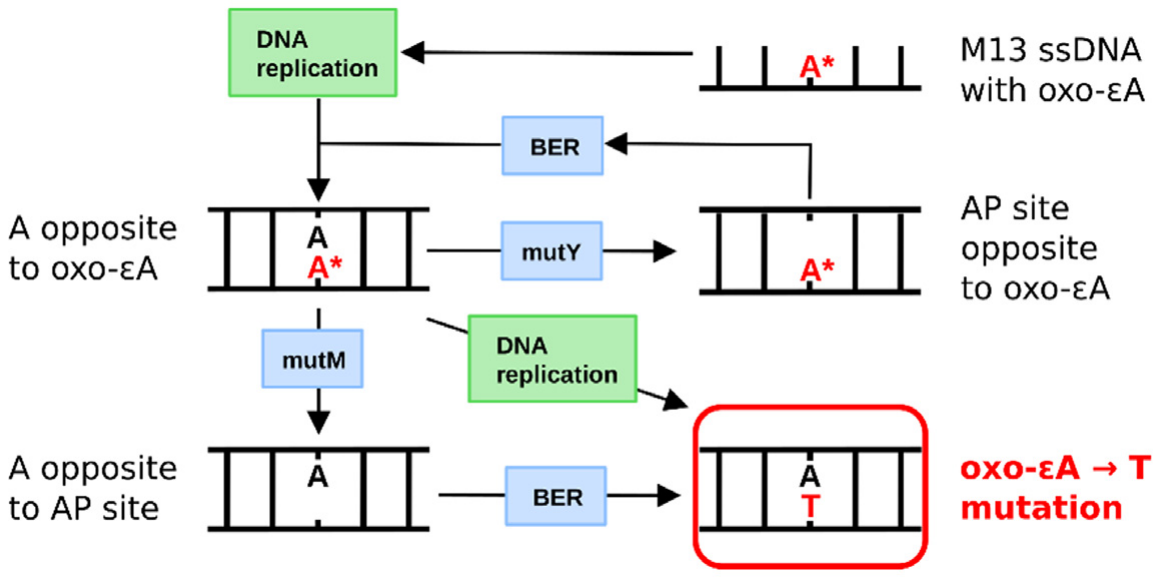
Proposed scheme of oxo-ϵA-driven mutagenesis in *E. coli* and the involvement of the BER pathway. AP site is an apurinic site.

It is puzzling that oxo-ϵA is not an obvious substrate for common repair pathways in *E. coli*. However, it should be borne in mind that DNA with oxo-ϵA in our study was single-stranded when entering the cell, and therefore, some repair factors might not be fully operative against single-stranded DNA. It is known that both MutM and MutY will ignore oxo-ϵA in single stranded DNA but, once replication occurs, oxo-ϵA predominantly exists in an oxo-ϵA:A base pair (Figures 4 and 5). If this pair is a substrate for MutM, this enzyme will excise oxo-ϵA and leave behind the A on the opposite strand, which would direct other BER enzymes to place a T opposite this A, leading to the observed A→T mutations. Similarly, if the oxo-ϵA:A pair is recognized by MutY, which normally excises A from oxoG:A mismatches, the A will be removed leaving an abasic site opposite oxo-ϵA. It is possible that the BER polymerase (pol *I*) would repeat the mistake and place A once again across from oxo-ϵA, leading to the same mutagenic outcome, or worse, futile cycling and delayed phage replication (Figure 9). Taken together, the *E. coli* MutM and MutY glycosylases are at best inactive against oxo-ϵA, and at worst, they may even facilitate mutation formation rather than participate in lesion repair. These considerations are consistent with the data of primer elongation *in vitro* and the proposed model that oxo-ϵA is smoothly bypassed without attendant toxicity and mostly pairs with adenine, yielding the almost quantitative A→T mutation observed.

Another DNA repair pathway that could operate on oxo-ϵA is AlkB-mediated direct damage reversal (45). The Fe(II)- and alpha-ketoglutarate-dependent dioxygenase AlkB recognizes ϵA and directly removes its etheno bridge to restore adenine in both single-stranded and double-stranded DNA. The innate activity of AlkB renders ϵA non-mutagenic in WT *E. coli* (34,58). However, in the present study, the mutagenic ability of oxo-ϵA was unaffected by the AlkB status of the cells (Figure 8). This observation indicates that the addition of the C8 oxygen to the ϵA lesion eliminated the affinity of AlkB to oxo-ϵA. We note that AlkB-mediated repair requires coordination of Fe^2+^ atom between two oxygens of alpha-ketoglutarate and two oxygens of Asp133 of the enzyme (45). For oxo-ϵA the presence of the extra oxygen on C8 may disrupt this coordination environment, as the protonated N7 would fail to build a bridge with Q132, crucial for positioning of the damaged base in the enzyme active site (59). As AlkB is a fast enzyme that repairs ϵA in both single-stranded and double-stranded DNA, the syn conformation of the lesion should not influence the repair process (60). Yet another possibility is that even if the AlkB catalyzed oxidation occurs, the epoxide and diol intermediates may be longer-lived compared to the intermediates of the ϵA reaction with AlkB. Regardless of these possible scenarios, our data show that AlkB activity is unable to prevent the oxo-ϵA:A mispairing and its subsequent mutagenic outcome.

In addition to direct damage reversal and base excision, one could speculate that oxo-ϵA could be repaired by other DNA repair systems, such as nucleotide excision repair (NER) and mismatch repair (MMR). NER recognizes the distortions in the DNA double helix caused by the DNA lesions, rather than the lesions themselves (61). However, oxo-ϵA placed opposite adenine in the DNA duplex did not significantly alter the geometry and stability of helix, and therefore, it is unlikely that NER could contribute to the repair of oxo-ϵA in our settings.

The *E. coli* MMR system recognizes a mismatched base and nicks the strand with a wrong base to initiate the excision reaction. This system is highly efficient in reducing G:C→T:A transversions and moderately efficient in preventing A:T→T:A transversions (62), and its proximal enzyme, MutS, was shown to recognize 8-oxoG incorporated from the nucleotide pool into the nascent DNA chain (63). MMR determines the newly synthesized strand by the absence of adenine methylation at a GATC site. [reviewed here (64)]. The single-stranded M13 phage DNA used in our experiments to replicate lesion-bearing and control nucleotides in *E. coli* is not methylated, and theoretically any strand—either the original or complementary one—could be considered as a ‘nascent’ strand by MMR, and therefore could be repaired via MMR. The opportunity of either DNA strand repair was demonstrated on the *E. coli* cells with inactivated *dam* gene responsible for DNA methylation (65). However, as oxo-ϵA pairs with A, whichever strand is excised, the result will be the same: an A:T→T:A mutation at the lesion position. Therefore, even if MMR would act upon the oxo-ϵA:A pairing, its effect would be undistinguishable.

One additional important and interesting feature of oxo-ϵA is its high efficiency of replication. Unlike other bridged adenine lesions such as ϵA, 1,*N*^6^-ethano-adenine and 1,*N*^6^-butadiene-adenine, which are strong replication blockers and not very mutagenic (29,66,67), oxo-ϵA is highly mutagenic and does not affect replication rates in *E. coli* (Supplementary Figure S10). Once again, the best mechanistic explanation is that, unlike other alkyl adenine lesions, oxo-ϵA is seamlessly accommodated within the DNA duplex without provoking significant distortions that could disrupt DNA polymerase activity (Figure 5) or attract the attention of the repair enzymes. The 8-oxo group of oxo-ϵA and/or the protonated N7 offer the possibility of a new hydrogen bond (with an incoming A), a bond that does not exist for any of the aforementioned adenine lesions. Hence, we cannot exclude the possibility that oxo-ϵA can be recognized as a non-instructional base/lesion because, as a default, bacterial DNA polymerases tend to insert A opposite to non-instructional lesions and AP sites (the A-rule) (68). This phenomenon would also account for the dominant A→T transversion that we observe here. However, in most cases the A-rule is invoked only when bypass polymerases are induced (the SOS response). While various stresses can induce the SOS response, common electroporation techniques that we used in this study, are known to be safe and pose no risk of genotoxicity (69). Thus, our results do not support the potential contribution of SOS‐inducible DNA polymerases in the observed A→T transversions.

The absence of a repair system for oxo-ϵA in *E. coli* together with the efficient replicative bypass of the lesion can offer unique opportunities for using oxo-ϵA as a synthetic model base for applications in biotechnology and medicine. For example, oxo-ϵA could be used for studying cellular repair processes (70) or developing MutY/MutT specific inhibitors that may find utility as antitumor drugs (71–73). In addition, it could find utility in constructing thermodynamically stable and high affinity aptamers and other complex supramolecular DNA structures to target living cells and organisms. Aptamers are single stranded DNA and RNA oligonucleotides that can fold into defined architectures and, like antibodies, can recognize other types of molecules, including metal ions, small molecules, toxins, proteins and DNA *in vitro* and *in vivo* (74–76). Since oxo-ϵA has an extended aromatic system which stacking properties differ from thymine according to our UV melting studies, it can provide desirable hydrophobic and stacking contacts with target nucleic acids, proteins, peptides or small molecules, while maintaining the thymine-like H-bonding pattern. In this regard, oxo-ϵA can be useful in heavily-modified aptamers like SOMAmers with functional groups absent in natural nucleic acids that can improve SELEX success rates, especially for difficult protein targets (77). As oxo-ϵA can participate in metal-mediated pairing (54), one additional level of complexity can be added to form desired folded non-canonical 3D structures based on nucleic acids. Moreover, the fluorescent properties of oxo-ϵA (54) provide an additional axis for the development of oligonucleotide therapeutics that can be used to visualize unbiased intracellular and intra organismal distribution of nucleic acids (78), analysis of DNA structures and DNA–protein interactions.

## CONCLUSIONS

Oxo-ϵA is a DNA modification that combines two naturally-occurring lesions, 7,8-dihydro-8-oxoadenine and 1,*N*^6^-ethenoadenine. Using UV-, CD- and NMR spectroscopies and molecular modeling, this modification in DNA duplexes was shown to adopt primarily a syn conformation and mimic the base-paring behavior of the natural base thymine. Our molecular design strategy conceptualized a modified nucleobase for which predefined hybridization properties are achieved by blocking ability of one of the rotamers to interact with the opposite nucleobase. Importantly, the modification caused mostly A→T transversion mutations *in vitro* and in *E. coli* as evidence of its thymine mimicry, and was not repaired by either BER enzymes (MutM and MutY) or the direct-reversal AlkB enzyme, which removes the etheno bridge from 1,*N*^6^-ethenoadenine. In addition, oxo-ϵA was not a significant DNA replication inhibitor in *E. coli* and its mutagenic properties did not depend on sequence context. Moreover, the ability of oxo-ϵA to mimic thymine by adopting a syn conformation during replication emphasizes the role of conformational equilibria in nucleic acids in living cells and can be beneficial for multiple practical applications. Indeed, its minimal local distortions of nucleic acid architecture and ability to evade DNA repair systems are assets for those applications. Thus, oxo-ϵA, being a near perfect and fluorescent thymine mimic, will find applications in DNA-based molecular tools to monitor rearrangements and intracellular distribution of DNA and DNA–protein complexes without distortion of DNA architecture and to develop chemically diverse modified aptamers. We expect that the opportunity to stabilize the syn conformation of nucleobase and to fine-tune Hoogsteen base-pairing ability opens up prospects for the creation of similar modified DNA-based molecular tools.

## DATA AVAILABILITY

Atomic coordinates and structural factors have been deposited with the Protein Data bank under accession number 7NBP.

## Supplementary Material

gkac148_Supplemental_File

## References

[1] Anisenko A.N. , Knyazhanskaya E.S., Zalevsky A.O., Agapkina J.Y., Sizov A.I., Zatsepin T.S., Gottikh M.B. Characterization of HIV-1 integrase interaction with human Ku70 protein and initial implications for drug targeting. Sci. Rep. 2017; 7:5649. 28717247 10.1038/s41598-017-05659-5 PMC5514147

[2] Clark J.L. , Hollecker L., Mason J.C., Stuyver L.J., Tharnish P.M., Lostia S., McBrayer T.R., Schinazi R.F., Watanabe K.A., Otto M.J. et al. Design, synthesis, and antiviral activity of 2′-deoxy-2′-fluoro-2′-C-methylcytidine, a potent inhibitor of hepatitis c virus replication. J. Med. Chem. 2005; 48:5504–5508. 16107149 10.1021/jm0502788

[3] Freier S.M. , Altmann K.H. The ups and downs of nucleic acid duplex stability: structure-stability studies on chemically-modified DNA:RNA duplexes. Nucleic Acids Res. 1997; 25:4429–4443. 9358149 10.1093/nar/25.22.4429 PMC147101

[4] Schlegel M.K. , Foster D.J., Kel’in A.V., Zlatev I., Bisbe A., Jayaraman M., Lackey J.G., Rajeev K.G., Charisse K., Harp J. et al. Chirality dependent potency enhancement and structural impact of glycol nucleic acid modification on siRNA. J. Am. Chem. Soc. 2017; 139:8537–8546. 28570818 10.1021/jacs.7b02694

[5] Mayer G. The chemical biology of aptamers. Angew. Chem. Int. Edit. 2009; 48:2672–2689. 10.1002/anie.200804643 19319884

[6] Crooke S.T. , Seth P.P., Vickers T.A., Liang X.H. The interaction of phosphorothioate-containing RNA targeted drugs with proteins is a critical determinant of the therapeutic effects of these agents. J. Am. Chem. Soc. 2020; 142:14754–14771. 32786803 10.1021/jacs.0c04928

[7] Liczner C. , Duke K., Juneau G., Egli M., Wilds C.J. Beyond ribose and phosphate: selected nucleic acid modifications for structure/function investigations and therapeutic applications. Beilstein J. Org. Chem. 2021; 17:908–931. 33981365 10.3762/bjoc.17.76 PMC8093555

[8] Beck J.D. , Reidenbach D., Salomon N., Sahin U., Tureci O., Vormehr M., Kranz L.M. mRNA therapeutics in cancer immunotherapy. Mol. Cancer. 2021; 20:69. 33858437 10.1186/s12943-021-01348-0 PMC8047518

[9] Chen T. , Hongdilokkul N., Liu Z.X., Thirunavukarasu D., Romesberg F.E. The expanding world of DNA and RNA. Curr. Opin. Chem. Biol. 2016; 34:80–87. 27565457 10.1016/j.cbpa.2016.08.001 PMC5107331

[10] Malyshev D.A. , Dhami K., Lavergne T., Chen T.J., Dai N., Foster J.M., Correa I.R., Romesberg F.E. A semi-synthetic organism with an expanded genetic alphabet. Nature. 2014; 509:385–388. 24805238 10.1038/nature13314 PMC4058825

[11] Hirao I. , Kimoto M. Unnatural base pair systems toward the expansion of the genetic alphabet in the central dogma. P. Jpn. Acad. B-Phys. 2012; 88:345–367. 10.2183/pjab.88.345 PMC3422687 22850726

[12] Benner S.A. , Yang Z.Y., Chen F. Synthetic biology, tinkering biology, and artificial biology. What are we learning. Cr. Chim. 2011; 14:372–387. 10.1016/j.crci.2010.06.013 PMC6034680 29983697

[13] Horn T. , Chang C.A., Collins M.L. Hybridization properties of the 5-methyl-isocytidine/isoguanosine base pair in synthetic oligodeoxynucleotides. Tetrahedron Lett. 1995; 36:2033–2036.

[14] Liu Q. , Liu G.C., Wang T., Fu J., Li R.J., Song L.L., Wang Z.G., Ding B.Q., Chen F. Enhanced stability of DNA nanostructures by incorporation of unnatural base pairs. Chem. Phys. Chem. 2017; 18:2977–2980. 28856771 10.1002/cphc.201700809

[15] Grollman A.P. , Moriya M. Mutagenesis by 8-oxoguanine - an enemy within. Trends Genet. 1993; 9:246–249. 8379000 10.1016/0168-9525(93)90089-z

[16] Kamiya H. Mutagenic potentials of damaged nucleic acids produced by reactive oxygen/nitrogen species: approaches using synthetic oligonucleotides and nucleotides. Nucleic Acids Res. 2003; 31:517–531. 12527759 10.1093/nar/gkg137 PMC140503

[17] Bonicel A. , Mariaggi N., Hughes E., Teoule R. *In vitro* gamma-irradiation of DNA - identification of radio-induced chemical modifications of the adenine moiety. Radiat Res. 1980; 83:19–26. 6994167

[18] Cooke M.S. , Evans M.D., Dizdaroglu M., Lunec J. Oxidative DNA damage: mechanisms, mutation, and disease. FASEB. J. 2003; 17:1195–1214. 12832285 10.1096/fj.02-0752rev

[19] Wood M.L. , Esteve A., Morningstar M.L., Kuziemko G.M., Essigmann J.M. Genetic effects of oxidative DNA damage - comparative mutagenesis of 7,8-dihydro-8-oxoguanine and 7,8-dihydro-8-oxoadenine in *escherichia**coli*. Nucleic Acids Res. 1992; 20:6023–6032. 1461734 10.1093/nar/20.22.6023 PMC334469

[20] Batra V.K. , Shock D.D., Beard W.A., McKenna C.E., Wilson S.H. Binary complex crystal structure of DNA polymerase β reveals multiple conformations of the templating 8-oxoguanine lesion. Proc. Natl. Acad. Sci. U.S.A. 2012; 109:113–118. 22178760 10.1073/pnas.1112235108 PMC3252918

[21] Hsu G.W. , Ober M., Carell T., Beese L.S. Error-prone replication of oxidatively damaged DNA by a high-fidelity DNA polymerase. Nature. 2004; 431:217–221. 15322558 10.1038/nature02908

[22] Brieba L.G. , Eichman B.F., Kokoska R.J., Doublie S., Kunkel T.A., Ellenberger T. Structural basis for the dual coding potential of 8-oxoguanosine by a high-fidelity DNA polymerase. EMBO J. 2004; 23:3452–3461. 15297882 10.1038/sj.emboj.7600354 PMC516626

[23] Rechkoblit O. , Malinina L., Cheng Y., Geacintov N.E., Broyde S., Patel D.J. Impact of conformational heterogeneity of oxoG lesions and their pairing partners on bypass fidelity by y family polymerases. Structure. 2009; 17:725–736. 19446528 10.1016/j.str.2009.03.011 PMC4193663

[24] Beard W.A. , Batra V.K., Wilson S.H. DNA polymerase structure-based insight on the mutagenic properties of 8-oxoguanine. Mutat. Res.-Gen. Tox. Environ. 2010; 703:18–23. 10.1016/j.mrgentox.2010.07.013 PMC3023916 20696268

[25] Wood M.L. , Dizdaroglu M., Gajewski E., Essigmann J.M. Mechanistic studies of ionizing-radiation and oxidative mutagenesis - genetic effects of a single 8-hydroxyguanine (7-hydro-8-oxoguanine) residue inserted at a unique site in a viral genome. Biochemistry. 1990; 29:7024–7032. 2223758 10.1021/bi00482a011

[26] Koag M.C. , Jung H., Lee S. Mutagenic replication of the major oxidative adenine lesion 7,8-dihydro-8-oxoadenine by human DNA polymerases. J. Am. Chem. Soc. 2019; 141:4584–4596. 30817143 10.1021/jacs.8b08551 PMC6657779

[27] Leonard G.A. , Guy A., Brown T., Teoule R., Hunter W.N. Conformation of guanine:8-oxoadenine base pairs in the crystal structure of d(Cgcgaatt(O8a)Gcg). Biochemistry. 1992; 31:8415–8420. 1390625 10.1021/bi00151a004

[28] Koag M.C. , Jung H., Lee S. Mutagenesis mechanism of the major oxidative adenine lesion 7,8-dihydro-8-oxoadenine. Nucleic Acids Res. 2020; 48:5119–5134. 32282906 10.1093/nar/gkaa193 PMC7229865

[29] Delaney J.C. , Smeester L., Wong C.Y., Frick L.E., Taghizadeh K., Wishnok J.S., Drennan C.L., Samson L.D., Essigmann J.M. AlkB reverses etheno DNA lesions caused by lipid oxidation in vitro and in vivo. Nat. Struct. Mol. Biol. 2005; 12:855–860. 16200073 10.1038/nsmb996

[30] Guengerich F.P. , Crawford W.M., Watanabe P.G. Activation of vinyl-chloride to covalently bound metabolites - roles of 2-chloroethylene oxide and 2-chloroacetaldehyde. Biochemistry. 1979; 18:5177–5182. 497175 10.1021/bi00590a023

[31] Elghissassi F. , Barbin A., Nair J., Bartsch H. Formation of 1,N-6-ethenoadenine and 3,N-4-ethenocytosine by lipid-peroxidation products and nucleic-acid bases. Chem. Res. Toxicol. 1995; 8:278–283. 7766812 10.1021/tx00044a013

[32] Santos C.D. , Kouchakdjian M., Yarema K., Basu A., Essigmann J., Patel D.J. NMR studies of the exocyclic 1,N6-ethenodeoxyadenosine adduct (epsilon dA) opposite deoxyguanosine in a DNA duplex. ϵdA(syn)-dG(anti) pairing at the lesion site. Biochemistry. 1991; 30:1828–1835. 1993197 10.1021/bi00221a015

[33] Pandya G.A. , Moriya M. 1,N-6-ethenodeoxyadenosine, a DNA adduct highly mutagenic in mammalian cells. Biochemistry. 1996; 35:11487–11492. 8784204 10.1021/bi960170h

[34] Levine R.L. , Yang I.Y., Hossain M., Pandya G.A., Grollman A.P., Moriya M. Mutagenesis induced by a single 1,N-6-ethenodeoxyadenosine adduct in human cells. Cancer Res. 2000; 60:4098–4104. 10945616

[35] Tolentino J.H. , Burke T.J., Mukhopadhyay S., McGregor W.G., Basu A.K. Inhibition of DNA replication fork progression and mutagenic potential of 1,N-6-ethenoadenine and 8-oxoguanine in human cell extracts. Nucleic Acids Res. 2008; 36:1300–1308. 18184697 10.1093/nar/gkm1157 PMC2275085

[36] Moran S. , Ren R.X.F., Rumney S., Kool E.T. Difluorotoluene, a nonpolar isostere for thymine, codes specifically and efficiently for adenine in DNA replication. J. Am. Chem. Soc. 1997; 119:2056–2057. 20737028 10.1021/ja963718g PMC2925312

[37] Moran S. , Ren R.X.F., Kool E.T. A thymidine triphosphate shape analog lacking Watson-Crick pairing ability is replicated with high sequence selectivity. Proc. Natl. Acad. Sci. U.S.A. 1997; 94:10506–10511. 9380669 10.1073/pnas.94.20.10506 PMC23390

[38] Malyshev D.A. , Romesberg F.E. The expanded genetic alphabet. Angew. Chem. Int. Ed. 2015; 54:11930–11944. 10.1002/anie.201502890 PMC4798003 26304162

[39] Steinmetzger C. , Bauerlein C., Hobartner C. Supramolecular fluorescence resonance energy transfer in nucleobase-modified fluorogenic RNA aptamers. Angew. Chem. Int. Edit. 2020; 59:6760–6764. 10.1002/anie.201916707 PMC7187157 32052536

[40] Delaney J.C. , Essigmann J.M. Assays for determining lesion bypass efficiency and mutagenicity of site-specific DNA lesions in vivo. Method Enzymol. 2006; 408:1–15. 10.1016/S0076-6879(06)08001-3 16793359

[41] Chang S.C. , Fedeles B.I., Wu J., Delaney J.C., Li D.Y., Zhao L.L., Christov P.P., Yau E., Singh V., Jost M. et al. Next-generation sequencing reveals the biological significance of the N-2,3-ethenoguanine lesion in vivo. Nucleic Acids Res. 2015; 43:5489–5500. 25837992 10.1093/nar/gkv243 PMC4477646

[42] Chatgilialoglu C. , Navacchia M.L., Postigo A. A facile one-pot synthesis of 8-oxo-7,8-dihydro-(2′-deoxy)adenosine in water. Tetrahedron Lett. 2006; 47:711–714.

[43] Kouchakdjian M. , Eisenberg M., Yarema K., Basu A., Essigmann J., Patel D.J. NMR studies of the exocyclic 1,N(6)-ethenodeoxyadenosine adduct (ϵ-dA) opposite thymidine in a DNA duplex - nonplanar alignment of ϵ-dA(anti) and dT(anti) at the lesion site. Biochemistry. 1991; 30:1820–1828. 1993196 10.1021/bi00221a014

[44] Chen J.J. , Tsai C.H., Cai X., Horhota A.T., McLaughlin L.W., Szostak J.W. Enzymatic primer-extension with glycerol-nucleoside triphosphates on DNA templates. PLoS One. 2009; 4:e4949. 19305495 10.1371/journal.pone.0004949 PMC2654545

[45] Fedeles B.I. , Singh V., Delaney J.C., Li D.Y., Essigmann J.M. The AlkB family of Fe(II)/alpha-ketoglutarate-dependent dioxygenases: repairing nucleic acid alkylation damage and beyond. J. Biol. Chem. 2015; 290:20734–20742. 26152727 10.1074/jbc.R115.656462 PMC4543635

[46] Talhaoui I. , Couve S., Ishchenko A.A., Kunz C., Schar P., Saparbaev M. 7,8-dihydro-8-oxoadenine, a highly mutagenic adduct, is repaired by *escherichia**coli* and human mismatch-specific uracil/thymine-DNA glycosylases. Nucleic Acids Res. 2013; 41:912–923. 23209024 10.1093/nar/gks1149 PMC3553953

[47] Girard P.M. , D’Ham C., Cadet J., Boiteux S. Opposite base-dependent excision of 7,8-dihydro-8-oxoadenine by the ogg1 protein of *Saccharomyces**cerevisiae*. Carcinogenesis. 1998; 19:1299–1305. 9683192 10.1093/carcin/19.7.1299

[48] Tchou J. , Kasai H., Shibutani S., Chung M.H., Laval J., Grollman A.P., Nishimura S. 8-Oxoguanine (8-hydroxyguanine) DNA glycosylase and its substrate-specificity. Proc. Natl. Acad. Sci. U.S.A. 1991; 88:4690–4694. 2052552 10.1073/pnas.88.11.4690 PMC51731

[59] Michaels M.L. , Cruz C., Grollman A.P., Miller J.H. Evidence that MutY and MutM combine to prevent mutations by an oxidatively damaged form of guanine in DNA. Proc. Natl. Acad. Sci. U.S.A. 1992; 89:7022–7025. 1495996 10.1073/pnas.89.15.7022 PMC49637

[50] David S.S. , Wiliams S.D. Chemistry of glycosylases and endonucleases involved in base-excision repair. Chem. Rev. 1998; 98:1221–1261. 11848931 10.1021/cr980321h

[51] Hazra T.K. , Hill J.W., Izumi T., Mitra S. Multiple DNA glycosylases for repair of 8-oxoguanine and their potential *in vivo* functions. Prog. Nucleic Acid Res. 2001; 68:193–205. 10.1016/s0079-6603(01)68100-5 11554297

[52] Delaney J.C. , Essigmann J.M. Effect of sequence context on O-6-methylguanine repair and replication in vivo. Biochemistry. 2001; 40:14968–14975. 11732917 10.1021/bi015578f

[53] Fedeles B.I. , Essigmann J.M. Impact of DNA lesion repair, replication and formation on the mutational spectra of environmental carcinogens: aflatoxin B-1 as a case study. DNA Repair. 2018; 71:12–22. 30309820 10.1016/j.dnarep.2018.08.008 PMC6340726

[54] Schonrath I. , Tsvetkov V.B., Barcelo-Oliver M., Hebenbrock M., Zatsepin T.S., Aralov A.V., Muller J. Silver(I)-mediated base pairing in DNA involving the artificial nucleobase 7,8-dihydro-8-oxo-1,N-6-ethenoadenine. J. Inorg. Biochem. 2021; 219:111369. 33878529 10.1016/j.jinorgbio.2021.111369

[55] Krishnamurthy R. Role of pK(a) of nucleobases in the origins of chemical evolution. Acc. Chem. Res. 2012; 45:2035–2044. 22533519 10.1021/ar200262x PMC3525050

[56] Nikolova E.N. , Kim E., Wise A.A., O’Brien P.J., Andricioaei I., Al-Hashimi H.M. Transient hoogsteen base pairs in canonical duplex DNA. Nature. 2011; 470:498–502. 21270796 10.1038/nature09775 PMC3074620

[57] Alvey H.S. , Gottardo F.L., Nikolova E.N., Al-Hashimi H.M. Widespread transient hoogsteen base pairs in canonical duplex DNA with variable energetics. Nat. Commun. 2014; 5:4786. 25185517 10.1038/ncomms5786 PMC4537320

[58] Basu A.K. , Wood M.L., Niedernhofer L.J., Ramos L.A., Essigmann J.M. Mutagenic and genotoxic effects of vinyl chloride-induced DNA lesions - 1,N(6)-ethenoadenine, 3,N(4)-ethenocytosine, and 4-amino-5-(Imidazol-2-yl)imidazole. Biochemistry. 1993; 32:12793–12801. 8251500 10.1021/bi00210a031

[59] Yi C.Q. , Jia G.F., Hou G.H., Dai Q., Zhang W., Zheng G.Q., Jian X., Yang C.G., Cui Q.A., He C.A. Iron-catalysed oxidation intermediates captured in a DNA repair dioxygenase. Nature. 2010; 468:330–333. 21068844 10.1038/nature09497 PMC3058853

[60] Baldwin M.R. , Admiraal S.J., O’Brien P.J. Transient kinetic analysis of oxidative dealkylation by the direct reversal DNA repair enzyme alkB. J. Biol. Chem. 2020; 295:7317–7326. 32284330 10.1074/jbc.RA120.013517 PMC7247310

[61] Scharer O.D. Achieving broad substrate specificity in damage recognition by binding accessible nondamaged DNA. Mol. Cell. 2007; 28:184–186. 17964258 10.1016/j.molcel.2007.10.006

[62] Long H.A. , Miller S.F., Williams E., Lynch M. Specificity of the DNA mismatch repair system (MMR) and mutagenesis bias in bacteria. Mol. Biol. Evol. 2018; 35:2414–2421. 29939310 10.1093/molbev/msy134 PMC6188547

[63] Colussi C. , Parlanti E., Degan P., Aquilina G., Barnes D., Macpherson P., Karran P., Crescenzi M., Dogliotti E., Bignami M. The mammalian mismatch repair pathway removes DNA 8-oxodGMP incorporated from the oxidized dNTP pool. Curr. Biol. 2002; 12:912–918. 12062055 10.1016/s0960-9822(02)00863-1

[64] Fukui K. DNA mismatch repair in eukaryotes and bacteria. J. Nucleic Acids. 2010; 2010:260512. 20725617 10.4061/2010/260512 PMC2915661

[65] Marinus M.G. , Morris N.R. Biological function for 6-methyladenine residues in the DNA of *Escherichia**coli* K12. J. Mol. Biol. 1974; 85:309–322. 4600143 10.1016/0022-2836(74)90366-0

[66] Frick L.E. , Delaney J.C., Wong C., Drennan C.L., Essigmann J.M. Alleviation of 1,N-6-ethanoadenine genotoxicity by the *escherichia**coli* adaptive response protein alkB. Proc. Natl. Acad. Sci. U.S.A. 2007; 104:755–760. 17213319 10.1073/pnas.0607377104 PMC1783386

[67] Chang S.C. , Seneviratne U.I., Wu J., Tretyakova N., Essigmann J.M. 1,3-Butadiene-induced adenine DNA adducts are genotoxic but only weakly mutagenic when replicated in *escherichia**coli* of various repair and replication backgrounds. Chem. Res. Toxicol. 2017; 30:1230–1239. 28394575 10.1021/acs.chemrestox.7b00064 PMC5512570

[68] Strauss B. , Rabkin S., Sagher D., Moore P. The role of DNA-polymerase in base substitution mutagenesis on non-instructional templates. Biochimie. 1982; 64:829–838. 6215955 10.1016/s0300-9084(82)80138-7

[69] Gusbeth C. , Frey W., Volkmann H., Schwartz T., Bluhm H. Pulsed electric field treatment for bacteria reduction and its impact on hospital wastewater. Chemosphere. 2009; 75:228–233. 19168200 10.1016/j.chemosphere.2008.11.066

[70] Majumdar C. , McKibbin P.L., Krajewski A.E., Manlove A.H., Lee J.K., David S.S. Unique hydrogen bonding of adenine with the oxidatively damaged base 8-oxoguanine enables specific recognition and repair by DNA glycosylase mutY. J. Am. Chem. Soc. 2020; 142:20340–20350. 33202125 10.1021/jacs.0c06767 PMC9187209

[71] Gad H. , Koolmeister T., Jemth A.S., Eshtad S., Jacques S.A., Ström C.E., Svensson L.M., Schultz N., Lundbäck T., Einarsdottir B.O. et al. MTH1 inhibition eradicates cancer by preventing sanitation of the dNTP pool. Nature. 2014; 508:215–221. 24695224 10.1038/nature13181

[72] Berglund U.W. , Sanjiv K., Gad H., Kalderen C., Koolmeister T., Pham T., Gokturk C., Jafari R., Maddalo G., Seashore-Ludlow B. et al. Validation and development of MTH1 inhibitors for treatment of cancer. Ann. Oncol. 2016; 27:2275–2283. 27827301 10.1093/annonc/mdw429

[73] Sharbeen G. , Youkhana J., Mawson A., McCarroll J., Nunez A., Biankin A., Johns A., Goldstein D., Phillips P. MutY-homolog (MYH) inhibition reduces pancreatic cancer cell growth and increases chemosensitivity. Oncotarget. 2017; 8:9216–9229. 27999205 10.18632/oncotarget.13985 PMC5354726

[74] Bouvier-Muller A. , Duconge F. Application of aptamers for *in vivo* molecular imaging and theranostics. Adv. Drug. Deliver. Rev. 2018; 134:94–106. 10.1016/j.addr.2018.08.004 30125606

[75] Ismail S.I. , Alshaer W. Therapeutic aptamers in discovery, preclinical and clinical stages. Adv. Drug. Deliver. Rev. 2018; 134:51–64. 10.1016/j.addr.2018.08.006 30125605

[76] Hermann T. , Patel D.J. Biochemistry - adaptive recognition by nucleic acid aptamers. Science. 2000; 287:820–825. 10657289 10.1126/science.287.5454.820

[77] Davies D.R. , Gelinas A.D., Zhang C., Rohloff J.C., Carter J.D., O’Connell D., Waugh S.M., Wolk S.K., Mayfield W.S., Burgin A.B. et al. Unique motifs and hydrophobic interactions shape the binding of modified DNA ligands to protein targets. Proc. Natl. Acad. Sci. U.S.A. 2012; 109:19971–19976. 23139410 10.1073/pnas.1213933109 PMC3523867

[78] Nilsson J.R. , Baladi T., Gallud A., Baždarević D., Lemurell M., Esbjörner E.K., Wilhelmsson L.M., Dahlén A. Fluorescent base analogues in gapmers enable stealth labeling of antisense oligonucleotide therapeutics. Sci. Rep. 2021; 11:11365. 34059711 10.1038/s41598-021-90629-1 PMC8166847

