# Supplementary material for: 7,8-Dihydro-8-oxo-1,N6-ethenoadenine: an exclusively Hoogsteen-paired thymine mimic in DNA that induces A→T transversions in Escherichia coli
Source: Nucleic Acids Res. 2022 Mar 2;50(6):3056–69. doi: 10.1093/nar/gkac148 (PMC8989528; doi:10.1093/nar/gkac148)
Supplement: gkac148_Supplemental_File [file gkac148_supplemental_file.pdf]

Supplementary Information

7,8-Dihydro-8-oxo-1,*N*⁶-ethenoadenine – an exclusively Hoogsteen-paired thymine mimic in DNA that induces A→T transversions in *Escherichia coli*

Andrey V. Aralov[1,*], Nina Gubina[2,3], Cristina Cabrero[4], Vladimir B. Tsvetkov[5,6], Anton V. Turaev[5], Bogdan I. Fedeles[2], Robert G. Croy[2], Ekaterina A. Isaakova[5], Denis Melnik[7], Svetlana Dukova[7], Dmitriy Y. Ryazantsev[1], Alexei A. Khrulev[1], Anna M. Varizhuk[5,8], Carlos González[4], Timofei S. Zatsepin [7,9,*] and John M. Essigmann[2]

* To whom correspondence should be addressed. Tel: +79266062910; Email: Baruh238@mail.ru Correspondence may also be addressed to T.S. Zatsepin. Tel: +79265248570; Email: t.zatsepin@skoltech.ru

Experimental Procedures	1
1. Chemistry	1
2. Oligonucleotide synthesis	3
3. Circular dichroism and UV-melting	4
4. Molecular modeling	4
5. NMR spectroscopy	5
6. Biological experiments	6
Figures S1 to S11	10
Tables S1 to S12	21
NMR data	69
HPLC and MS data	73
M13 genome sequences	100
References	106

Experimental Procedures

1. Chemistry

General. All reagents were commercially available unless otherwise mentioned and used without further purification. All solvents were purchased from commercial sources. Column chromatography (CC) was performed on silica gel (0.040–0.063 mm, Merck, Germany). Thin layer chromatography (TLC) was performed on plates (Merck) precoated with silica gel (60 µm, F254) and visualized using UV light (254 and 365 nm). ^{1}H and ^{13}C NMR spectra were recorded 600 MHz and 151 MHz instruments (Bruker Avance III 600 spectrometer), respectively. ^{31}P and ^{19}F NMR spectra were recorded on 162 and 376 MHz instruments (Varian INOVA 400 NMR spectrometer or Bruker Avance III 600 spectrometer), respectively. Chemical shifts are reported in δ (ppm) units using residual ^{1}H signals from deuterated solvents as references. The coupling constants (J) are given in Hz. ESI HR mass spectra were acquired

on a LTQ FT Ultra (Thermo Electron Corp., Bremen, Germany) mass spectrometer in a negative ion mode. 2'-deoxy-7,8-dihydro-8-oxoadenosine were prepared according to literature (1).

2'-Deoxy-7,8-dihydro-8-oxo-1,N^6-ethenoadenosine **2**

To 2'-deoxy-7,8-dihydro-8-oxoadenosine **1** (0.54 g, 2.0 mmol) was added sodium acetate buffer (20 mL) containing NaOAc (0.52 g, 6.3 mmol) and CH_3COOH (0.78 mL, 13.7 mmol). To the stirred mixture was added 50 wt % chloroacetaldehyde solution in water (0.8 mL, 5.0 mmol) and the stirring was continued at 25°C for 24 h. Then, an additional 50 wt % chloroacetaldehyde solution in water (0.8 mL, 5.0 mmol) was added, and the mixture was stirred at 25°C for additional 24 h and concentrated under reduced pressure to dryness. The crude product was purified by column chromatography on silica gel (4-10% MeOH in CH_2Cl_2) yielding **2** as an off-white powder (0.49 g, 1.67 mmol, 84%). ^{1}H NMR (600 MHz, DMSO–*d$_6$*): δ 12.02 (br s, 1H), 9.16 (s, 1H), 8.03 (s, 1H), 7.57 (s, 1H), 6.26 (t, *J* = 7.3 Hz, 1H), 5.20 (d, J = 4.0 Hz, 1H), 4.74 (t, J = 5.7 Hz, 1H), 4.45-4.41 (m, 1H), 3.82-3.77 (m, 1H), 3.65-3.60 (m, 1H), 3.50-3.45 (m, 1H), 3.12-3.06 (m, 1H), 2.10-2.04 (m, 1H). ^{13}C NMR (151 MHz, DMSO–*d$_6$*): δ 151.9, 134.9, 134.1, 133.8, 132.7, 111.1, 107.7, 87.1, 81.4, 71.1, 62.1, 35.7. HRMS (ESI) m/z: calcd for $C_{12}H_{12}N_5O_4^-$ [M-H]$^-$: 290.0895; found 290.0894.

5'-O-(4,4'-Dimethoxytrityl)-2'-deoxy-7,8-dihydro-8-oxo-1,N^6-ethenoadenosine **3**

Compound **2** (0.45 g, 1.54 mmol) was co-evaporated with anhydrous pyridine (2 x 5 mL), dissolved in anhydrous pyridine (15 mL) and to the resulting solution 4,4'-dimethoxytrityl chloride (0.64 g, 1.9 mmol) was added. After 3 h at room temperature the organics were diluted with DCM (50 mL) and washed with 5% aqueous $NaHCO_3$ solution (50 mL) and saline (50 mL). The organic layer was dried over Na_2SO_4, concentrated and co-evaporated with toluene (3 x 25 mL). Purification was performed by silica gel column chromatography on silica gel (0-3% MeOH in CH_2Cl_2 with 0.1% TEA) yielding **3** as a yellowish foam (0.72 g, 1.22 mmol, 79%). ^{1}H NMR (600 MHz, DMSO–*d$_6$*): δ 12.01 (br s, 1H, 8.96 (s, 1H, 8.02 (s, 1H), 7.57 (s, 1H, 7.36-7.32 (m, 2H), 7.24-7.14 (m, 6H), 7.11-7.07 (m, 1H), 6.80-6.75 (m, 2H), 6.75-6.71 (m, 2H), 6.27 (t, J = 6.8 Hz, 1H), 5.24 (d, J = 4.6 Hz, 1H), 4.53-4.48 (m, 1H), 3.96-3.91 (m, 1H), 3.68 (s, 3H), 3.64 (s, 3H), 3.24-3.19 (m, 1H), 3.16-3.12 (m, 1H), 3.10-3.04 (m, 1H), 2.17-2.12 (m, 1H). ^{13}C NMR (151 MHz, DMSO–*d$_6$*): δ 157.8, 157.7, 151.9, 144.8, 135.6, 135.6, 134.9 (C6), 133.8, 133.7, 132.6, 129.5, 129.4, 127.6, 127.3, 126.2, 112.8, 112.7, 111.0, 107.8, 85.1, 85.0, 81.0, 70.9, 64.2, 54.8, 54.7, 35.9. HRMS (ESI) m/z: calcd for $C_{33}H_{30}N_5O_6^-$ [M-H]$^-$: 592.2202; found 592.2196.

7,8-Dihydro-8-oxo-1,N^6-ethenoadenine-N^9-β-[2'-deoxy-3'-O-(2-cyanoethyl)-N,N-diisopropylphosphoramidite-5'-O-(4,4'-dimethoxytrityl)-ribonucleoside] **4**

Compound **3** (0.65 g, 1.10 mmol) was co-evaporated from CH_2Cl_2 (2 x 10 mL) and dissolved in anhydrous DCM (5 mL) and diisopropylethylamine (0.77 mL, 4.40 mmol). To the

solution at 0°C under argon atmosphere was added 2-cyanoethyl diisopropylchlorophosphoramidite (0.40 g, 1.70 mmol). After 30 min the reaction was quenched by the addition of MeOH (0.5 mL) and the organics diluted by the addition of ethyl acetate (25 mL). The solution was then washed with 5% aqueous $NaHCO_3$ solution (25 mL) and brine (25 mL), dried over Na2SO4 and concentrated. The crude product was purified by column chromatography on silica gel (CH_2Cl_2:EtOAc:TEA 45:45:10) yielding **4** as a pale yellow foam (0.71 g, 0.89 mmol, 81%). ^{31}P NMR (243 MHz, DMSO–d_6): δ 147.5, 146.9. ^{1}H NMR (600 MHz, DMSO–d_6): δ 12.03 (br s, 1H), 9.00 (s, 1H), 8.05 & 8.04 (d, J = 1.3 Hz, 1H), 7.58 (d, J = 1.3 Hz, 1H), 7.36-7.30 (m, 2H), 7.24-7.13 (m, 6H), 7.12-7.08 (m, 1H), 6.79-6.75 (m, 2H), 6.74-6.70 (m, 2H), 6.31-6.25 (m, 1H), 4.91-4.85 & 4.84-4.78 (m, 1H), 4.09-4.03 (m, 1H), 3.79-3.60 (m, 8H), 3.60-3.47 (m, 2H), 3.28-3.12 (m, 3H), 2.76 & 2.66 (t, J = 5.9 Hz, 2H), 2.43-2.37 & 2.37-2.31 (m, 1H), 1.15-1.00 (m, 12H). ^{13}C NMR (151 MHz, DMSO–d_6): δ 157.9, 157.8, 157.8, 151.8, 151.8, 144.7, 144.7, 135.6, 135.5, 135.4, 134.8, 133.8, 133.8, 133.7, 132.5, 132.5, 129.5, 129.5, 129.4, 129.4, 127.6, 127.5, 127.3, 126.3, 118.7, 118.5, 112.8, 112.7, 111.1, 107.9, 107.9, 85.2, 85.2, 83.8 (d, $^{3}J_{C-P}$ = 4.4 Hz), 83.7 (d, $^{3}J_{C-P}$ = 5.8 Hz), 80.8, 80.7, 73.3 (d, $^{2}J_{C-P}$ = 17.6 Hz), 72.5 (d, $^{2}J_{C-P}$ = 16.2 Hz), 63.7, 63.4, 58.4 (d, $^{2}J_{C-P}$ = 18.0 Hz), 58.3 (d, $^{2}J_{C-P}$ = 18.0 Hz), 54.8, 54.8, 54.7, 42.4, 42.4, 35.2, 35.0, 24.2, 24.2, 24.2, 24.1, 24.1, 24.0, 24.0, 19.7 (d, $^{3}J_{C-P}$ = 7.0 Hz), 19.6 (d, $^{3}J_{C-P}$ = 7.0 Hz). HRMS (ESI) m/z: calculated for $C_{42}H_{47}N_7O_7P^-$ [M-H]$^-$: 792.3280; found 792.3278.

2. Oligonucleotide synthesis

Control oligonucleotide 16-mers (5'-GAAGACCNANGCGTCC-3'), barcodes, scaffolds and primers for PCR were obtained from Integrated DNA Technologies (IDT, Coralville, IA, USA). Oligonucleotide 16-mers (5'-GAAGACCNXNGCGTCC-3') with 8-oxo-1,N^6-ethenoadenine (X) were synthesized by using the phosphoramidite solid-phase method in an MM-12 (Bioautomation) DNA/RNA oligonucleotide synthesizer. Protected 2′-deoxyribonucleoside 3′-phosphoramidites, Unylinker-CPG (500Å) and *S*-ethylthio-1H-tetrazole were purchased from ChemGenes. Common synthesis followed by deprotection using an aqueous saturated ammonia solution at 55°C overnight. The solution was evaporated, and an aliquot was analyzed by HPLC and ESI-MS. HPLC analysis and purification of oligonucleotides were carried out using Agilent 1260 HPLC system equipped with autosampler and fraction collector on a 4.6 × 250 mm Jupiter C18 column (5 μm, Phenomenex); a buffer A: 0.05 M ammonium acetate (pH 7), 5% acetonitrile; a buffer B: 0.03 M ammonium acetate, 80% acetonitrile, pH 7; a gradient of B: 0→15% (1 CV), 15→50% (10 CV); a flow rate of 1 mL/min; temperature 45°C. ESI-MS analysis for the oligonucleotides was performed using Thermo Scientific LCQ Fleet with Dionex Ultimate 3000 HPLC system. The HPLC instrument was equipped with a 2.1×50 mm Jupiter C18 column (5 μm, Phenomenex); buffer A: 10 mM diisopropylamine, 15 mM 1,1,1,3,3,3-hexafluoroisopropanol; buffer B: 10 mM diisopropylamine, 15 mM 1,1,1,3,3,3-hexafluoroisopropanol, 80% acetonitrile. Salts were washed out with buffer A (4 CV) followed by a step of 100% buffer B (2 CV) with a flow rate of 0.3 mL/min; temperature 45°C. The mass spectral (MS) analysis of the oligonucleotides was

carried out in negative mode (capillary voltage 3500 V, dry temp 160°C), and raw spectra were deconvoluted using ProMass software (ENovatia, USA).

3. Circular dichroism and UV-melting

Duplex samples (5 µM in a buffer containing 140 mM KCl and 5 mM sodium phosphate, pH 7.2) were heated to 95°C for 5 min, cooled slowly to a room temperature and incubated at 4°C for 1 h prior to melting experiments.

The melting curves were obtained by measuring absorbance at 260 nm every 1°C upon sample heating at a rate of 0.5°C/min. The experimental data were fitted in ProData software (Applied Photophysics, UK) using the Boltzmann sigmoidal equation: $A = A_b + \left(\frac{A_t - A_b}{1 + exp\left(\frac{x_0 - x}{w}\right)}\right)$, where A refers to absorbance, and A_b and A_t are the minimum and maximum values, respectively, x is temperature, x_0 is the transition temperature, and w is a fitting parameter.

Circular dichroism (CD) spectra of samples were recorded on a Chirascan spectrophotometer (Applied Photophysics, UK) at 20°C. CD was registered between 220 and 330 nm, and molar circular dichroism $\Delta\varepsilon$ was calculated as follows: $\Delta\varepsilon = \frac{\theta}{3.3 * 10^4 * C * l}$, where θ is the ellipticity (mdeg), C is the ON concentration (M), and l is the optical path length (cm).

4. Molecular modeling

The 3D models of the X^s and X^a were built using the molecular graphics software package Sybyl-X (Certara, USA). Partial charges on the X^s and X^a atoms were calculated according to the following scheme. Calculation of electron density distribution were performed by using second-order *Møller–Plesset* perturbation theory (MP2) (2), implicit consideration of the solvent effect with application of the conductor-like polarizable continuum model (CPCM) (3) and cc-pvdz basis sets. Then the Merz-Singh-Kollman scheme (4) was applied to the obtained electron density distribution for calculation of the grid for the electrostatic potential fitting with the following parameters: (6/41=10) - the number of surfaces around the atoms and (6/42=17) - the density of test points on these surfaces. The RESP (Restrained ElectroStatic Potential) method (5) was applied to fitting of the grid obtained in the previous step for calculation of partial atomic charges. All quantum mechanics simulations were carried out using the Gaussian 09 program (6).

Molecular dynamics simulations (MD) were carried out using the Amber 18 suite of programs (7). The influence of solvent water was simulated by using OPC3 (8). The simulation was performed by using periodical boundary conditions and a rectangular box. The buffer between complex DNA and the periodic box wall was at least 15 Å. The parameters needed for calculation of interatomic energies were taken from the force fields OL15 (9, 10) for DNA and from gaff2 general Amber force field (11) for the X^s and X^a. For neutralizing of the negative charge K+ ions also were used. At the beginning of computing the investigated systems were minimized by two steps. At the first stage, location of the solvent molecules was optimized by

using 1000 steps (500 steps of steepest descent followed by 500 steps of conjugate gradient), at that mobility of all solute atoms was restrained with a force constant of 500 kcal*mol-1*Å-2. At the second stage, the optimization was accomplished without any restriction using 2500 steps (1000 steps of steepest decent, 1500 steps of conjugate gradient). Then gradual heating to 300 K during 20 ps was performed. To avoid wild fluctuations for investigated systems in this stage, weak harmonic restrains were used with a force constant of 10 kcal*mol-1*Å-2 for all atoms being that were not a part of the solvent. The SHAKE (12) algorithm was applied to constrain bonds to hydrogen atoms, that allowed to use 2 fs step. Scaling of nonbonded 1–4 Van der Waals and electrostatic interactions were performed by the standard Amber values. The cutoff distance for non-bonded interactions was equal to 10 Å and the long-range electrostatics calculated using the particle mesh Ewald method (13). The MD simulations in production phase were carried out using constant temperature (T = 300 K) and constant pressure (p = 1 atm) over 80 ns. To control the temperature, a Langevin thermostat was used with the collision frequency of 1 ps^{-1}. Energy of the DNA was estimated by using the GBSA approach. The free energy was calculated as the sum of the electrostatic energies (Eq), Van der Waals energies (E_{VDW}), and energy of solvation. The energy of solvation was calculated as the sum of the polar and nonpolar contributions. The polar contribution ($E_{GB)}$ was computed using the Generalized Born (GB) method and the algorithm developed by Onufriev et al. for calculating the effective Born radii (14). The non-polar contribution to the solvation energy (Esurf), which includes solute-solvent van der Waals interactions and the free energy of cavity formation in solvent, was estimated from a solvent-accessible surface area (SASA). Snapshot visualization was performed using VMD (15). To estimate the number of hydrogen bonds, the following parameters were used: donor-acceptor distance of 3.2 Å and angle cut-off of 20 degrees.

5. NMR spectroscopy

NMR spectroscopy. Samples of the duplex (ODN5-ODN6, see Table S1) were suspended in 500 μL of a buffer (0.1 M NaCl, 10 mM phosphate, and 1 mM EDTA, 90:10 H_2O/D_2O, pH 7.0). NMR spectra were acquired in Bruker AVANCE spectrometers operating at 600 or 800 MHz and equipped with cryoprobes. TOCSY experiments were recorded with standard MLEV-17 spin-lock sequence, and 80-ms mixing time. The NOESY spectra were acquired with mixing times of 50 and 150 ms. Water suppression was achieved by including an excitation sculpting module in the pulse sequence prior to acquisition. Experiments were recorded at several temperatures (Figure S5). Assignment lists are reported at T=3 ºC (Table S4).

*NMR constraints***.** Qualitative distance constraints were obtained from NOE cross-peaks according to their intensity. In addition to these experimentally derived constraints, Watson-Crick hydrogen bond restraints were used. Target values for distances and angles related to hydrogen bonds were set as described from crystallographic data. No backbone angle constraints were employed. Distance constraints with their corresponding error bounds were incorporated into the AMBER potential energy map by defining a flat-well potential term.

Structure determination from NMR restraints. Structures were calculated with the SANDER module of the molecular dynamics package AMBER 18.0 (7). Starting models of the duplex were built in the A- and B- canonical structures. These structures were placed in the center of a water-box with around 4000 water molecules and sodium counterions to obtain electroneutral systems. We used the parmbsc1 (16) revision of the parm99 force field. The protocol for the constrained molecular dynamics refinement consisted of a quick minimization, followed by a 100 ps thermalization at 298 K, and an equilibration period of 100 ps using a standard equilibration process (17). Then, a 1 ns trajectory was run, and averaged structures were obtained from last 500 ps by averaging snapshots during 10 ps of every 100 ps. A total of 10 structures were obtained (five structures from the run starting from the A-form, and other five structures from the B-form).
Analysis of the representative structures as well as the MD trajectories was carried out with the programs X3DNA (18), MOLMOL (19), Pymol, and several AMBER analysis tools.

6. Biological experiments

Vectors and Escherichia coli strains. DNA adduct-bearing oligonucleotides and barcodes were cloned into M13Mp7(L2) phage. M13mp7(L2) is a derivative of M13mp7 with an extended palindromic multiple cloning site (20, 21) M13mp7(L2) was a gift from C. W. Lawrence (University of Rochester). The complete sequence of WT M13Mp7(L2) is included at the end of this document to help readers find the sites to which probes anneal.

Large-scale growth of the WT phage was performed in GW5100 (JM103 (*endA1 glnV44 sbcBC rpsL thi-1 Δ(lac-proAB)* F'[*traD36 proAB+ lacIq lacZΔM15*]; streptomycin resistant) (22) plus *P1*-(23)); the strain was obtained from G. Walker, of the Massachusetts Institute of Technology (MIT).

M13 constructs were electroporated into AB1157 (wild-type, *thr-1, leu-6, thi-1, lacY1, galK2, ara-14, xyl-5, mtl-1, proA2, his-4, argE3, str-31* (same as *rpsl-31*), *tsx-33, supE44*), *mutY-* (AB1157, *mutY*::mini-*Tn10-tet*), *mutM-* (AB1157, *mutM-*), *mutY-/mutM-* (AB1157, *mutM-, mutY-*), HK81 (AB1157, *thr-1 leu-6 proA2 his-4 argE3 thi-1 lacY1 galK2 ara-14 xyl-5 mtl-1 tsx-33 strA31 supE44 nalA*) and HK82 (HK81, *alkB22* point mutation). *mutY-, mutM-* and *mutY-/mutM-* cells were obtained from J. Miller (University of California, Los Angeles). HK81 and HK82 were provided by L. Samson, MIT.

Phage progeny were plated using NR9050 (F′ *prolacIZΔM15, Δprolac, suB*) from R. M. Schaaper (National Institute of Environmental Health Sciences) as an indicator strain and regrown in SCS110 cells (*rpsL* (*Str r*) *thr leu endA thi-1 lacY galK galT ara tonA tsx dam dcm supE44* Δ*(lac-proAB) [F′ traD36 proAB lacI q Z*Δ*M15*]; streptomycin resistant) (Agilent).

Purification of the WT M13Mp7(L2) phage genome. The single stranded M13mp7(L2) genome was prepared as described in Delaney and Essigmann (24), with a few modifications. Briefly, a starter culture was made by plugging a well isolated plaque with a sterile Pasteur pipette from a lawn of *E. coli* NR9050 infected with M13mp7(L2) phage. The plug was vortexed in 1 mL of LB media and spun down at 18000g for 4 min. Two hundred μL of the

supernatant were added to 2.5 h culture of GW5100 cells (10 mL in 2xYT medium) and grown for 8 h at 37°C to achieve a typical titer of $>10^{12}$ pfu/mL. The phage supernatant was obtained by centrifugation at 7500 rpm, 4°C, for 10 min.

Then, 2x500 mL of 2xYT were inoculated by 1 mL of GW5100 O/N cells and grown with aeration in a 1L baffled flasks at 37°C for 3 h. After that, 2 mL of the starter phage supernatant were added to the cultures and grown for 8 h. Cell cultures was aliquoted to spin bottles, cells were pelleted by centrifugation at 7500 rpm, 4°C, for 10 min and discarded. Phage were precipitated for 48 h with 4% PEG8000 and 0.5 M NaCl (final concentrations), collected by centrifugation at 10000 rpm for 10 min and resuspended in total 20 mL of TE (pH 8.0). Phage DNA was extracted from 5 mL aliquots three times by 3 mL of phenol/chloroform/isoamyl alcohol (25:24:1). The aqueous phases were pooled and passed through a small hydroxyapatite column (1 g Bio-Gel HTP hydroxyapatite (Bio-Rad), 12 mm x 16 cm), washed with 10 mL TE (pH 8.0), and eluted in 15 mL of phosphate buffer (0.16 M KH_2PO_4 / 0.16 M K_2HPO_4). One mL fractions of eluate were collected into separate tubes, and ssDNA concentrations were measured in a NanoDrop™ OneC instrument (Thermo Scientific™). Fractions with highest concentrations of DNA were pooled together, diluted with TE (pH 8.0) to 12 mL, aliquoted into four portions and purified on Microsep Advance centrifugation filters (100K) (PALL) three times, by dilution in 3 mL TE (pH 8.0) and centrifugation at 4500 rpm for 12 min. Purified phage DNA solutions were pooled together, adjusted to 1 µM and kept at -80°C prior to use.

Construction of control and lesion-containing M13Mp7(L2) genomes. Barcoded lesion-containing genomes were constructed based on a previously reported method (25) with some modifications. Briefly, 30 pmol of a single 16-mer (5'-GAAGACCTXGGCGTCC-3', X is the lesion) or of a pool containing an equimolar mixture of 16 16-mers (5'-GAAGACCNXNGCGTCC-3', X is the lesion, Ns are variable nucleotides generating $2^4 = 16$ possible trinucleotide combinations) were ligated to 30 pmol of 18-mer containing a trinucleotide barcode unique to each single lesion or to a pool (5'-CACGGTBBBTGCTCTGAC-3'; BBB is a trinucleotide barcode) with T4 DNA ligase (New England Biolabs) and a scaffold sequence complementary to the 3'-end of the barcode-containing 18-mer and the 5'-end of the lesion-containing 16-mer (5'-GGTCTTCGTCAGAGCA-3'). The 34-mer ligation product was then ligated to 20 pmol of EcoRI (New England Biolabs) linearized M13mp7(L2) single-stranded bacteriophage genomic DNA with additional T4 DNA ligase and two genome construction scaffolds: 5'-ACCGTGCACTGAATCATGGTCATAGC-3' and 5'-AAAACGACGGCCAGTGAATTGGACGC-3', 25 pmol each. After the removal of scaffolds by the 3'-exonuclease activity of T4 DNA polymerase (New England Biolabs), the ligated M13 genome was extracted with phenol/chloroform/isoamyl alcohol (25:24:1) (Invitrogen) and purified with QIAprep Spin Miniprep Kit (Qiagen) following the manufacturer's protocol.

Replication of the phage genome construct in E. coli. Electrocompetent cells were prepared as described in Delaney and Essigmann (24). Briefly, 2 mL of the overnight bacterial culture were added to 200 mL of Luria broth in a 0.5 L flask and grown with aeration for a few hours until the optical density of the solution reached 0.5 at 600 nm. Cells were spun down by centrifugation at 6000 rpm for 10 min, washed three times with ice-cold sterile water, resuspended in 3 mL of an ice-cold sterile glycerol and kept in small aliquots at -80°C prior to use.

One hundred fmol of a single genome or 480 fmol of a pool (NAN or NXN) were electroporated into 100 µL of *E. coli* competent cells with a peak discharge 2.5 kV for > 4.6 ms on average, in a 2-mm electroporation cuvette (VWR). Each electroporation was done in triplicate. After electroporation, cells were immediately transferred into 1 mL of LB medium. One hundred µL aliquots were used to check the efficiency of each electroporation as described previously (20, 24), the remainder of the mixture was transferred to 10 mL LB medium and grown for 6 h at 37°C. All electroporations resulted in at least 5.3×10^4 initial events (infective centers), with most of the samples producing $>10^5$ events. After the 6 h incubation, the progeny phage were amplified in SCS110 cells at 37°C for 7 h to dilute out the residual genomic DNA used for electroporation. Cells were pelleted, and the supernatant containing phage progeny was isolated and stored at 4°C. Single-stranded DNA from the progeny phage was isolated with the QIAprep Spin Miniprep Kit (Qiagen) following the manufacturer's instructions.

An ~1 kb sequence covering the region of interest was amplified in triplicate by PCR with 250 pg of the M13 template, 600 nmol of each PCR primer (forward: 5'-CGATTTCGGAACCACCATCAAACAGG-3', reverse: 5'-TGAGAGTCTGGAGCAAACAAGAGAATCG-3') and Phusion® High-Fidelity DNA Polymerase (New England Biolabs). The PCR program included initial denaturation at 98°C for 2 min, 25 cycles of 98°C for 10 s, 71°C for 20 s and 72°C for 30 s, and final extension at 72°C for 2 min. The PCR product was purified with the QIAquick PCR Purification Kit (Qiagen) and stored at −20°C.

*Library preparation and next-generation sequencing***.** Triplicates of purified 1 kb PCR amplicons were pooled, diluted to 2 ng/µL, and, for each resultant biological sample, three technical replicates of 20 µL were submitted for next generation sequencing at the MIT BioMicro Center (URL: https://openwetware.org/wiki/BioMicroCenter). Libraries were prepared using a low volume pipetting robot mosquito® HV genomics instrument (SPT Labtech, Melbourn, United Kingdom) and Nextera DNA Flex Library Prep (now called Illumina DNA Prep, Illumina) instrument following the instructions of the manufacturer. Each sample was barcoded by two specific barcodes from IDT for Illumina Nextera DNA Unique Dual Indexes Sets A and B (now called IDT for Illumina DNA/RNA UD Indexes Sets A and B). Indexed samples were pooled and submitted for sequencing on a MiSeq instrument (Illumina) in one flowcell with 300 + 300 paired-end, v3 chemistry.

Next-generation sequencing data analysis. The quality of reads was evaluated by FASTQC software (26). The total number of read pairs for the experiment with two genomes and MutY/MutM deficient cells was 1,491,652 accounting in average for 20,717 read pairs per each genome. The total number of read pairs for two pools of 16 genomes and AlkB deficient cells was 6,766,147, giving in average 11,876 read pairs per each genome. The Nextera Flex adapters were removed by Trimmomatic v. 0.39 (27). Paired-end reads were concatenated using PEAR v0.9.10 (28) with 10 bases as a minimal length of read and a base PHRED quality score threshold 33. Then, a self-written script was used to extract the reads having a 34-mer insert (18-mer barcode and 16-mer lesion oligonucleotide) and to split them into groups associated with lesions – and within each lesion-related group – into subgroups associated with 16 trinucleotide contexts, where applicable. As an anchor to pick the reads, we used the 5'-ACGGT$N_1N_2N_3$TGCTCTGACGAAGACC$N_4N_5N_6$GCGTC-3' sequence, which is a 32-base part of the 34-mer insert where $N_1N_2N_3$ corresponds to barcode trinucleotide, and $N_4N_5N_6$ to the lesion trinucleotide. The total number of the fragments after all steps of data trimming and filtration was 80,895 for the experiment with MutM/MutY deficient cells and 387,565 for the experiment with the AlkB deficient strain. On average, 5.5% of raw fragments were selected to estimate the mutation frequency at the lesion site.

The selected fragments were mapped to the M13mp7(L2) genome containing TAG 18-mer and TAG 16-mer insertions using Bowtie 2 v.2.2.6 (29). The full sequence of this genome is provided at the end of this document. The efficiency of mapping was 98.8% for the experiment with MutM/MutY deficient strains and 98.4% for the experiment with AlkB deficient strain. Median coverage at the lesion site was 1109 fragments per genome for the experiment with MutM/MutY deficient strains and 584 fragments per genome for the experiment with the AlkB deficient strain, which is sufficient to infer statistically significant information about mutation frequency.

Mapped reads were piled up using SAMtools v.0.1.19 (30) with the options "mpileup−AB −d1000000". Finally, the mutation rate was calculated for each base inside the region of interest (6244..6280 bases) using a custom Python script. The miscoding percentage was obtained by calculating the relative proportion of each native base (A, C, G, or T) at the site previously occupied by the lesion (Tables S7 and S9 for MutM/MutY deficient strains, Table S10 for AlkB deficient strain). Statistical comparison between genotypes and between sequence contexts was performed using ANOVA with the Tukey post-hoc test (Tables S8 and S10 for the MutM/MutY deficient strains, Table S11 for the AlkB deficient strain).

Statistical treatment (ANOVA with Tukey's post-hoc test) was performed using R (R Core Team (2020)). R: A language and environment for statistical computing. R Foundation for Statistical Computing, Vienna, Austria. URL https://www.R-project.org/); $p<0.05$ was taken as a significance threshold. Data were visualized using R package ggplot2 (31).

Figures S1 to S11

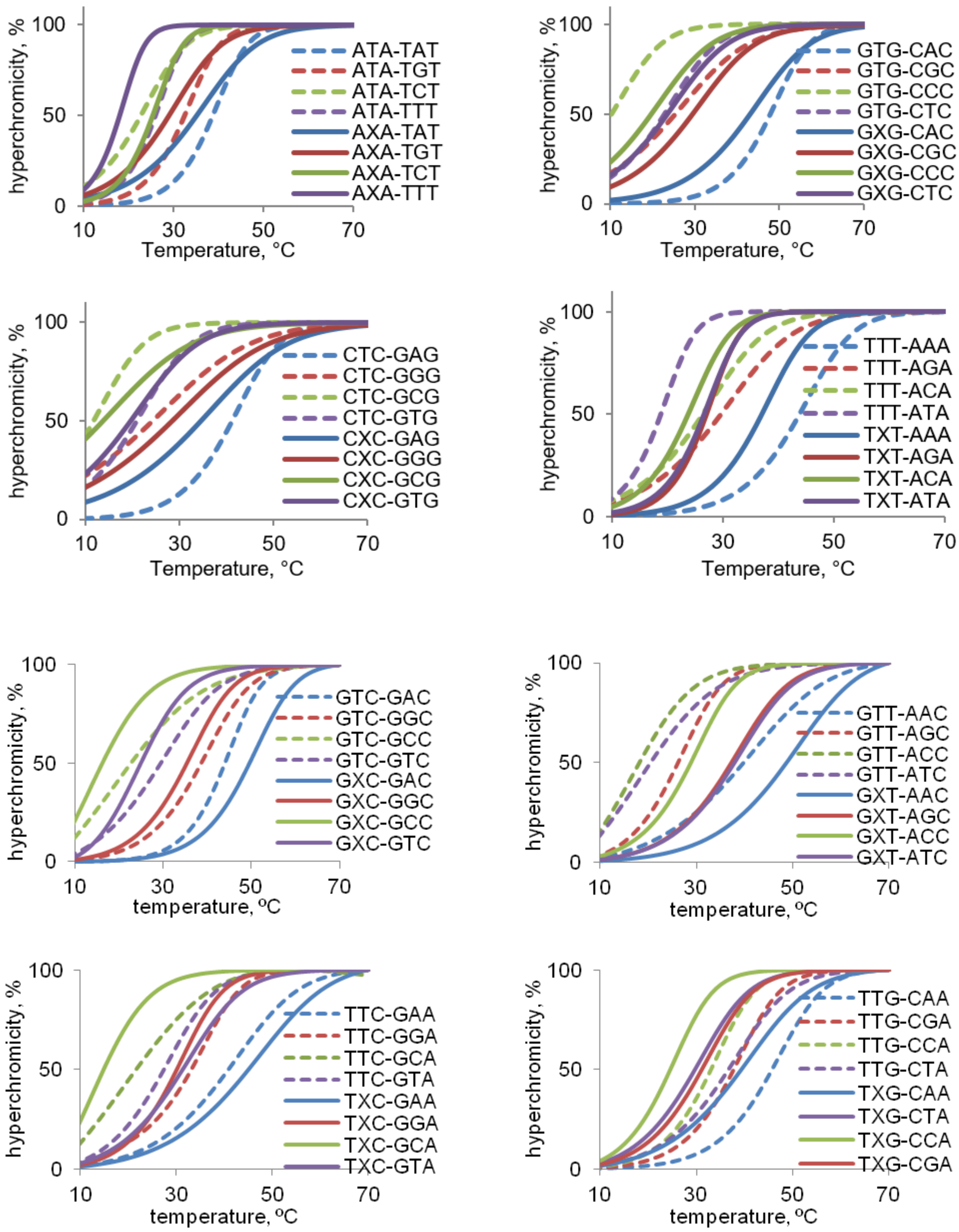

Fig. S1. Melting curves of the duplexes formed by a native (5'-GCANTNTACG-3') or modified (5'-GCANXNTACG-3', X = oxo-εA) strand and a complement (5'-CGTANNNTGC-3').

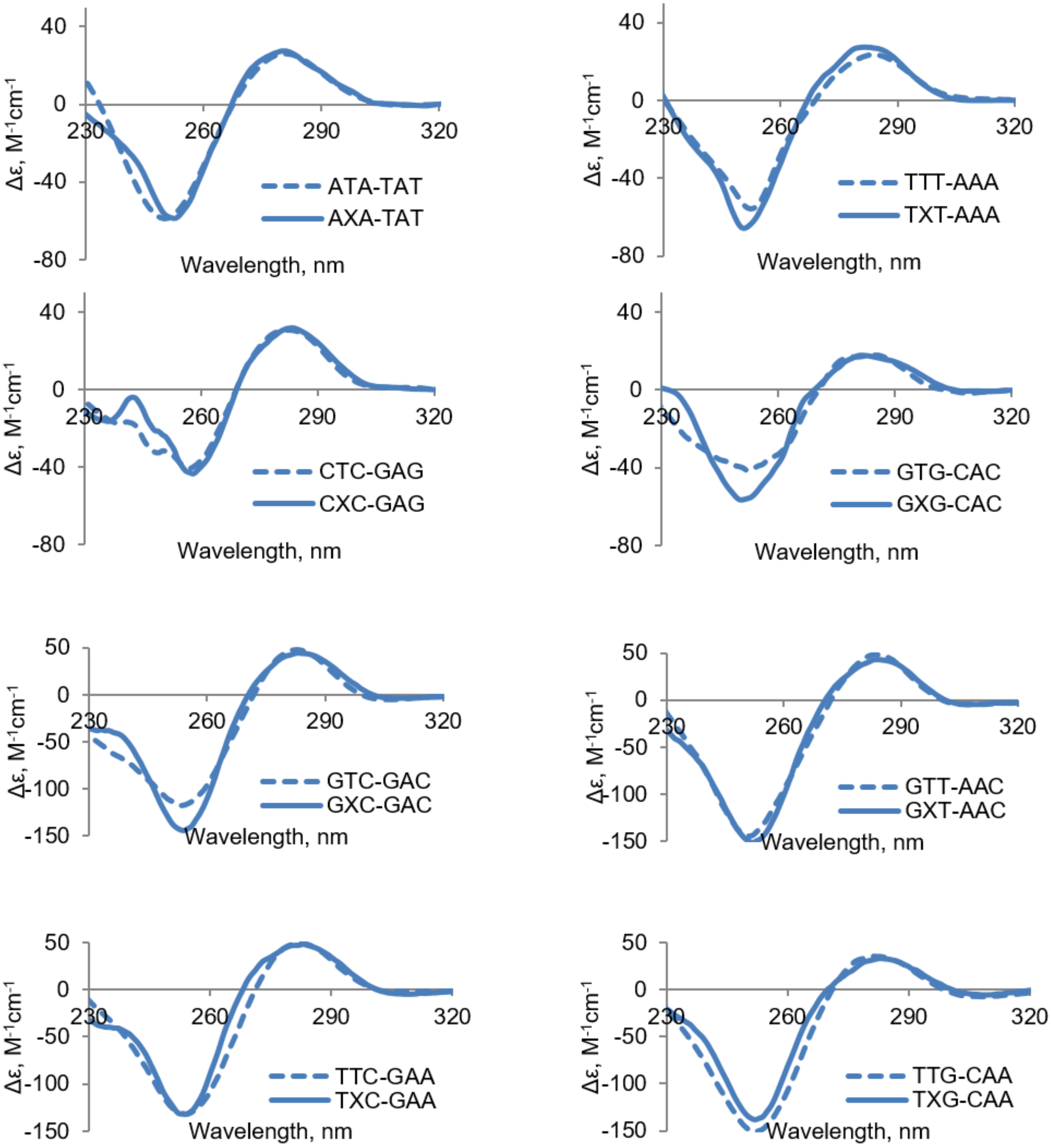

Fig. S2. CD spectra of the duplexes formed by native (5'-GCANTNTACG-3') or modified (5'-GCANXNTACG-3', X = oxo-εA) strand and complement (5'-CGTANANTGC-3').

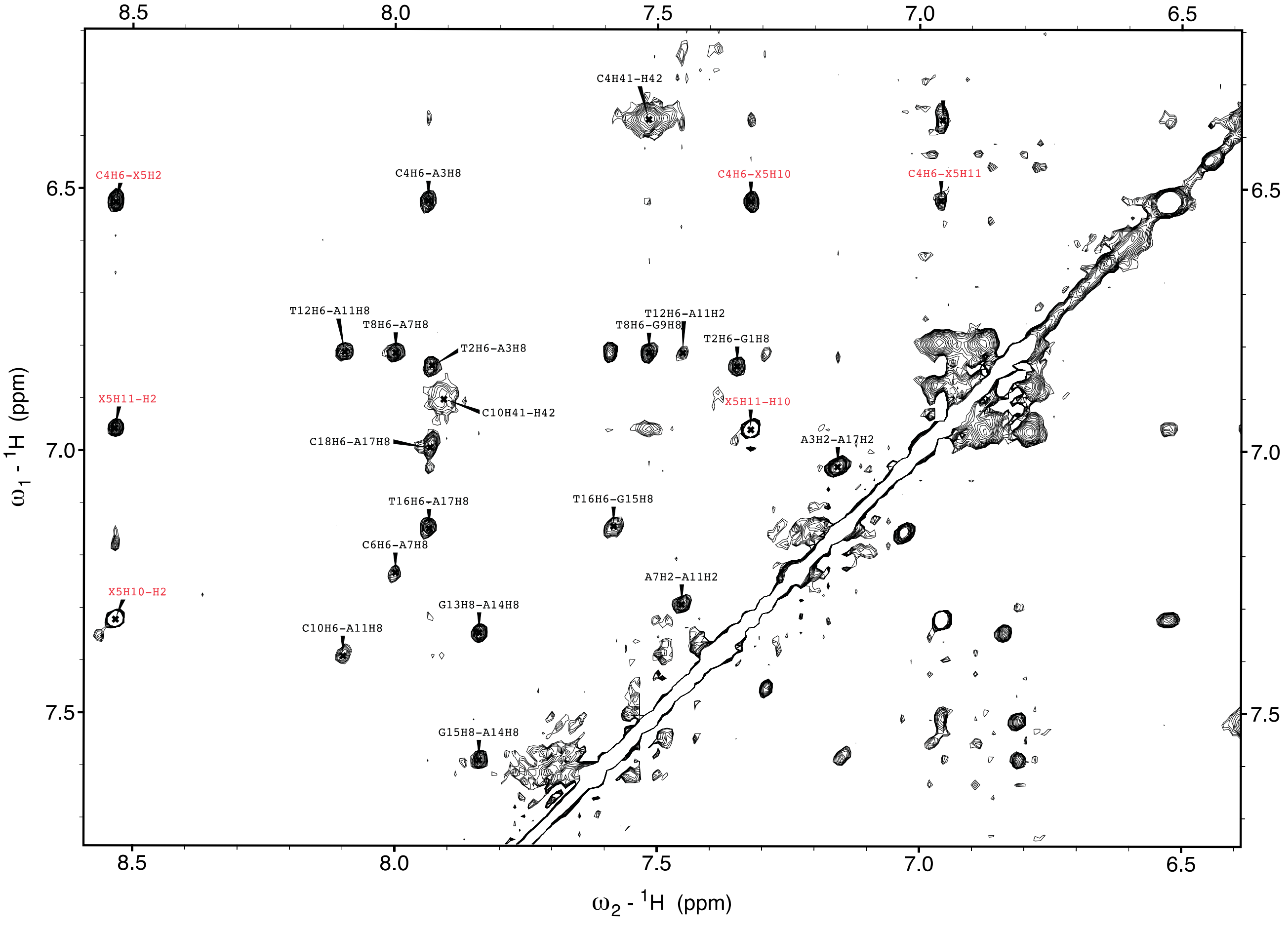

Fig. S3. Aromatic-aromatic region of the NOESY spectra (mixing time = 150ms, T= 5ºC). X5 resonances are labeled in red.

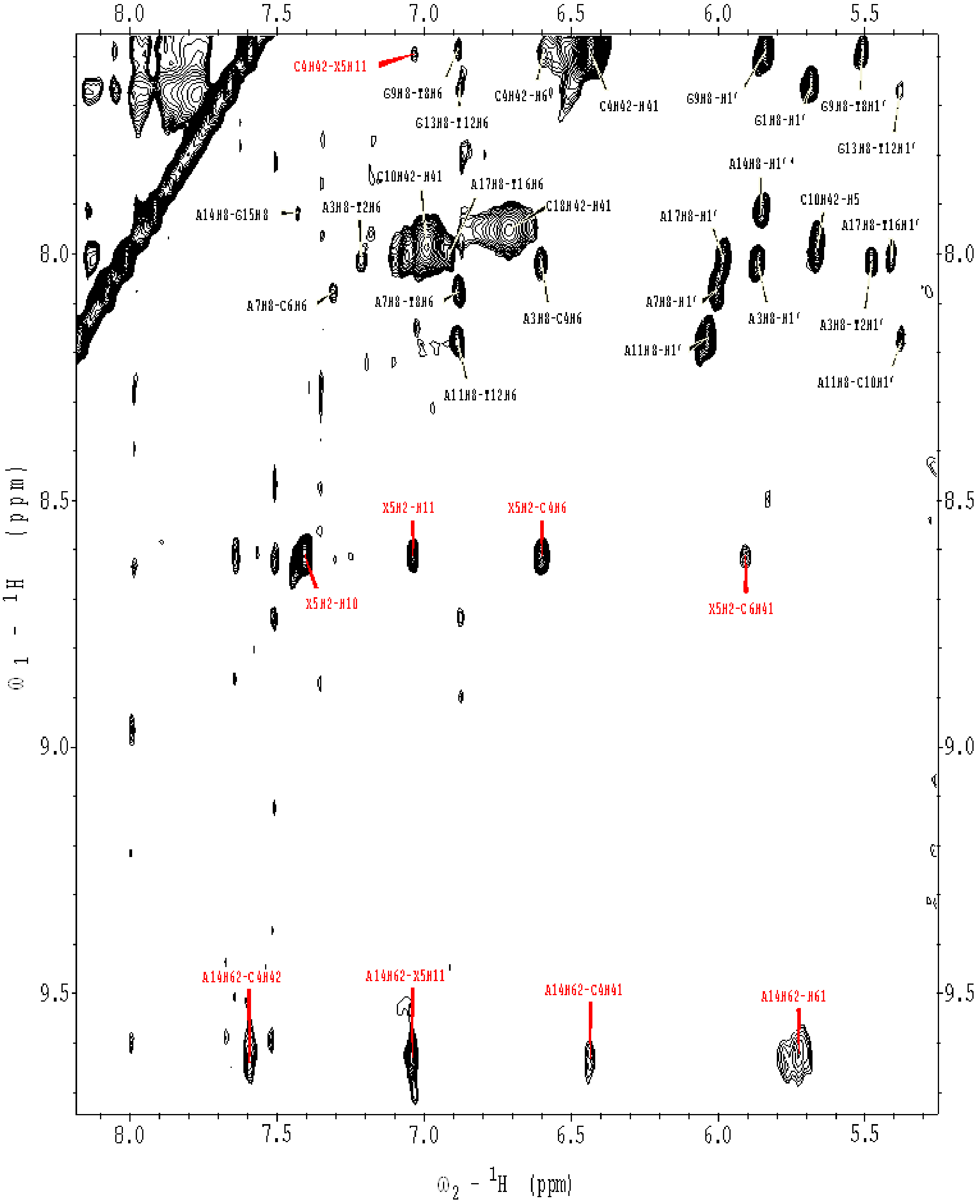

Fig. S4. Exchangeable proton region of the NOESY spectra (mixing time = 75 ms, T= 2ºC). Cross-peaks involving X5 and A14 resonances are labelled in red.

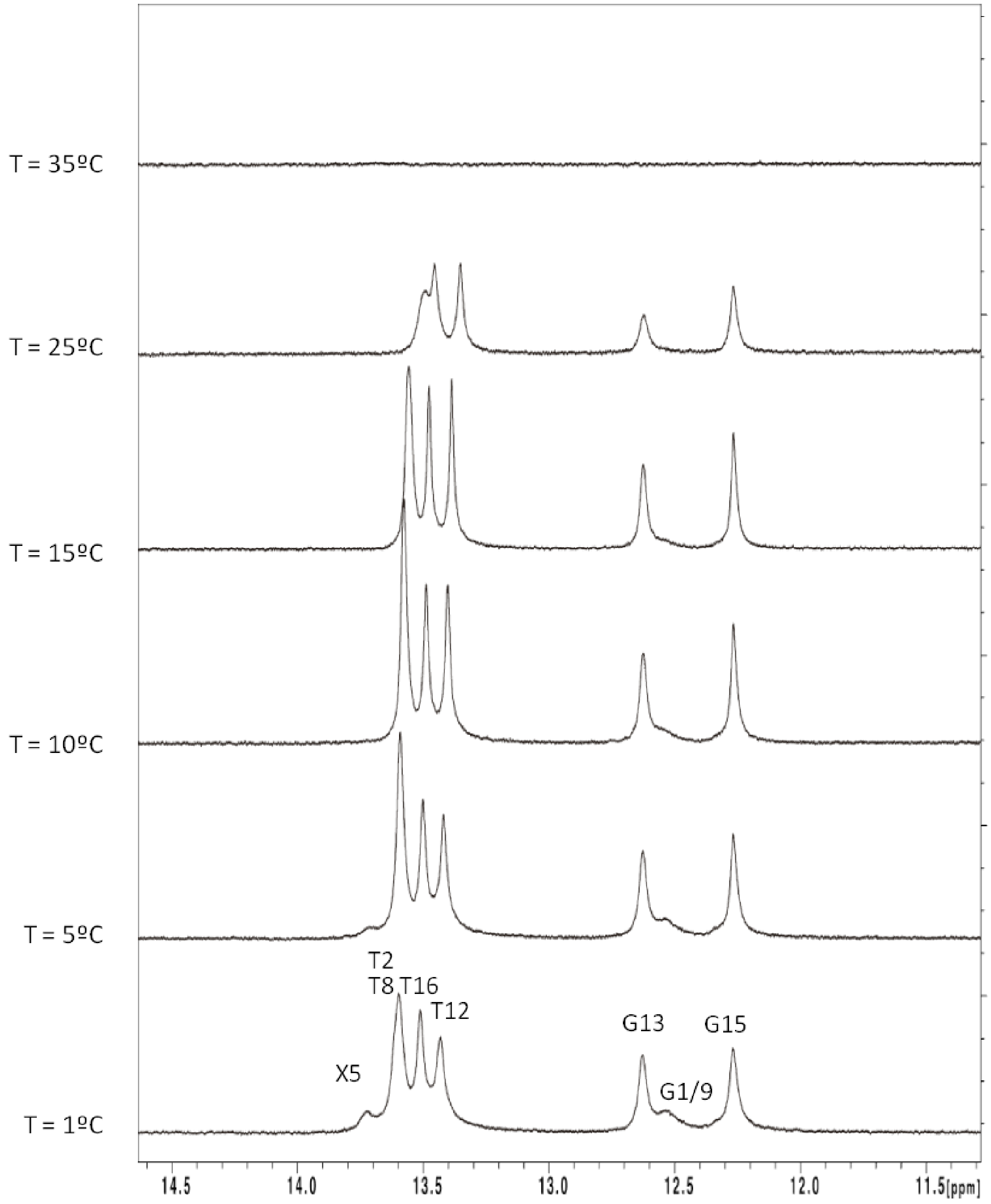

Fig. S5. ^{1}H-NMR melting experiment. Exchangeable protons region of the NMR spectra at different temperatures.

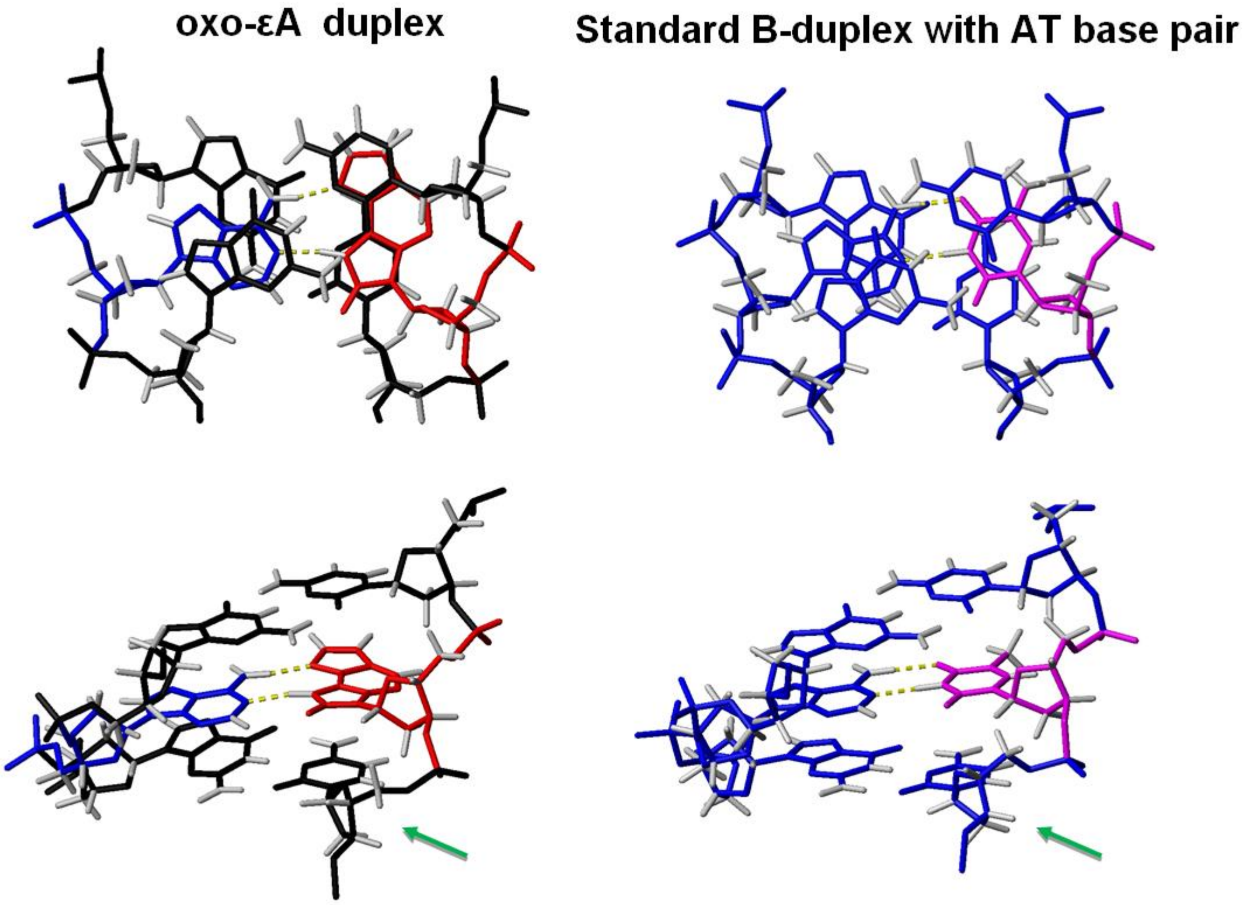

Fig. S6. Two views of the oxo-εA:A base pair and the surrounding nucleotides in the solution. Structure determined by NMR methods (left), and a comparison with a standard B-form duplex with an A:T base pair in the same position (right). Minor changes in the backbone geometry are indicated with green arrows. Oxo-εA is shown in red and thymine in magenta.

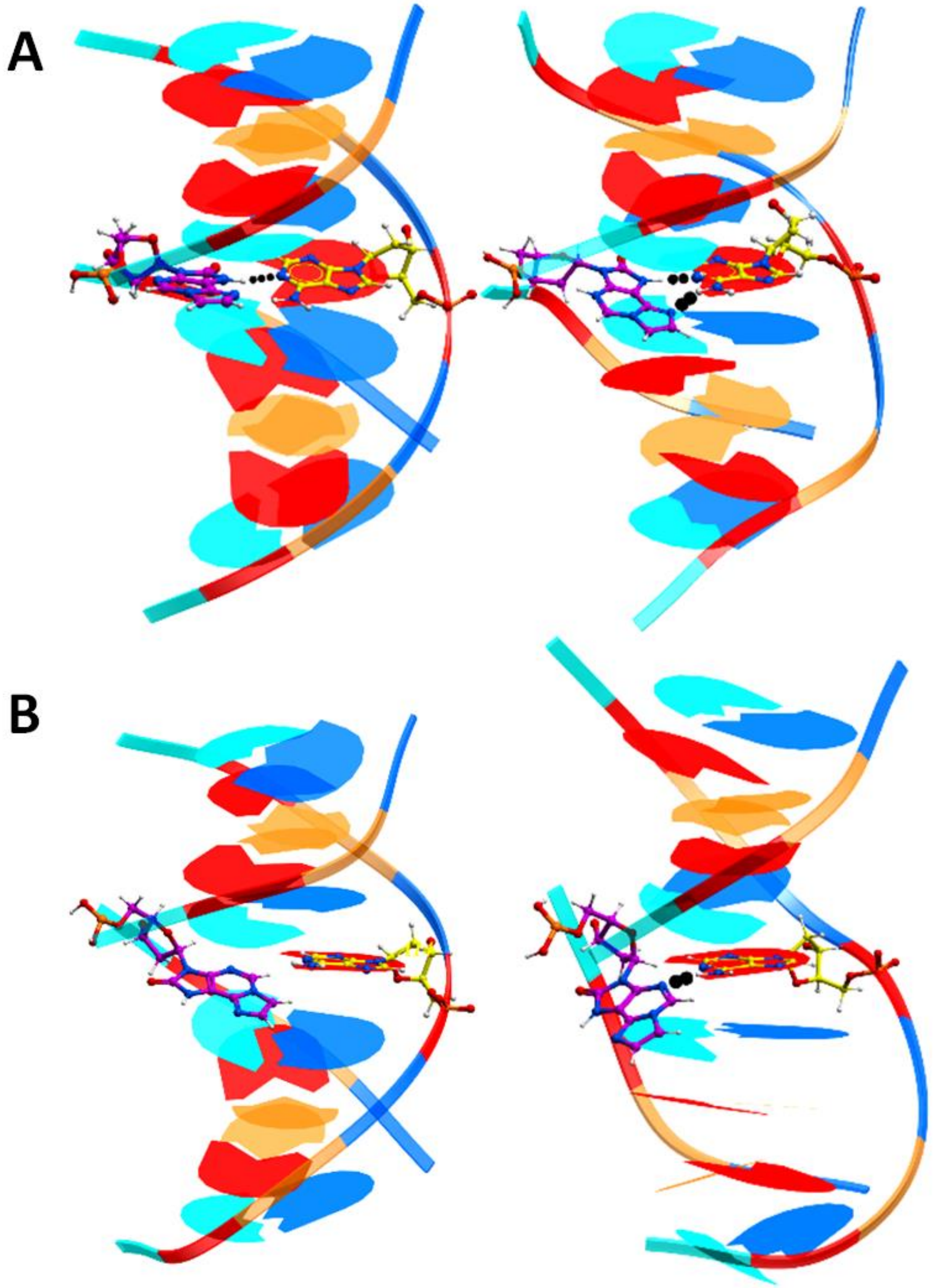

Fig. S7. MD simulations of the duplex containing central **X**-A pair at start (left) and after 70 ns (right): $\mathbf{X^{S}}$ (**A**) and $\mathbf{X^{A}}$ (**B**).

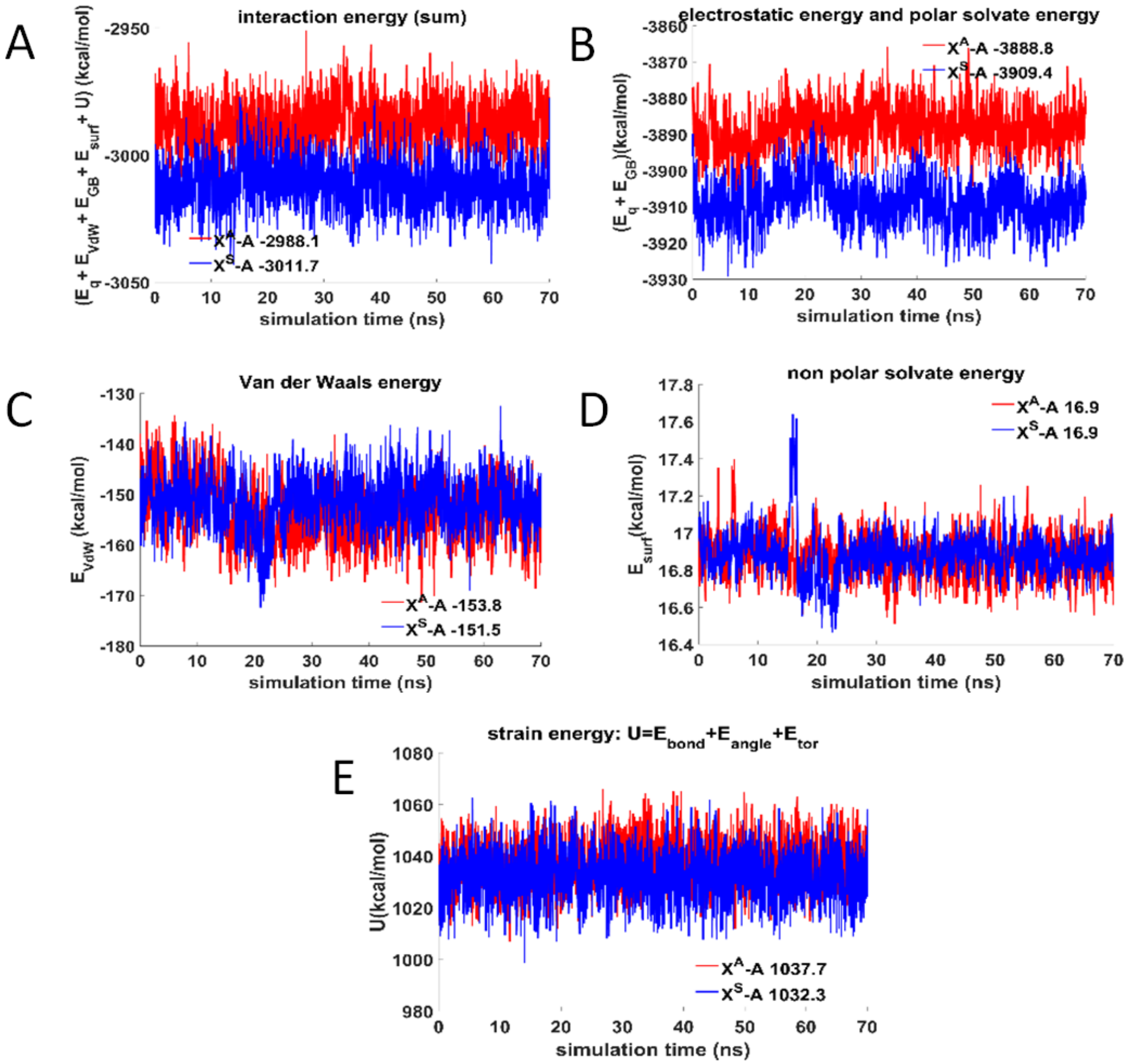

Fig. S8. Comparison of contributions in free energies of X^S–A and X^A-A: sum of internal energy and energy of solvation (**A**); E_{eq} + E_{GB} – electrostatic energy of the molecule in the gas phase and the polar component of solvation energy computed using the Generalized Born (GB) method (**B**); E_{VdW} – Van der Waals energy (**C**); E_{surf} – non-polar component of solvation energy (**D**); U = E_{bond}+ E_{angle}+ E_{tor} , e.g. E_{bond}, E_{angle} and E_{tor} –bond, angle and torsion stress energies (**E**). The energy plots were smoothed using the moving average method (span = 5). Average energy values are indicated in the Figure legends.

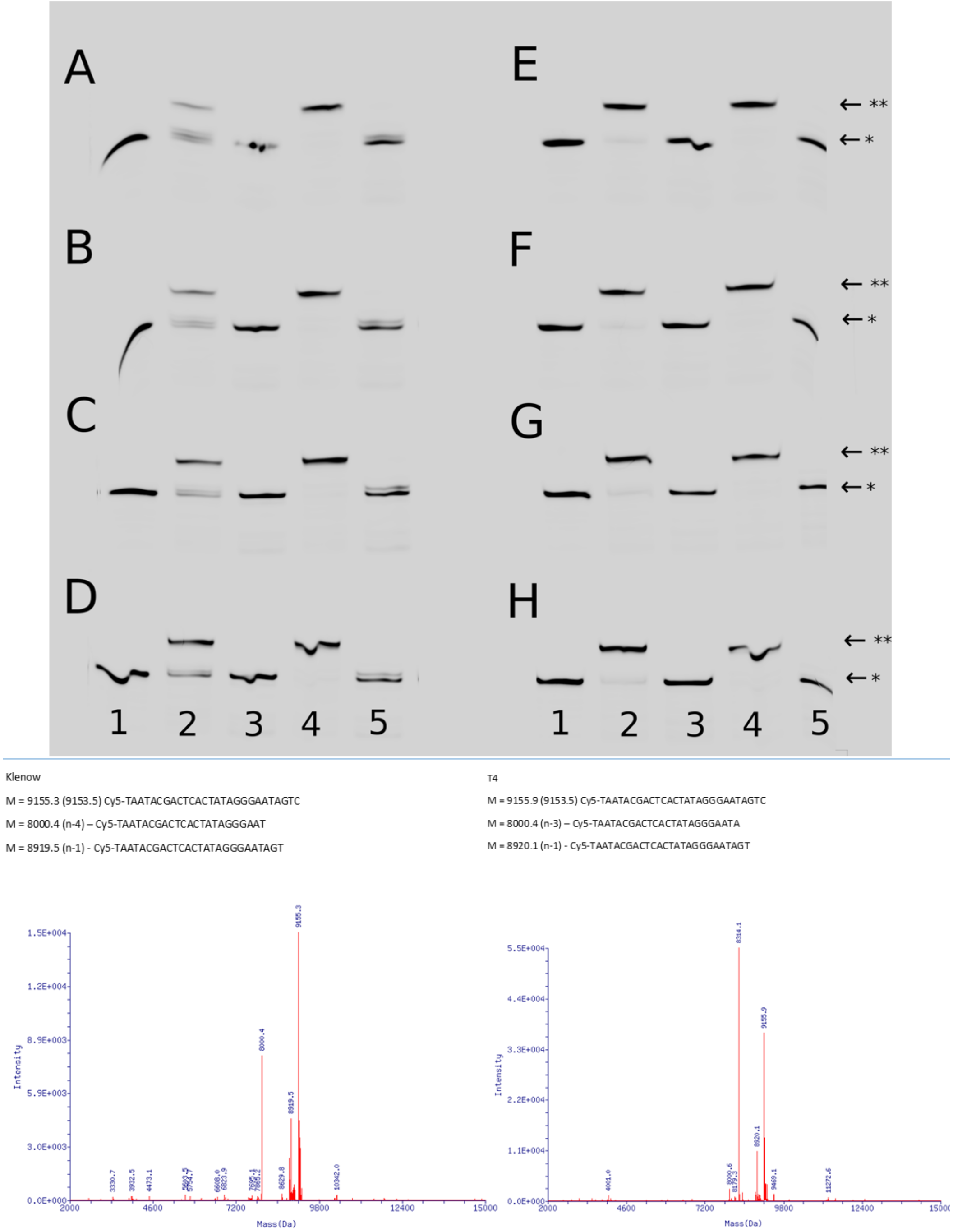

Fig. S9. Primer extension assay using the native (**ODN11**, T at 7-position) or modified (**ODN12**, oxo-εA at 7-position) template: after incubation with Klenow fragment for 1 (A), 2 (B), 3 (C) or 4 (D) h, or with T4 DNA polymerase for 1 (E), 2 (F), 3 (G) or 4 (H) h; lane 1 – **ODN12**+1 μL 0.5M EDTA; lane 2 - **ODN12**+dNTPs, lane 3 - **ODN12**+dNTPs without dATP, lane 4 - **ODN11**+dNTPs, lane 5 - **ODN11**+dNTPs without dATP (upper panel, * –primer, ** - primer extension product). Elongation product analysis by MS (lower panel).

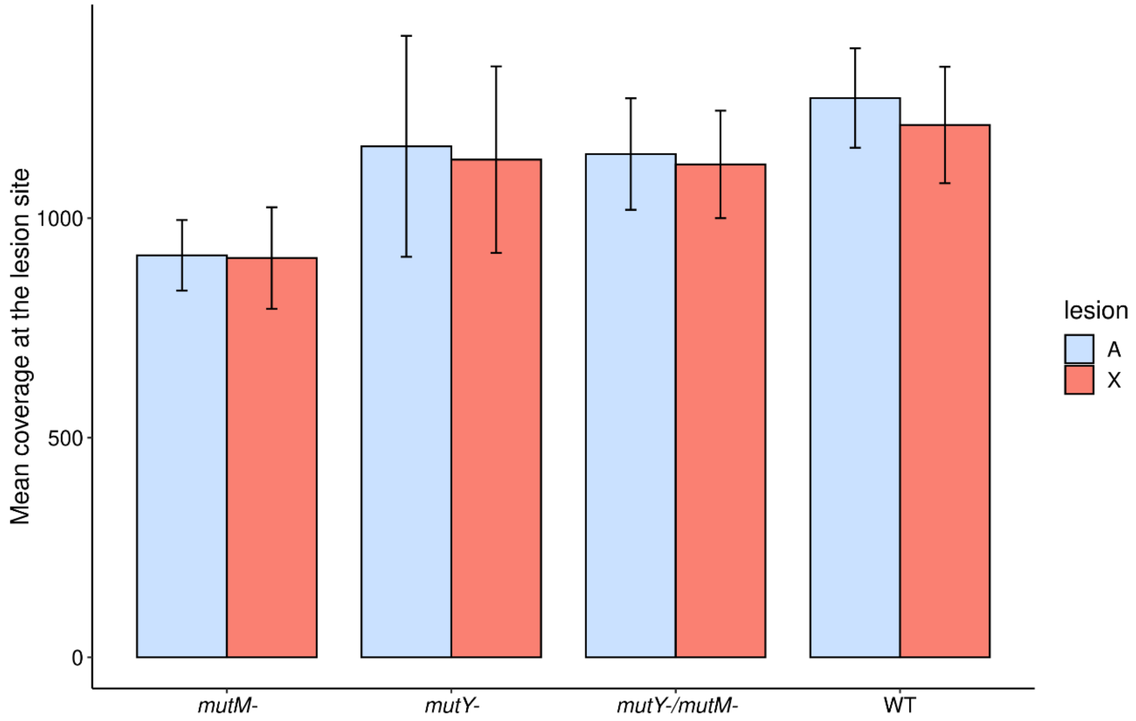

Fig. S10. Sequencing coverage at the lesion site. A = adenine. X = oxo-εA. The x-axis indicates the four *E. coli* genotypes analyzed. Data indicate the average number of reads available after sequencing data analysis for each genome and genotype. Error bars denote standard deviations for all technical and biological replicates (n=9).

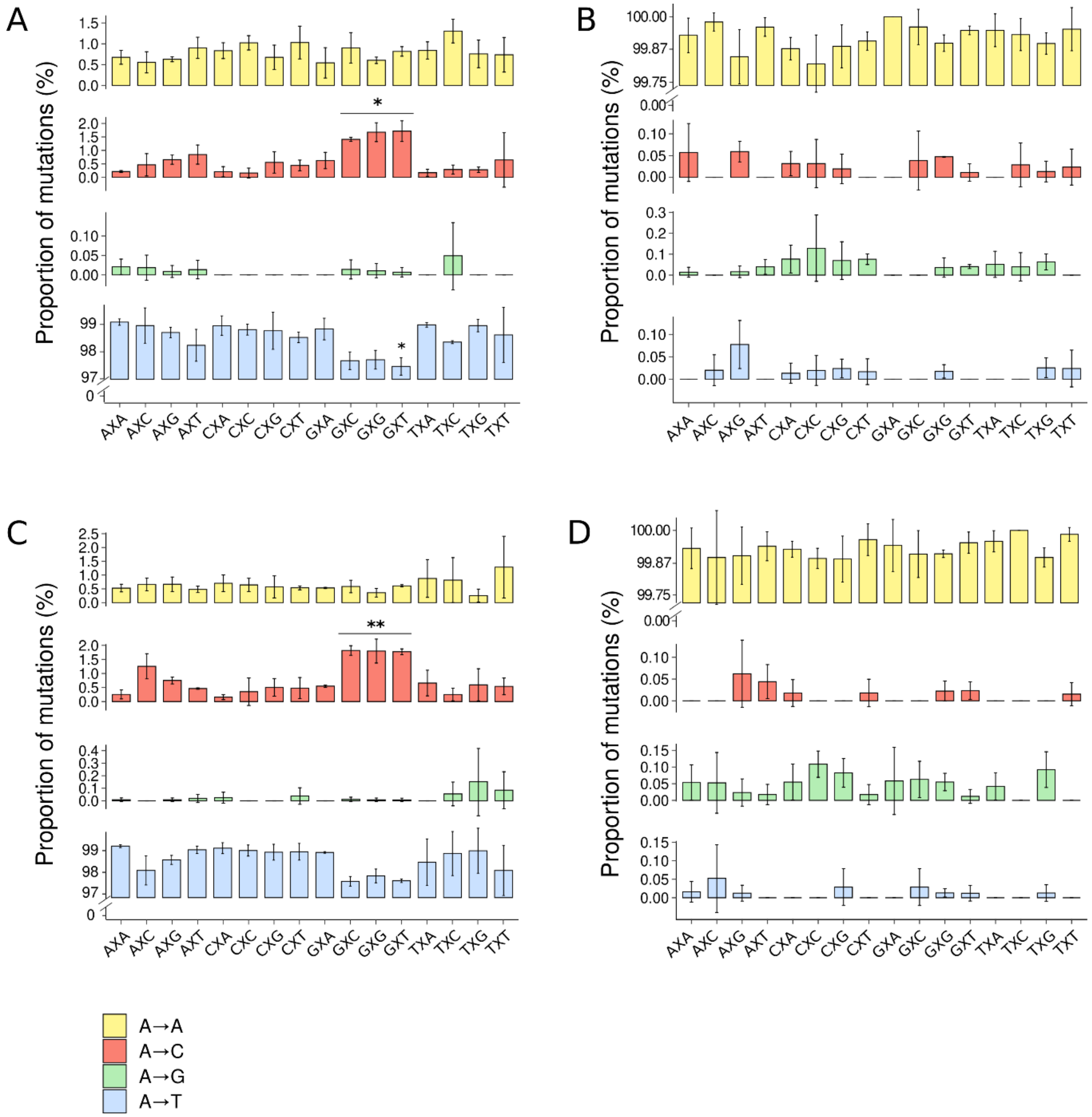

Fig. S11. Sequence context-dependent mutagenic properties of oxo-εA in *E. coli* cells possessing and lacking the AlkB protein. (A) Mutagenesis of oxo-εA in the WT strain (*alkB*+). (B) Mutagenesis of the unmodified A control genome in the WT strain (*alkB*+). (C) Mutagenesis of oxo-εA in *alkB*- (AlkB-deficient) cells. (D) Mutagenesis of the A control in the *alkB*- strain. The x-axis shows the trinucleotide contexts in which the modification or control bases were evaluated. The y-axis denotes relative percentage composition of a base at the lesion site (with respect to the total coverage).

Tables S1 to S12

Table S1. Oligodeoxynucleotide (ODN) sequences and MS data.

code	Sequence 5`→3`	m/z found (calculated)
ODN1	GCA<u>A**X**A</u>TACG	3084.6 (3084.1)
ODN2	GCA<u>T**X**T</u>TACG	3071.8 (3066.0)
ODN3	GCA<u>C**X**C</u>TACG	3039.4 (3036.1)
ODN4	GCA<u>G**X**G</u>TACG	3118.9 (3116.1)
ODN5	GCA<u>G**X**C</u>TACG	3078.0 (3076.1)
ODN6	GCA<u>G**X**T</u>TACG	3093.0 (3091.1)
ODN7	GCA<u>T**X**C</u>TACG	3053.1 (3051.1)
ODN8	GCA<u>T**X**G</u>TACG	3093.0 (3091.1)
ODN9	GTA<u>C**X**C</u>ATG	2763.4 (2761.9)
ODN10	CATGAGTAC	-
ODN11	GACTATTCCCTATAGTGAGTCGTATTA	-
ODN12	GACTAT**X**CCCTATAGTGAGTCGTATTA	8313.4 (8309.1)
ODN13	Cy5-TAATACGACTCACTATAGGG	6989.9 (6987.3)
ODN14	GAAGACC<u>A**X**A</u>GCGTCC	4923.8 (4923.3)
ODN15	GAAGACC<u>A**X**C</u>GCGTCC	4899.6 (4899.3)
ODN16	GAAGACC<u>A**X**G</u>GCGTCC	4939.9 (4939.2)
ODN17	GAAGACC<u>A**X**T</u>GCGTCC	4914.8 (4914.2)
ODN18	GAAGACC<u>C**X**A</u>GCGTCC	4899.5 (4899.2)
ODN19	GAAGACC<u>C**X**C</u>GCGTCC	4875.5 (4875.2)
ODN20	GAAGACC<u>C**X**G</u>GCGTCC	4915.6 (4915.2)
ODN21	GAAGACC<u>C**X**T</u>GCGTCC	4890.5 (4890.2)
ODN22	GAAGACC<u>G**X**A</u>GCGTCC	4939.5 (4939.2)
ODN23	GAAGACC<u>G**X**C</u>GCGTCC	4915.4 (4915.2)
ODN24	GAAGACC<u>G**X**G</u>GCGTCC	4955.6 (4955.2)
ODN25	GAAGACC<u>G**X**T</u>GCGTCC	4930.1 (4930.2)
ODN26	GAAGACC<u>T**X**A</u>GCGTCC	4914.1 (4914.2)
ODN27	GAAGACC<u>T**X**C</u>GCGTCC	4890.6 (4890.2)
ODN28	GAAGACC<u>T**X**G</u>GCGTCC	4930.4 (4930.2)
ODN29	GAAGACC<u>T**X**T</u>GCGTCC	4905.2 (4905.2)

X = oxo-εA nucleotide

Table S2. Thermal stabilities (T_m) of native and modified duplexes.

Code (central triplet) of strand 1*	T_m values of modified duplexes with A/G/C/T opposite X (central triplets in strand 2)			
	A	G	C	T
ODN1<br>(A**X**A)	(TAT)<br>35 (-4)**	(TGT)<br>29 (-4)	(TCT)<br>25 (+2)	(TTT)<br>18 (-8)
ODN2<br>(T**X**T)	(AAA)<br>37 (-7)	(AGA)<br>27 (-3)	(ACA)<br>24 (-2)	(ATA)<br>27 (+8)
ODN 3<br>(C**X**C)	(GAG)<br>35 (-6)	(GGG)<br>28 (+4)	(GCG)<br>14 (+2)	(GTG)<br>20 (-1)
ODN4<br>(G**X**G)	(CAC)<br>43 (-5)	(CGC)<br>29 (+4)	(CCC)<br>20 (+10)	(CTC)<br>23 (0)
ODN5<br>(G**X**C)	(GAC)<br>48 (+6)	(GGC)<br>33 (-3)	(GCC)<br>8 (-4)	(GTC)<br>18 (-8)
ODN6<br>(G**X**T)	(AAC)<br>46 (+10)	(AGC)<br>35 (+9)	(ACC)<br>27 (+15)	(ATC)<br>35 (+23)
ODN7<br>(T**X**C)	(GAA)<br>42 (+4)	(GGA)<br>28 (-5)	(GCA)<br>7 (-7)	(GTA)<br>28 (+4)
ODN8<br>(T**X**G)	(CAA)<br>35 (-8)	(CGA)<br>28 (-8)	(CCA)<br>21 (-10)	(CTA)<br>26 (-8)

* **X** = oxo-εA nucleotide

** ΔT_m is shown in brackets ($\Delta Tm = Tm$ modified duplex – Tm_native duplex). Native duplexes are those harboring T instead of X in the central triplet of strand 1, ±1°C (ΔT_m ±2°C)

Table S3. Thermodynamic parameters of duplex formation from single strands.

Strand 1/2	Delta H, kcal/mol				Delta S, cal/(mol*K)			
	TAT	TGT	TCT	TTT	TAT	TGT	TCT	TTT
ATA	-69 ± 2	-65 ± 2	-48 ± 2	-74 ± 2	-196 ± 6	-187 ± 4	-136 ± 6	-223 ± 9
AXA	-40 ± 1	-43 ± 1	-72 ± 2	-82 ± 2	-103 ± 2	-117 ± 2	-214 ± 6	-255 ± 2
	CAC	CGC	CCC	CTC	CAC	CGC	CCC	CTC
GTG	-61 ± 2	-29.1 ± 0.4	-38 ± 2	-38.8 ± 0.6	-164 ± 4	-72 ± 1	-107 ± 8	-106 ± 2
GXG	-39.5 ± 0.6	-34.4 ± 0.6	-33 ± 4	-34.4 ± 0.9	-99 ± 2	-88 ± 2	-88 ± 9	-90 ± 6
	GAG	GGG	GCG	GTG	GAG	GGG	GCG	GTG
CTC	-55 ± 1	-24.2 ± 0.2	-42.1 ± 0.2	-41.5 ± 0.6	-148 ± 5	-55.8 ± 0.8	-122 ± 3	-116 ± 3
CXC	-29.2 ± 0.6	-25.4 ± 0.4	-24.8 ± 0.3	-33.6 ± 0.5	-69 ± 3	-58.6 ± 0.9	-60.7 ± 0.2	-89 ± 1
	AAA	AGA	ACA	ATA	AAA	AGA	ACA	ATA
TTT	-58.5 ± 0.8	-39.4 ± 0.5	-44.0 ± 0.8	-76.0 ± 0.2	-159 ± 4	-104 ± 2	-121 ± 2	-235 ± 3
TXT	-61 ± 2	-80 ± 2	-64 ± 2	-74 ± 3	-172 ± 6	-241 ± 5	-189 ± 7	-221 ± 7
	GAC	GTC	GCC	GGC	GAC	GTC	GCC	GGC
GTC	-68 ± 6	-36 ± 3	-22.6 ± 0.7	-48 ± 3	-190 ± 20	-94 ± 9	-52 ± 2	-130 ± 8
GXC	-57 ± 4	-39 ± 3	-31 ± 5	-50 ± 3	-150 ± 10	-105 ± 9	-84 ± 9	-135 ± 8
	AAC	ATC	ACC	AGC	AAC	ATC	ACC	AGC
GTT	-32 ± 2	-27.5 ± 0.7	-34 ± 3	-53 ± 2	-76 ± 5	-70 ± 3	-92 ± 9	-150 ± 7
GXT	-40 ± 1	-44 ± 2	-55 ± 8	-45 ± 2	-99 ± 4	-115 ± 7	-160 ± 20	-119 ± 5
	GAA	GTA	GCA	GGA	GAA	GTA	GCA	GGA
TTC	-38 ± 1	-45 ± 4	-23 ± 5	-59 ± 6	-94 ± 4	-125 ± 8	-53 ± 5	-166 ± 9
TXC	-34.6 ± 0.7	-41 ± 2	-35 ± 5	-53 ± 2	-83 ± 2	-108 ± 6	-97 ± 9	-148 ± 6
	CAA	CTA	CCA	CGA	CAA	CTA	CCA	CGA
TTG	-53.6 ± 0.8	-42 ± 1	-56 ± 2	-55 ± 2	-142 ± 3	-110 ± 3	-158 ± 5	-152 ± 8
TXG	-37 ± 2	-45 ± 3	-54 ± 5	-47 ± 3	-93 ± 6	-122 ± 9	-155 ± 9	-130 ± 8

Table S4: ^{1}H NMR assignment list of **[EDA]** duplex. (Chemical shifts are in ppm, n.o = not observed).

Assignment list										
Residue	H1/H3	H41/H21	H42/H22	H6/H8	H2/H5/Me	H1’	H2’	H2’’	H3’	H4’
G1	n.o	n.o	n.o	7.58	-	5.61	2.34	2.43	4.48	3.89
T2	13.35	-	-	7.15	1.05	5.41	1.92	2.23	4.60	3.93
A3	-	n.o.	n.o.	7.94	7.03	5.78	2.31	2.43	4.70	n.o
C4	-	6.37	7.52	6.53	4.37	5.31	1.17	1.83	4.44	3.81
X5	H2=8.53 H10=7.32 H11=6.96 H7 =13.47					5.63	2.26	2.99	4.64	3.93
C6	-	5.84	7.17	7.23	4.98	4.86	1.87	2.03	4.53	3.82
A7	-	n.o.	n.o.	8.00	7.29	5.92	2.38	2.59	4.71	4.12
T8	13.37	-	-	6.81	1.17	5.43	1.53	1.98	4.52	3.79
G9	n.o.	n.o	n.o	7.52	-	5.76	1.99	2.28	4.35	3.84
C10	-	6.90	7.91	7.39	5.58	5.30	1.72	2.12	4.39	3.74
A11	-	n.o.	n.o.	8.10	7.45	5.96	2.44	2.63	4.71	4.10
T12	13.18	-	-	6.81	1.02	5.30	1.58	1.95	4.52	3.85
G13	12.00	n.o.	n.o.	7.59	-	5.00	2.37	2.40	4.68	3.99
A14	-	5.72	9.62	7.84	7.44	5.78	2.44	2.62	4.15	n.o.
G15	12.37	n.o.	n.o.	7.35	-	5.52	2.18	2.40	4.62	4.08
T16	13.26	-	-	6.84	1.10	5.33	1.68	2.09	4.52	3.85
A17	-	n.o.	n.o.	7.93	7.16	5.90	2.32	2.54	4.68	4.08
C18	-	6.64	7.88	6.99	4.90	5.68	1.75	1.79	4.14	3.67

Table S5. Experimental constraints and calculation statistics of [**EDA**] duplex.

EXPERIMENTAL DISTANCE CONSTRAINTS			
RESTRAINTS	Total number		196
	Intra-residue		57
	Sequential		115
	Range > 1		24
RMSD [Å]	Bases	Well-defined+	0.35±0.08
	Heavy atoms	All	0.68±0.11
		Well-defined+	0.57±0.11
	Backbone	All	0.82±0.13
		Well-defined+	0.66±0.14
Violations [Å]	Total sum	Average	2.69
		Range	2.54 … 2.93
	Maximum	Average	0.31
		Range	0.29 … 0.36
Energy *[kcal/mol]*	NOE	Average	14.15
		Range	13.27 … 15.71
	Total	Average	-995
		Range	-1086 … -899

+ All except terminal residues

Table S6: Experimental distance constraints involving protons of *oxo-εA* residue in **[EDA]** duplex.[+]

[EDA] duplex				
Residues				*Distance/ Å*
5 EDA	H2	4 DC	H1’	4
			H2’	4
			H5	5
		6 DC	H41	4
			H5	4
	H11	4 DC	H41	4
			H6	4
			H5	4
		6 DC	H41	5
		14 DA	H62	4
	H10	4 DC	H41	5
			H6	3
			H5	4
			H1’	5.5
			H2’	4
			H2’’	5.5
		6 DC	H41	5.5
			H5	6
	H7	14 DA	H2	4
	H1’	6 DC	H6	4.5
	H2’			3
	H2’’			4
			H5	4

[+] Most distances involving nucleobase protons of oxo-εA are only consistent with a syn conformation of oxo-εA. If the glycosidic angle of oxo-εA is flipped to the anti conformation, most of these distances are larger than 6 Å, and the corresponding NOEs would be very weak or undetectable, in contrast to the experimental data.

Table S7: Average dihedral angles and order parameters of the structure of **[EDA]** duplex.

	**Pseudorot**		**α**		**β**		**γ**		**δ**		**ε**		**ζ**		**χ**	
	**P**	**Am**	**A**	**O.P**	**A**	**O. P**	**A**	**O. P**	**A**	**O. P**	**A**	**O. P**	**A**	**O. P**	**A**	**O. P**
1 G	177.3	32					53.2	0.7	143.5	1.0	-167.9	1.0	-89.9	1.0	-124.5	1.0
2 T	143.1	42	-71.1	1.0	175.8	1.0	52.4	1.0	130.8	1.0	-177.9	1.0	-100.8	1.0	-109.8	1.0
3 A	150.2	38	-72.1	1.0	-173.3	1.0	52.1	1.0	132.8	1.0	-175.6	1.0	-97.4	1.0	-116.0	1.0
4 C	143.5	43	-68.2	1.0	175.2	1.0	58.9	1.0	134.7	1.0	-156.2	0.9	-127.9	0.9	-111.6	1.0
5 EDA	146.2	34	-72.4	0.6	178.3	1.0	66.1	0.7	129.3	1.0	-175.5	1.0	-86.6	1.0	65.0	1.0
6 C	113.2	38	-66.3	1.0	164.7	1.0	59.8	1.0	107.1	1.0	-168.9	1.0	-89.1	1.0	-127.1	1.0
7 A	143.9	39	-74.0	1.0	178.4	1.0	51.5	1.0	129.5	1.0	-178.2	1.0	-103.1	1.0	-109.9	1.0
8 T	146.7	40	-68.3	1.0	177.2	1.0	58.5	1.0	133.6	1.0	-177.5	1.0	-96.2	1.0	-112.8	1.0
9 G	143.1	44	-70.7	1.0	-178.3	1.0	51.1	1.0	135.8	1.0					-119.0	1.0
10 C	149.8	41					55.0	0.8	136.3	1.0	-169.0	1.0	-97	1.0	-122.7	1.0
11 A	162.5	37	-80.5	1.0	-166.2	1.0	46.8	1.0	138.2	1.0	179.8	1.0	-95.3	1.0	-109.4	1.0
12 T	134.3	40	-69.4	1.0	177.7	1.0	55.1	1.0	123.9	1.0	-167.2	1.0	-91.6	1.0	-121.0	1.0
13 G	136.7	43	-76.4	1.0	176.4	1.0	50.6	1.0	127.2	1.0	-162.5	0.9	-127.8	0.8	-105.8	1.0
14 A	149.3	36	-67.3	1.0	168.7	1.0	53.2	1.0	132.0	1.0	-173.2	1.0	-94.3	1.0	-114.9	1.0
15 G	137.5	39	-69.2	1.0	176.2	1.0	55.5	1.0	125.0	1.0	-176.4	1.0	-108.1	1.0	-117.2	1.0
16 T	154.0	36	-67.7	1.0	-179.6	1.0	58.3	1.0	134.9	1.0	178.5	1.0	-95.8	1.0	-111.3	1.0
17 A	142.2	41	-67.0	1.0	-179.7	1.0	55.2	1.0	130.7	1.0	-173.3	1.0	-93.9	1.0	-121.3	1.0
18 C	138.2	45	-71.3	1.0	179.6	1.0	52.1	1.0	130.9	1.0					-121.2	1.0

Table S8. Percentage of snapshots from MD simulations containing hydrogen bonds in X^S–A and X^A–A base pairs.

donor	acceptor	occupancy
X^S-NH	dA-N	87,55
dA-NH	X^S-N	50,64
dA-NH	X^A-N	39,37

Table S9. Percentage of mutations. Experiment: A and oxo-εA (X) in WT, *mutM-*, *mutY-*, *mutM-/mutY-* backgrounds.

Cell line	Lesion/control nucleotide	%T mean	% T SD	% G mean	% G SD	% C mean	% C SD
WT	A	1.377	0.383	1.609	0.072	0.036	0.020
mutY-	A	1.338	0.326	1.147	0.077	0.042	0.051
mutM-	A	1.801	0.219	1.566	0.090	0.063	0.110
mutY-/mutM-	A	1.573	0.123	1.018	0.120	0.018	0.016
WT	X	96.164	0.201	1.507	0.133	0.703	0.206
mutY-	X	96.793	0.574	1.436	0.206	0.696	0.440
mutM-	X	96.727	0.103	1.427	0.034	0.277	0.093
mutY-/mutM-	X	96.692	0.335	1.573	0.181	0.504	0.297

Table S10. Intra-group ANOVA with Tukey post-hoc test. Experiment: A and oxo-εA (X) in WT, *mutM-*, *mutY-*, *mutM-/mutY-* backgrounds. **group** = mutation type (A/X > T/G/C). ANOVA is performed among all strains for a mutation type. **comparisons** – pairwise comparisons between cell lines within one group (one mutation type). **diff** – difference between means. **lwr, upr** – confidence levels. p **adj** – p-value adjusted by Tukey test. Yellow color – p adj < 0.05

group	comparisons	diff	lwr	upr	p adj
A>C	*mutY-_vs_mutM-*	-0.0214	-0.1832	0.1405	0.9730
A>C	*mutY-/mutM-_vs_mutM-*	-0.0455	-0.2074	0.1164	0.8052
A>C	WT_*vs_mutM-*	-0.0278	-0.1897	0.1341	0.9440
A>C	*mutY-/mutM-_vs_mutY-*	-0.0241	-0.1860	0.1377	0.9620
A>C	WT_*vs_mutY-*	-0.0064	-0.1683	0.1555	0.9992
A>C	WT_*vs_mutY-/mutM-*	0.0177	-0.1442	0.1796	0.9841
A>G	*mutY-_vs_mutM-*	-0.4192	-0.6584	-0.1800	0.0022
A>G	*mutY-/mutM-_vs_mutM-*	-0.5484	-0.7876	-0.3093	0.0004
A>G	WT_*vs_mutM-*	0.0426	-0.1966	0.2818	0.9382
A>G	*mutY-/mutM-_vs_mutY-*	-0.1293	-0.3684	0.1099	0.3694
A>G	WT_*vs_mutY-*	0.4618	0.2226	0.7010	0.0012
A>G	WT_*vs_mutY-/mutM-*	0.5910	0.3519	0.8302	0.0002
A>T	*mutY-_vs_mutM-*	-0.4631	-1.1982	0.2720	0.2581
A>T	*mutY-/mutM-_vs_mutM-*	-0.2278	-0.9629	0.5073	0.7578
A>T	WT_*vs_mutM-*	-0.4242	-1.1592	0.3109	0.3203
A>T	*mutY-/mutM-_vs_mutY-*	0.2353	-0.4998	0.9704	0.7404
A>T	WT_*vs_mutY-*	0.0390	-0.6961	0.7741	0.9981

A>T	WT_*vs_mutY-/mutM-*	-0.1963	-0.9314	0.5388	0.8271
X>C	*mutY-_vs_mutM-*	0.4182	-0.3355	1.1720	0.3494
X>C	*mutY-/mutM-_vs_mutM-*	0.2265	-0.5272	0.9802	0.7737
X>C	WT_*vs_mutM-*	0.4257	-0.3280	1.1795	0.3360
X>C	*mutY-/mutM-_vs_mutY-*	-0.1917	-0.9455	0.5620	0.8461
X>C	WT_*vs_mutY-*	0.0075	-0.7462	0.7612	1.0000
X>C	WT_*vs_mutY-/mutM-*	0.1992	-0.5545	0.9530	0.8313
X>G	*mutY-_vs_mutM-*	0.0093	-0.3917	0.4103	0.9998
X>G	*mutY-/mutM-_vs_mutM-*	0.1462	-0.2548	0.5473	0.6617
X>G	WT_*vs_mutM-*	0.0796	-0.3214	0.4806	0.9176
X>G	*mutY-/mutM-_vs_mutY-*	0.1369	-0.2641	0.5379	0.7031
X>G	WT_*vs_mutY-*	0.0703	-0.3307	0.4713	0.9408
X>G	WT_*vs_mutY-/mutM-*	-0.0666	-0.4677	0.3344	0.9488
X>T	*mutY-_vs_mutM-*	0.0666	-0.8514	0.9846	0.9952
X>T	*mutY-/mutM-_vs_mutM-*	-0.0347	-0.9527	0.8833	0.9993
X>T	WT_*vs_mutM-*	-0.5628	-1.4808	0.3551	0.2768
X>T	*mutY-/mutM-_vs_mutY-*	-0.1014	-1.0194	0.8166	0.9838
X>T	WT_*vs_mutY-*	-0.6295	-1.5475	0.2885	0.2039
X>T	WT_*vs_mutY-/mutM-*	-0.5281	-1.4461	0.3899	0.3225

Table S11. Percentage of mutations. Experiment: A and oxo-εA (X) in WT and *alkB* backgrounds.

Lesion/control nucleotide	Cell line	Trinucleotide context	% T mean	% T SD	% G mean	% G SD	% C mean	% C SD
A	*alkB*-	AXA	0.02	0.02	0.05	0.05	0.00	0.00
A	*alkB*-	AXC	0.05	0.05	0.05	0.09	0.00	0.00
A	*alkB*-	AXG	0.01	0.01	0.02	0.04	0.06	0.08
A	*alkB*-	AXT	0.00	0.00	0.02	0.03	0.04	0.04
A	*alkB*-	CXA	0.00	0.00	0.05	0.05	0.02	0.03
A	*alkB*-	CXC	0.00	0.00	0.11	0.04	0.00	0.00
A	*alkB*-	CXG	0.03	0.03	0.08	0.04	0.00	0.00
A	*alkB*-	CXT	0.00	0.00	0.02	0.03	0.02	0.03
A	*alkB*-	GXA	0.00	0.00	0.06	0.10	0.00	0.00
A	*alkB*-	GXC	0.03	0.03	0.06	0.05	0.00	0.00
A	*alkB*-	GXG	0.01	0.01	0.06	0.03	0.02	0.02
A	*alkB*-	GXT	0.01	0.01	0.01	0.02	0.02	0.02
A	*alkB*-	TXA	0.00	0.00	0.04	0.04	0.00	0.00
A	*alkB*-	TXC	0.00	0.00	0.00	0.00	0.00	0.00
A	*alkB*-	TXG	0.01	0.01	0.09	0.05	0.00	0.00
A	*alkB*-	TXT	0.00	0.00	0.00	0.00	0.02	0.03
A	WT	AXA	0.00	0.00	0.01	0.02	0.06	0.07
A	WT	AXC	0.02	0.02	0.00	0.00	0.00	0.00
A	WT	AXG	0.08	0.08	0.02	0.03	0.06	0.02
A	WT	AXT	0.00	0.00	0.04	0.04	0.00	0.00
A	WT	CXA	0.01	0.01	0.08	0.07	0.03	0.03
A	WT	CXC	0.02	0.02	0.13	0.16	0.03	0.06
A	WT	CXG	0.02	0.02	0.07	0.09	0.02	0.03
A	WT	CXT	0.02	0.02	0.08	0.03	0.00	0.00
A	WT	GXA	0.00	0.00	0.00	0.00	0.00	0.00
A	WT	GXC	0.00	0.00	0.00	0.00	0.04	0.07
A	WT	GXG	0.02	0.02	0.04	0.05	0.05	0.00
A	WT	GXT	0.00	0.00	0.04	0.01	0.01	0.02
A	WT	TXA	0.00	0.00	0.05	0.06	0.00	0.00
A	WT	TXC	0.00	0.00	0.04	0.07	0.03	0.05
A	WT	TXG	0.03	0.03	0.06	0.04	0.01	0.02
A	WT	TXT	0.02	0.02	0.00	0.00	0.02	0.04
X	*alkB*-	AXA	99.21	99.21	0.01	0.01	0.25	0.16
X	*alkB*-	AXC	98.09	98.09	0.00	0.00	1.26	0.45

X	*alkB*-	AXG	98.57	98.57	0.01	0.01	0.75	0.12
X	*alkB*-	AXT	99.04	99.04	0.02	0.03	0.46	0.02
X	*alkB*-	CXA	99.12	99.12	0.03	0.04	0.16	0.09
X	*alkB*-	CXC	99.01	99.01	0.00	0.00	0.35	0.49
X	*alkB*-	CXG	98.93	98.93	0.00	0.00	0.50	0.31
X	*alkB*-	CXT	98.95	98.95	0.04	0.07	0.48	0.38
X	*alkB*-	GXA	98.92	98.92	0.00	0.00	0.55	0.04
X	*alkB*-	GXC	97.58	97.58	0.01	0.02	1.82	0.17
X	*alkB*-	GXG	97.83	97.83	0.01	0.01	1.80	0.43
X	*alkB*-	GXT	97.61	97.61	0.01	0.01	1.77	0.10
X	*alkB*-	TXA	98.46	98.46	0.00	0.00	0.66	0.46
X	*alkB*-	TXC	98.88	98.88	0.05	0.09	0.25	0.23
X	*alkB*-	TXG	99.00	99.00	0.15	0.26	0.60	0.57
X	*alkB*-	TXT	98.09	98.09	0.08	0.15	0.54	0.30
X	WT	AXA	99.08	99.08	0.02	0.02	0.22	0.04
X	WT	AXC	98.96	98.96	0.02	0.03	0.47	0.41
X	WT	AXG	98.70	98.70	0.01	0.02	0.66	0.17
X	WT	AXT	98.24	98.24	0.01	0.02	0.85	0.35
X	WT	CXA	98.95	98.95	0.00	0.00	0.21	0.19
X	WT	CXC	98.81	98.81	0.00	0.00	0.17	0.18
X	WT	CXG	98.77	98.77	0.00	0.00	0.56	0.40
X	WT	CXT	98.53	98.53	0.00	0.00	0.44	0.20
X	WT	GXA	98.83	98.83	0.00	0.00	0.62	0.30
X	WT	GXC	97.67	97.67	0.01	0.02	1.41	0.07
X	WT	GXG	97.71	97.71	0.01	0.02	1.68	0.35
X	WT	GXT	97.46	97.46	0.01	0.01	1.71	0.38
X	WT	TXA	98.98	98.98	0.00	0.00	0.17	0.13
X	WT	TXC	98.35	98.35	0.05	0.09	0.29	0.17
X	WT	TXG	98.96	98.96	0.00	0.00	0.29	0.10
X	WT	TXT	98.62	98.62	0.00	0.00	0.65	1.01

Table S12. Intra-group ANOVA with Tukey post-hoc test. Experiment: A and oxo-εA (X) in WT and *alkB*- backgrounds. **cell line** – cell line. **mutation** - mutation type (A/X > T/G/C). **comparisons** – pairwise comparisons between trinucleotide lesion contexts for each group (cell line plus mutation type). ANOVA is performed among all sequence contexts for a group. **diff** – difference between means. **lwr, upr** – confidence levels. p **adj** – p-value adjusted by Tukey test.

cell line	mutation	comparisons	diff	lwr	upr	p adj
WT	A>C	AXC-AXA	-0.06	-0.16	0.05	0.81
WT	A>C	AXG-AXA	0.00	-0.10	0.11	1.00
WT	A>C	AXT-AXA	-0.06	-0.16	0.05	0.81
WT	A>C	CXA-AXA	-0.03	-0.13	0.08	1.00
WT	A>C	CXC-AXA	-0.03	-0.13	0.08	1.00
WT	A>C	CXG-AXA	-0.04	-0.14	0.07	0.99
WT	A>C	CXT-AXA	-0.06	-0.16	0.05	0.81
WT	A>C	GXA-AXA	-0.06	-0.16	0.05	0.81
WT	A>C	GXC-AXA	-0.02	-0.12	0.09	1.00
WT	A>C	GXG-AXA	-0.01	-0.12	0.10	1.00
WT	A>C	GXT-AXA	-0.05	-0.15	0.06	0.96
WT	A>C	TXA-AXA	-0.06	-0.16	0.05	0.81
WT	A>C	TXC-AXA	-0.03	-0.13	0.08	1.00
WT	A>C	TXG-AXA	-0.04	-0.15	0.06	0.97
WT	A>C	TXT-AXA	-0.03	-0.14	0.07	1.00
WT	A>C	AXG-AXC	0.06	-0.05	0.17	0.77
WT	A>C	AXT-AXC	0.00	-0.11	0.11	1.00
WT	A>C	CXA-AXC	0.03	-0.07	0.14	1.00
WT	A>C	CXC-AXC	0.03	-0.07	0.14	1.00
WT	A>C	CXG-AXC	0.02	-0.09	0.13	1.00
WT	A>C	CXT-AXC	0.00	-0.11	0.11	1.00
WT	A>C	GXA-AXC	0.00	-0.11	0.11	1.00
WT	A>C	GXC-AXC	0.04	-0.07	0.14	0.99
WT	A>C	GXG-AXC	0.05	-0.06	0.15	0.94
WT	A>C	GXT-AXC	0.01	-0.09	0.12	1.00
WT	A>C	TXA-AXC	0.00	-0.11	0.11	1.00
WT	A>C	TXC-AXC	0.03	-0.08	0.13	1.00
WT	A>C	TXG-AXC	0.01	-0.09	0.12	1.00
WT	A>C	TXT-AXC	0.02	-0.08	0.13	1.00
WT	A>C	AXT-AXG	-0.06	-0.17	0.05	0.77
WT	A>C	CXA-AXG	-0.03	-0.13	0.08	1.00
WT	A>C	CXC-AXG	-0.03	-0.13	0.08	1.00

WT	A>C	CXG-AXG	-0.04	-0.15	0.07	0.99
WT	A>C	CXT-AXG	-0.06	-0.17	0.05	0.77
WT	A>C	GXA-AXG	-0.06	-0.17	0.05	0.77
WT	A>C	GXC-AXG	-0.02	-0.13	0.09	1.00
WT	A>C	GXG-AXG	-0.01	-0.12	0.09	1.00
WT	A>C	GXT-AXG	-0.05	-0.15	0.06	0.94
WT	A>C	TXA-AXG	-0.06	-0.17	0.05	0.77
WT	A>C	TXC-AXG	-0.03	-0.14	0.08	1.00
WT	A>C	TXG-AXG	-0.05	-0.15	0.06	0.96
WT	A>C	TXT-AXG	-0.04	-0.14	0.07	1.00
WT	A>C	CXA-AXT	0.03	-0.07	0.14	1.00
WT	A>C	CXC-AXT	0.03	-0.07	0.14	1.00
WT	A>C	CXG-AXT	0.02	-0.09	0.13	1.00
WT	A>C	CXT-AXT	0.00	-0.11	0.11	1.00
WT	A>C	GXA-AXT	0.00	-0.11	0.11	1.00
WT	A>C	GXC-AXT	0.04	-0.07	0.14	0.99
WT	A>C	GXG-AXT	0.05	-0.06	0.15	0.94
WT	A>C	GXT-AXT	0.01	-0.09	0.12	1.00
WT	A>C	TXA-AXT	0.00	-0.11	0.11	1.00
WT	A>C	TXC-AXT	0.03	-0.08	0.13	1.00
WT	A>C	TXG-AXT	0.01	-0.09	0.12	1.00
WT	A>C	TXT-AXT	0.02	-0.08	0.13	1.00
WT	A>C	CXC-CXA	0.00	-0.11	0.11	1.00
WT	A>C	CXG-CXA	-0.01	-0.12	0.09	1.00
WT	A>C	CXT-CXA	-0.03	-0.14	0.07	1.00
WT	A>C	GXA-CXA	-0.03	-0.14	0.07	1.00
WT	A>C	GXC-CXA	0.01	-0.10	0.11	1.00
WT	A>C	GXG-CXA	0.02	-0.09	0.12	1.00
WT	A>C	GXT-CXA	-0.02	-0.13	0.09	1.00
WT	A>C	TXA-CXA	-0.03	-0.14	0.07	1.00
WT	A>C	TXC-CXA	0.00	-0.11	0.10	1.00
WT	A>C	TXG-CXA	-0.02	-0.12	0.09	1.00
WT	A>C	TXT-CXA	-0.01	-0.11	0.10	1.00
WT	A>C	CXG-CXC	-0.01	-0.12	0.09	1.00
WT	A>C	CXT-CXC	-0.03	-0.14	0.07	1.00
WT	A>C	GXA-CXC	-0.03	-0.14	0.07	1.00
WT	A>C	GXC-CXC	0.01	-0.10	0.11	1.00
WT	A>C	GXG-CXC	0.02	-0.09	0.12	1.00
WT	A>C	GXT-CXC	-0.02	-0.13	0.09	1.00
WT	A>C	TXA-CXC	-0.03	-0.14	0.07	1.00

WT	A>C	TXC-CXC	0.00	-0.11	0.10	1.00
WT	A>C	TXG-CXC	-0.02	-0.12	0.09	1.00
WT	A>C	TXT-CXC	-0.01	-0.11	0.10	1.00
WT	A>C	CXT-CXG	-0.02	-0.13	0.09	1.00
WT	A>C	GXA-CXG	-0.02	-0.13	0.09	1.00
WT	A>C	GXC-CXG	0.02	-0.09	0.13	1.00
WT	A>C	GXG-CXG	0.03	-0.08	0.13	1.00
WT	A>C	GXT-CXG	-0.01	-0.11	0.10	1.00
WT	A>C	TXA-CXG	-0.02	-0.13	0.09	1.00
WT	A>C	TXC-CXG	0.01	-0.10	0.12	1.00
WT	A>C	TXG-CXG	-0.01	-0.11	0.10	1.00
WT	A>C	TXT-CXG	0.00	-0.10	0.11	1.00
WT	A>C	GXA-CXT	0.00	-0.11	0.11	1.00
WT	A>C	GXC-CXT	0.04	-0.07	0.14	0.99
WT	A>C	GXG-CXT	0.05	-0.06	0.15	0.94
WT	A>C	GXT-CXT	0.01	-0.09	0.12	1.00
WT	A>C	TXA-CXT	0.00	-0.11	0.11	1.00
WT	A>C	TXC-CXT	0.03	-0.08	0.13	1.00
WT	A>C	TXG-CXT	0.01	-0.09	0.12	1.00
WT	A>C	TXT-CXT	0.02	-0.08	0.13	1.00
WT	A>C	GXC-GXA	0.04	-0.07	0.14	0.99
WT	A>C	GXG-GXA	0.05	-0.06	0.15	0.94
WT	A>C	GXT-GXA	0.01	-0.09	0.12	1.00
WT	A>C	TXA-GXA	0.00	-0.11	0.11	1.00
WT	A>C	TXC-GXA	0.03	-0.08	0.13	1.00
WT	A>C	TXG-GXA	0.01	-0.09	0.12	1.00
WT	A>C	TXT-GXA	0.02	-0.08	0.13	1.00
WT	A>C	GXG-GXC	0.01	-0.10	0.11	1.00
WT	A>C	GXT-GXC	-0.03	-0.13	0.08	1.00
WT	A>C	TXA-GXC	-0.04	-0.14	0.07	0.99
WT	A>C	TXC-GXC	-0.01	-0.12	0.10	1.00
WT	A>C	TXG-GXC	-0.03	-0.13	0.08	1.00
WT	A>C	TXT-GXC	-0.02	-0.12	0.09	1.00
WT	A>C	GXT-GXG	-0.04	-0.14	0.07	0.99
WT	A>C	TXA-GXG	-0.05	-0.15	0.06	0.94
WT	A>C	TXC-GXG	-0.02	-0.12	0.09	1.00
WT	A>C	TXG-GXG	-0.03	-0.14	0.07	1.00
WT	A>C	TXT-GXG	-0.02	-0.13	0.08	1.00
WT	A>C	TXA-GXT	-0.01	-0.12	0.09	1.00
WT	A>C	TXC-GXT	0.02	-0.09	0.12	1.00

WT	A>C	TXG-GXT	0.00	-0.10	0.11	1.00
WT	A>C	TXT-GXT	0.01	-0.09	0.12	1.00
WT	A>C	TXC-TXA	0.03	-0.08	0.13	1.00
WT	A>C	TXG-TXA	0.01	-0.09	0.12	1.00
WT	A>C	TXT-TXA	0.02	-0.08	0.13	1.00
WT	A>C	TXG-TXC	-0.02	-0.12	0.09	1.00
WT	A>C	TXT-TXC	-0.01	-0.11	0.10	1.00
WT	A>C	TXT-TXG	0.01	-0.10	0.12	1.00
alkB-	A>C	AXC-AXA	0.00	-0.08	0.08	1.00
alkB-	A>C	AXG-AXA	0.06	-0.02	0.14	0.27
alkB-	A>C	AXT-AXA	0.04	-0.04	0.12	0.78
alkB-	A>C	CXA-AXA	0.02	-0.06	0.10	1.00
alkB-	A>C	CXC-AXA	0.00	-0.08	0.08	1.00
alkB-	A>C	CXG-AXA	0.00	-0.08	0.08	1.00
alkB-	A>C	CXT-AXA	0.02	-0.06	0.10	1.00
alkB-	A>C	GXA-AXA	0.00	-0.08	0.08	1.00
alkB-	A>C	GXC-AXA	0.00	-0.08	0.08	1.00
alkB-	A>C	GXG-AXA	0.02	-0.06	0.10	1.00
alkB-	A>C	GXT-AXA	0.02	-0.06	0.10	1.00
alkB-	A>C	TXA-AXA	0.00	-0.08	0.08	1.00
alkB-	A>C	TXC-AXA	0.00	-0.08	0.08	1.00
alkB-	A>C	TXG-AXA	0.00	-0.08	0.08	1.00
alkB-	A>C	TXT-AXA	0.02	-0.06	0.10	1.00
alkB-	A>C	AXG-AXC	0.06	-0.02	0.14	0.27
alkB-	A>C	AXT-AXC	0.04	-0.04	0.12	0.78
alkB-	A>C	CXA-AXC	0.02	-0.06	0.10	1.00
alkB-	A>C	CXC-AXC	0.00	-0.08	0.08	1.00
alkB-	A>C	CXG-AXC	0.00	-0.08	0.08	1.00
alkB-	A>C	CXT-AXC	0.02	-0.06	0.10	1.00
alkB-	A>C	GXA-AXC	0.00	-0.08	0.08	1.00
alkB-	A>C	GXC-AXC	0.00	-0.08	0.08	1.00
alkB-	A>C	GXG-AXC	0.02	-0.06	0.10	1.00
alkB-	A>C	GXT-AXC	0.02	-0.06	0.10	1.00
alkB-	A>C	TXA-AXC	0.00	-0.08	0.08	1.00
alkB-	A>C	TXC-AXC	0.00	-0.08	0.08	1.00
alkB-	A>C	TXG-AXC	0.00	-0.08	0.08	1.00
alkB-	A>C	TXT-AXC	0.02	-0.06	0.10	1.00
alkB-	A>C	AXT-AXG	-0.02	-0.10	0.06	1.00
alkB-	A>C	CXA-AXG	-0.04	-0.12	0.04	0.77
alkB-	A>C	CXC-AXG	-0.06	-0.14	0.02	0.27

alkB-	A>C	CXG-AXG	-0.06	-0.14	0.02	0.27
alkB-	A>C	CXT-AXG	-0.04	-0.12	0.04	0.78
alkB-	A>C	GXA-AXG	-0.06	-0.14	0.02	0.27
alkB-	A>C	GXC-AXG	-0.06	-0.14	0.02	0.27
alkB-	A>C	GXG-AXG	-0.04	-0.12	0.04	0.88
alkB-	A>C	GXT-AXG	-0.04	-0.12	0.04	0.90
alkB-	A>C	TXA-AXG	-0.06	-0.14	0.02	0.27
alkB-	A>C	TXC-AXG	-0.06	-0.14	0.02	0.27
alkB-	A>C	TXG-AXG	-0.06	-0.14	0.02	0.27
alkB-	A>C	TXT-AXG	-0.05	-0.13	0.03	0.71
alkB-	A>C	CXA-AXT	-0.03	-0.11	0.05	1.00
alkB-	A>C	CXC-AXT	-0.04	-0.12	0.04	0.78
alkB-	A>C	CXG-AXT	-0.04	-0.12	0.04	0.78
alkB-	A>C	CXT-AXT	-0.03	-0.11	0.05	1.00
alkB-	A>C	GXA-AXT	-0.04	-0.12	0.04	0.78
alkB-	A>C	GXC-AXT	-0.04	-0.12	0.04	0.78
alkB-	A>C	GXG-AXT	-0.02	-0.10	0.06	1.00
alkB-	A>C	GXT-AXT	-0.02	-0.10	0.06	1.00
alkB-	A>C	TXA-AXT	-0.04	-0.12	0.04	0.78
alkB-	A>C	TXC-AXT	-0.04	-0.12	0.04	0.78
alkB-	A>C	TXG-AXT	-0.04	-0.12	0.04	0.78
alkB-	A>C	TXT-AXT	-0.03	-0.11	0.05	0.99
alkB-	A>C	CXC-CXA	-0.02	-0.10	0.06	1.00
alkB-	A>C	CXG-CXA	-0.02	-0.10	0.06	1.00
alkB-	A>C	CXT-CXA	0.00	-0.08	0.08	1.00
alkB-	A>C	GXA-CXA	-0.02	-0.10	0.06	1.00
alkB-	A>C	GXC-CXA	-0.02	-0.10	0.06	1.00
alkB-	A>C	GXG-CXA	0.00	-0.08	0.08	1.00
alkB-	A>C	GXT-CXA	0.01	-0.07	0.09	1.00
alkB-	A>C	TXA-CXA	-0.02	-0.10	0.06	1.00
alkB-	A>C	TXC-CXA	-0.02	-0.10	0.06	1.00
alkB-	A>C	TXG-CXA	-0.02	-0.10	0.06	1.00
alkB-	A>C	TXT-CXA	0.00	-0.08	0.08	1.00
alkB-	A>C	CXG-CXC	0.00	-0.08	0.08	1.00
alkB-	A>C	CXT-CXC	0.02	-0.06	0.10	1.00
alkB-	A>C	GXA-CXC	0.00	-0.08	0.08	1.00
alkB-	A>C	GXC-CXC	0.00	-0.08	0.08	1.00
alkB-	A>C	GXG-CXC	0.02	-0.06	0.10	1.00
alkB-	A>C	GXT-CXC	0.02	-0.06	0.10	1.00
alkB-	A>C	TXA-CXC	0.00	-0.08	0.08	1.00

alkB-	A>C	TXC-CXC	0.00	-0.08	0.08	1.00
alkB-	A>C	TXG-CXC	0.00	-0.08	0.08	1.00
alkB-	A>C	TXT-CXC	0.02	-0.06	0.10	1.00
alkB-	A>C	CXT-CXG	0.02	-0.06	0.10	1.00
alkB-	A>C	GXA-CXG	0.00	-0.08	0.08	1.00
alkB-	A>C	GXC-CXG	0.00	-0.08	0.08	1.00
alkB-	A>C	GXG-CXG	0.02	-0.06	0.10	1.00
alkB-	A>C	GXT-CXG	0.02	-0.06	0.10	1.00
alkB-	A>C	TXA-CXG	0.00	-0.08	0.08	1.00
alkB-	A>C	TXC-CXG	0.00	-0.08	0.08	1.00
alkB-	A>C	TXG-CXG	0.00	-0.08	0.08	1.00
alkB-	A>C	TXT-CXG	0.02	-0.06	0.10	1.00
alkB-	A>C	GXA-CXT	-0.02	-0.10	0.06	1.00
alkB-	A>C	GXC-CXT	-0.02	-0.10	0.06	1.00
alkB-	A>C	GXG-CXT	0.00	-0.08	0.08	1.00
alkB-	A>C	GXT-CXT	0.01	-0.07	0.09	1.00
alkB-	A>C	TXA-CXT	-0.02	-0.10	0.06	1.00
alkB-	A>C	TXC-CXT	-0.02	-0.10	0.06	1.00
alkB-	A>C	TXG-CXT	-0.02	-0.10	0.06	1.00
alkB-	A>C	TXT-CXT	0.00	-0.08	0.08	1.00
alkB-	A>C	GXC-GXA	0.00	-0.08	0.08	1.00
alkB-	A>C	GXG-GXA	0.02	-0.06	0.10	1.00
alkB-	A>C	GXT-GXA	0.02	-0.06	0.10	1.00
alkB-	A>C	TXA-GXA	0.00	-0.08	0.08	1.00
alkB-	A>C	TXC-GXA	0.00	-0.08	0.08	1.00
alkB-	A>C	TXG-GXA	0.00	-0.08	0.08	1.00
alkB-	A>C	TXT-GXA	0.02	-0.06	0.10	1.00
alkB-	A>C	GXG-GXC	0.02	-0.06	0.10	1.00
alkB-	A>C	GXT-GXC	0.02	-0.06	0.10	1.00
alkB-	A>C	TXA-GXC	0.00	-0.08	0.08	1.00
alkB-	A>C	TXC-GXC	0.00	-0.08	0.08	1.00
alkB-	A>C	TXG-GXC	0.00	-0.08	0.08	1.00
alkB-	A>C	TXT-GXC	0.02	-0.06	0.10	1.00
alkB-	A>C	GXT-GXG	0.00	-0.08	0.08	1.00
alkB-	A>C	TXA-GXG	-0.02	-0.10	0.06	1.00
alkB-	A>C	TXC-GXG	-0.02	-0.10	0.06	1.00
alkB-	A>C	TXG-GXG	-0.02	-0.10	0.06	1.00
alkB-	A>C	TXT-GXG	-0.01	-0.09	0.07	1.00
alkB-	A>C	TXA-GXT	-0.02	-0.10	0.06	1.00
alkB-	A>C	TXC-GXT	-0.02	-0.10	0.06	1.00

alkB-	A>C	TXG-GXT	-0.02	-0.10	0.06	1.00
alkB-	A>C	TXT-GXT	-0.01	-0.09	0.07	1.00
alkB-	A>C	TXC-TXA	0.00	-0.08	0.08	1.00
alkB-	A>C	TXG-TXA	0.00	-0.08	0.08	1.00
alkB-	A>C	TXT-TXA	0.02	-0.06	0.10	1.00
alkB-	A>C	TXG-TXC	0.00	-0.08	0.08	1.00
alkB-	A>C	TXT-TXC	0.02	-0.06	0.10	1.00
alkB-	A>C	TXT-TXG	0.02	-0.06	0.10	1.00
WT	A>G	AXC-AXA	-0.01	-0.19	0.16	1.00
WT	A>G	AXG-AXA	0.00	-0.17	0.18	1.00
WT	A>G	AXT-AXA	0.03	-0.15	0.20	1.00
WT	A>G	CXA-AXA	0.06	-0.11	0.24	0.99
WT	A>G	CXC-AXA	0.12	-0.06	0.29	0.53
WT	A>G	CXG-AXA	0.06	-0.12	0.23	1.00
WT	A>G	CXT-AXA	0.06	-0.11	0.24	0.99
WT	A>G	GXA-AXA	-0.01	-0.19	0.16	1.00
WT	A>G	GXC-AXA	-0.01	-0.19	0.16	1.00
WT	A>G	GXG-AXA	0.02	-0.15	0.20	1.00
WT	A>G	GXT-AXA	0.03	-0.15	0.20	1.00
WT	A>G	TXA-AXA	0.04	-0.14	0.21	1.00
WT	A>G	TXC-AXA	0.03	-0.15	0.20	1.00
WT	A>G	TXG-AXA	0.05	-0.13	0.22	1.00
WT	A>G	TXT-AXA	-0.01	-0.19	0.16	1.00
WT	A>G	AXG-AXC	0.02	-0.16	0.19	1.00
WT	A>G	AXT-AXC	0.04	-0.14	0.21	1.00
WT	A>G	CXA-AXC	0.08	-0.10	0.25	0.95
WT	A>G	CXC-AXC	0.13	-0.05	0.30	0.35
WT	A>G	CXG-AXC	0.07	-0.11	0.24	0.98
WT	A>G	CXT-AXC	0.08	-0.10	0.25	0.95
WT	A>G	GXA-AXC	0.00	-0.17	0.17	1.00
WT	A>G	GXC-AXC	0.00	-0.17	0.17	1.00
WT	A>G	GXG-AXC	0.04	-0.14	0.21	1.00
WT	A>G	GXT-AXC	0.04	-0.13	0.22	1.00
WT	A>G	TXA-AXC	0.05	-0.12	0.23	1.00
WT	A>G	TXC-AXC	0.04	-0.14	0.21	1.00
WT	A>G	TXG-AXC	0.06	-0.11	0.24	0.99
WT	A>G	TXT-AXC	0.00	-0.17	0.17	1.00
WT	A>G	AXT-AXG	0.02	-0.15	0.20	1.00
WT	A>G	CXA-AXG	0.06	-0.11	0.24	0.99
WT	A>G	CXC-AXG	0.11	-0.06	0.29	0.56

WT	A>G	CXG-AXG	0.05	-0.12	0.23	1.00
WT	A>G	CXT-AXG	0.06	-0.11	0.23	0.99
WT	A>G	GXA-AXG	-0.02	-0.19	0.16	1.00
WT	A>G	GXC-AXG	-0.02	-0.19	0.16	1.00
WT	A>G	GXG-AXG	0.02	-0.15	0.19	1.00
WT	A>G	GXT-AXG	0.02	-0.15	0.20	1.00
WT	A>G	TXA-AXG	0.04	-0.14	0.21	1.00
WT	A>G	TXC-AXG	0.02	-0.15	0.20	1.00
WT	A>G	TXG-AXG	0.05	-0.13	0.22	1.00
WT	A>G	TXT-AXG	-0.02	-0.19	0.16	1.00
WT	A>G	CXA-AXT	0.04	-0.14	0.21	1.00
WT	A>G	CXC-AXT	0.09	-0.09	0.26	0.86
WT	A>G	CXG-AXT	0.03	-0.14	0.20	1.00
WT	A>G	CXT-AXT	0.04	-0.14	0.21	1.00
WT	A>G	GXA-AXT	-0.04	-0.21	0.14	1.00
WT	A>G	GXC-AXT	-0.04	-0.21	0.14	1.00
WT	A>G	GXG-AXT	0.00	-0.18	0.17	1.00
WT	A>G	GXT-AXT	0.00	-0.17	0.18	1.00
WT	A>G	TXA-AXT	0.01	-0.16	0.19	1.00
WT	A>G	TXC-AXT	0.00	-0.17	0.17	1.00
WT	A>G	TXG-AXT	0.02	-0.15	0.20	1.00
WT	A>G	TXT-AXT	-0.04	-0.21	0.14	1.00
WT	A>G	CXC-CXA	0.05	-0.12	0.23	1.00
WT	A>G	CXG-CXA	-0.01	-0.18	0.17	1.00
WT	A>G	CXT-CXA	0.00	-0.18	0.17	1.00
WT	A>G	GXA-CXA	-0.08	-0.25	0.10	0.95
WT	A>G	GXC-CXA	-0.08	-0.25	0.10	0.95
WT	A>G	GXG-CXA	-0.04	-0.22	0.13	1.00
WT	A>G	GXT-CXA	-0.04	-0.21	0.14	1.00
WT	A>G	TXA-CXA	-0.03	-0.20	0.15	1.00
WT	A>G	TXC-CXA	-0.04	-0.21	0.14	1.00
WT	A>G	TXG-CXA	-0.01	-0.19	0.16	1.00
WT	A>G	TXT-CXA	-0.08	-0.25	0.10	0.95
WT	A>G	CXG-CXC	-0.06	-0.23	0.12	0.99
WT	A>G	CXT-CXC	-0.05	-0.23	0.12	1.00
WT	A>G	GXA-CXC	-0.13	-0.30	0.05	0.35
WT	A>G	GXC-CXC	-0.13	-0.30	0.05	0.35
WT	A>G	GXG-CXC	-0.09	-0.27	0.08	0.82
WT	A>G	GXT-CXC	-0.09	-0.26	0.09	0.87
WT	A>G	TXA-CXC	-0.08	-0.25	0.10	0.95

WT	A>G	TXC-CXC	-0.09	-0.26	0.09	0.86
WT	A>G	TXG-CXC	-0.07	-0.24	0.11	0.99
WT	A>G	TXT-CXC	-0.13	-0.30	0.05	0.35
WT	A>G	CXT-CXG	0.01	-0.17	0.18	1.00
WT	A>G	GXA-CXG	-0.07	-0.24	0.11	0.98
WT	A>G	GXC-CXG	-0.07	-0.24	0.11	0.98
WT	A>G	GXG-CXG	-0.03	-0.21	0.14	1.00
WT	A>G	GXT-CXG	-0.03	-0.20	0.15	1.00
WT	A>G	TXA-CXG	-0.02	-0.19	0.16	1.00
WT	A>G	TXC-CXG	-0.03	-0.20	0.14	1.00
WT	A>G	TXG-CXG	-0.01	-0.18	0.17	1.00
WT	A>G	TXT-CXG	-0.07	-0.24	0.11	0.98
WT	A>G	GXA-CXT	-0.08	-0.25	0.10	0.95
WT	A>G	GXC-CXT	-0.08	-0.25	0.10	0.95
WT	A>G	GXG-CXT	-0.04	-0.21	0.13	1.00
WT	A>G	GXT-CXT	-0.04	-0.21	0.14	1.00
WT	A>G	TXA-CXT	-0.02	-0.20	0.15	1.00
WT	A>G	TXC-CXT	-0.04	-0.21	0.14	1.00
WT	A>G	TXG-CXT	-0.01	-0.19	0.16	1.00
WT	A>G	TXT-CXT	-0.08	-0.25	0.10	0.95
WT	A>G	GXC-GXA	0.00	-0.17	0.17	1.00
WT	A>G	GXG-GXA	0.04	-0.14	0.21	1.00
WT	A>G	GXT-GXA	0.04	-0.13	0.22	1.00
WT	A>G	TXA-GXA	0.05	-0.12	0.23	1.00
WT	A>G	TXC-GXA	0.04	-0.14	0.21	1.00
WT	A>G	TXG-GXA	0.06	-0.11	0.24	0.99
WT	A>G	TXT-GXA	0.00	-0.17	0.17	1.00
WT	A>G	GXG-GXC	0.04	-0.14	0.21	1.00
WT	A>G	GXT-GXC	0.04	-0.13	0.22	1.00
WT	A>G	TXA-GXC	0.05	-0.12	0.23	1.00
WT	A>G	TXC-GXC	0.04	-0.14	0.21	1.00
WT	A>G	TXG-GXC	0.06	-0.11	0.24	0.99
WT	A>G	TXT-GXC	0.00	-0.17	0.17	1.00
WT	A>G	GXT-GXG	0.00	-0.17	0.18	1.00
WT	A>G	TXA-GXG	0.02	-0.16	0.19	1.00
WT	A>G	TXC-GXG	0.00	-0.17	0.18	1.00
WT	A>G	TXG-GXG	0.03	-0.15	0.20	1.00
WT	A>G	TXT-GXG	-0.04	-0.21	0.14	1.00
WT	A>G	TXA-GXT	0.01	-0.16	0.19	1.00
WT	A>G	TXC-GXT	0.00	-0.18	0.17	1.00

WT	A>G	TXG-GXT	0.02	-0.15	0.20	1.00
WT	A>G	TXT-GXT	-0.04	-0.22	0.13	1.00
WT	A>G	TXC-TXA	-0.01	-0.19	0.16	1.00
WT	A>G	TXG-TXA	0.01	-0.16	0.19	1.00
WT	A>G	TXT-TXA	-0.05	-0.23	0.12	1.00
WT	A>G	TXG-TXC	0.02	-0.15	0.20	1.00
WT	A>G	TXT-TXC	-0.04	-0.21	0.14	1.00
WT	A>G	TXT-TXG	-0.06	-0.24	0.11	0.99
alkB-	A>G	AXC-AXA	0.00	-0.15	0.15	1.00
alkB-	A>G	AXG-AXA	-0.03	-0.18	0.12	1.00
alkB-	A>G	AXT-AXA	-0.04	-0.19	0.12	1.00
alkB-	A>G	CXA-AXA	0.00	-0.15	0.15	1.00
alkB-	A>G	CXC-AXA	0.06	-0.10	0.21	0.99
alkB-	A>G	CXG-AXA	0.03	-0.12	0.18	1.00
alkB-	A>G	CXT-AXA	-0.04	-0.19	0.11	1.00
alkB-	A>G	GXA-AXA	0.00	-0.15	0.16	1.00
alkB-	A>G	GXC-AXA	0.01	-0.14	0.16	1.00
alkB-	A>G	GXG-AXA	0.00	-0.15	0.15	1.00
alkB-	A>G	GXT-AXA	-0.04	-0.19	0.11	1.00
alkB-	A>G	TXA-AXA	-0.01	-0.16	0.14	1.00
alkB-	A>G	TXC-AXA	-0.05	-0.20	0.10	0.99
alkB-	A>G	TXG-AXA	0.04	-0.11	0.19	1.00
alkB-	A>G	TXT-AXA	-0.05	-0.20	0.10	0.99
alkB-	A>G	AXG-AXC	-0.03	-0.18	0.12	1.00
alkB-	A>G	AXT-AXC	-0.03	-0.19	0.12	1.00
alkB-	A>G	CXA-AXC	0.00	-0.15	0.15	1.00
alkB-	A>G	CXC-AXC	0.06	-0.09	0.21	0.99
alkB-	A>G	CXG-AXC	0.03	-0.12	0.18	1.00
alkB-	A>G	CXT-AXC	-0.04	-0.19	0.12	1.00
alkB-	A>G	GXA-AXC	0.01	-0.15	0.16	1.00
alkB-	A>G	GXC-AXC	0.01	-0.14	0.16	1.00
alkB-	A>G	GXG-AXC	0.00	-0.15	0.15	1.00
alkB-	A>G	GXT-AXC	-0.04	-0.19	0.11	1.00
alkB-	A>G	TXA-AXC	-0.01	-0.16	0.14	1.00
alkB-	A>G	TXC-AXC	-0.05	-0.20	0.10	0.99
alkB-	A>G	TXG-AXC	0.04	-0.11	0.19	1.00
alkB-	A>G	TXT-AXC	-0.05	-0.20	0.10	0.99
alkB-	A>G	AXT-AXG	-0.01	-0.16	0.14	1.00
alkB-	A>G	CXA-AXG	0.03	-0.12	0.18	1.00
alkB-	A>G	CXC-AXG	0.08	-0.07	0.24	0.76

alkB-	A>G	CXG-AXG	0.06	-0.09	0.21	0.98
alkB-	A>G	CXT-AXG	-0.01	-0.16	0.14	1.00
alkB-	A>G	GXA-AXG	0.03	-0.12	0.19	1.00
alkB-	A>G	GXC-AXG	0.04	-0.11	0.19	1.00
alkB-	A>G	GXG-AXG	0.03	-0.12	0.18	1.00
alkB-	A>G	GXT-AXG	-0.01	-0.16	0.14	1.00
alkB-	A>G	TXA-AXG	0.02	-0.13	0.17	1.00
alkB-	A>G	TXC-AXG	-0.02	-0.17	0.13	1.00
alkB-	A>G	TXG-AXG	0.07	-0.08	0.22	0.94
alkB-	A>G	TXT-AXG	-0.02	-0.17	0.13	1.00
alkB-	A>G	CXA-AXT	0.04	-0.11	0.19	1.00
alkB-	A>G	CXC-AXT	0.09	-0.06	0.24	0.67
alkB-	A>G	CXG-AXT	0.06	-0.09	0.22	0.96
alkB-	A>G	CXT-AXT	0.00	-0.15	0.15	1.00
alkB-	A>G	GXA-AXT	0.04	-0.11	0.19	1.00
alkB-	A>G	GXC-AXT	0.05	-0.11	0.20	1.00
alkB-	A>G	GXG-AXT	0.04	-0.11	0.19	1.00
alkB-	A>G	GXT-AXT	-0.01	-0.16	0.15	1.00
alkB-	A>G	TXA-AXT	0.02	-0.13	0.18	1.00
alkB-	A>G	TXC-AXT	-0.02	-0.17	0.13	1.00
alkB-	A>G	TXG-AXT	0.07	-0.08	0.23	0.89
alkB-	A>G	TXT-AXT	-0.02	-0.17	0.13	1.00
alkB-	A>G	CXC-CXA	0.05	-0.10	0.20	0.99
alkB-	A>G	CXG-CXA	0.03	-0.12	0.18	1.00
alkB-	A>G	CXT-CXA	-0.04	-0.19	0.11	1.00
alkB-	A>G	GXA-CXA	0.00	-0.15	0.15	1.00
alkB-	A>G	GXC-CXA	0.01	-0.14	0.16	1.00
alkB-	A>G	GXG-CXA	0.00	-0.15	0.15	1.00
alkB-	A>G	GXT-CXA	-0.04	-0.19	0.11	1.00
alkB-	A>G	TXA-CXA	-0.01	-0.16	0.14	1.00
alkB-	A>G	TXC-CXA	-0.05	-0.21	0.10	0.99
alkB-	A>G	TXG-CXA	0.04	-0.11	0.19	1.00
alkB-	A>G	TXT-CXA	-0.05	-0.21	0.10	0.99
alkB-	A>G	CXG-CXC	-0.03	-0.18	0.12	1.00
alkB-	A>G	CXT-CXC	-0.09	-0.24	0.06	0.66
alkB-	A>G	GXA-CXC	-0.05	-0.20	0.10	1.00
alkB-	A>G	GXC-CXC	-0.05	-0.20	0.11	1.00
alkB-	A>G	GXG-CXC	-0.05	-0.20	0.10	0.99
alkB-	A>G	GXT-CXC	-0.10	-0.25	0.05	0.58
alkB-	A>G	TXA-CXC	-0.07	-0.22	0.08	0.95

alkB-	A>G	TXC-CXC	-0.11	-0.26	0.04	0.39
alkB-	A>G	TXG-CXC	-0.02	-0.17	0.13	1.00
alkB-	A>G	TXT-CXC	-0.11	-0.26	0.04	0.39
alkB-	A>G	CXT-CXG	-0.07	-0.22	0.09	0.96
alkB-	A>G	GXA-CXG	-0.02	-0.18	0.13	1.00
alkB-	A>G	GXC-CXG	-0.02	-0.17	0.13	1.00
alkB-	A>G	GXG-CXG	-0.03	-0.18	0.12	1.00
alkB-	A>G	GXT-CXG	-0.07	-0.22	0.08	0.92
alkB-	A>G	TXA-CXG	-0.04	-0.19	0.11	1.00
alkB-	A>G	TXC-CXG	-0.08	-0.23	0.07	0.79
alkB-	A>G	TXG-CXG	0.01	-0.14	0.16	1.00
alkB-	A>G	TXT-CXG	-0.08	-0.23	0.07	0.79
alkB-	A>G	GXA-CXT	0.04	-0.11	0.19	1.00
alkB-	A>G	GXC-CXT	0.05	-0.11	0.20	1.00
alkB-	A>G	GXG-CXT	0.04	-0.11	0.19	1.00
alkB-	A>G	GXT-CXT	-0.01	-0.16	0.15	1.00
alkB-	A>G	TXA-CXT	0.02	-0.13	0.18	1.00
alkB-	A>G	TXC-CXT	-0.02	-0.17	0.13	1.00
alkB-	A>G	TXG-CXT	0.07	-0.08	0.23	0.88
alkB-	A>G	TXT-CXT	-0.02	-0.17	0.13	1.00
alkB-	A>G	GXC-GXA	0.00	-0.15	0.16	1.00
alkB-	A>G	GXG-GXA	0.00	-0.15	0.15	1.00
alkB-	A>G	GXT-GXA	-0.05	-0.20	0.10	1.00
alkB-	A>G	TXA-GXA	-0.02	-0.17	0.13	1.00
alkB-	A>G	TXC-GXA	-0.06	-0.21	0.09	0.98
alkB-	A>G	TXG-GXA	0.03	-0.12	0.18	1.00
alkB-	A>G	TXT-GXA	-0.06	-0.21	0.09	0.98
alkB-	A>G	GXG-GXC	-0.01	-0.16	0.14	1.00
alkB-	A>G	GXT-GXC	-0.05	-0.20	0.10	1.00
alkB-	A>G	TXA-GXC	-0.02	-0.17	0.13	1.00
alkB-	A>G	TXC-GXC	-0.06	-0.21	0.09	0.97
alkB-	A>G	TXG-GXC	0.03	-0.12	0.18	1.00
alkB-	A>G	TXT-GXC	-0.06	-0.21	0.09	0.97
alkB-	A>G	GXT-GXG	-0.04	-0.19	0.11	1.00
alkB-	A>G	TXA-GXG	-0.01	-0.16	0.14	1.00
alkB-	A>G	TXC-GXG	-0.06	-0.21	0.10	0.99
alkB-	A>G	TXG-GXG	0.04	-0.11	0.19	1.00
alkB-	A>G	TXT-GXG	-0.06	-0.21	0.10	0.99
alkB-	A>G	TXA-GXT	0.03	-0.12	0.18	1.00
alkB-	A>G	TXC-GXT	-0.01	-0.16	0.14	1.00

alkB-	A>G	TXG-GXT	0.08	-0.07	0.23	0.82
alkB-	A>G	TXT-GXT	-0.01	-0.16	0.14	1.00
alkB-	A>G	TXC-TXA	-0.04	-0.19	0.11	1.00
alkB-	A>G	TXG-TXA	0.05	-0.10	0.20	1.00
alkB-	A>G	TXT-TXA	-0.04	-0.19	0.11	1.00
alkB-	A>G	TXG-TXC	0.09	-0.06	0.24	0.65
alkB-	A>G	TXT-TXC	0.00	-0.15	0.15	1.00
alkB-	A>G	TXT-TXG	-0.09	-0.24	0.06	0.65
WT	A>T	AXC-AXA	0.02	-0.05	0.09	1.00
WT	A>T	AXG-AXA	0.08	0.00	0.15	0.03
WT	A>T	AXT-AXA	0.00	-0.07	0.07	1.00
WT	A>T	CXA-AXA	0.01	-0.06	0.09	1.00
WT	A>T	CXC-AXA	0.02	-0.05	0.09	1.00
WT	A>T	CXG-AXA	0.02	-0.05	0.10	1.00
WT	A>T	CXT-AXA	0.02	-0.06	0.09	1.00
WT	A>T	GXA-AXA	0.00	-0.07	0.07	1.00
WT	A>T	GXC-AXA	0.00	-0.07	0.07	1.00
WT	A>T	GXG-AXA	0.02	-0.06	0.09	1.00
WT	A>T	GXT-AXA	0.00	-0.07	0.07	1.00
WT	A>T	TXA-AXA	0.00	-0.07	0.07	1.00
WT	A>T	TXC-AXA	0.00	-0.07	0.07	1.00
WT	A>T	TXG-AXA	0.03	-0.05	0.10	0.99
WT	A>T	TXT-AXA	0.02	-0.05	0.10	1.00
WT	A>T	AXG-AXC	0.06	-0.02	0.13	0.27
WT	A>T	AXT-AXC	-0.02	-0.09	0.05	1.00
WT	A>T	CXA-AXC	-0.01	-0.08	0.07	1.00
WT	A>T	CXC-AXC	0.00	-0.07	0.07	1.00
WT	A>T	CXG-AXC	0.00	-0.07	0.08	1.00
WT	A>T	CXT-AXC	0.00	-0.08	0.07	1.00
WT	A>T	GXA-AXC	-0.02	-0.09	0.05	1.00
WT	A>T	GXC-AXC	-0.02	-0.09	0.05	1.00
WT	A>T	GXG-AXC	0.00	-0.08	0.07	1.00
WT	A>T	GXT-AXC	-0.02	-0.09	0.05	1.00
WT	A>T	TXA-AXC	-0.02	-0.09	0.05	1.00
WT	A>T	TXC-AXC	-0.02	-0.09	0.05	1.00
WT	A>T	TXG-AXC	0.01	-0.07	0.08	1.00
WT	A>T	TXT-AXC	0.00	-0.07	0.08	1.00
WT	A>T	AXT-AXG	-0.08	-0.15	0.00	0.03
WT	A>T	CXA-AXG	-0.06	-0.14	0.01	0.14
WT	A>T	CXC-AXG	-0.06	-0.13	0.02	0.25

WT	A>T	CXG-AXG	-0.05	-0.13	0.02	0.36
WT	A>T	CXT-AXG	-0.06	-0.13	0.01	0.20
WT	A>T	GXA-AXG	-0.08	-0.15	0.00	0.03
WT	A>T	GXC-AXG	-0.08	-0.15	0.00	0.03
WT	A>T	GXG-AXG	-0.06	-0.13	0.01	0.21
WT	A>T	GXT-AXG	-0.08	-0.15	0.00	0.03
WT	A>T	TXA-AXG	-0.08	-0.15	0.00	0.03
WT	A>T	TXC-AXG	-0.08	-0.15	0.00	0.03
WT	A>T	TXG-AXG	-0.05	-0.13	0.02	0.41
WT	A>T	TXT-AXG	-0.05	-0.13	0.02	0.37
WT	A>T	CXA-AXT	0.01	-0.06	0.09	1.00
WT	A>T	CXC-AXT	0.02	-0.05	0.09	1.00
WT	A>T	CXG-AXT	0.02	-0.05	0.10	1.00
WT	A>T	CXT-AXT	0.02	-0.06	0.09	1.00
WT	A>T	GXA-AXT	0.00	-0.07	0.07	1.00
WT	A>T	GXC-AXT	0.00	-0.07	0.07	1.00
WT	A>T	GXG-AXT	0.02	-0.06	0.09	1.00
WT	A>T	GXT-AXT	0.00	-0.07	0.07	1.00
WT	A>T	TXA-AXT	0.00	-0.07	0.07	1.00
WT	A>T	TXC-AXT	0.00	-0.07	0.07	1.00
WT	A>T	TXG-AXT	0.03	-0.05	0.10	0.99
WT	A>T	TXT-AXT	0.02	-0.05	0.10	1.00
WT	A>T	CXC-CXA	0.01	-0.07	0.08	1.00
WT	A>T	CXG-CXA	0.01	-0.06	0.08	1.00
WT	A>T	CXT-CXA	0.00	-0.07	0.08	1.00
WT	A>T	GXA-CXA	-0.01	-0.09	0.06	1.00
WT	A>T	GXC-CXA	-0.01	-0.09	0.06	1.00
WT	A>T	GXG-CXA	0.00	-0.07	0.08	1.00
WT	A>T	GXT-CXA	-0.01	-0.09	0.06	1.00
WT	A>T	TXA-CXA	-0.01	-0.09	0.06	1.00
WT	A>T	TXC-CXA	-0.01	-0.09	0.06	1.00
WT	A>T	TXG-CXA	0.01	-0.06	0.09	1.00
WT	A>T	TXT-CXA	0.01	-0.06	0.08	1.00
WT	A>T	CXG-CXC	0.00	-0.07	0.08	1.00
WT	A>T	CXT-CXC	0.00	-0.08	0.07	1.00
WT	A>T	GXA-CXC	-0.02	-0.09	0.05	1.00
WT	A>T	GXC-CXC	-0.02	-0.09	0.05	1.00
WT	A>T	GXG-CXC	0.00	-0.08	0.07	1.00
WT	A>T	GXT-CXC	-0.02	-0.09	0.05	1.00
WT	A>T	TXA-CXC	-0.02	-0.09	0.05	1.00

WT	A>T	TXC-CXC	-0.02	-0.09	0.05	1.00
WT	A>T	TXG-CXC	0.01	-0.07	0.08	1.00
WT	A>T	TXT-CXC	0.00	-0.07	0.08	1.00
WT	A>T	CXT-CXG	-0.01	-0.08	0.07	1.00
WT	A>T	GXA-CXG	-0.02	-0.10	0.05	1.00
WT	A>T	GXC-CXG	-0.02	-0.10	0.05	1.00
WT	A>T	GXG-CXG	-0.01	-0.08	0.07	1.00
WT	A>T	GXT-CXG	-0.02	-0.10	0.05	1.00
WT	A>T	TXA-CXG	-0.02	-0.10	0.05	1.00
WT	A>T	TXC-CXG	-0.02	-0.10	0.05	1.00
WT	A>T	TXG-CXG	0.00	-0.07	0.07	1.00
WT	A>T	TXT-CXG	0.00	-0.07	0.07	1.00
WT	A>T	GXA-CXT	-0.02	-0.09	0.06	1.00
WT	A>T	GXC-CXT	-0.02	-0.09	0.06	1.00
WT	A>T	GXG-CXT	0.00	-0.07	0.07	1.00
WT	A>T	GXT-CXT	-0.02	-0.09	0.06	1.00
WT	A>T	TXA-CXT	-0.02	-0.09	0.06	1.00
WT	A>T	TXC-CXT	-0.02	-0.09	0.06	1.00
WT	A>T	TXG-CXT	0.01	-0.06	0.08	1.00
WT	A>T	TXT-CXT	0.01	-0.07	0.08	1.00
WT	A>T	GXC-GXA	0.00	-0.07	0.07	1.00
WT	A>T	GXG-GXA	0.02	-0.06	0.09	1.00
WT	A>T	GXT-GXA	0.00	-0.07	0.07	1.00
WT	A>T	TXA-GXA	0.00	-0.07	0.07	1.00
WT	A>T	TXC-GXA	0.00	-0.07	0.07	1.00
WT	A>T	TXG-GXA	0.03	-0.05	0.10	0.99
WT	A>T	TXT-GXA	0.02	-0.05	0.10	1.00
WT	A>T	GXG-GXC	0.02	-0.06	0.09	1.00
WT	A>T	GXT-GXC	0.00	-0.07	0.07	1.00
WT	A>T	TXA-GXC	0.00	-0.07	0.07	1.00
WT	A>T	TXC-GXC	0.00	-0.07	0.07	1.00
WT	A>T	TXG-GXC	0.03	-0.05	0.10	0.99
WT	A>T	TXT-GXC	0.02	-0.05	0.10	1.00
WT	A>T	GXT-GXG	-0.02	-0.09	0.06	1.00
WT	A>T	TXA-GXG	-0.02	-0.09	0.06	1.00
WT	A>T	TXC-GXG	-0.02	-0.09	0.06	1.00
WT	A>T	TXG-GXG	0.01	-0.07	0.08	1.00
WT	A>T	TXT-GXG	0.01	-0.07	0.08	1.00
WT	A>T	TXA-GXT	0.00	-0.07	0.07	1.00
WT	A>T	TXC-GXT	0.00	-0.07	0.07	1.00

WT	A>T	TXG-GXT	0.03	-0.05	0.10	0.99
WT	A>T	TXT-GXT	0.02	-0.05	0.10	1.00
WT	A>T	TXC-TXA	0.00	-0.07	0.07	1.00
WT	A>T	TXG-TXA	0.03	-0.05	0.10	0.99
WT	A>T	TXT-TXA	0.02	-0.05	0.10	1.00
WT	A>T	TXG-TXC	0.03	-0.05	0.10	0.99
WT	A>T	TXT-TXC	0.02	-0.05	0.10	1.00
WT	A>T	TXT-TXG	0.00	-0.07	0.07	1.00
alkB-	A>T	AXC-AXA	0.04	-0.06	0.13	0.98
alkB-	A>T	AXG-AXA	0.00	-0.10	0.09	1.00
alkB-	A>T	AXT-AXA	-0.02	-0.11	0.08	1.00
alkB-	A>T	CXA-AXA	-0.02	-0.11	0.08	1.00
alkB-	A>T	CXC-AXA	-0.02	-0.11	0.08	1.00
alkB-	A>T	CXG-AXA	0.01	-0.08	0.11	1.00
alkB-	A>T	CXT-AXA	-0.02	-0.11	0.08	1.00
alkB-	A>T	GXA-AXA	-0.02	-0.11	0.08	1.00
alkB-	A>T	GXC-AXA	0.01	-0.08	0.11	1.00
alkB-	A>T	GXG-AXA	0.00	-0.10	0.09	1.00
alkB-	A>T	GXT-AXA	0.00	-0.10	0.09	1.00
alkB-	A>T	TXA-AXA	-0.02	-0.11	0.08	1.00
alkB-	A>T	TXC-AXA	-0.02	-0.11	0.08	1.00
alkB-	A>T	TXG-AXA	0.00	-0.10	0.09	1.00
alkB-	A>T	TXT-AXA	-0.02	-0.11	0.08	1.00
alkB-	A>T	AXG-AXC	-0.04	-0.13	0.05	0.96
alkB-	A>T	AXT-AXC	-0.05	-0.15	0.04	0.77
alkB-	A>T	CXA-AXC	-0.05	-0.15	0.04	0.77
alkB-	A>T	CXC-AXC	-0.05	-0.15	0.04	0.77
alkB-	A>T	CXG-AXC	-0.02	-0.12	0.07	1.00
alkB-	A>T	CXT-AXC	-0.05	-0.15	0.04	0.77
alkB-	A>T	GXA-AXC	-0.05	-0.15	0.04	0.77
alkB-	A>T	GXC-AXC	-0.02	-0.12	0.07	1.00
alkB-	A>T	GXG-AXC	-0.04	-0.13	0.05	0.97
alkB-	A>T	GXT-AXC	-0.04	-0.13	0.05	0.96
alkB-	A>T	TXA-AXC	-0.05	-0.15	0.04	0.77
alkB-	A>T	TXC-AXC	-0.05	-0.15	0.04	0.77
alkB-	A>T	TXG-AXC	-0.04	-0.13	0.05	0.96
alkB-	A>T	TXT-AXC	-0.05	-0.15	0.04	0.77
alkB-	A>T	AXT-AXG	-0.01	-0.11	0.08	1.00
alkB-	A>T	CXA-AXG	-0.01	-0.11	0.08	1.00
alkB-	A>T	CXC-AXG	-0.01	-0.11	0.08	1.00

alkB-	A>T	CXG-AXG	0.02	-0.08	0.11	1.00
alkB-	A>T	CXT-AXG	-0.01	-0.11	0.08	1.00
alkB-	A>T	GXA-AXG	-0.01	-0.11	0.08	1.00
alkB-	A>T	GXC-AXG	0.02	-0.08	0.11	1.00
alkB-	A>T	GXG-AXG	0.00	-0.09	0.09	1.00
alkB-	A>T	GXT-AXG	0.00	-0.09	0.09	1.00
alkB-	A>T	TXA-AXG	-0.01	-0.11	0.08	1.00
alkB-	A>T	TXC-AXG	-0.01	-0.11	0.08	1.00
alkB-	A>T	TXG-AXG	0.00	-0.09	0.09	1.00
alkB-	A>T	TXT-AXG	-0.01	-0.11	0.08	1.00
alkB-	A>T	CXA-AXT	0.00	-0.09	0.09	1.00
alkB-	A>T	CXC-AXT	0.00	-0.09	0.09	1.00
alkB-	A>T	CXG-AXT	0.03	-0.07	0.12	1.00
alkB-	A>T	CXT-AXT	0.00	-0.09	0.09	1.00
alkB-	A>T	GXA-AXT	0.00	-0.09	0.09	1.00
alkB-	A>T	GXC-AXT	0.03	-0.07	0.12	1.00
alkB-	A>T	GXG-AXT	0.01	-0.08	0.11	1.00
alkB-	A>T	GXT-AXT	0.01	-0.08	0.11	1.00
alkB-	A>T	TXA-AXT	0.00	-0.09	0.09	1.00
alkB-	A>T	TXC-AXT	0.00	-0.09	0.09	1.00
alkB-	A>T	TXG-AXT	0.01	-0.08	0.11	1.00
alkB-	A>T	TXT-AXT	0.00	-0.09	0.09	1.00
alkB-	A>T	CXC-CXA	0.00	-0.09	0.09	1.00
alkB-	A>T	CXG-CXA	0.03	-0.07	0.12	1.00
alkB-	A>T	CXT-CXA	0.00	-0.09	0.09	1.00
alkB-	A>T	GXA-CXA	0.00	-0.09	0.09	1.00
alkB-	A>T	GXC-CXA	0.03	-0.07	0.12	1.00
alkB-	A>T	GXG-CXA	0.01	-0.08	0.11	1.00
alkB-	A>T	GXT-CXA	0.01	-0.08	0.11	1.00
alkB-	A>T	TXA-CXA	0.00	-0.09	0.09	1.00
alkB-	A>T	TXC-CXA	0.00	-0.09	0.09	1.00
alkB-	A>T	TXG-CXA	0.01	-0.08	0.11	1.00
alkB-	A>T	TXT-CXA	0.00	-0.09	0.09	1.00
alkB-	A>T	CXG-CXC	0.03	-0.07	0.12	1.00
alkB-	A>T	CXT-CXC	0.00	-0.09	0.09	1.00
alkB-	A>T	GXA-CXC	0.00	-0.09	0.09	1.00
alkB-	A>T	GXC-CXC	0.03	-0.07	0.12	1.00
alkB-	A>T	GXG-CXC	0.01	-0.08	0.11	1.00
alkB-	A>T	GXT-CXC	0.01	-0.08	0.11	1.00
alkB-	A>T	TXA-CXC	0.00	-0.09	0.09	1.00

alkB-	A>T	TXC-CXC	0.00	-0.09	0.09	1.00
alkB-	A>T	TXG-CXC	0.01	-0.08	0.11	1.00
alkB-	A>T	TXT-CXC	0.00	-0.09	0.09	1.00
alkB-	A>T	CXT-CXG	-0.03	-0.12	0.07	1.00
alkB-	A>T	GXA-CXG	-0.03	-0.12	0.07	1.00
alkB-	A>T	GXC-CXG	0.00	-0.09	0.09	1.00
alkB-	A>T	GXG-CXG	-0.02	-0.11	0.08	1.00
alkB-	A>T	GXT-CXG	-0.02	-0.11	0.08	1.00
alkB-	A>T	TXA-CXG	-0.03	-0.12	0.07	1.00
alkB-	A>T	TXC-CXG	-0.03	-0.12	0.07	1.00
alkB-	A>T	TXG-CXG	-0.02	-0.11	0.08	1.00
alkB-	A>T	TXT-CXG	-0.03	-0.12	0.07	1.00
alkB-	A>T	GXA-CXT	0.00	-0.09	0.09	1.00
alkB-	A>T	GXC-CXT	0.03	-0.07	0.12	1.00
alkB-	A>T	GXG-CXT	0.01	-0.08	0.11	1.00
alkB-	A>T	GXT-CXT	0.01	-0.08	0.11	1.00
alkB-	A>T	TXA-CXT	0.00	-0.09	0.09	1.00
alkB-	A>T	TXC-CXT	0.00	-0.09	0.09	1.00
alkB-	A>T	TXG-CXT	0.01	-0.08	0.11	1.00
alkB-	A>T	TXT-CXT	0.00	-0.09	0.09	1.00
alkB-	A>T	GXC-GXA	0.03	-0.07	0.12	1.00
alkB-	A>T	GXG-GXA	0.01	-0.08	0.11	1.00
alkB-	A>T	GXT-GXA	0.01	-0.08	0.11	1.00
alkB-	A>T	TXA-GXA	0.00	-0.09	0.09	1.00
alkB-	A>T	TXC-GXA	0.00	-0.09	0.09	1.00
alkB-	A>T	TXG-GXA	0.01	-0.08	0.11	1.00
alkB-	A>T	TXT-GXA	0.00	-0.09	0.09	1.00
alkB-	A>T	GXG-GXC	-0.02	-0.11	0.08	1.00
alkB-	A>T	GXT-GXC	-0.02	-0.11	0.08	1.00
alkB-	A>T	TXA-GXC	-0.03	-0.12	0.07	1.00
alkB-	A>T	TXC-GXC	-0.03	-0.12	0.07	1.00
alkB-	A>T	TXG-GXC	-0.02	-0.11	0.08	1.00
alkB-	A>T	TXT-GXC	-0.03	-0.12	0.07	1.00
alkB-	A>T	GXT-GXG	0.00	-0.10	0.09	1.00
alkB-	A>T	TXA-GXG	-0.01	-0.11	0.08	1.00
alkB-	A>T	TXC-GXG	-0.01	-0.11	0.08	1.00
alkB-	A>T	TXG-GXG	0.00	-0.09	0.09	1.00
alkB-	A>T	TXT-GXG	-0.01	-0.11	0.08	1.00
alkB-	A>T	TXA-GXT	-0.01	-0.11	0.08	1.00
alkB-	A>T	TXC-GXT	-0.01	-0.11	0.08	1.00

alkB-	A>T	TXG-GXT	0.00	-0.09	0.10	1.00
alkB-	A>T	TXT-GXT	-0.01	-0.11	0.08	1.00
alkB-	A>T	TXC-TXA	0.00	-0.09	0.09	1.00
alkB-	A>T	TXG-TXA	0.01	-0.08	0.11	1.00
alkB-	A>T	TXT-TXA	0.00	-0.09	0.09	1.00
alkB-	A>T	TXG-TXC	0.01	-0.08	0.11	1.00
alkB-	A>T	TXT-TXC	0.00	-0.09	0.09	1.00
alkB-	A>T	TXT-TXG	-0.01	-0.11	0.08	1.00
WT	X>C	AXC-AXA	0.25	-0.83	1.33	1.00
WT	X>C	AXG-AXA	0.44	-0.64	1.52	0.97
WT	X>C	AXT-AXA	0.63	-0.45	1.71	0.72
WT	X>C	CXA-AXA	-0.01	-1.09	1.07	1.00
WT	X>C	CXC-AXA	-0.05	-1.13	1.03	1.00
WT	X>C	CXG-AXA	0.34	-0.74	1.42	1.00
WT	X>C	CXT-AXA	0.23	-0.85	1.30	1.00
WT	X>C	GXA-AXA	0.41	-0.67	1.48	0.99
WT	X>C	GXC-AXA	1.19	0.11	2.27	0.02
WT	X>C	GXG-AXA	1.46	0.38	2.53	0.00
WT	X>C	GXT-AXA	1.49	0.42	2.57	0.00
WT	X>C	TXA-AXA	-0.05	-1.12	1.03	1.00
WT	X>C	TXC-AXA	0.07	-1.01	1.15	1.00
WT	X>C	TXG-AXA	0.07	-1.01	1.14	1.00
WT	X>C	TXT-AXA	0.43	-0.65	1.51	0.98
WT	X>C	AXG-AXC	0.19	-0.89	1.27	1.00
WT	X>C	AXT-AXC	0.38	-0.70	1.46	0.99
WT	X>C	CXA-AXC	-0.26	-1.33	0.82	1.00
WT	X>C	CXC-AXC	-0.30	-1.38	0.78	1.00
WT	X>C	CXG-AXC	0.09	-0.99	1.17	1.00
WT	X>C	CXT-AXC	-0.02	-1.10	1.06	1.00
WT	X>C	GXA-AXC	0.16	-0.92	1.24	1.00
WT	X>C	GXC-AXC	0.94	-0.14	2.02	0.14
WT	X>C	GXG-AXC	1.21	0.13	2.29	0.02
WT	X>C	GXT-AXC	1.25	0.17	2.32	0.01
WT	X>C	TXA-AXC	-0.29	-1.37	0.78	1.00
WT	X>C	TXC-AXC	-0.18	-1.25	0.90	1.00
WT	X>C	TXG-AXC	-0.18	-1.26	0.90	1.00
WT	X>C	TXT-AXC	0.18	-0.90	1.26	1.00
WT	X>C	AXT-AXG	0.19	-0.89	1.27	1.00
WT	X>C	CXA-AXG	-0.45	-1.53	0.63	0.97
WT	X>C	CXC-AXG	-0.49	-1.57	0.59	0.93

WT	X>C	CXG-AXG	-0.10	-1.18	0.98	1.00
WT	X>C	CXT-AXG	-0.21	-1.29	0.86	1.00
WT	X>C	GXA-AXG	-0.03	-1.11	1.04	1.00
WT	X>C	GXC-AXG	0.75	-0.33	1.83	0.44
WT	X>C	GXG-AXG	1.02	-0.06	2.09	0.08
WT	X>C	GXT-AXG	1.05	-0.02	2.13	0.06
WT	X>C	TXA-AXG	-0.48	-1.56	0.59	0.94
WT	X>C	TXC-AXG	-0.37	-1.45	0.71	0.99
WT	X>C	TXG-AXG	-0.37	-1.45	0.70	0.99
WT	X>C	TXT-AXG	-0.01	-1.09	1.07	1.00
WT	X>C	CXA-AXT	-0.64	-1.71	0.44	0.70
WT	X>C	CXC-AXT	-0.68	-1.76	0.40	0.60
WT	X>C	CXG-AXT	-0.29	-1.37	0.79	1.00
WT	X>C	CXT-AXT	-0.40	-1.48	0.68	0.99
WT	X>C	GXA-AXT	-0.22	-1.30	0.86	1.00
WT	X>C	GXC-AXT	0.56	-0.52	1.64	0.84
WT	X>C	GXG-AXT	0.83	-0.25	1.91	0.29
WT	X>C	GXT-AXT	0.87	-0.21	1.94	0.23
WT	X>C	TXA-AXT	-0.67	-1.75	0.41	0.62
WT	X>C	TXC-AXT	-0.56	-1.63	0.52	0.85
WT	X>C	TXG-AXT	-0.56	-1.64	0.52	0.84
WT	X>C	TXT-AXT	-0.20	-1.28	0.88	1.00
WT	X>C	CXC-CXA	-0.04	-1.12	1.03	1.00
WT	X>C	CXG-CXA	0.35	-0.73	1.42	1.00
WT	X>C	CXT-CXA	0.23	-0.84	1.31	1.00
WT	X>C	GXA-CXA	0.41	-0.66	1.49	0.98
WT	X>C	GXC-CXA	1.20	0.12	2.28	0.02
WT	X>C	GXG-CXA	1.46	0.39	2.54	0.00
WT	X>C	GXT-CXA	1.50	0.42	2.58	0.00
WT	X>C	TXA-CXA	-0.04	-1.12	1.04	1.00
WT	X>C	TXC-CXA	0.08	-1.00	1.16	1.00
WT	X>C	TXG-CXA	0.07	-1.00	1.15	1.00
WT	X>C	TXT-CXA	0.44	-0.64	1.51	0.97
WT	X>C	CXG-CXC	0.39	-0.69	1.47	0.99
WT	X>C	CXT-CXC	0.28	-0.80	1.36	1.00
WT	X>C	GXA-CXC	0.46	-0.62	1.54	0.96
WT	X>C	GXC-CXC	1.24	0.17	2.32	0.01
WT	X>C	GXG-CXC	1.51	0.43	2.59	0.00
WT	X>C	GXT-CXC	1.55	0.47	2.62	0.00
WT	X>C	TXA-CXC	0.01	-1.07	1.09	1.00

WT	X>C	TXC-CXC	0.12	-0.95	1.20	1.00
WT	X>C	TXG-CXC	0.12	-0.96	1.20	1.00
WT	X>C	TXT-CXC	0.48	-0.60	1.56	0.94
WT	X>C	CXT-CXG	-0.11	-1.19	0.97	1.00
WT	X>C	GXA-CXG	0.07	-1.01	1.15	1.00
WT	X>C	GXC-CXG	0.85	-0.23	1.93	0.25
WT	X>C	GXG-CXG	1.12	0.04	2.20	0.04
WT	X>C	GXT-CXG	1.16	0.08	2.23	0.03
WT	X>C	TXA-CXG	-0.38	-1.46	0.70	0.99
WT	X>C	TXC-CXG	-0.27	-1.34	0.81	1.00
WT	X>C	TXG-CXG	-0.27	-1.35	0.81	1.00
WT	X>C	TXT-CXG	0.09	-0.99	1.17	1.00
WT	X>C	GXA-CXT	0.18	-0.90	1.26	1.00
WT	X>C	GXC-CXT	0.97	-0.11	2.04	0.12
WT	X>C	GXG-CXT	1.23	0.15	2.31	0.01
WT	X>C	GXT-CXT	1.27	0.19	2.35	0.01
WT	X>C	TXA-CXT	-0.27	-1.35	0.81	1.00
WT	X>C	TXC-CXT	-0.15	-1.23	0.92	1.00
WT	X>C	TXG-CXT	-0.16	-1.24	0.92	1.00
WT	X>C	TXT-CXT	0.20	-0.88	1.28	1.00
WT	X>C	GXC-GXA	0.79	-0.29	1.86	0.37
WT	X>C	GXG-GXA	1.05	-0.03	2.13	0.06
WT	X>C	GXT-GXA	1.09	0.01	2.17	0.05
WT	X>C	TXA-GXA	-0.45	-1.53	0.63	0.97
WT	X>C	TXC-GXA	-0.33	-1.41	0.74	1.00
WT	X>C	TXG-GXA	-0.34	-1.42	0.74	1.00
WT	X>C	TXT-GXA	0.02	-1.06	1.10	1.00
WT	X>C	GXG-GXC	0.27	-0.81	1.34	1.00
WT	X>C	GXT-GXC	0.30	-0.78	1.38	1.00
WT	X>C	TXA-GXC	-1.24	-2.31	-0.16	0.01
WT	X>C	TXC-GXC	-1.12	-2.20	-0.04	0.04
WT	X>C	TXG-GXC	-1.13	-2.20	-0.05	0.03
WT	X>C	TXT-GXC	-0.76	-1.84	0.32	0.42
WT	X>C	GXT-GXG	0.04	-1.04	1.12	1.00
WT	X>C	TXA-GXG	-1.50	-2.58	-0.42	0.00
WT	X>C	TXC-GXG	-1.38	-2.46	-0.31	0.00
WT	X>C	TXG-GXG	-1.39	-2.47	-0.31	0.00
WT	X>C	TXT-GXG	-1.03	-2.11	0.05	0.07
WT	X>C	TXA-GXT	-1.54	-2.62	-0.46	0.00
WT	X>C	TXC-GXT	-1.42	-2.50	-0.34	0.00

WT	X>C	TXG-GXT	-1.43	-2.51	-0.35	0.00
WT	X>C	TXT-GXT	-1.07	-2.14	0.01	0.06
WT	X>C	TXC-TXA	0.12	-0.96	1.19	1.00
WT	X>C	TXG-TXA	0.11	-0.97	1.19	1.00
WT	X>C	TXT-TXA	0.47	-0.60	1.55	0.95
WT	X>C	TXG-TXC	-0.01	-1.08	1.07	1.00
WT	X>C	TXT-TXC	0.36	-0.72	1.43	1.00
WT	X>C	TXT-TXG	0.36	-0.72	1.44	1.00
alkB-	X>C	AXC-AXA	1.00	0.04	1.97	0.04
alkB-	X>C	AXG-AXA	0.50	-0.47	1.46	0.85
alkB-	X>C	AXT-AXA	0.21	-0.76	1.17	1.00
alkB-	X>C	CXA-AXA	-0.10	-1.06	0.87	1.00
alkB-	X>C	CXC-AXA	0.10	-0.87	1.06	1.00
alkB-	X>C	CXG-AXA	0.25	-0.72	1.22	1.00
alkB-	X>C	CXT-AXA	0.22	-0.74	1.19	1.00
alkB-	X>C	GXA-AXA	0.29	-0.67	1.26	1.00
alkB-	X>C	GXC-AXA	1.57	0.60	2.53	0.00
alkB-	X>C	GXG-AXA	1.54	0.58	2.51	0.00
alkB-	X>C	GXT-AXA	1.52	0.55	2.48	0.00
alkB-	X>C	TXA-AXA	0.40	-0.56	1.37	0.97
alkB-	X>C	TXC-AXA	0.00	-0.97	0.96	1.00
alkB-	X>C	TXG-AXA	0.34	-0.62	1.31	0.99
alkB-	X>C	TXT-AXA	0.29	-0.68	1.25	1.00
alkB-	X>C	AXG-AXC	-0.50	-1.47	0.46	0.84
alkB-	X>C	AXT-AXC	-0.79	-1.76	0.17	0.21
alkB-	X>C	CXA-AXC	-1.10	-2.06	-0.13	0.01
alkB-	X>C	CXC-AXC	-0.90	-1.87	0.06	0.09
alkB-	X>C	CXG-AXC	-0.75	-1.72	0.21	0.28
alkB-	X>C	CXT-AXC	-0.78	-1.74	0.19	0.23
alkB-	X>C	GXA-AXC	-0.71	-1.67	0.26	0.37
alkB-	X>C	GXC-AXC	0.56	-0.40	1.53	0.71
alkB-	X>C	GXG-AXC	0.54	-0.42	1.51	0.76
alkB-	X>C	GXT-AXC	0.52	-0.45	1.48	0.81
alkB-	X>C	TXA-AXC	-0.60	-1.56	0.37	0.63
alkB-	X>C	TXC-AXC	-1.00	-1.97	-0.04	0.04
alkB-	X>C	TXG-AXC	-0.66	-1.63	0.31	0.47
alkB-	X>C	TXT-AXC	-0.71	-1.68	0.25	0.35
alkB-	X>C	AXT-AXG	-0.29	-1.25	0.68	1.00
alkB-	X>C	CXA-AXG	-0.59	-1.56	0.37	0.64
alkB-	X>C	CXC-AXG	-0.40	-1.37	0.57	0.97

alkB-	X>C	CXG-AXG	-0.25	-1.21	0.72	1.00
alkB-	X>C	CXT-AXG	-0.27	-1.24	0.69	1.00
alkB-	X>C	GXA-AXG	-0.20	-1.17	0.76	1.00
alkB-	X>C	GXC-AXG	1.07	0.10	2.03	0.02
alkB-	X>C	GXG-AXG	1.05	0.08	2.01	0.02
alkB-	X>C	GXT-AXG	1.02	0.06	1.99	0.03
alkB-	X>C	TXA-AXG	-0.09	-1.06	0.87	1.00
alkB-	X>C	TXC-AXG	-0.50	-1.46	0.47	0.85
alkB-	X>C	TXG-AXG	-0.16	-1.12	0.81	1.00
alkB-	X>C	TXT-AXG	-0.21	-1.18	0.76	1.00
alkB-	X>C	CXA-AXT	-0.31	-1.27	0.66	1.00
alkB-	X>C	CXC-AXT	-0.11	-1.08	0.85	1.00
alkB-	X>C	CXG-AXT	0.04	-0.92	1.01	1.00
alkB-	X>C	CXT-AXT	0.02	-0.95	0.98	1.00
alkB-	X>C	GXA-AXT	0.09	-0.88	1.05	1.00
alkB-	X>C	GXC-AXT	1.36	0.39	2.32	0.00
alkB-	X>C	GXG-AXT	1.34	0.37	2.30	0.00
alkB-	X>C	GXT-AXT	1.31	0.35	2.28	0.00
alkB-	X>C	TXA-AXT	0.20	-0.77	1.16	1.00
alkB-	X>C	TXC-AXT	-0.21	-1.18	0.75	1.00
alkB-	X>C	TXG-AXT	0.13	-0.83	1.10	1.00
alkB-	X>C	TXT-AXT	0.08	-0.89	1.04	1.00
alkB-	X>C	CXC-CXA	0.19	-0.77	1.16	1.00
alkB-	X>C	CXG-CXA	0.35	-0.62	1.31	0.99
alkB-	X>C	CXT-CXA	0.32	-0.64	1.29	1.00
alkB-	X>C	GXA-CXA	0.39	-0.57	1.36	0.97
alkB-	X>C	GXC-CXA	1.66	0.70	2.63	0.00
alkB-	X>C	GXG-CXA	1.64	0.68	2.61	0.00
alkB-	X>C	GXT-CXA	1.62	0.65	2.58	0.00
alkB-	X>C	TXA-CXA	0.50	-0.46	1.47	0.84
alkB-	X>C	TXC-CXA	0.10	-0.87	1.06	1.00
alkB-	X>C	TXG-CXA	0.44	-0.53	1.41	0.93
alkB-	X>C	TXT-CXA	0.39	-0.58	1.35	0.98
alkB-	X>C	CXG-CXC	0.15	-0.81	1.12	1.00
alkB-	X>C	CXT-CXC	0.13	-0.84	1.09	1.00
alkB-	X>C	GXA-CXC	0.20	-0.77	1.16	1.00
alkB-	X>C	GXC-CXC	1.47	0.50	2.43	0.00
alkB-	X>C	GXG-CXC	1.45	0.48	2.41	0.00
alkB-	X>C	GXT-CXC	1.42	0.46	2.39	0.00
alkB-	X>C	TXA-CXC	0.31	-0.66	1.27	1.00

alkB-	X>C	TXC-CXC	-0.10	-1.06	0.87	1.00
alkB-	X>C	TXG-CXC	0.24	-0.72	1.21	1.00
alkB-	X>C	TXT-CXC	0.19	-0.78	1.16	1.00
alkB-	X>C	CXT-CXG	-0.03	-0.99	0.94	1.00
alkB-	X>C	GXA-CXG	0.04	-0.92	1.01	1.00
alkB-	X>C	GXC-CXG	1.32	0.35	2.28	0.00
alkB-	X>C	GXG-CXG	1.29	0.33	2.26	0.00
alkB-	X>C	GXT-CXG	1.27	0.30	2.24	0.00
alkB-	X>C	TXA-CXG	0.16	-0.81	1.12	1.00
alkB-	X>C	TXC-CXG	-0.25	-1.22	0.71	1.00
alkB-	X>C	TXG-CXG	0.09	-0.87	1.06	1.00
alkB-	X>C	TXT-CXG	0.04	-0.93	1.00	1.00
alkB-	X>C	GXA-CXT	0.07	-0.89	1.04	1.00
alkB-	X>C	GXC-CXT	1.34	0.38	2.31	0.00
alkB-	X>C	GXG-CXT	1.32	0.36	2.29	0.00
alkB-	X>C	GXT-CXT	1.30	0.33	2.26	0.00
alkB-	X>C	TXA-CXT	0.18	-0.78	1.15	1.00
alkB-	X>C	TXC-CXT	-0.23	-1.19	0.74	1.00
alkB-	X>C	TXG-CXT	0.12	-0.85	1.08	1.00
alkB-	X>C	TXT-CXT	0.06	-0.90	1.03	1.00
alkB-	X>C	GXC-GXA	1.27	0.31	2.24	0.00
alkB-	X>C	GXG-GXA	1.25	0.28	2.22	0.00
alkB-	X>C	GXT-GXA	1.23	0.26	2.19	0.00
alkB-	X>C	TXA-GXA	0.11	-0.85	1.08	1.00
alkB-	X>C	TXC-GXA	-0.30	-1.26	0.67	1.00
alkB-	X>C	TXG-GXA	0.05	-0.92	1.01	1.00
alkB-	X>C	TXT-GXA	-0.01	-0.97	0.96	1.00
alkB-	X>C	GXG-GXC	-0.02	-0.99	0.94	1.00
alkB-	X>C	GXT-GXC	-0.05	-1.01	0.92	1.00
alkB-	X>C	TXA-GXC	-1.16	-2.13	-0.19	0.01
alkB-	X>C	TXC-GXC	-1.57	-2.53	-0.60	0.00
alkB-	X>C	TXG-GXC	-1.22	-2.19	-0.26	0.00
alkB-	X>C	TXT-GXC	-1.28	-2.24	-0.31	0.00
alkB-	X>C	GXT-GXG	-0.02	-0.99	0.94	1.00
alkB-	X>C	TXA-GXG	-1.14	-2.10	-0.17	0.01
alkB-	X>C	TXC-GXG	-1.55	-2.51	-0.58	0.00
alkB-	X>C	TXG-GXG	-1.20	-2.17	-0.24	0.00
alkB-	X>C	TXT-GXG	-1.26	-2.22	-0.29	0.00
alkB-	X>C	TXA-GXT	-1.11	-2.08	-0.15	0.01
alkB-	X>C	TXC-GXT	-1.52	-2.49	-0.56	0.00

alkB-	X>C	TXG-GXT	-1.18	-2.14	-0.21	0.01
alkB-	X>C	TXT-GXT	-1.23	-2.20	-0.27	0.00
alkB-	X>C	TXC-TXA	-0.41	-1.37	0.56	0.96
alkB-	X>C	TXG-TXA	-0.06	-1.03	0.90	1.00
alkB-	X>C	TXT-TXA	-0.12	-1.08	0.85	1.00
alkB-	X>C	TXG-TXC	0.34	-0.62	1.31	0.99
alkB-	X>C	TXT-TXC	0.29	-0.68	1.26	1.00
alkB-	X>C	TXT-TXG	-0.05	-1.02	0.91	1.00
WT	X>G	AXC-AXA	0.00	-0.08	0.08	1.00
WT	X>G	AXG-AXA	-0.01	-0.09	0.07	1.00
WT	X>G	AXT-AXA	-0.01	-0.08	0.07	1.00
WT	X>G	CXA-AXA	-0.02	-0.10	0.06	1.00
WT	X>G	CXC-AXA	-0.02	-0.10	0.06	1.00
WT	X>G	CXG-AXA	-0.02	-0.10	0.06	1.00
WT	X>G	CXT-AXA	-0.02	-0.10	0.06	1.00
WT	X>G	GXA-AXA	-0.02	-0.10	0.06	1.00
WT	X>G	GXC-AXA	-0.01	-0.08	0.07	1.00
WT	X>G	GXG-AXA	-0.01	-0.09	0.07	1.00
WT	X>G	GXT-AXA	-0.01	-0.09	0.06	1.00
WT	X>G	TXA-AXA	-0.02	-0.10	0.06	1.00
WT	X>G	TXC-AXA	0.03	-0.05	0.11	0.99
WT	X>G	TXG-AXA	-0.02	-0.10	0.06	1.00
WT	X>G	TXT-AXA	-0.02	-0.10	0.06	1.00
WT	X>G	AXG-AXC	-0.01	-0.09	0.07	1.00
WT	X>G	AXT-AXC	0.00	-0.08	0.07	1.00
WT	X>G	CXA-AXC	-0.02	-0.10	0.06	1.00
WT	X>G	CXC-AXC	-0.02	-0.10	0.06	1.00
WT	X>G	CXG-AXC	-0.02	-0.10	0.06	1.00
WT	X>G	CXT-AXC	-0.02	-0.10	0.06	1.00
WT	X>G	GXA-AXC	-0.02	-0.10	0.06	1.00
WT	X>G	GXC-AXC	0.00	-0.08	0.07	1.00
WT	X>G	GXG-AXC	-0.01	-0.09	0.07	1.00
WT	X>G	GXT-AXC	-0.01	-0.09	0.07	1.00
WT	X>G	TXA-AXC	-0.02	-0.10	0.06	1.00
WT	X>G	TXC-AXC	0.03	-0.05	0.11	0.98
WT	X>G	TXG-AXC	-0.02	-0.10	0.06	1.00
WT	X>G	TXT-AXC	-0.02	-0.10	0.06	1.00
WT	X>G	AXT-AXG	0.00	-0.07	0.08	1.00
WT	X>G	CXA-AXG	-0.01	-0.09	0.07	1.00
WT	X>G	CXC-AXG	-0.01	-0.09	0.07	1.00

WT	X>G	CXG-AXG	-0.01	-0.09	0.07	1.00
WT	X>G	CXT-AXG	-0.01	-0.09	0.07	1.00
WT	X>G	GXA-AXG	-0.01	-0.09	0.07	1.00
WT	X>G	GXC-AXG	0.01	-0.07	0.08	1.00
WT	X>G	GXG-AXG	0.00	-0.08	0.08	1.00
WT	X>G	GXT-AXG	0.00	-0.08	0.08	1.00
WT	X>G	TXA-AXG	-0.01	-0.09	0.07	1.00
WT	X>G	TXC-AXG	0.04	-0.04	0.12	0.85
WT	X>G	TXG-AXG	-0.01	-0.09	0.07	1.00
WT	X>G	TXT-AXG	-0.01	-0.09	0.07	1.00
WT	X>G	CXA-AXT	-0.01	-0.09	0.06	1.00
WT	X>G	CXC-AXT	-0.01	-0.09	0.06	1.00
WT	X>G	CXG-AXT	-0.01	-0.09	0.06	1.00
WT	X>G	CXT-AXT	-0.01	-0.09	0.06	1.00
WT	X>G	GXA-AXT	-0.01	-0.09	0.06	1.00
WT	X>G	GXC-AXT	0.00	-0.08	0.08	1.00
WT	X>G	GXG-AXT	0.00	-0.08	0.07	1.00
WT	X>G	GXT-AXT	-0.01	-0.08	0.07	1.00
WT	X>G	TXA-AXT	-0.01	-0.09	0.06	1.00
WT	X>G	TXC-AXT	0.04	-0.04	0.11	0.93
WT	X>G	TXG-AXT	-0.01	-0.09	0.06	1.00
WT	X>G	TXT-AXT	-0.01	-0.09	0.06	1.00
WT	X>G	CXC-CXA	0.00	-0.08	0.08	1.00
WT	X>G	CXG-CXA	0.00	-0.08	0.08	1.00
WT	X>G	CXT-CXA	0.00	-0.08	0.08	1.00
WT	X>G	GXA-CXA	0.00	-0.08	0.08	1.00
WT	X>G	GXC-CXA	0.01	-0.06	0.09	1.00
WT	X>G	GXG-CXA	0.01	-0.07	0.09	1.00
WT	X>G	GXT-CXA	0.01	-0.07	0.08	1.00
WT	X>G	TXA-CXA	0.00	-0.08	0.08	1.00
WT	X>G	TXC-CXA	0.05	-0.03	0.13	0.60
WT	X>G	TXG-CXA	0.00	-0.08	0.08	1.00
WT	X>G	TXT-CXA	0.00	-0.08	0.08	1.00
WT	X>G	CXG-CXC	0.00	-0.08	0.08	1.00
WT	X>G	CXT-CXC	0.00	-0.08	0.08	1.00
WT	X>G	GXA-CXC	0.00	-0.08	0.08	1.00
WT	X>G	GXC-CXC	0.01	-0.06	0.09	1.00
WT	X>G	GXG-CXC	0.01	-0.07	0.09	1.00
WT	X>G	GXT-CXC	0.01	-0.07	0.08	1.00
WT	X>G	TXA-CXC	0.00	-0.08	0.08	1.00

WT	X>G	TXC-CXC	0.05	-0.03	0.13	0.60
WT	X>G	TXG-CXC	0.00	-0.08	0.08	1.00
WT	X>G	TXT-CXC	0.00	-0.08	0.08	1.00
WT	X>G	CXT-CXG	0.00	-0.08	0.08	1.00
WT	X>G	GXA-CXG	0.00	-0.08	0.08	1.00
WT	X>G	GXC-CXG	0.01	-0.06	0.09	1.00
WT	X>G	GXG-CXG	0.01	-0.07	0.09	1.00
WT	X>G	GXT-CXG	0.01	-0.07	0.08	1.00
WT	X>G	TXA-CXG	0.00	-0.08	0.08	1.00
WT	X>G	TXC-CXG	0.05	-0.03	0.13	0.60
WT	X>G	TXG-CXG	0.00	-0.08	0.08	1.00
WT	X>G	TXT-CXG	0.00	-0.08	0.08	1.00
WT	X>G	GXA-CXT	0.00	-0.08	0.08	1.00
WT	X>G	GXC-CXT	0.01	-0.06	0.09	1.00
WT	X>G	GXG-CXT	0.01	-0.07	0.09	1.00
WT	X>G	GXT-CXT	0.01	-0.07	0.08	1.00
WT	X>G	TXA-CXT	0.00	-0.08	0.08	1.00
WT	X>G	TXC-CXT	0.05	-0.03	0.13	0.60
WT	X>G	TXG-CXT	0.00	-0.08	0.08	1.00
WT	X>G	TXT-CXT	0.00	-0.08	0.08	1.00
WT	X>G	GXC-GXA	0.01	-0.06	0.09	1.00
WT	X>G	GXG-GXA	0.01	-0.07	0.09	1.00
WT	X>G	GXT-GXA	0.01	-0.07	0.08	1.00
WT	X>G	TXA-GXA	0.00	-0.08	0.08	1.00
WT	X>G	TXC-GXA	0.05	-0.03	0.13	0.60
WT	X>G	TXG-GXA	0.00	-0.08	0.08	1.00
WT	X>G	TXT-GXA	0.00	-0.08	0.08	1.00
WT	X>G	GXG-GXC	0.00	-0.08	0.07	1.00
WT	X>G	GXT-GXC	-0.01	-0.08	0.07	1.00
WT	X>G	TXA-GXC	-0.01	-0.09	0.06	1.00
WT	X>G	TXC-GXC	0.04	-0.04	0.11	0.94
WT	X>G	TXG-GXC	-0.01	-0.09	0.06	1.00
WT	X>G	TXT-GXC	-0.01	-0.09	0.06	1.00
WT	X>G	GXT-GXG	0.00	-0.08	0.07	1.00
WT	X>G	TXA-GXG	-0.01	-0.09	0.07	1.00
WT	X>G	TXC-GXG	0.04	-0.04	0.12	0.88
WT	X>G	TXG-GXG	-0.01	-0.09	0.07	1.00
WT	X>G	TXT-GXG	-0.01	-0.09	0.07	1.00
WT	X>G	TXA-GXT	-0.01	-0.08	0.07	1.00
WT	X>G	TXC-GXT	0.04	-0.04	0.12	0.80

WT	X>G	TXG-GXT	-0.01	-0.08	0.07	1.00
WT	X>G	TXT-GXT	-0.01	-0.08	0.07	1.00
WT	X>G	TXC-TXA	0.05	-0.03	0.13	0.60
WT	X>G	TXG-TXA	0.00	-0.08	0.08	1.00
WT	X>G	TXT-TXA	0.00	-0.08	0.08	1.00
WT	X>G	TXG-TXC	-0.05	-0.13	0.03	0.60
WT	X>G	TXT-TXC	-0.05	-0.13	0.03	0.60
WT	X>G	TXT-TXG	0.00	-0.08	0.08	1.00
alkB-	X>G	AXC-AXA	-0.01	-0.26	0.24	1.00
alkB-	X>G	AXG-AXA	0.00	-0.25	0.25	1.00
alkB-	X>G	AXT-AXA	0.01	-0.24	0.26	1.00
alkB-	X>G	CXA-AXA	0.02	-0.23	0.27	1.00
alkB-	X>G	CXC-AXA	-0.01	-0.26	0.24	1.00
alkB-	X>G	CXG-AXA	-0.01	-0.26	0.24	1.00
alkB-	X>G	CXT-AXA	0.03	-0.22	0.28	1.00
alkB-	X>G	GXA-AXA	-0.01	-0.26	0.24	1.00
alkB-	X>G	GXC-AXA	0.00	-0.25	0.25	1.00
alkB-	X>G	GXG-AXA	0.00	-0.25	0.25	1.00
alkB-	X>G	GXT-AXA	0.00	-0.25	0.25	1.00
alkB-	X>G	TXA-AXA	-0.01	-0.26	0.24	1.00
alkB-	X>G	TXC-AXA	0.05	-0.20	0.30	1.00
alkB-	X>G	TXG-AXA	0.14	-0.11	0.39	0.73
alkB-	X>G	TXT-AXA	0.08	-0.17	0.33	1.00
alkB-	X>G	AXG-AXC	0.01	-0.24	0.26	1.00
alkB-	X>G	AXT-AXC	0.02	-0.23	0.27	1.00
alkB-	X>G	CXA-AXC	0.03	-0.22	0.27	1.00
alkB-	X>G	CXC-AXC	0.00	-0.25	0.25	1.00
alkB-	X>G	CXG-AXC	0.00	-0.25	0.25	1.00
alkB-	X>G	CXT-AXC	0.04	-0.21	0.29	1.00
alkB-	X>G	GXA-AXC	0.00	-0.25	0.25	1.00
alkB-	X>G	GXC-AXC	0.01	-0.24	0.26	1.00
alkB-	X>G	GXG-AXC	0.01	-0.24	0.26	1.00
alkB-	X>G	GXT-AXC	0.01	-0.24	0.26	1.00
alkB-	X>G	TXA-AXC	0.00	-0.25	0.25	1.00
alkB-	X>G	TXC-AXC	0.05	-0.20	0.30	1.00
alkB-	X>G	TXG-AXC	0.15	-0.10	0.40	0.65
alkB-	X>G	TXT-AXC	0.08	-0.16	0.33	0.99
alkB-	X>G	AXT-AXG	0.01	-0.24	0.26	1.00
alkB-	X>G	CXA-AXG	0.02	-0.23	0.27	1.00
alkB-	X>G	CXC-AXG	-0.01	-0.26	0.24	1.00

alkB-	X>G	CXG-AXG	-0.01	-0.26	0.24	1.00
alkB-	X>G	CXT-AXG	0.03	-0.22	0.28	1.00
alkB-	X>G	GXA-AXG	-0.01	-0.26	0.24	1.00
alkB-	X>G	GXC-AXG	0.00	-0.25	0.25	1.00
alkB-	X>G	GXG-AXG	0.00	-0.25	0.25	1.00
alkB-	X>G	GXT-AXG	0.00	-0.25	0.25	1.00
alkB-	X>G	TXA-AXG	-0.01	-0.26	0.24	1.00
alkB-	X>G	TXC-AXG	0.05	-0.20	0.30	1.00
alkB-	X>G	TXG-AXG	0.14	-0.11	0.39	0.73
alkB-	X>G	TXT-AXG	0.08	-0.17	0.33	1.00
alkB-	X>G	CXA-AXT	0.01	-0.24	0.26	1.00
alkB-	X>G	CXC-AXT	-0.02	-0.27	0.23	1.00
alkB-	X>G	CXG-AXT	-0.02	-0.27	0.23	1.00
alkB-	X>G	CXT-AXT	0.02	-0.23	0.27	1.00
alkB-	X>G	GXA-AXT	-0.02	-0.27	0.23	1.00
alkB-	X>G	GXC-AXT	-0.01	-0.26	0.24	1.00
alkB-	X>G	GXG-AXT	-0.01	-0.26	0.24	1.00
alkB-	X>G	GXT-AXT	-0.01	-0.26	0.24	1.00
alkB-	X>G	TXA-AXT	-0.02	-0.27	0.23	1.00
alkB-	X>G	TXC-AXT	0.04	-0.21	0.29	1.00
alkB-	X>G	TXG-AXT	0.13	-0.12	0.38	0.81
alkB-	X>G	TXT-AXT	0.07	-0.18	0.32	1.00
alkB-	X>G	CXC-CXA	-0.03	-0.27	0.22	1.00
alkB-	X>G	CXG-CXA	-0.03	-0.27	0.22	1.00
alkB-	X>G	CXT-CXA	0.01	-0.24	0.26	1.00
alkB-	X>G	GXA-CXA	-0.03	-0.27	0.22	1.00
alkB-	X>G	GXC-CXA	-0.01	-0.26	0.24	1.00
alkB-	X>G	GXG-CXA	-0.02	-0.27	0.23	1.00
alkB-	X>G	GXT-CXA	-0.02	-0.27	0.23	1.00
alkB-	X>G	TXA-CXA	-0.03	-0.27	0.22	1.00
alkB-	X>G	TXC-CXA	0.03	-0.22	0.28	1.00
alkB-	X>G	TXG-CXA	0.13	-0.12	0.38	0.86
alkB-	X>G	TXT-CXA	0.06	-0.19	0.31	1.00
alkB-	X>G	CXG-CXC	0.00	-0.25	0.25	1.00
alkB-	X>G	CXT-CXC	0.04	-0.21	0.29	1.00
alkB-	X>G	GXA-CXC	0.00	-0.25	0.25	1.00
alkB-	X>G	GXC-CXC	0.01	-0.24	0.26	1.00
alkB-	X>G	GXG-CXC	0.01	-0.24	0.26	1.00
alkB-	X>G	GXT-CXC	0.01	-0.24	0.26	1.00
alkB-	X>G	TXA-CXC	0.00	-0.25	0.25	1.00

alkB-	X>G	TXC-CXC	0.05	-0.20	0.30	1.00
alkB-	X>G	TXG-CXC	0.15	-0.10	0.40	0.65
alkB-	X>G	TXT-CXC	0.08	-0.16	0.33	0.99
alkB-	X>G	CXT-CXG	0.04	-0.21	0.29	1.00
alkB-	X>G	GXA-CXG	0.00	-0.25	0.25	1.00
alkB-	X>G	GXC-CXG	0.01	-0.24	0.26	1.00
alkB-	X>G	GXG-CXG	0.01	-0.24	0.26	1.00
alkB-	X>G	GXT-CXG	0.01	-0.24	0.26	1.00
alkB-	X>G	TXA-CXG	0.00	-0.25	0.25	1.00
alkB-	X>G	TXC-CXG	0.05	-0.20	0.30	1.00
alkB-	X>G	TXG-CXG	0.15	-0.10	0.40	0.65
alkB-	X>G	TXT-CXG	0.08	-0.16	0.33	0.99
alkB-	X>G	GXA-CXT	-0.04	-0.29	0.21	1.00
alkB-	X>G	GXC-CXT	-0.03	-0.28	0.22	1.00
alkB-	X>G	GXG-CXT	-0.03	-0.28	0.22	1.00
alkB-	X>G	GXT-CXT	-0.03	-0.28	0.22	1.00
alkB-	X>G	TXA-CXT	-0.04	-0.29	0.21	1.00
alkB-	X>G	TXC-CXT	0.02	-0.23	0.27	1.00
alkB-	X>G	TXG-CXT	0.11	-0.14	0.36	0.93
alkB-	X>G	TXT-CXT	0.05	-0.20	0.30	1.00
alkB-	X>G	GXC-GXA	0.01	-0.24	0.26	1.00
alkB-	X>G	GXG-GXA	0.01	-0.24	0.26	1.00
alkB-	X>G	GXT-GXA	0.01	-0.24	0.26	1.00
alkB-	X>G	TXA-GXA	0.00	-0.25	0.25	1.00
alkB-	X>G	TXC-GXA	0.05	-0.20	0.30	1.00
alkB-	X>G	TXG-GXA	0.15	-0.10	0.40	0.65
alkB-	X>G	TXT-GXA	0.08	-0.16	0.33	0.99
alkB-	X>G	GXG-GXC	0.00	-0.25	0.25	1.00
alkB-	X>G	GXT-GXC	0.00	-0.25	0.25	1.00
alkB-	X>G	TXA-GXC	-0.01	-0.26	0.24	1.00
alkB-	X>G	TXC-GXC	0.04	-0.21	0.29	1.00
alkB-	X>G	TXG-GXC	0.14	-0.11	0.39	0.75
alkB-	X>G	TXT-GXC	0.07	-0.18	0.32	1.00
alkB-	X>G	GXT-GXG	0.00	-0.25	0.25	1.00
alkB-	X>G	TXA-GXG	-0.01	-0.26	0.24	1.00
alkB-	X>G	TXC-GXG	0.05	-0.20	0.30	1.00
alkB-	X>G	TXG-GXG	0.14	-0.11	0.39	0.72
alkB-	X>G	TXT-GXG	0.08	-0.17	0.33	1.00
alkB-	X>G	TXA-GXT	-0.01	-0.26	0.24	1.00
alkB-	X>G	TXC-GXT	0.05	-0.20	0.30	1.00

alkB-	X>G	TXG-GXT	0.14	-0.10	0.39	0.72
alkB-	X>G	TXT-GXT	0.08	-0.17	0.33	1.00
alkB-	X>G	TXC-TXA	0.05	-0.20	0.30	1.00
alkB-	X>G	TXG-TXA	0.15	-0.10	0.40	0.65
alkB-	X>G	TXT-TXA	0.08	-0.16	0.33	0.99
alkB-	X>G	TXG-TXC	0.10	-0.15	0.35	0.98
alkB-	X>G	TXT-TXC	0.03	-0.22	0.28	1.00
alkB-	X>G	TXT-TXG	-0.07	-0.32	0.18	1.00
WT	X>T	AXC-AXA	-0.13	-1.44	1.19	1.00
WT	X>T	AXG-AXA	-0.38	-1.70	0.93	1.00
WT	X>T	AXT-AXA	-0.85	-2.16	0.47	0.57
WT	X>T	CXA-AXA	-0.13	-1.44	1.19	1.00
WT	X>T	CXC-AXA	-0.27	-1.59	1.04	1.00
WT	X>T	CXG-AXA	-0.32	-1.63	1.00	1.00
WT	X>T	CXT-AXA	-0.56	-1.87	0.76	0.96
WT	X>T	GXA-AXA	-0.25	-1.57	1.06	1.00
WT	X>T	GXC-AXA	-1.41	-2.73	-0.10	0.03
WT	X>T	GXG-AXA	-1.37	-2.69	-0.06	0.03
WT	X>T	GXT-AXA	-1.62	-2.94	-0.31	0.01
WT	X>T	TXA-AXA	-0.10	-1.41	1.22	1.00
WT	X>T	TXC-AXA	-0.73	-2.05	0.58	0.78
WT	X>T	TXG-AXA	-0.13	-1.44	1.19	1.00
WT	X>T	TXT-AXA	-0.47	-1.78	0.85	0.99
WT	X>T	AXG-AXC	-0.25	-1.57	1.06	1.00
WT	X>T	AXT-AXC	-0.72	-2.04	0.59	0.79
WT	X>T	CXA-AXC	0.00	-1.32	1.31	1.00
WT	X>T	CXC-AXC	-0.15	-1.46	1.17	1.00
WT	X>T	CXG-AXC	-0.19	-1.51	1.12	1.00
WT	X>T	CXT-AXC	-0.43	-1.75	0.88	1.00
WT	X>T	GXA-AXC	-0.13	-1.44	1.19	1.00
WT	X>T	GXC-AXC	-1.28	-2.60	0.03	0.06
WT	X>T	GXG-AXC	-1.25	-2.56	0.07	0.08
WT	X>T	GXT-AXC	-1.49	-2.81	-0.18	0.01
WT	X>T	TXA-AXC	0.03	-1.29	1.34	1.00
WT	X>T	TXC-AXC	-0.60	-1.92	0.71	0.93
WT	X>T	TXG-AXC	0.00	-1.32	1.31	1.00
WT	X>T	TXT-AXC	-0.34	-1.66	0.97	1.00
WT	X>T	AXT-AXG	-0.47	-1.78	0.85	0.99
WT	X>T	CXA-AXG	0.25	-1.06	1.57	1.00
WT	X>T	CXC-AXG	0.11	-1.21	1.42	1.00

WT	X>T	CXG-AXG	0.06	-1.25	1.38	1.00
WT	X>T	CXT-AXG	-0.18	-1.49	1.14	1.00
WT	X>T	GXA-AXG	0.13	-1.19	1.44	1.00
WT	X>T	GXC-AXG	-1.03	-2.35	0.28	0.27
WT	X>T	GXG-AXG	-0.99	-2.31	0.32	0.32
WT	X>T	GXT-AXG	-1.24	-2.56	0.07	0.08
WT	X>T	TXA-AXG	0.28	-1.03	1.60	1.00
WT	X>T	TXC-AXG	-0.35	-1.67	0.97	1.00
WT	X>T	TXG-AXG	0.25	-1.06	1.57	1.00
WT	X>T	TXT-AXG	-0.09	-1.40	1.23	1.00
WT	X>T	CXA-AXT	0.72	-0.60	2.03	0.80
WT	X>T	CXC-AXT	0.57	-0.74	1.89	0.95
WT	X>T	CXG-AXT	0.53	-0.79	1.85	0.98
WT	X>T	CXT-AXT	0.29	-1.03	1.60	1.00
WT	X>T	GXA-AXT	0.60	-0.72	1.91	0.94
WT	X>T	GXC-AXT	-0.56	-1.88	0.75	0.96
WT	X>T	GXG-AXT	-0.53	-1.84	0.79	0.98
WT	X>T	GXT-AXT	-0.77	-2.09	0.54	0.70
WT	X>T	TXA-AXT	0.75	-0.57	2.06	0.75
WT	X>T	TXC-AXT	0.12	-1.20	1.43	1.00
WT	X>T	TXG-AXT	0.72	-0.59	2.04	0.79
WT	X>T	TXT-AXT	0.38	-0.94	1.69	1.00
WT	X>T	CXC-CXA	-0.14	-1.46	1.17	1.00
WT	X>T	CXG-CXA	-0.19	-1.50	1.13	1.00
WT	X>T	CXT-CXA	-0.43	-1.74	0.89	1.00
WT	X>T	GXA-CXA	-0.12	-1.44	1.19	1.00
WT	X>T	GXC-CXA	-1.28	-2.60	0.03	0.06
WT	X>T	GXG-CXA	-1.24	-2.56	0.07	0.08
WT	X>T	GXT-CXA	-1.49	-2.81	-0.18	0.01
WT	X>T	TXA-CXA	0.03	-1.28	1.35	1.00
WT	X>T	TXC-CXA	-0.60	-1.92	0.71	0.93
WT	X>T	TXG-CXA	0.00	-1.31	1.32	1.00
WT	X>T	TXT-CXA	-0.34	-1.65	0.98	1.00
WT	X>T	CXG-CXC	-0.04	-1.36	1.27	1.00
WT	X>T	CXT-CXC	-0.28	-1.60	1.03	1.00
WT	X>T	GXA-CXC	0.02	-1.29	1.34	1.00
WT	X>T	GXC-CXC	-1.14	-2.45	0.18	0.15
WT	X>T	GXG-CXC	-1.10	-2.42	0.21	0.18
WT	X>T	GXT-CXC	-1.35	-2.66	-0.03	0.04
WT	X>T	TXA-CXC	0.17	-1.14	1.49	1.00

WT	X>T	TXC-CXC	-0.46	-1.77	0.86	0.99
WT	X>T	TXG-CXC	0.15	-1.17	1.46	1.00
WT	X>T	TXT-CXC	-0.19	-1.51	1.12	1.00
WT	X>T	CXT-CXG	-0.24	-1.56	1.07	1.00
WT	X>T	GXA-CXG	0.07	-1.25	1.38	1.00
WT	X>T	GXC-CXG	-1.09	-2.41	0.22	0.19
WT	X>T	GXG-CXG	-1.06	-2.37	0.26	0.23
WT	X>T	GXT-CXG	-1.30	-2.62	0.01	0.05
WT	X>T	TXA-CXG	0.22	-1.10	1.53	1.00
WT	X>T	TXC-CXG	-0.41	-1.73	0.90	1.00
WT	X>T	TXG-CXG	0.19	-1.12	1.51	1.00
WT	X>T	TXT-CXG	-0.15	-1.47	1.16	1.00
WT	X>T	GXA-CXT	0.31	-1.01	1.62	1.00
WT	X>T	GXC-CXT	-0.85	-2.17	0.46	0.56
WT	X>T	GXG-CXT	-0.82	-2.13	0.50	0.63
WT	X>T	GXT-CXT	-1.06	-2.38	0.25	0.23
WT	X>T	TXA-CXT	0.46	-0.86	1.77	0.99
WT	X>T	TXC-CXT	-0.17	-1.49	1.14	1.00
WT	X>T	TXG-CXT	0.43	-0.88	1.75	1.00
WT	X>T	TXT-CXT	0.09	-1.22	1.41	1.00
WT	X>T	GXC-GXA	-1.16	-2.47	0.16	0.13
WT	X>T	GXG-GXA	-1.12	-2.44	0.19	0.16
WT	X>T	GXT-GXA	-1.37	-2.68	-0.05	0.03
WT	X>T	TXA-GXA	0.15	-1.16	1.47	1.00
WT	X>T	TXC-GXA	-0.48	-1.79	0.84	0.99
WT	X>T	TXG-GXA	0.13	-1.19	1.44	1.00
WT	X>T	TXT-GXA	-0.22	-1.53	1.10	1.00
WT	X>T	GXG-GXC	0.04	-1.28	1.35	1.00
WT	X>T	GXT-GXC	-0.21	-1.53	1.10	1.00
WT	X>T	TXA-GXC	1.31	0.00	2.63	0.05
WT	X>T	TXC-GXC	0.68	-0.63	2.00	0.85
WT	X>T	TXG-GXC	1.28	-0.03	2.60	0.06
WT	X>T	TXT-GXC	0.94	-0.37	2.26	0.40
WT	X>T	GXT-GXG	-0.25	-1.56	1.07	1.00
WT	X>T	TXA-GXG	1.28	-0.04	2.59	0.06
WT	X>T	TXC-GXG	0.64	-0.67	1.96	0.89
WT	X>T	TXG-GXG	1.25	-0.07	2.56	0.08
WT	X>T	TXT-GXG	0.91	-0.41	2.22	0.46
WT	X>T	TXA-GXT	1.52	0.21	2.84	0.01
WT	X>T	TXC-GXT	0.89	-0.42	2.21	0.49

WT	X>T	TXG-GXT	1.49	0.18	2.81	0.01
WT	X>T	TXT-GXT	1.15	-0.16	2.47	0.14
WT	X>T	TXC-TXA	-0.63	-1.95	0.68	0.91
WT	X>T	TXG-TXA	-0.03	-1.34	1.29	1.00
WT	X>T	TXT-TXA	-0.37	-1.68	0.95	1.00
WT	X>T	TXG-TXC	0.60	-0.71	1.92	0.93
WT	X>T	TXT-TXC	0.26	-1.05	1.58	1.00
WT	X>T	TXT-TXG	-0.34	-1.66	0.97	1.00
alkB-	X>T	AXC-AXA	-1.12	-2.93	0.69	0.63
alkB-	X>T	AXG-AXA	-0.64	-2.45	1.18	0.99
alkB-	X>T	AXT-AXA	-0.17	-1.98	1.64	1.00
alkB-	X>T	CXA-AXA	-0.09	-1.90	1.72	1.00
alkB-	X>T	CXC-AXA	-0.20	-2.01	1.61	1.00
alkB-	X>T	CXG-AXA	-0.28	-2.09	1.53	1.00
alkB-	X>T	CXT-AXA	-0.26	-2.07	1.55	1.00
alkB-	X>T	GXA-AXA	-0.29	-2.10	1.52	1.00
alkB-	X>T	GXC-AXA	-1.63	-3.44	0.18	0.12
alkB-	X>T	GXG-AXA	-1.38	-3.19	0.44	0.31
alkB-	X>T	GXT-AXA	-1.60	-3.41	0.21	0.13
alkB-	X>T	TXA-AXA	-0.74	-2.56	1.07	0.97
alkB-	X>T	TXC-AXA	-0.33	-2.14	1.48	1.00
alkB-	X>T	TXG-AXA	-0.21	-2.02	1.60	1.00
alkB-	X>T	TXT-AXA	-1.12	-2.93	0.69	0.63
alkB-	X>T	AXG-AXC	0.49	-1.32	2.30	1.00
alkB-	X>T	AXT-AXC	0.95	-0.86	2.76	0.84
alkB-	X>T	CXA-AXC	1.03	-0.78	2.84	0.75
alkB-	X>T	CXC-AXC	0.92	-0.89	2.73	0.87
alkB-	X>T	CXG-AXC	0.84	-0.97	2.65	0.93
alkB-	X>T	CXT-AXC	0.86	-0.95	2.67	0.91
alkB-	X>T	GXA-AXC	0.83	-0.98	2.64	0.93
alkB-	X>T	GXC-AXC	-0.51	-2.32	1.31	1.00
alkB-	X>T	GXG-AXC	-0.25	-2.06	1.56	1.00
alkB-	X>T	GXT-AXC	-0.48	-2.29	1.33	1.00
alkB-	X>T	TXA-AXC	0.38	-1.43	2.19	1.00
alkB-	X>T	TXC-AXC	0.79	-1.02	2.60	0.95
alkB-	X>T	TXG-AXC	0.91	-0.90	2.72	0.87
alkB-	X>T	TXT-AXC	0.00	-1.81	1.81	1.00
alkB-	X>T	AXT-AXG	0.46	-1.35	2.27	1.00
alkB-	X>T	CXA-AXG	0.54	-1.27	2.35	1.00
alkB-	X>T	CXC-AXG	0.43	-1.38	2.24	1.00

alkB-	X>T	CXG-AXG	0.35	-1.46	2.16	1.00
alkB-	X>T	CXT-AXG	0.37	-1.44	2.18	1.00
alkB-	X>T	GXA-AXG	0.35	-1.47	2.16	1.00
alkB-	X>T	GXC-AXG	-0.99	-2.80	0.82	0.79
alkB-	X>T	GXG-AXG	-0.74	-2.55	1.07	0.97
alkB-	X>T	GXT-AXG	-0.97	-2.78	0.84	0.82
alkB-	X>T	TXA-AXG	-0.11	-1.92	1.70	1.00
alkB-	X>T	TXC-AXG	0.30	-1.51	2.11	1.00
alkB-	X>T	TXG-AXG	0.42	-1.39	2.23	1.00
alkB-	X>T	TXT-AXG	-0.49	-2.30	1.32	1.00
alkB-	X>T	CXA-AXT	0.08	-1.73	1.89	1.00
alkB-	X>T	CXC-AXT	-0.03	-1.84	1.78	1.00
alkB-	X>T	CXG-AXT	-0.11	-1.92	1.70	1.00
alkB-	X>T	CXT-AXT	-0.09	-1.90	1.72	1.00
alkB-	X>T	GXA-AXT	-0.12	-1.93	1.69	1.00
alkB-	X>T	GXC-AXT	-1.45	-3.27	0.36	0.23
alkB-	X>T	GXG-AXT	-1.20	-3.01	0.61	0.52
alkB-	X>T	GXT-AXT	-1.43	-3.24	0.38	0.26
alkB-	X>T	TXA-AXT	-0.57	-2.38	1.24	1.00
alkB-	X>T	TXC-AXT	-0.16	-1.97	1.65	1.00
alkB-	X>T	TXG-AXT	-0.04	-1.85	1.77	1.00
alkB-	X>T	TXT-AXT	-0.95	-2.76	0.86	0.83
alkB-	X>T	CXC-CXA	-0.11	-1.92	1.70	1.00
alkB-	X>T	CXG-CXA	-0.19	-2.00	1.62	1.00
alkB-	X>T	CXT-CXA	-0.17	-1.98	1.64	1.00
alkB-	X>T	GXA-CXA	-0.20	-2.01	1.61	1.00
alkB-	X>T	GXC-CXA	-1.53	-3.34	0.28	0.17
alkB-	X>T	GXG-CXA	-1.28	-3.09	0.53	0.42
alkB-	X>T	GXT-CXA	-1.51	-3.32	0.30	0.19
alkB-	X>T	TXA-CXA	-0.65	-2.46	1.16	0.99
alkB-	X>T	TXC-CXA	-0.24	-2.05	1.57	1.00
alkB-	X>T	TXG-CXA	-0.12	-1.93	1.69	1.00
alkB-	X>T	TXT-CXA	-1.03	-2.84	0.78	0.75
alkB-	X>T	CXG-CXC	-0.08	-1.89	1.73	1.00
alkB-	X>T	CXT-CXC	-0.06	-1.87	1.75	1.00
alkB-	X>T	GXA-CXC	-0.09	-1.90	1.72	1.00
alkB-	X>T	GXC-CXC	-1.42	-3.23	0.39	0.26
alkB-	X>T	GXG-CXC	-1.17	-2.98	0.64	0.56
alkB-	X>T	GXT-CXC	-1.40	-3.21	0.41	0.29
alkB-	X>T	TXA-CXC	-0.54	-2.35	1.27	1.00

alkB-	X>T	TXC-CXC	-0.13	-1.94	1.68	1.00
alkB-	X>T	TXG-CXC	-0.01	-1.82	1.80	1.00
alkB-	X>T	TXT-CXC	-0.92	-2.73	0.89	0.87
alkB-	X>T	CXT-CXG	0.02	-1.79	1.83	1.00
alkB-	X>T	GXA-CXG	-0.01	-1.82	1.80	1.00
alkB-	X>T	GXC-CXG	-1.34	-3.15	0.47	0.34
alkB-	X>T	GXG-CXG	-1.09	-2.90	0.72	0.67
alkB-	X>T	GXT-CXG	-1.32	-3.13	0.49	0.37
alkB-	X>T	TXA-CXG	-0.46	-2.27	1.35	1.00
alkB-	X>T	TXC-CXG	-0.05	-1.86	1.76	1.00
alkB-	X>T	TXG-CXG	0.07	-1.74	1.88	1.00
alkB-	X>T	TXT-CXG	-0.84	-2.65	0.97	0.93
alkB-	X>T	GXA-CXT	-0.03	-1.84	1.78	1.00
alkB-	X>T	GXC-CXT	-1.36	-3.17	0.45	0.32
alkB-	X>T	GXG-CXT	-1.11	-2.92	0.70	0.64
alkB-	X>T	GXT-CXT	-1.34	-3.15	0.47	0.35
alkB-	X>T	TXA-CXT	-0.48	-2.29	1.33	1.00
alkB-	X>T	TXC-CXT	-0.07	-1.88	1.74	1.00
alkB-	X>T	TXG-CXT	0.05	-1.76	1.86	1.00
alkB-	X>T	TXT-CXT	-0.86	-2.67	0.95	0.91
alkB-	X>T	GXC-GXA	-1.34	-3.15	0.47	0.35
alkB-	X>T	GXG-GXA	-1.08	-2.90	0.73	0.68
alkB-	X>T	GXT-GXA	-1.31	-3.12	0.50	0.38
alkB-	X>T	TXA-GXA	-0.45	-2.27	1.36	1.00
alkB-	X>T	TXC-GXA	-0.04	-1.85	1.77	1.00
alkB-	X>T	TXG-GXA	0.08	-1.73	1.89	1.00
alkB-	X>T	TXT-GXA	-0.83	-2.64	0.98	0.93
alkB-	X>T	GXG-GXC	0.25	-1.56	2.06	1.00
alkB-	X>T	GXT-GXC	0.02	-1.79	1.84	1.00
alkB-	X>T	TXA-GXC	0.88	-0.93	2.69	0.90
alkB-	X>T	TXC-GXC	1.29	-0.52	3.10	0.40
alkB-	X>T	TXG-GXC	1.41	-0.40	3.22	0.27
alkB-	X>T	TXT-GXC	0.50	-1.31	2.31	1.00
alkB-	X>T	GXT-GXG	-0.23	-2.04	1.58	1.00
alkB-	X>T	TXA-GXG	0.63	-1.18	2.44	0.99
alkB-	X>T	TXC-GXG	1.04	-0.77	2.85	0.73
alkB-	X>T	TXG-GXG	1.16	-0.65	2.97	0.57
alkB-	X>T	TXT-GXG	0.25	-1.56	2.06	1.00
alkB-	X>T	TXA-GXT	0.86	-0.95	2.67	0.92
alkB-	X>T	TXC-GXT	1.27	-0.54	3.08	0.43

alkB-	X>T	TXG-GXT	1.39	-0.42	3.20	0.30
alkB-	X>T	TXT-GXT	0.48	-1.33	2.29	1.00
alkB-	X>T	TXC-TXA	0.41	-1.40	2.22	1.00
alkB-	X>T	TXG-TXA	0.53	-1.28	2.34	1.00
alkB-	X>T	TXT-TXA	-0.38	-2.19	1.43	1.00
alkB-	X>T	TXG-TXC	0.12	-1.69	1.93	1.00
alkB-	X>T	TXT-TXC	-0.79	-2.60	1.02	0.95
alkB-	X>T	TXT-TXG	-0.91	-2.72	0.90	0.87

NMR data

2'-Deoxy-7,8-dihydro-8-oxo-1,N^6-ethenoadenosine **2**

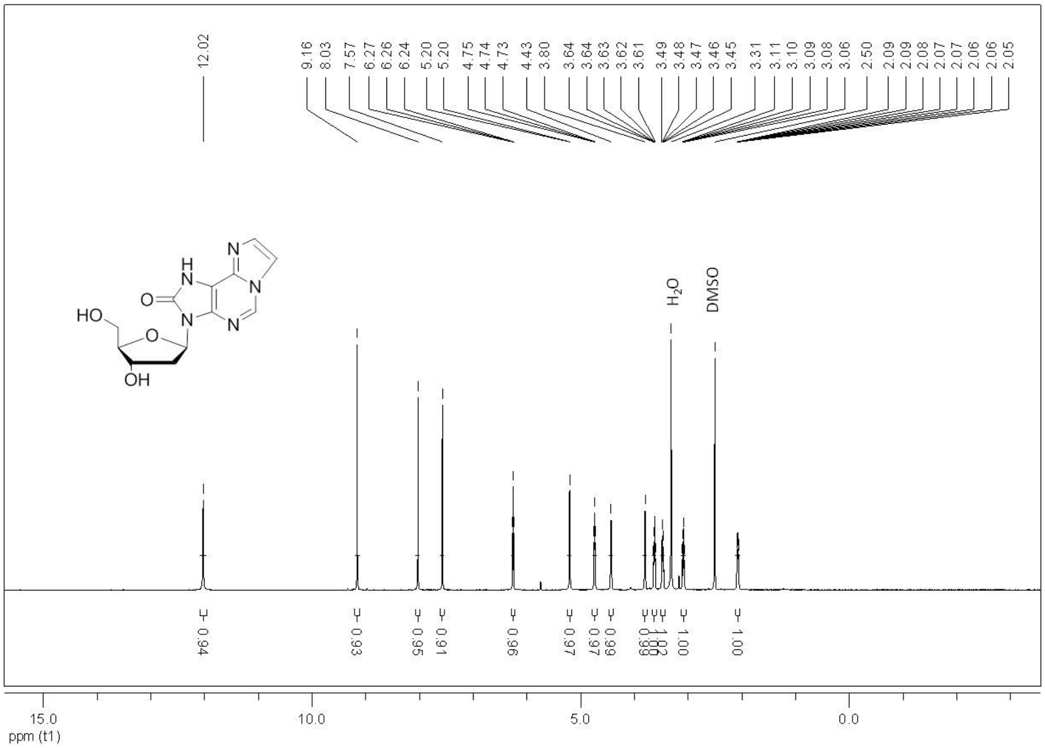

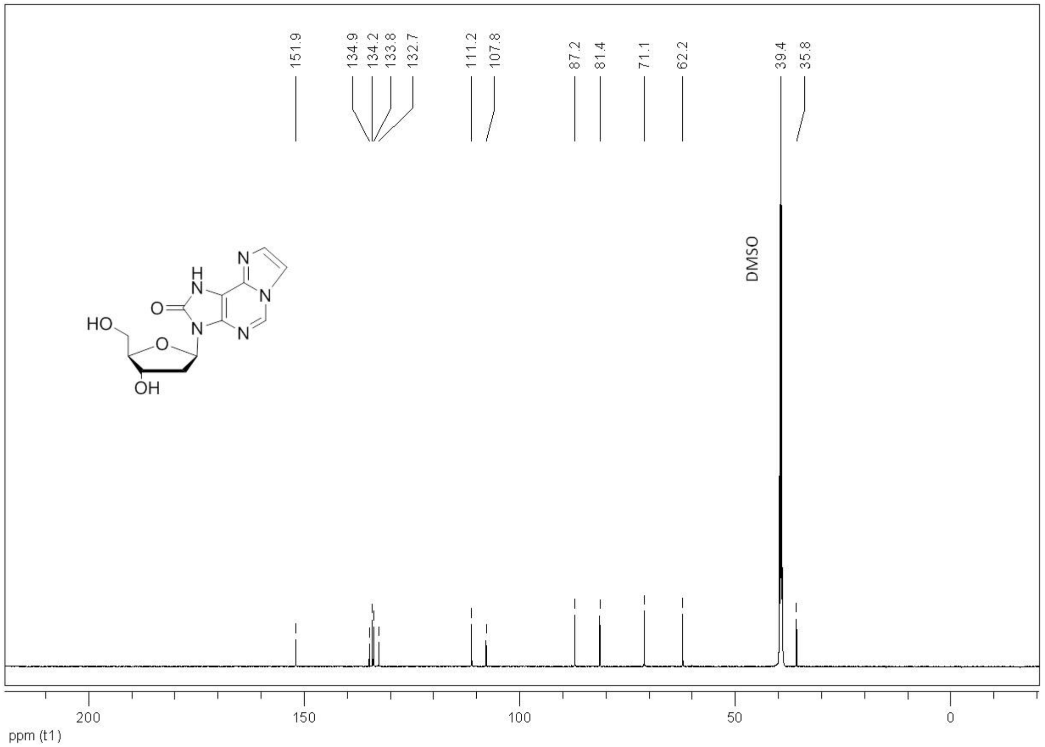

5'-O-(4,4'-Dimethoxytrityl)-2'-deoxy-7,8-dihydro-8-oxo-1,N^6-ethenoadenosine **3**

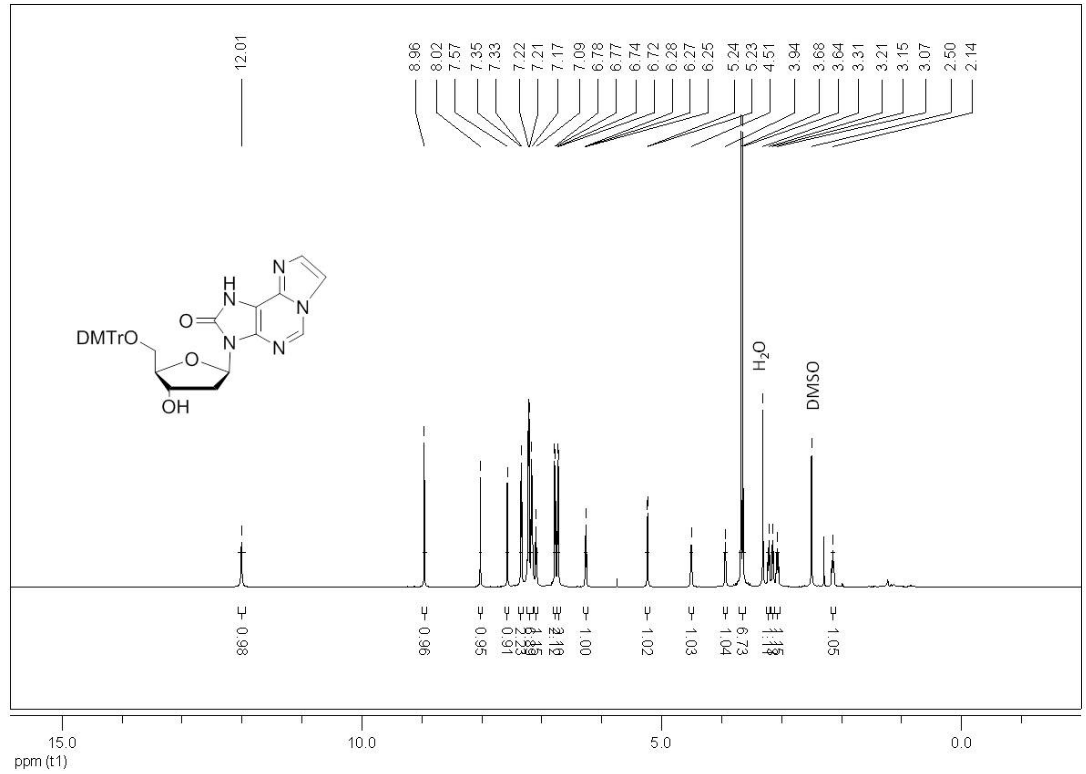

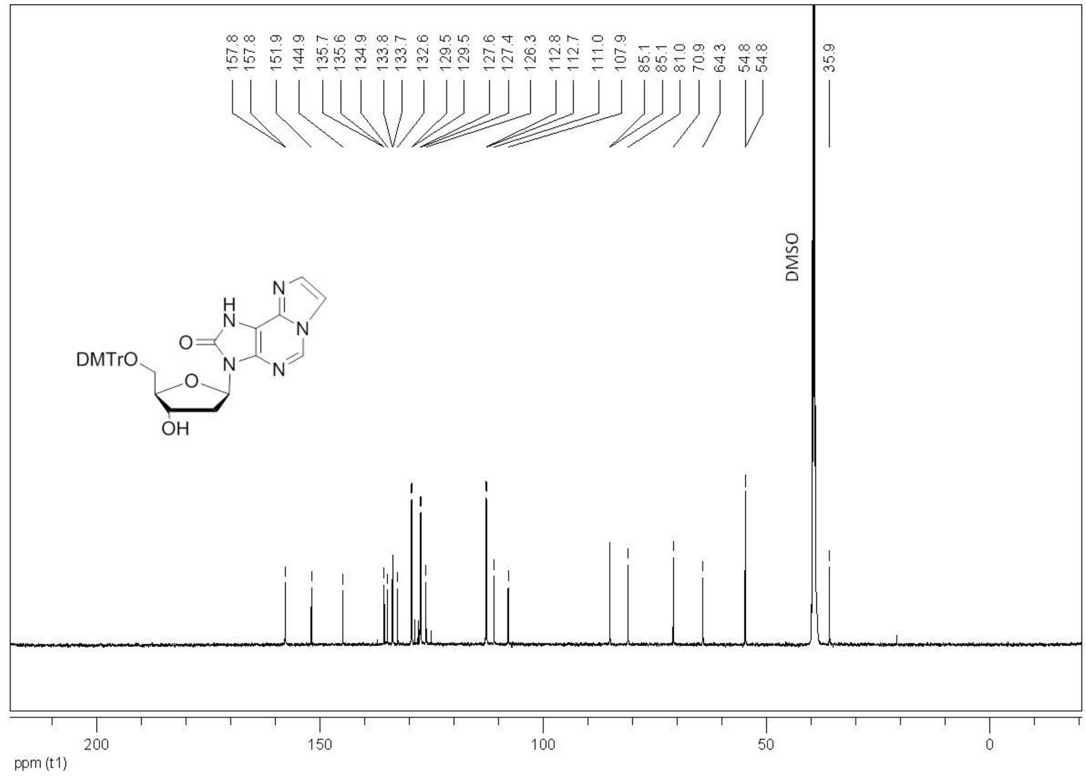

7,8-Dihydro-8-oxo-1,N⁶-ethenoadenine-N⁹-β-[2'-deoxy-3'-O-(2-cyanoethyl)-N,N-diisopropylphosphoramidite-5'-O-(4,4'-dimethoxytrityl)-ribonucleoside] **4**

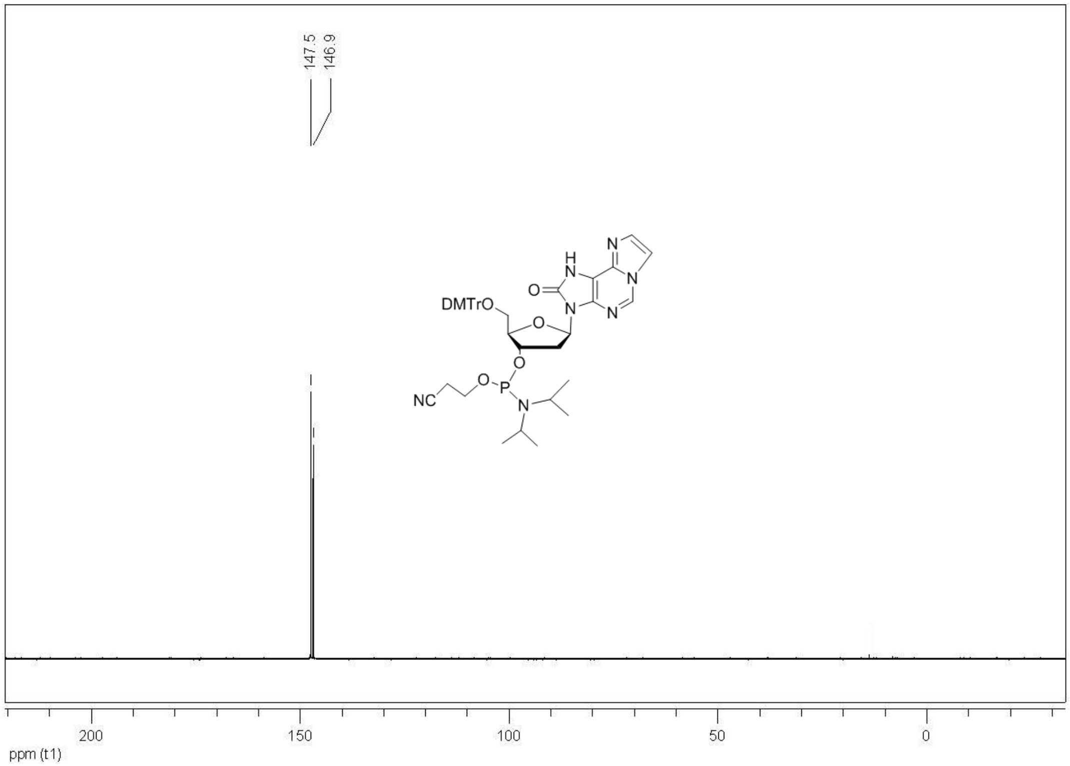

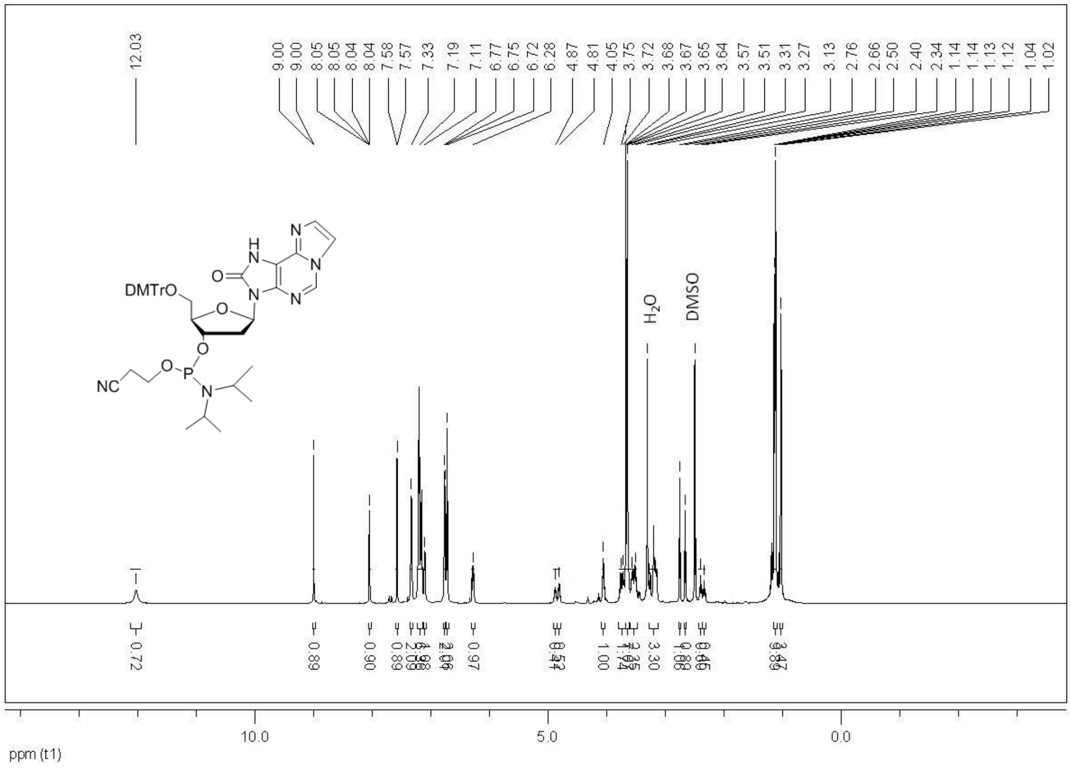

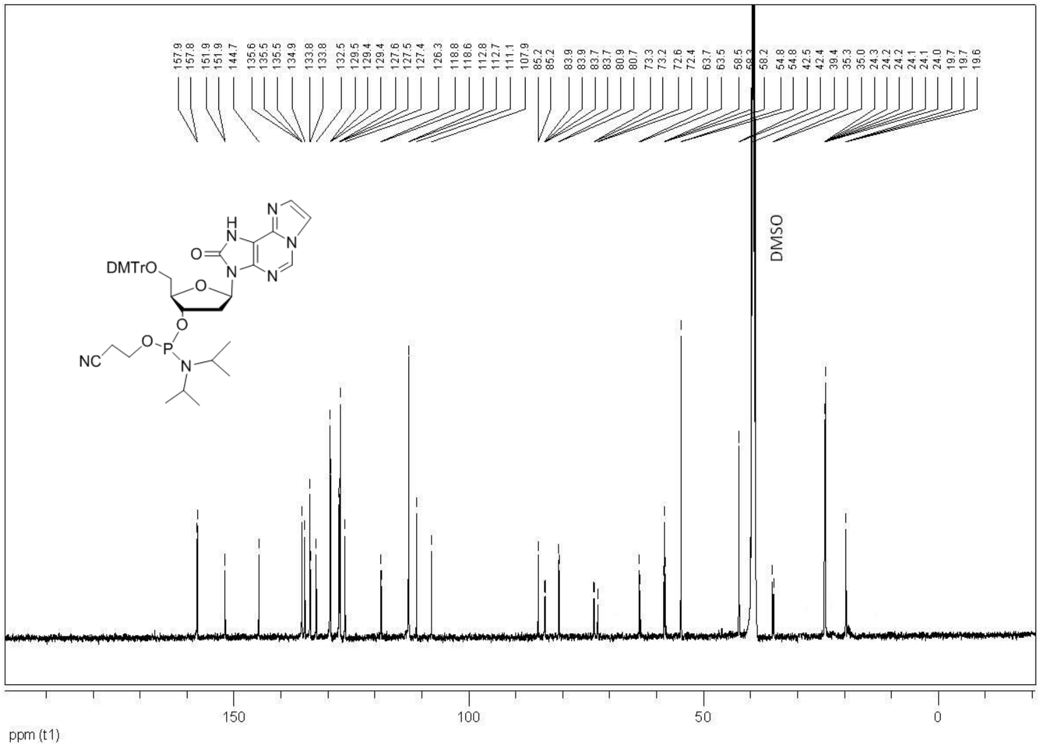
157.9 157.8 151.9 151.9 144.7 135.6 135.5 135.5 134.9 133.8 133.8 132.5 129.5 129.4 129.4 127.6 127.5 127.4 126.3 118.8 118.6 112.8 112.7 111.1 107.9 85.2 85.2 83.9 83.9 83.7 83.7 80.9 80.7 73.3 73.2 72.6 72.4 63.7 63.5 58.5 58.2 54.8 54.8 42.5 42.4 39.4 35.3 35.0 24.3 24.2 24.2 24.1 24.1 24.0 19.7 19.7 19.6
DMSO
DMTrO
O
NC
P
N
H
150
100
50
0
ppm (t1)

HPLC and MS data

ODN1 GCAA**X**ATACG

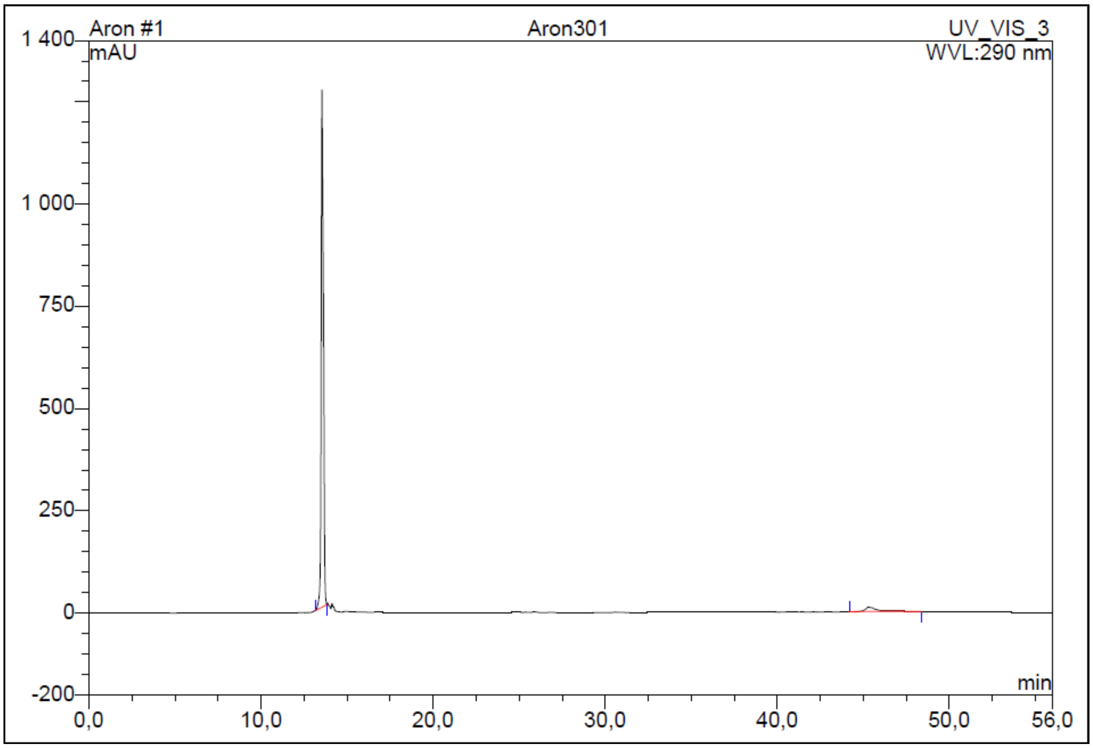

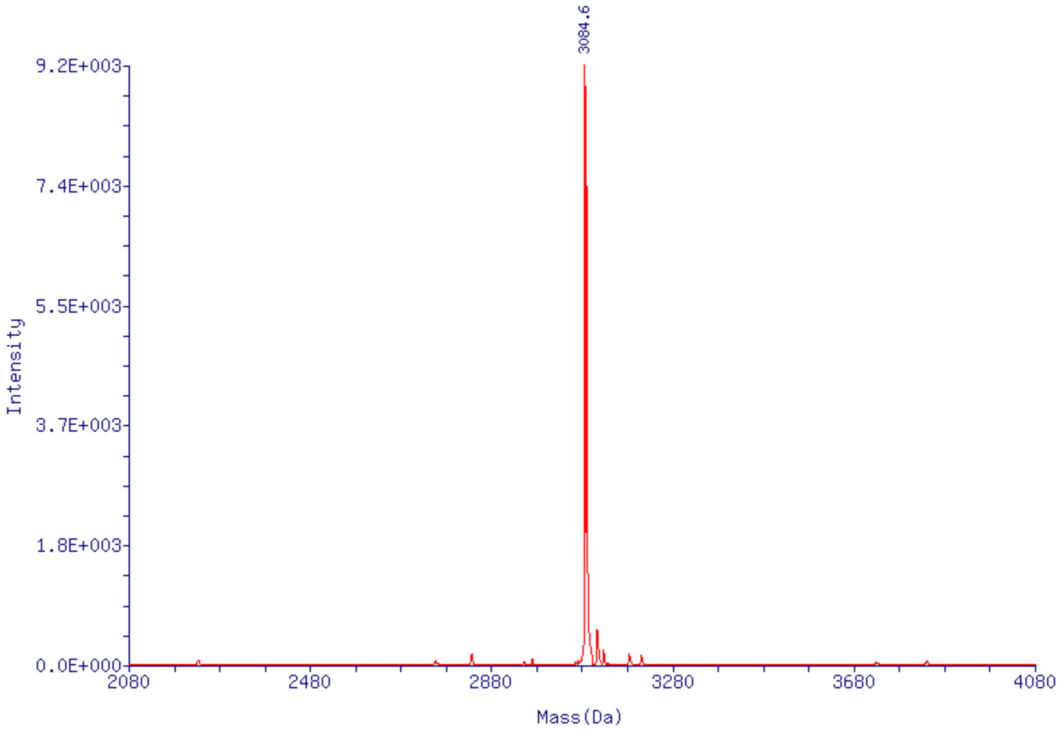

ODN2 GCAT**X**TTACG

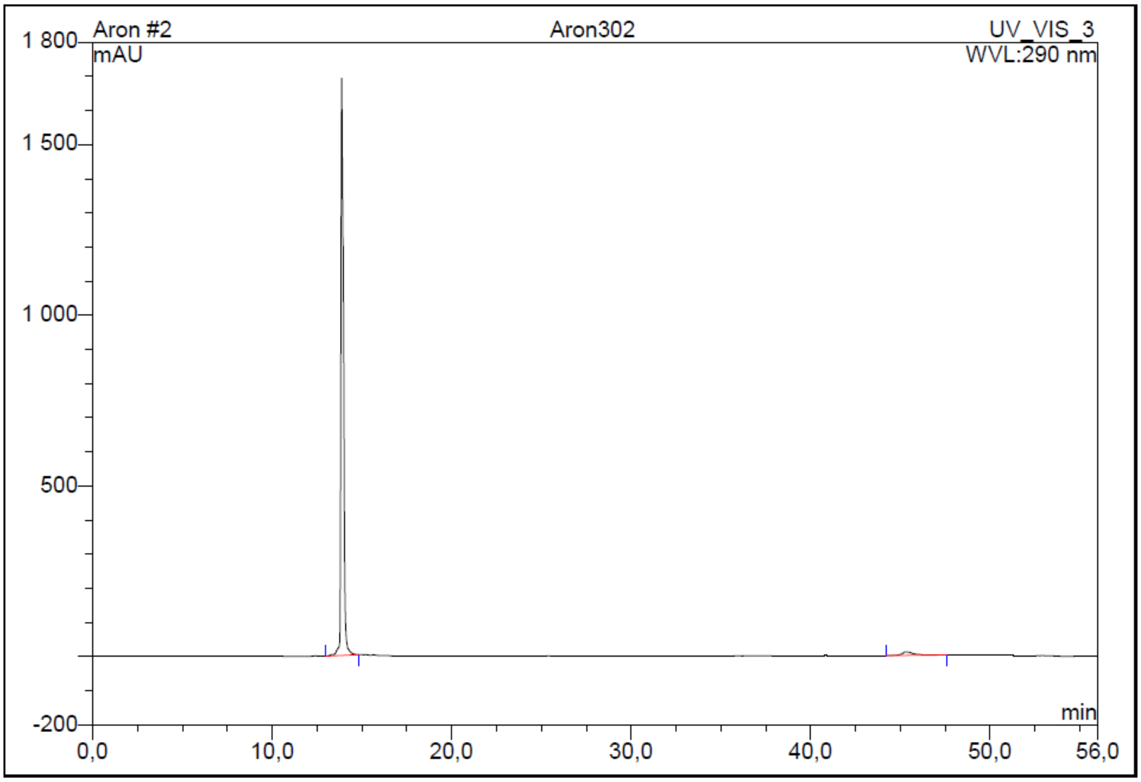

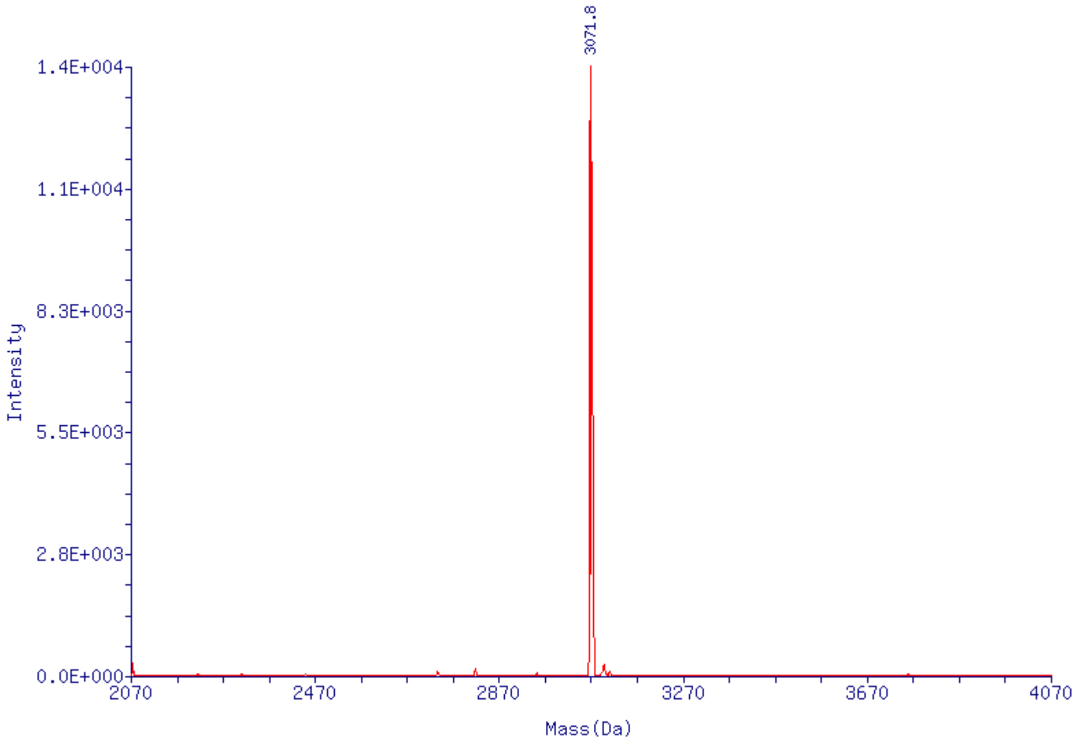

ODN3 GCAC**X**CTACG

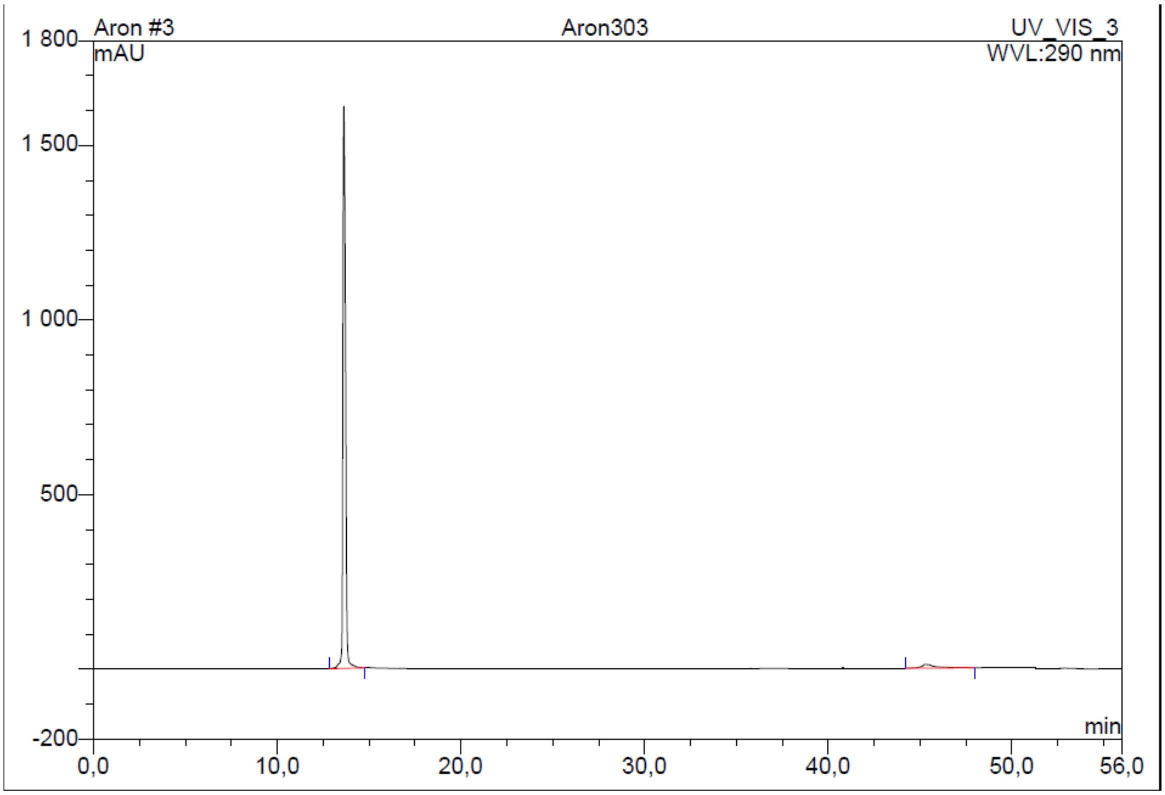

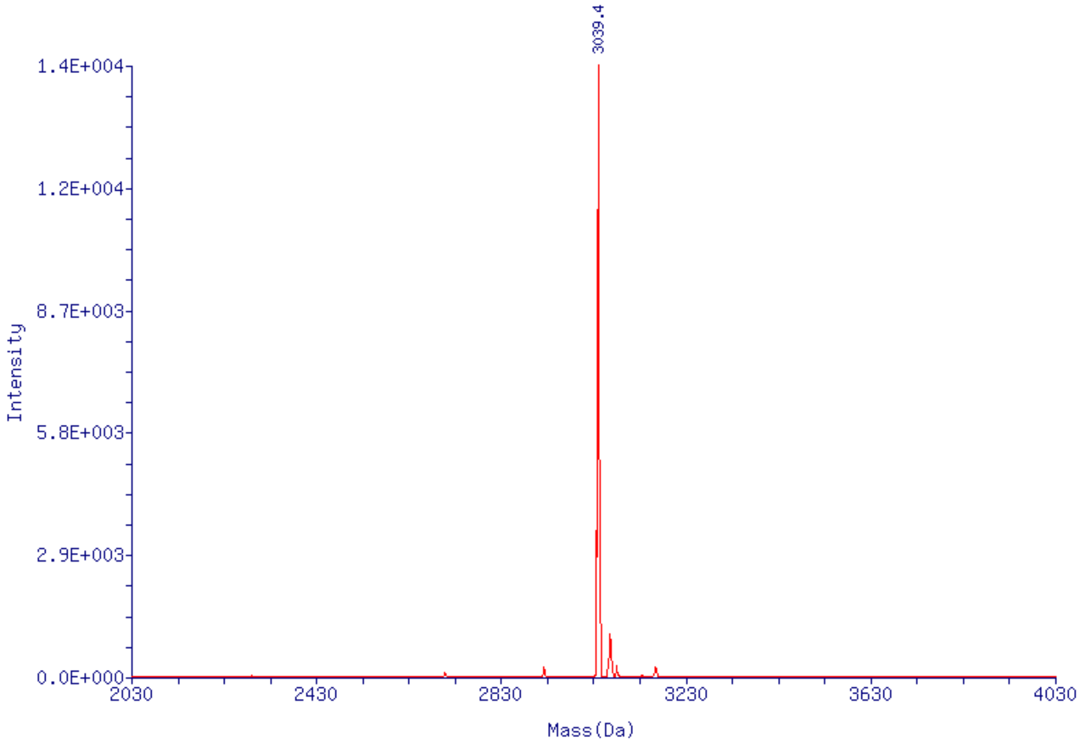

ODN4 GCAG**X**GTACG

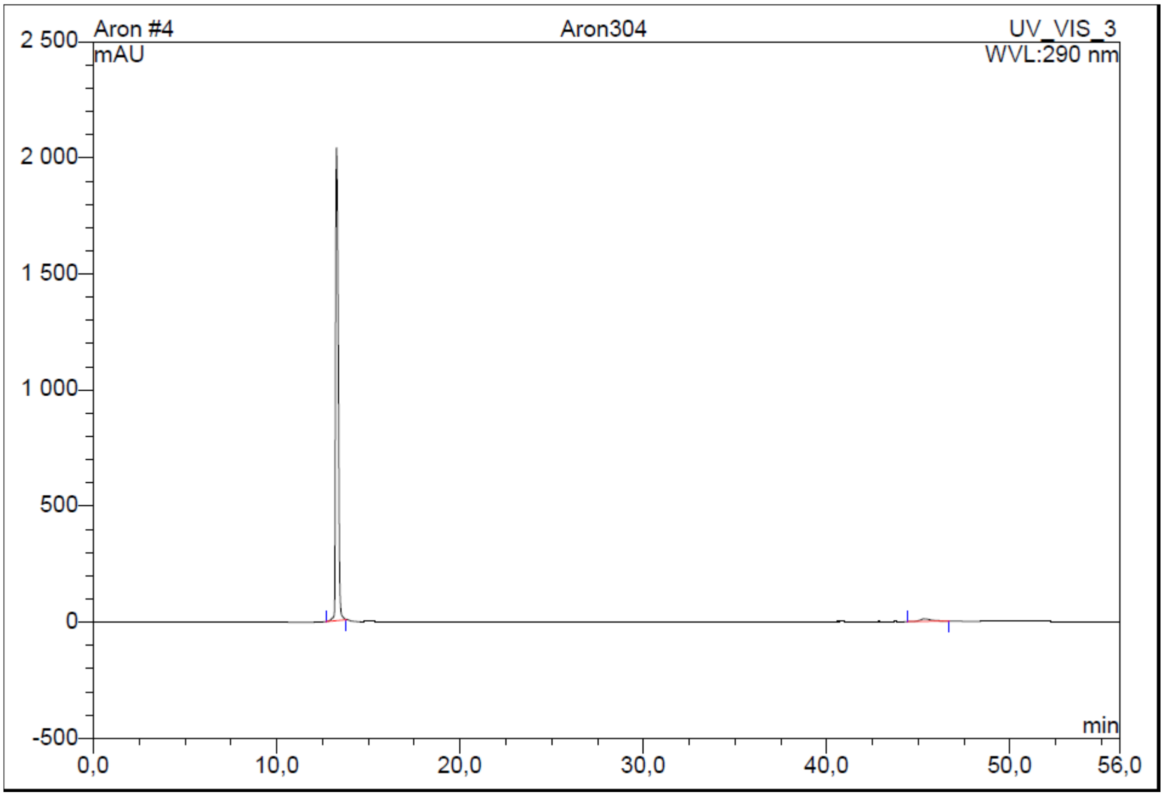

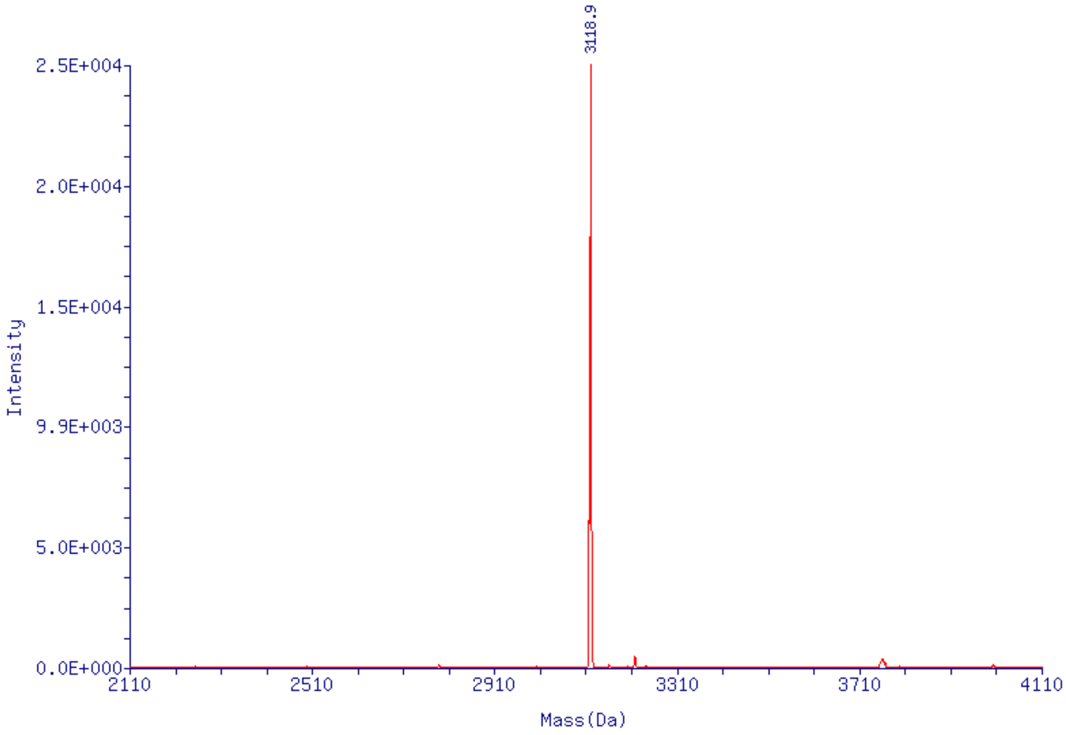

ODN5 GCAG**X**CTACG

LC/MS Chromatogram of 2:

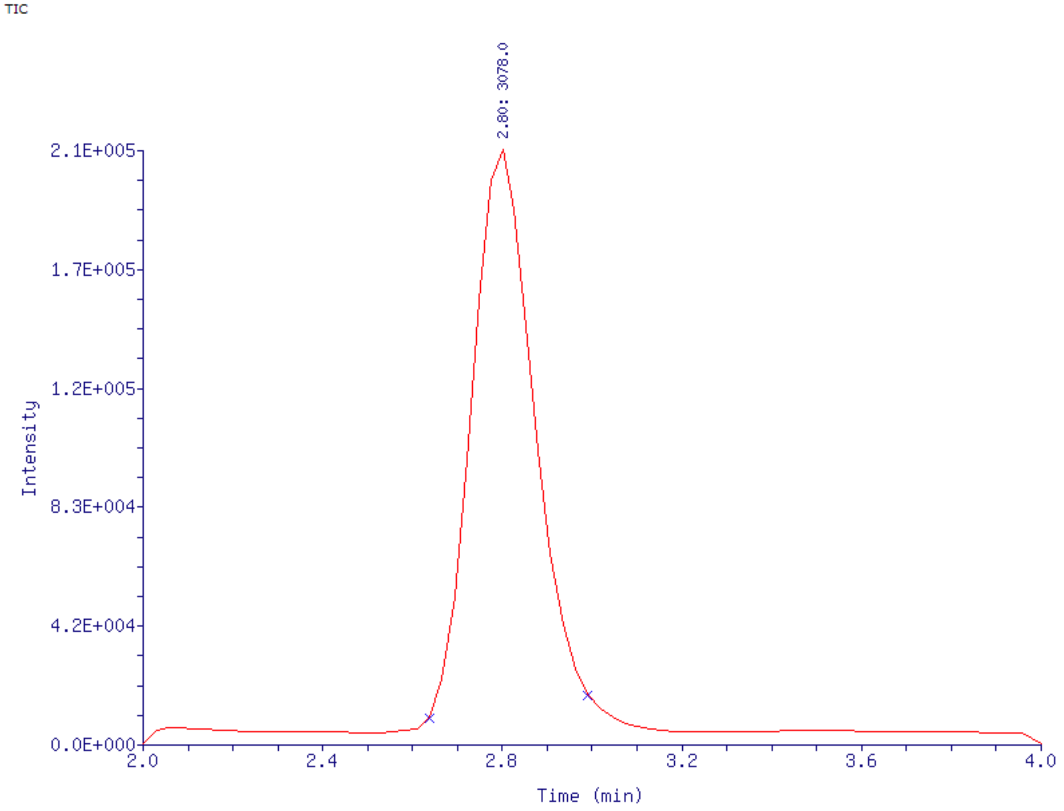

[<<] Mass Spectrum of 2:

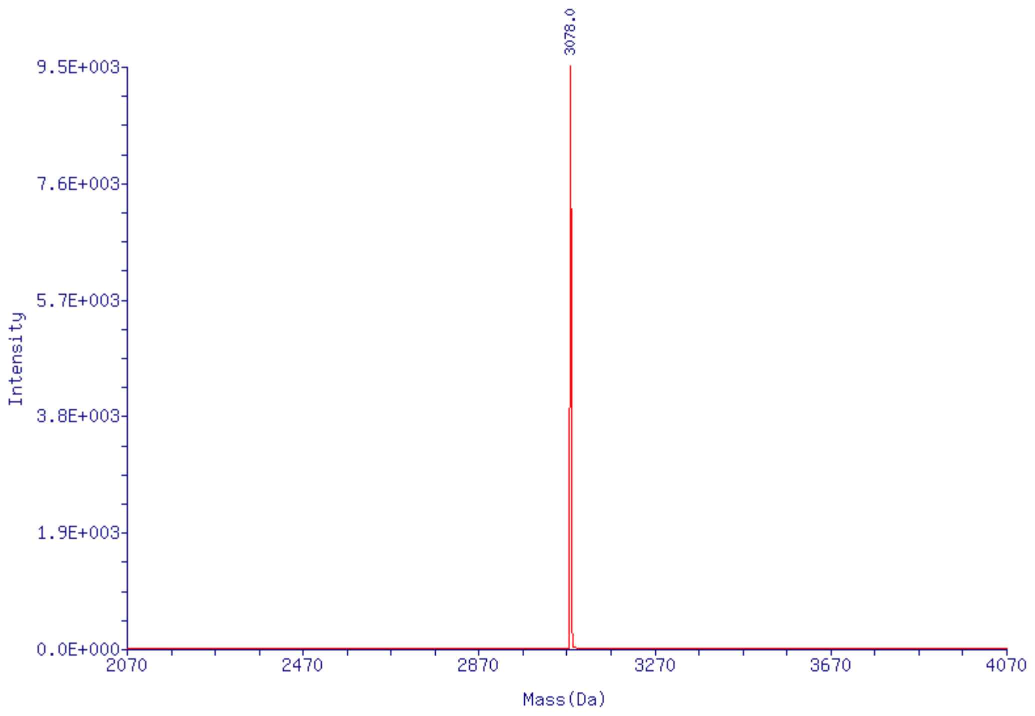

ODN6 GCAG**X**TTACG

[<<] [Top] [LC/UV]

LC/MS Chromatogram of 9:

TIC

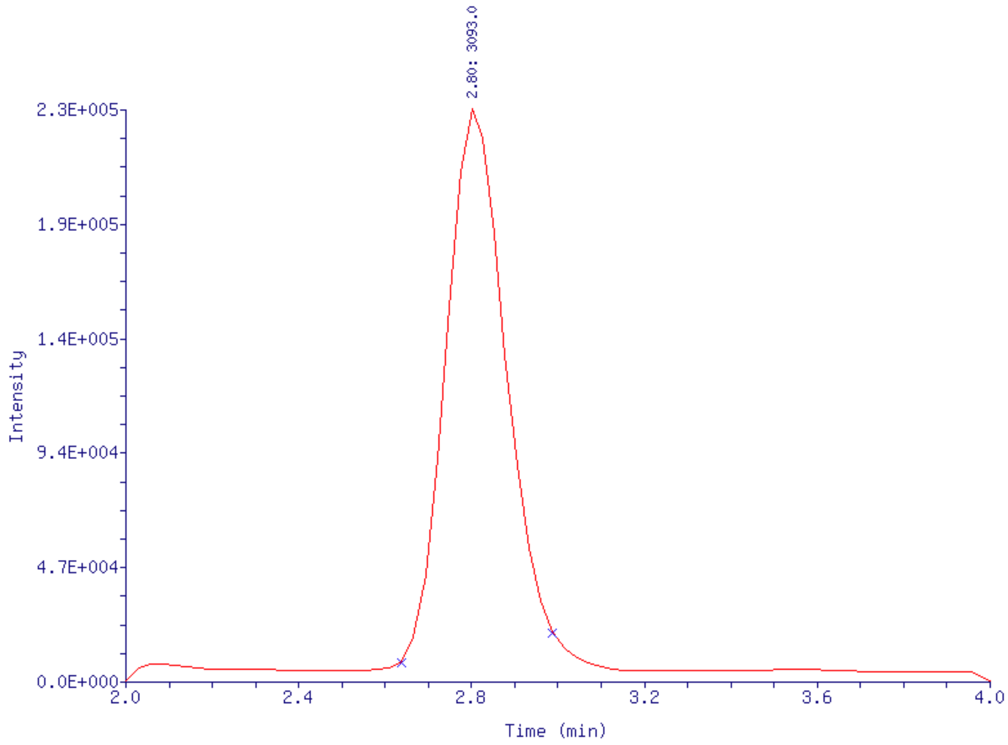

[<<] **Mass Spectrum of 9:**

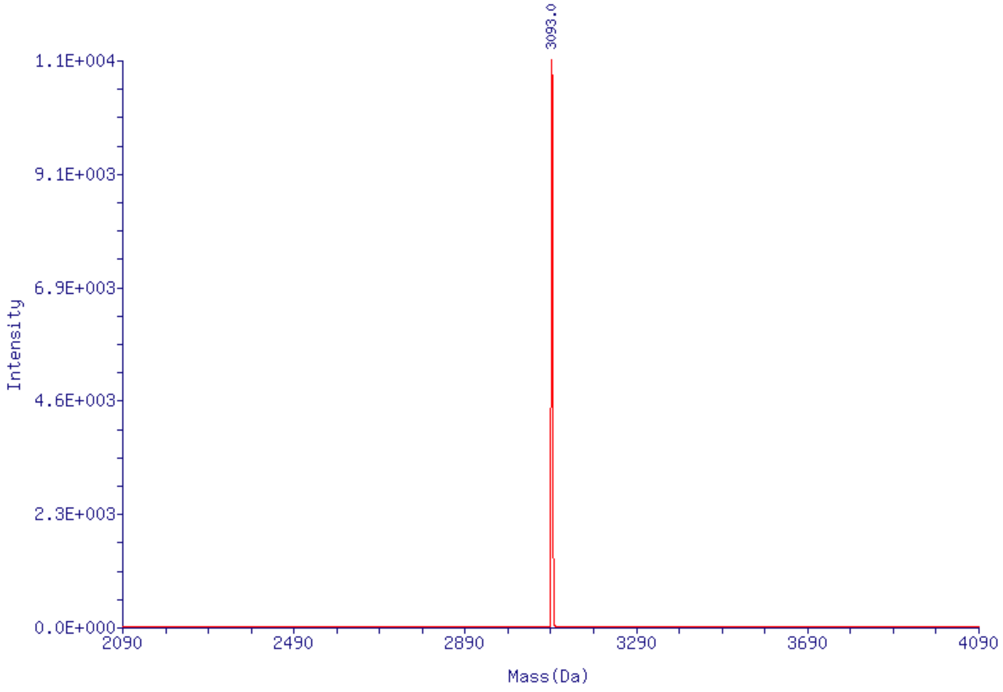

ODN7 GCAT**X**CTACG

[<<] [Top] [LC/UV]

LC/MS Chromatogram of 16:

TIC

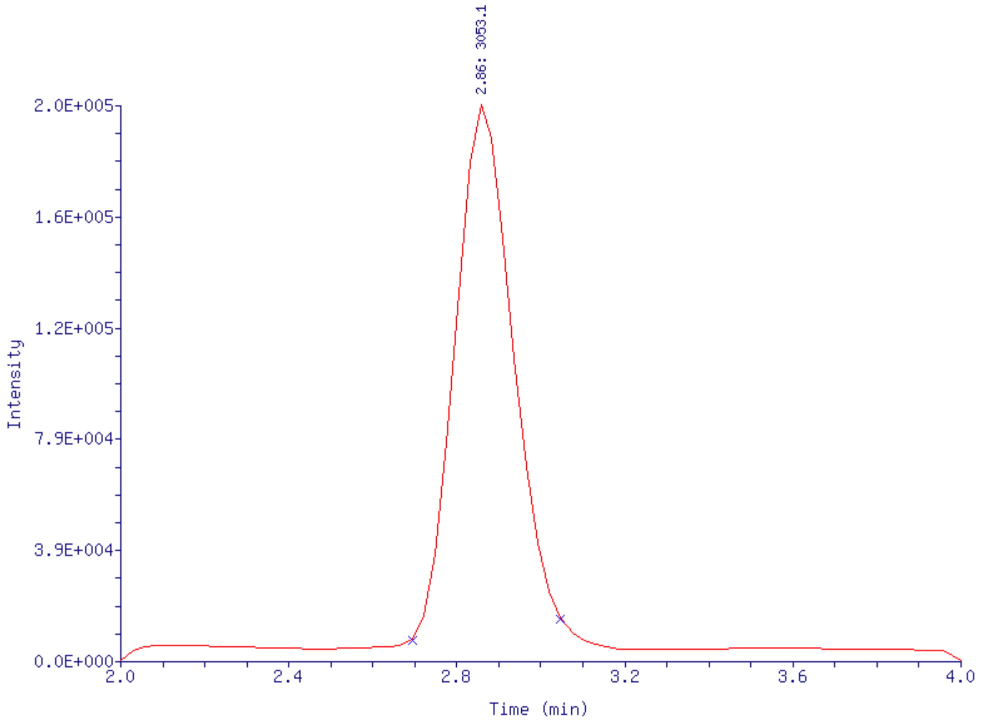

[<<] **Mass Spectrum of 16:**

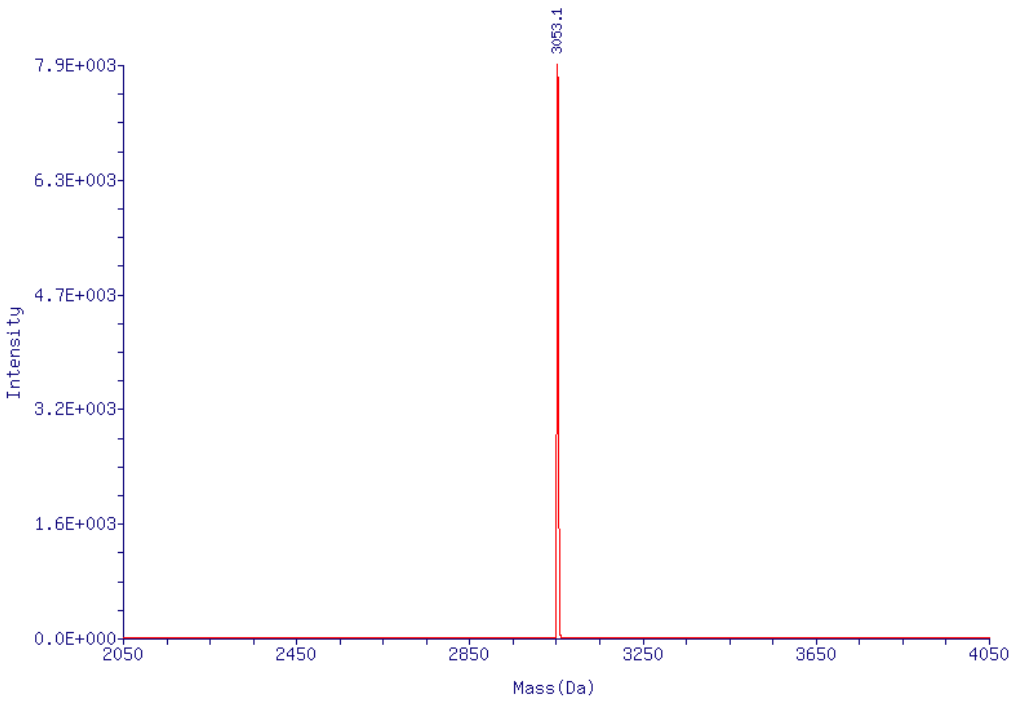

ODN8 GCAT**X**GTACG

[<<] [Top] [LC/UV]

LC/MS Chromatogram of 23:

TIC

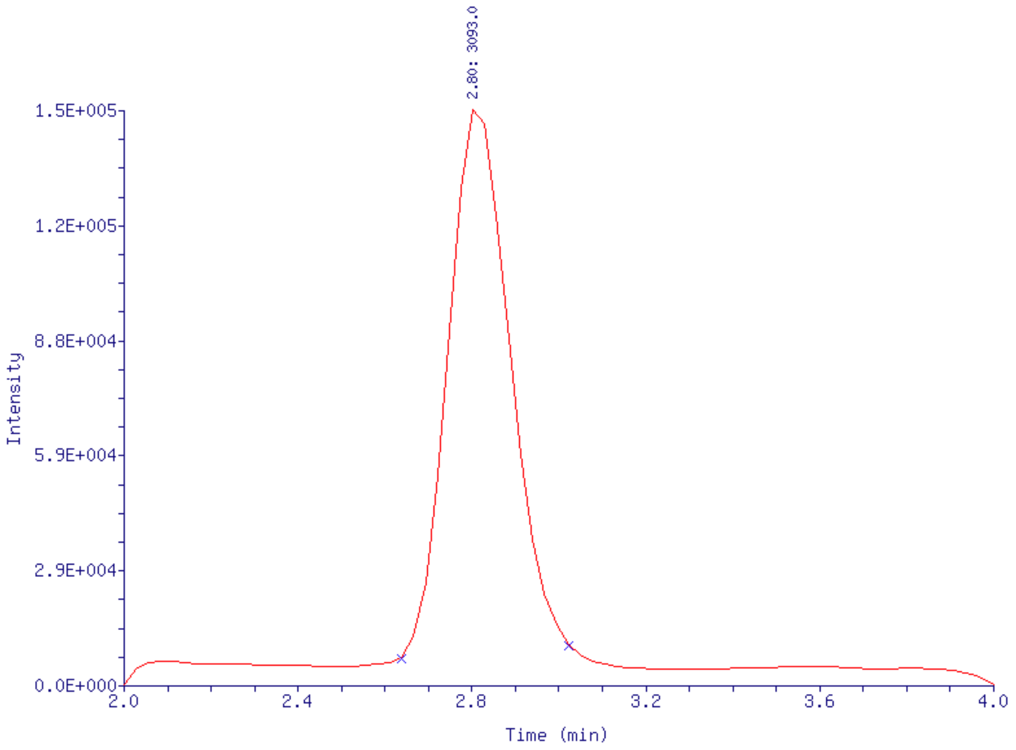

[<<] **Mass Spectrum of 23:**

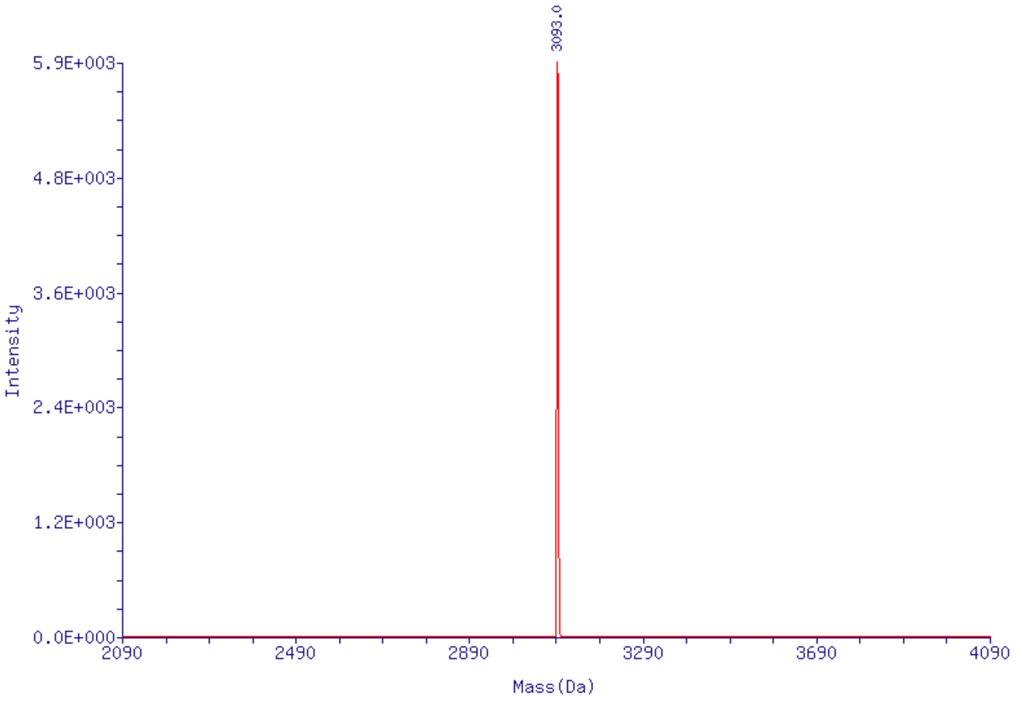

ODN9 GTAC**X**ACATG

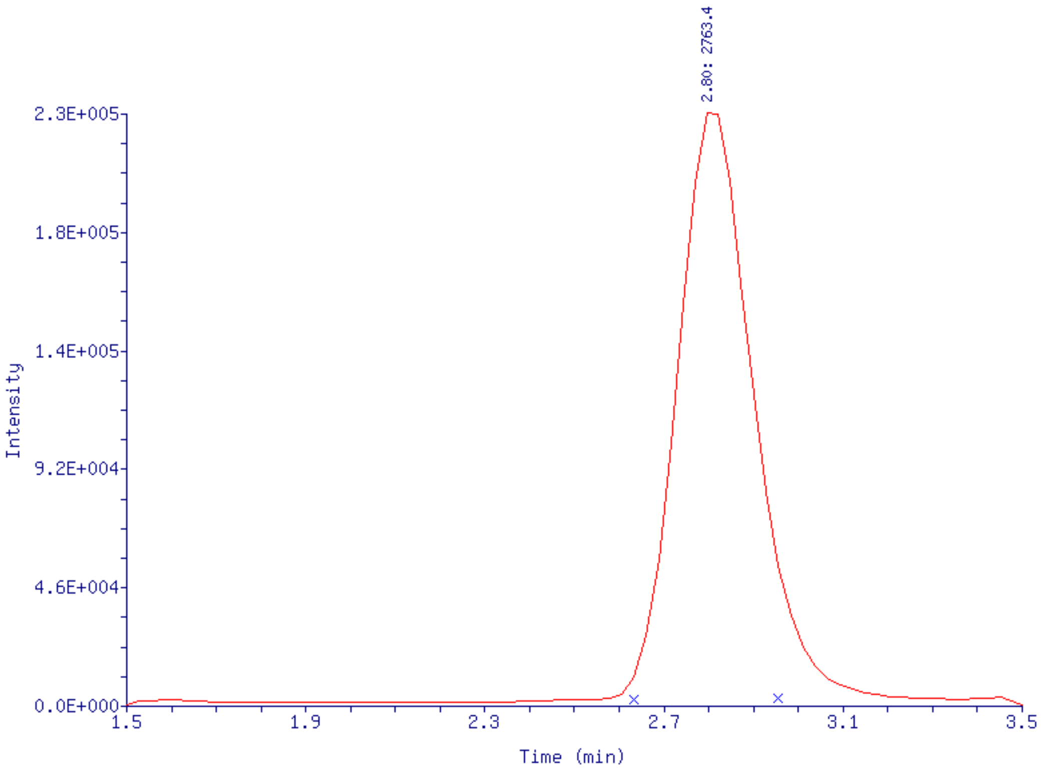

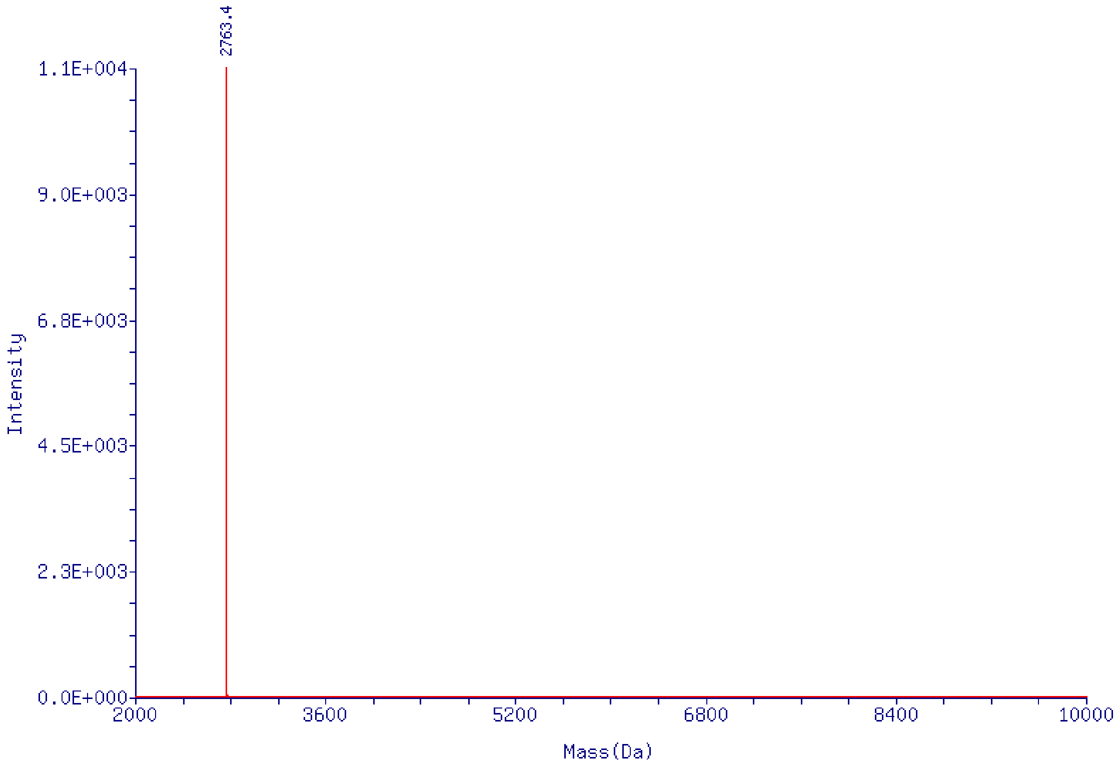

ODN12 GACTAT**X**CCCTATAGTGAGTCGTATTA

LC/MS Chromatogram of 26:

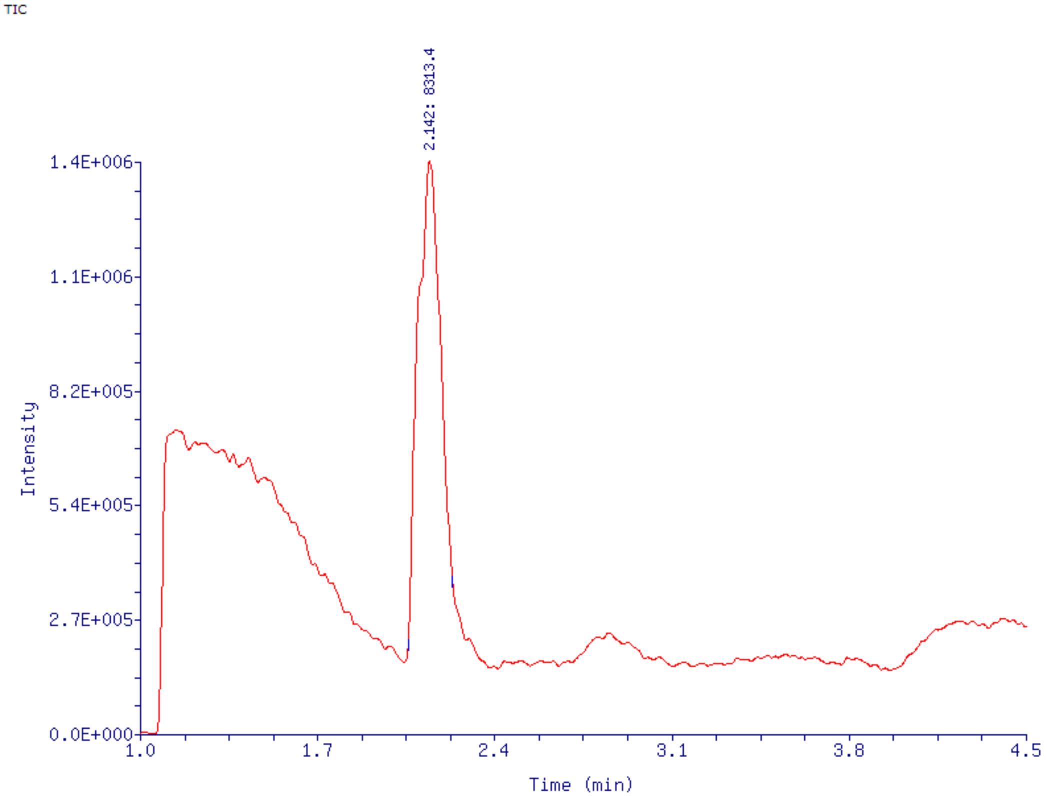

Deconvoluted Mass Spectrum of 26, RT = 2.142 min:

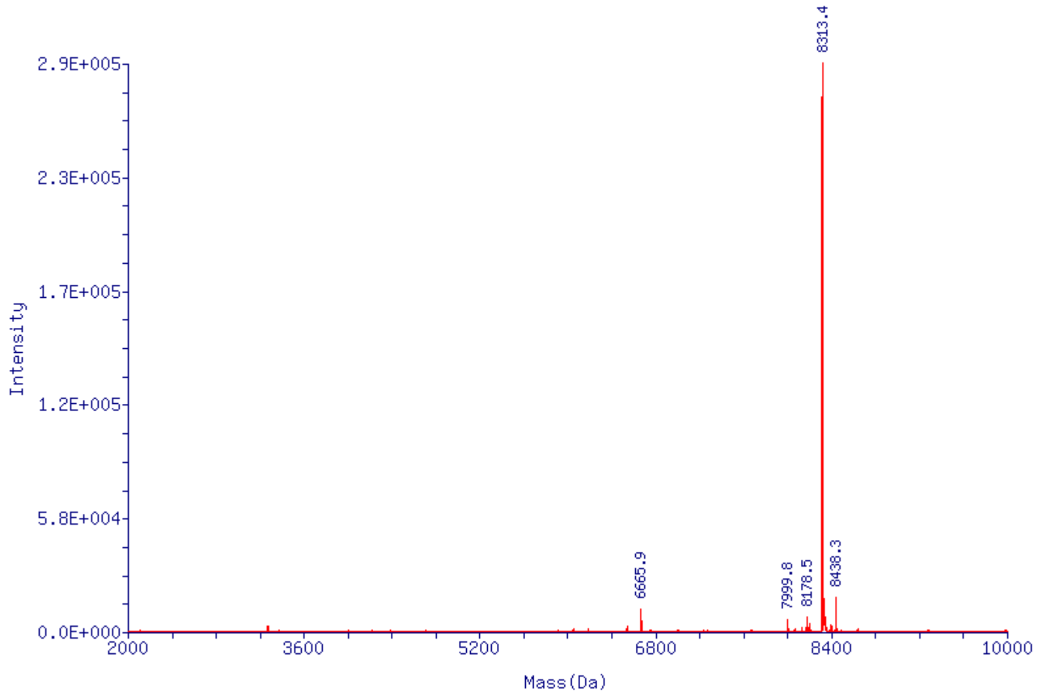

ODN13 Cy5-TAATACGACTCACTATAGGG

LC/MS Chromatogram of 7:

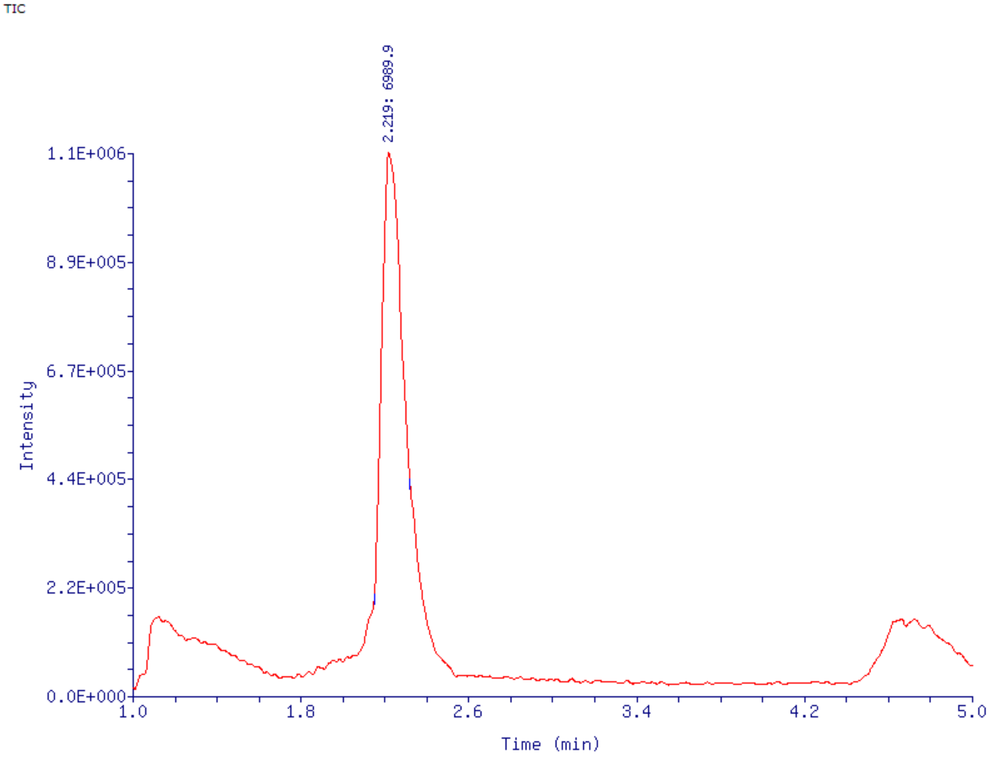

Deconvoluted Mass Spectrum of 7, RT = 2.219 min:

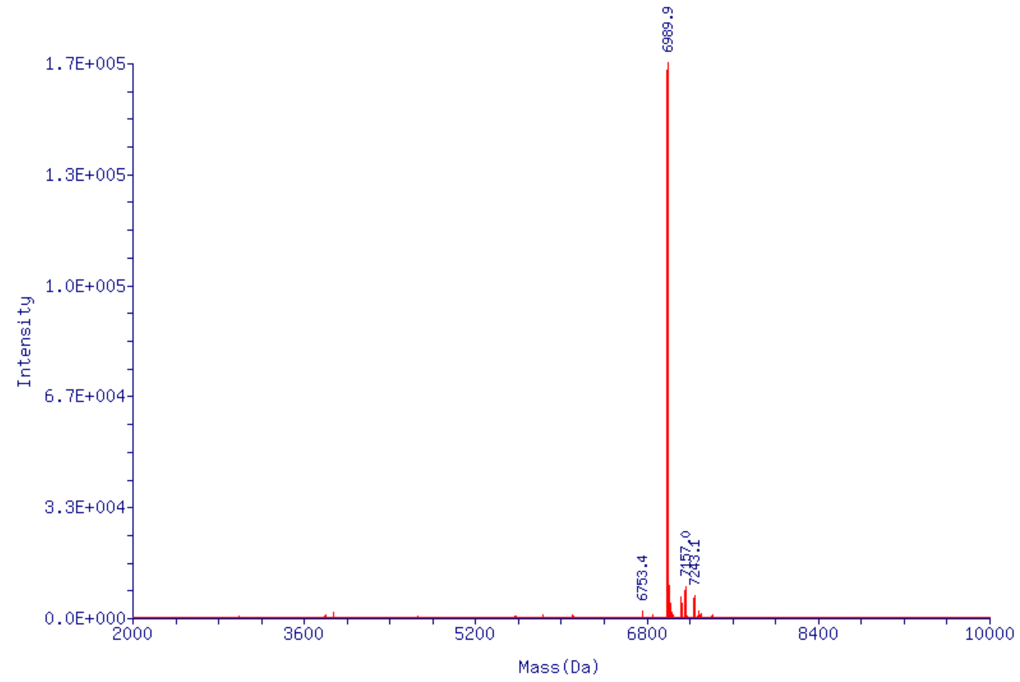

ODN14 GAAGACCA**X**AGCGTCC

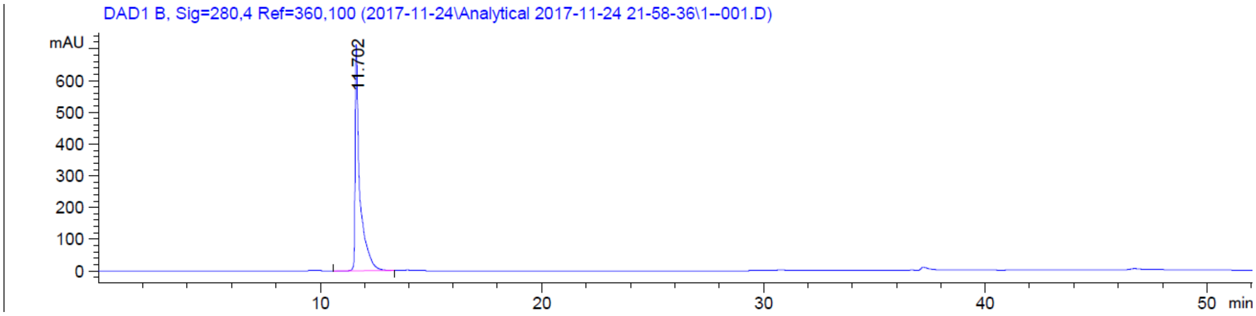

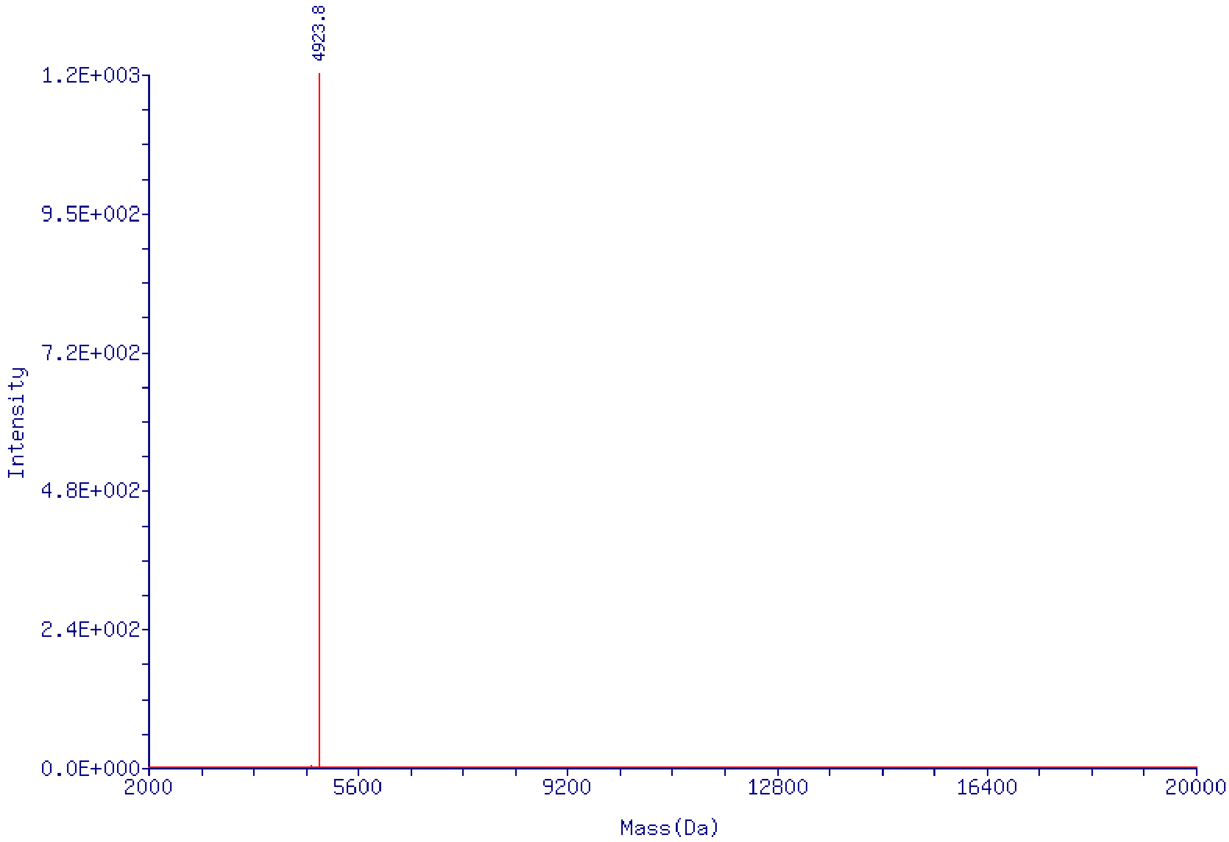

ODN15 GAAGACCA**X**CGCGTCC

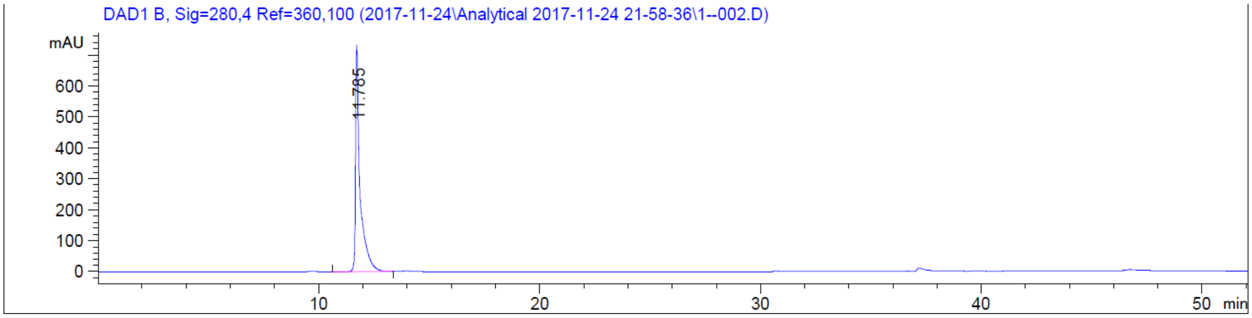

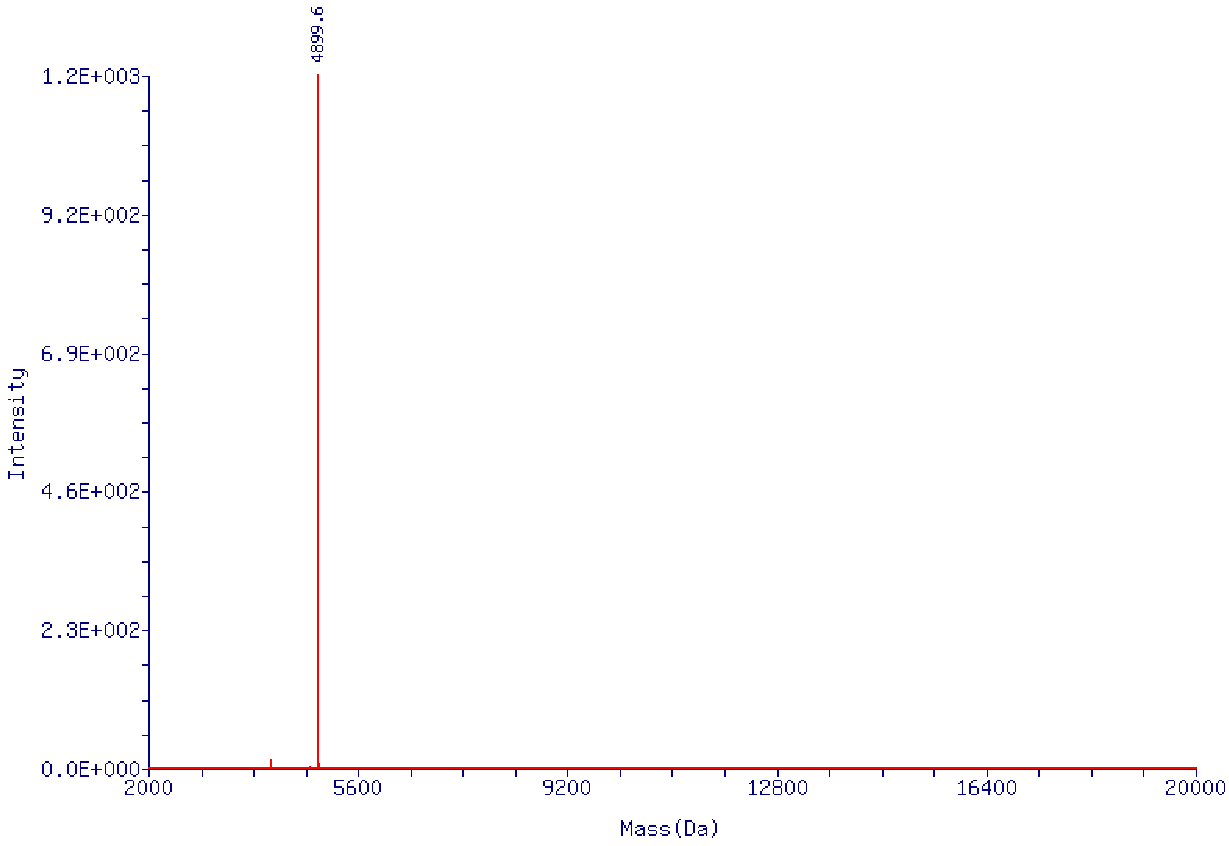

ODN16 GAAGACCA**X**GGCGTCC

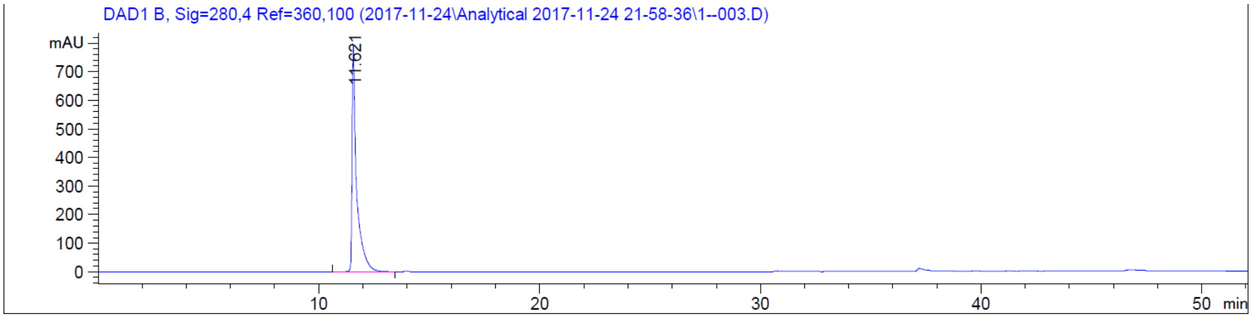

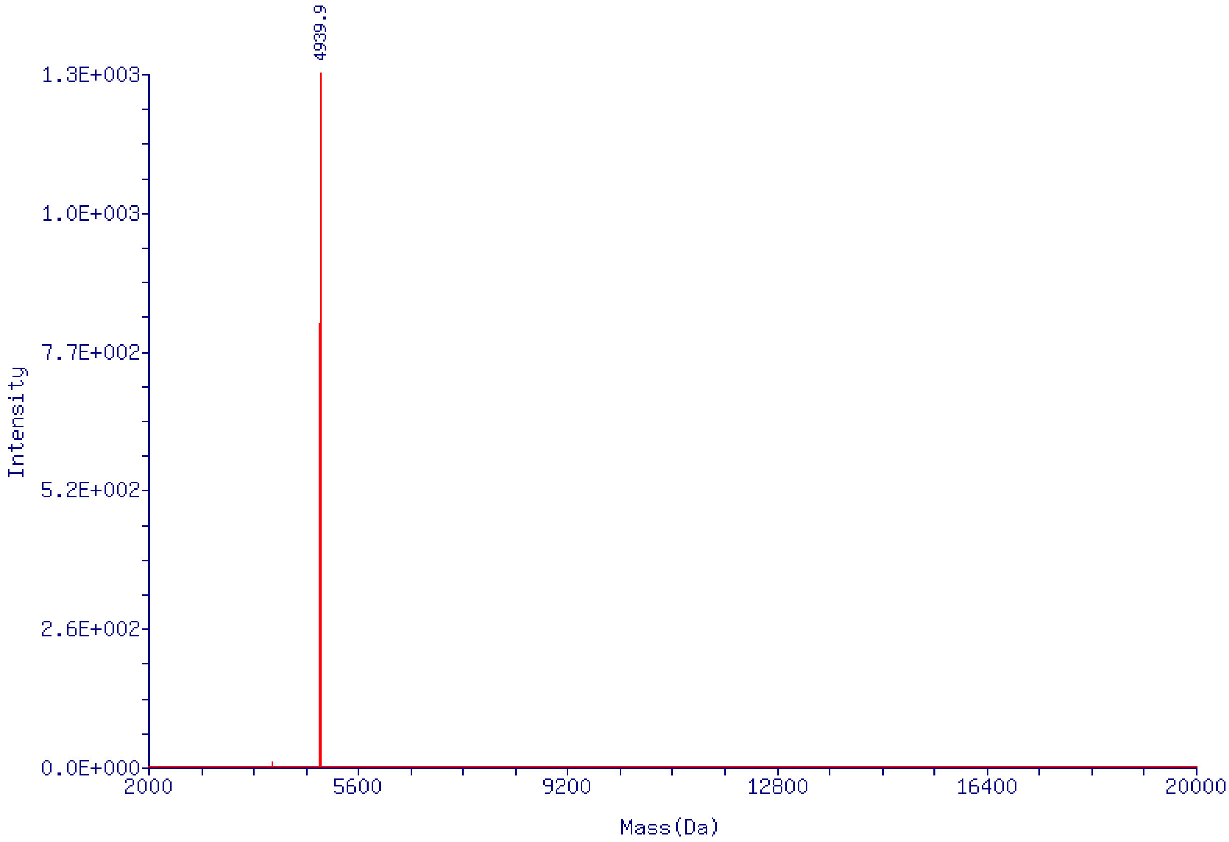

ODN17 GAAGACCA**X**TGCGTCC

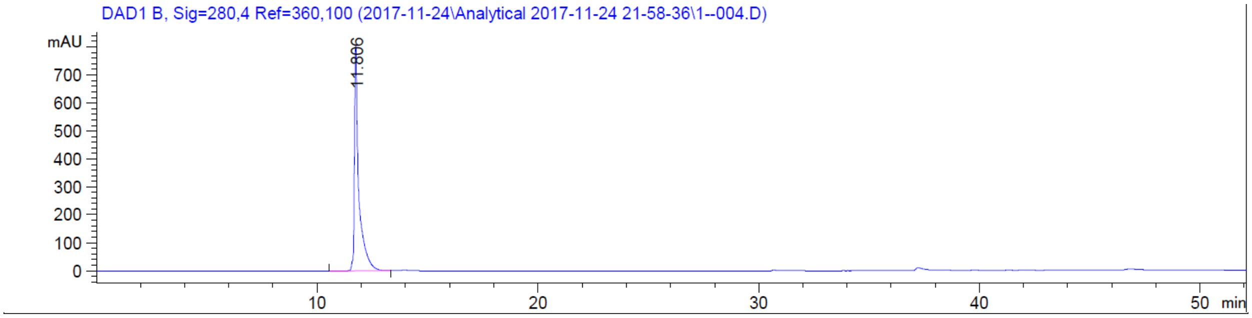

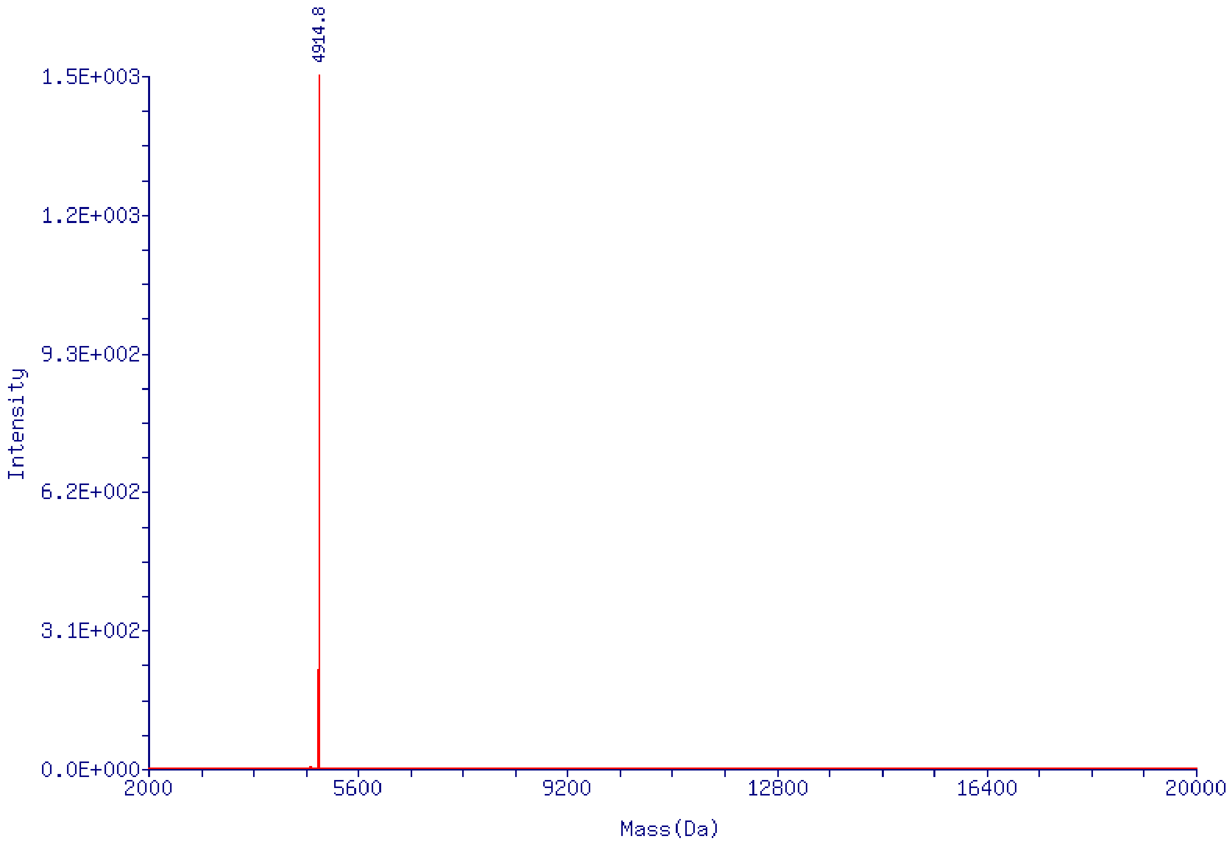

ODN18 GAAGACCC**X**AGCGTCC

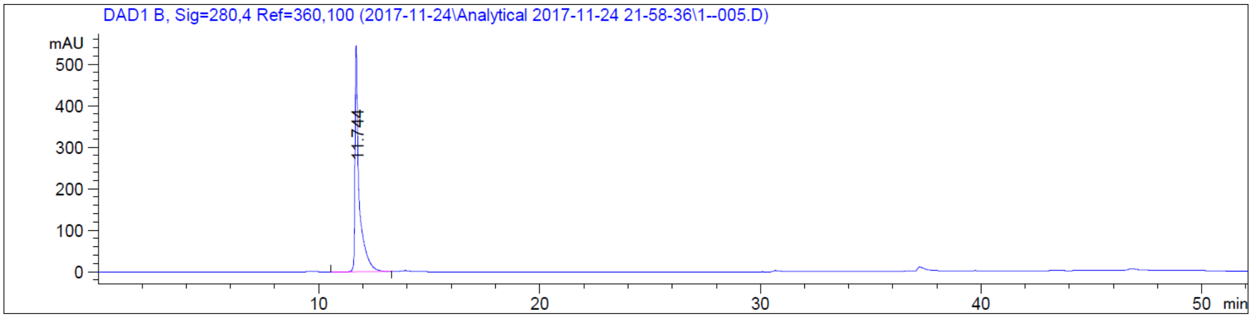

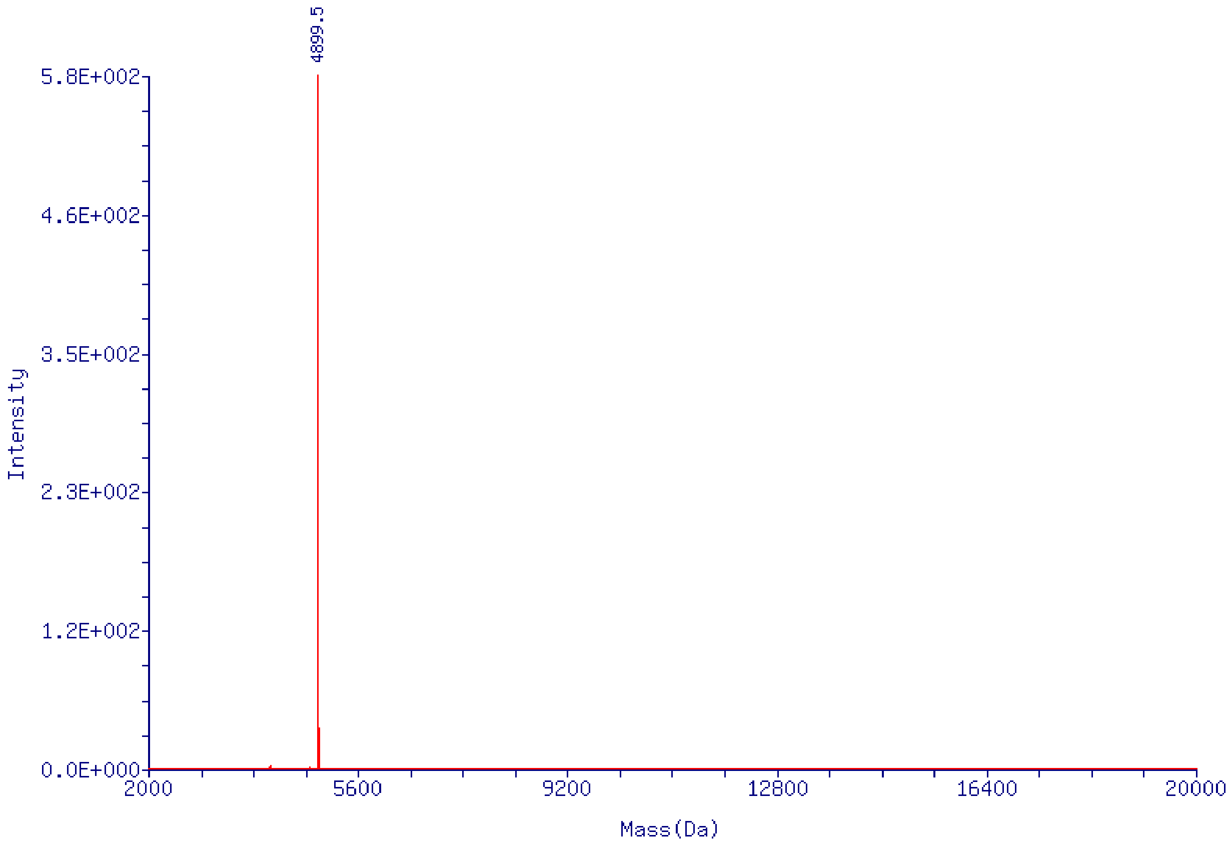

ODN19 GAAGACCC**X**CGCGTCC

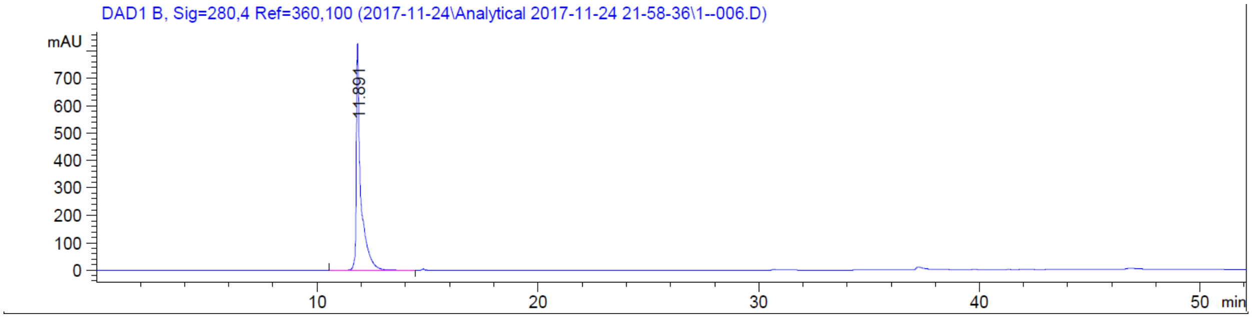

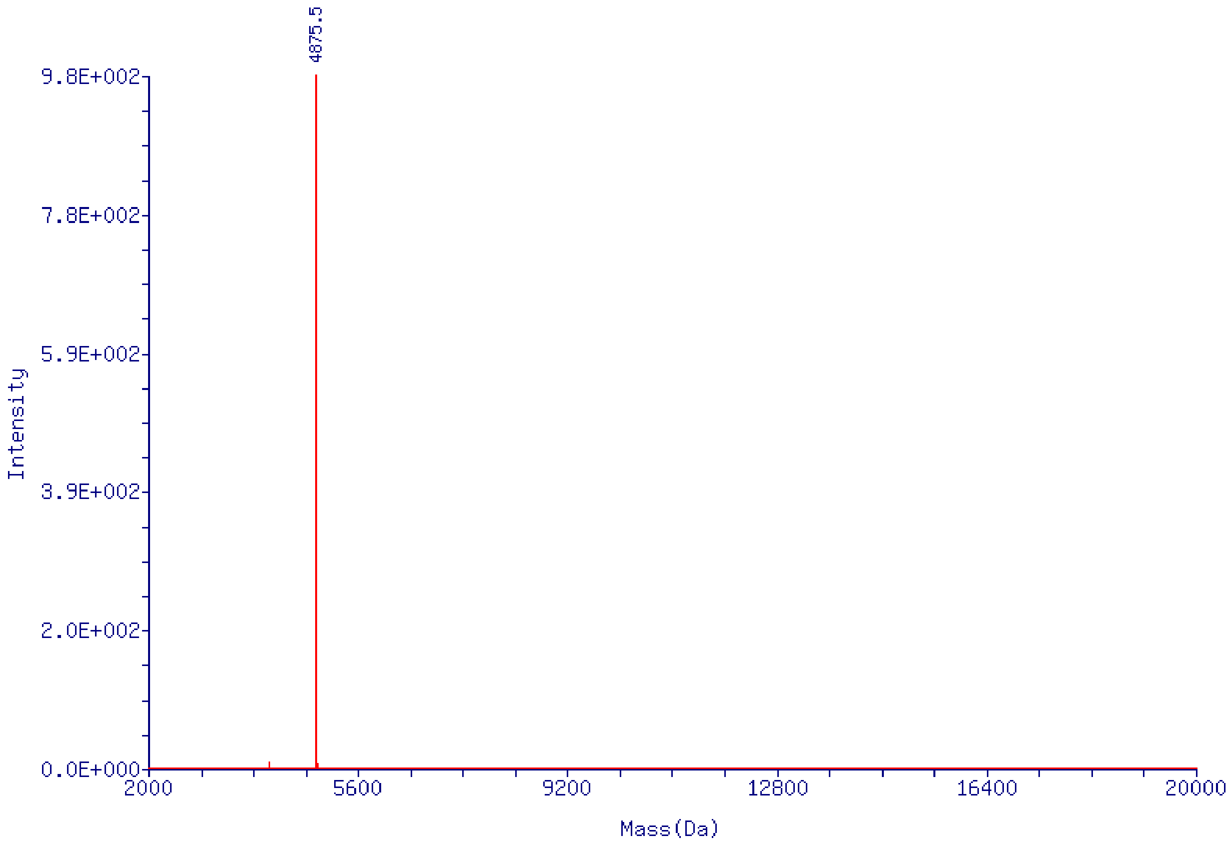

ODN20 GAAGACCC**X**GGCGTCC

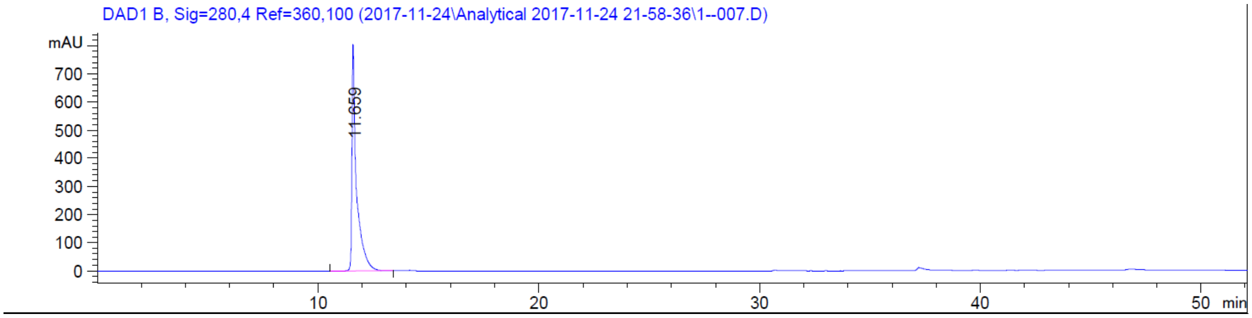

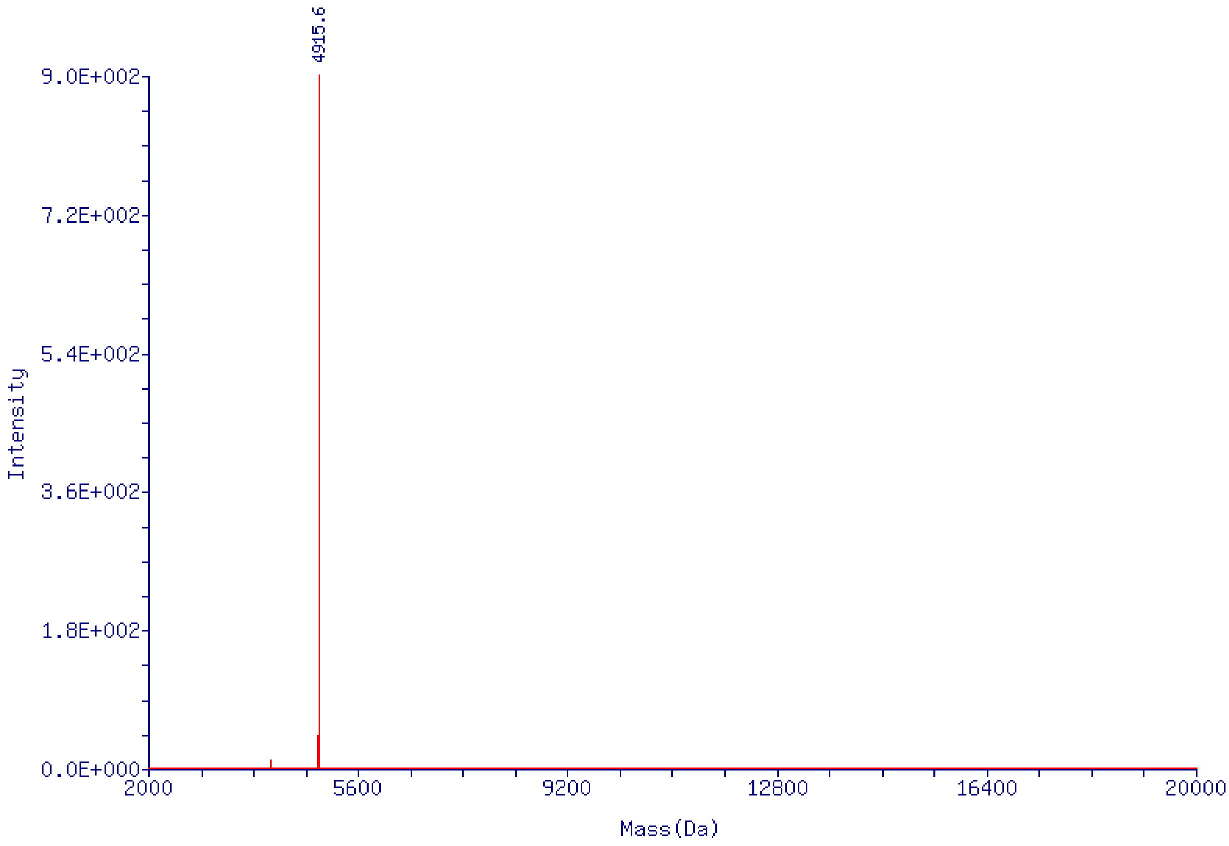

ODN21 GAAGACCC**X**TGCGTCC

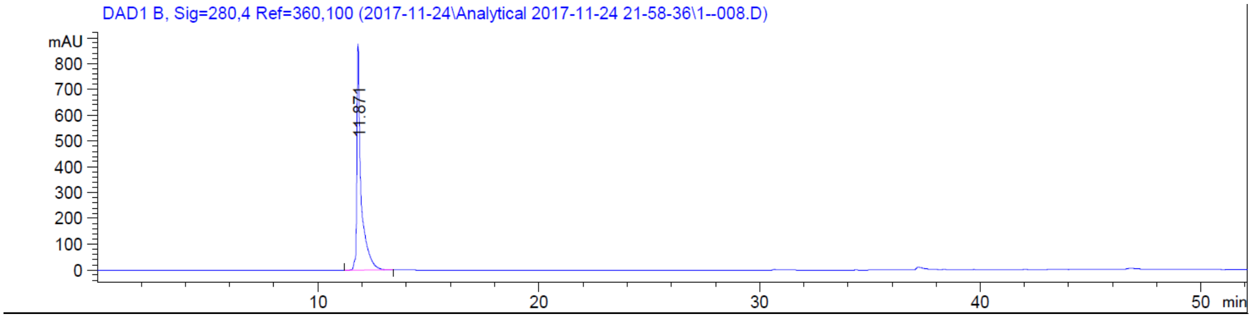

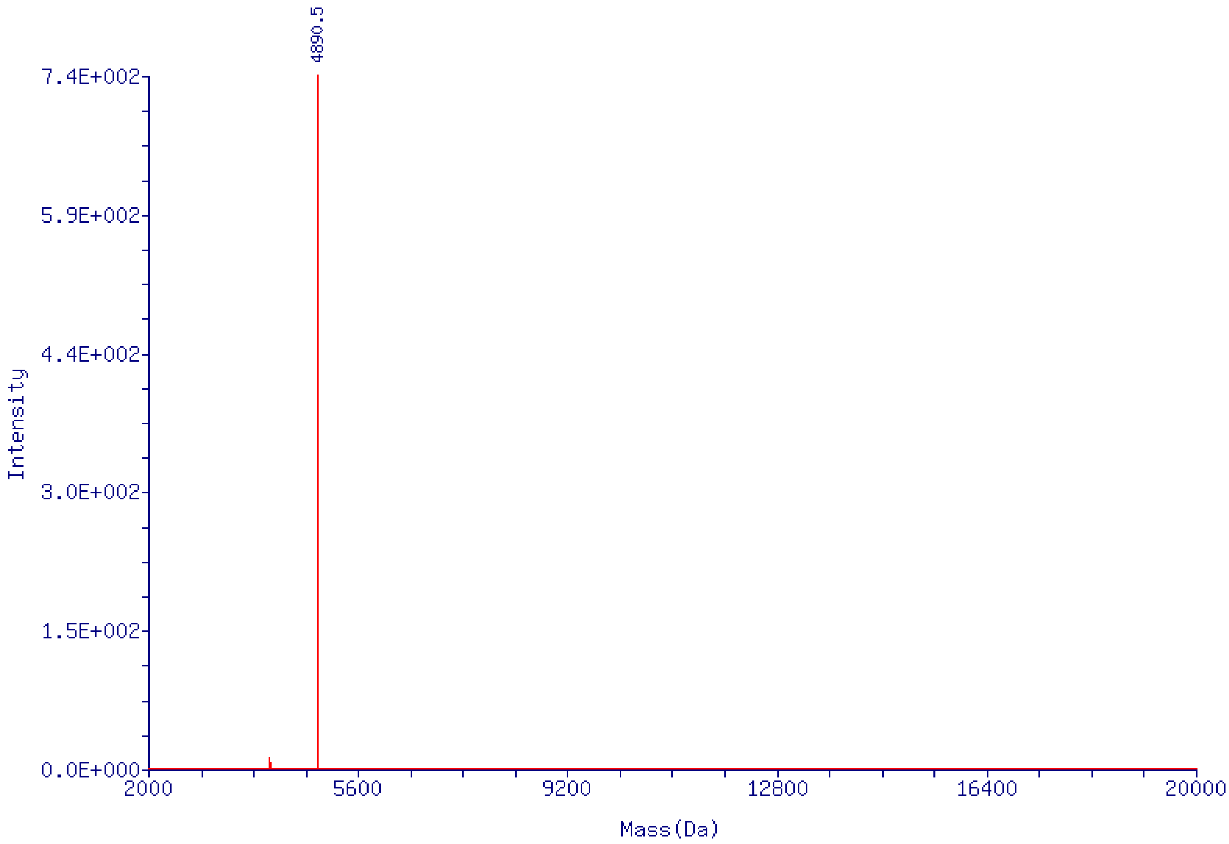

ODN22 GAAGACC**G**XAGCGTCC

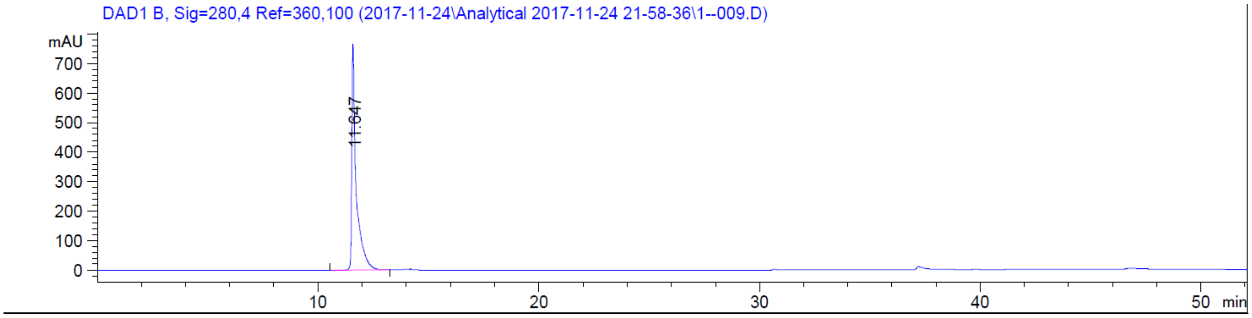

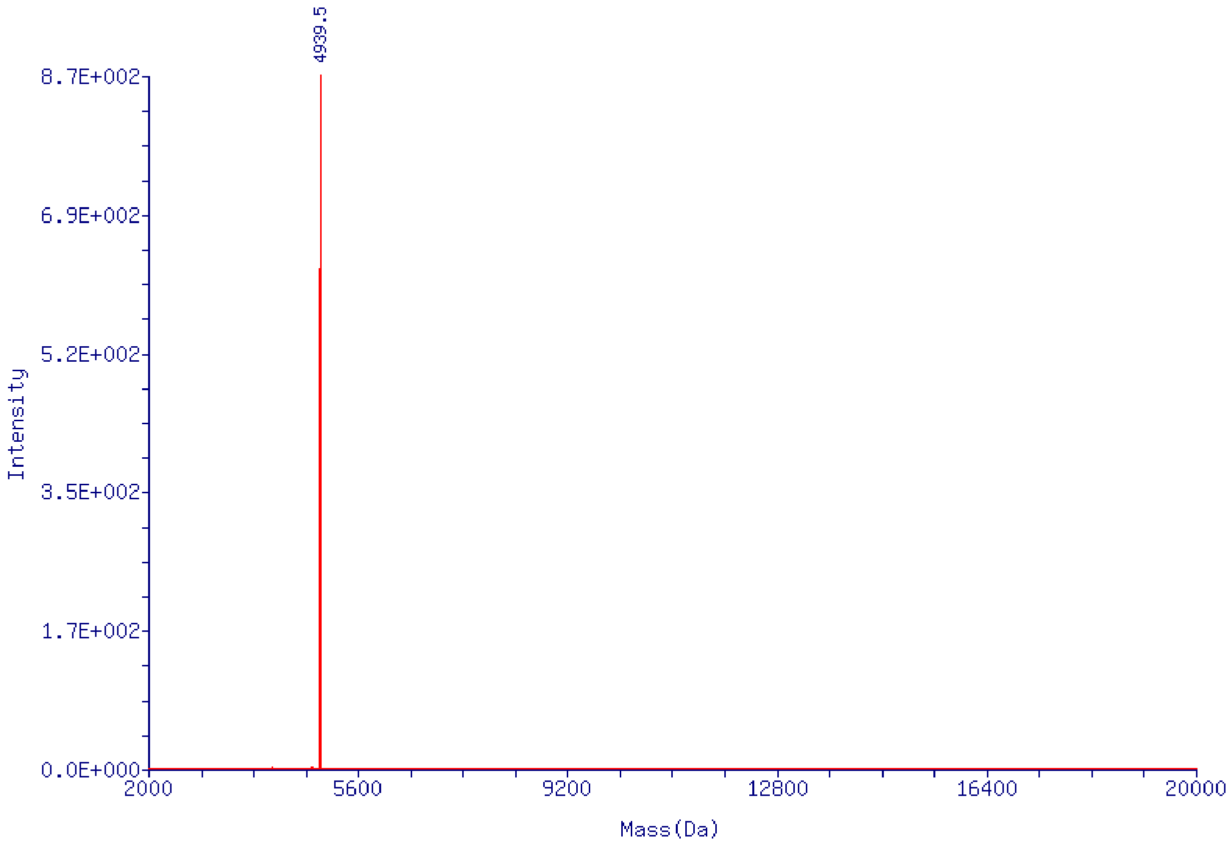

ODN23 GAAGACCG**X**CGCGTCC

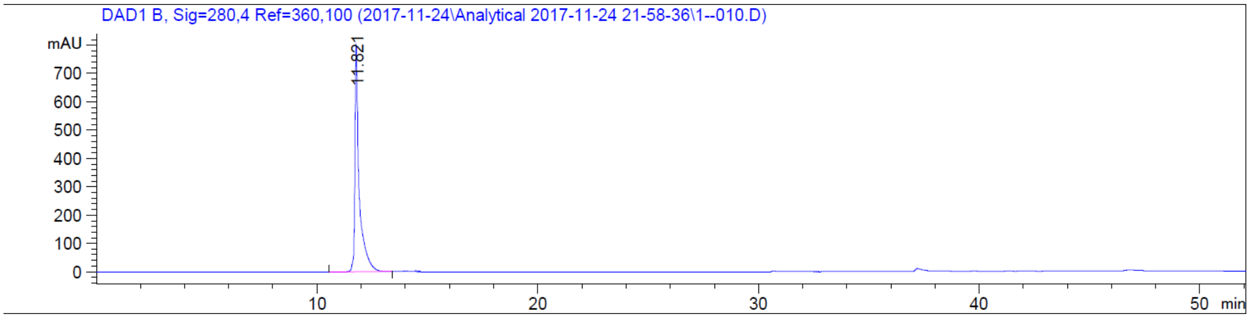

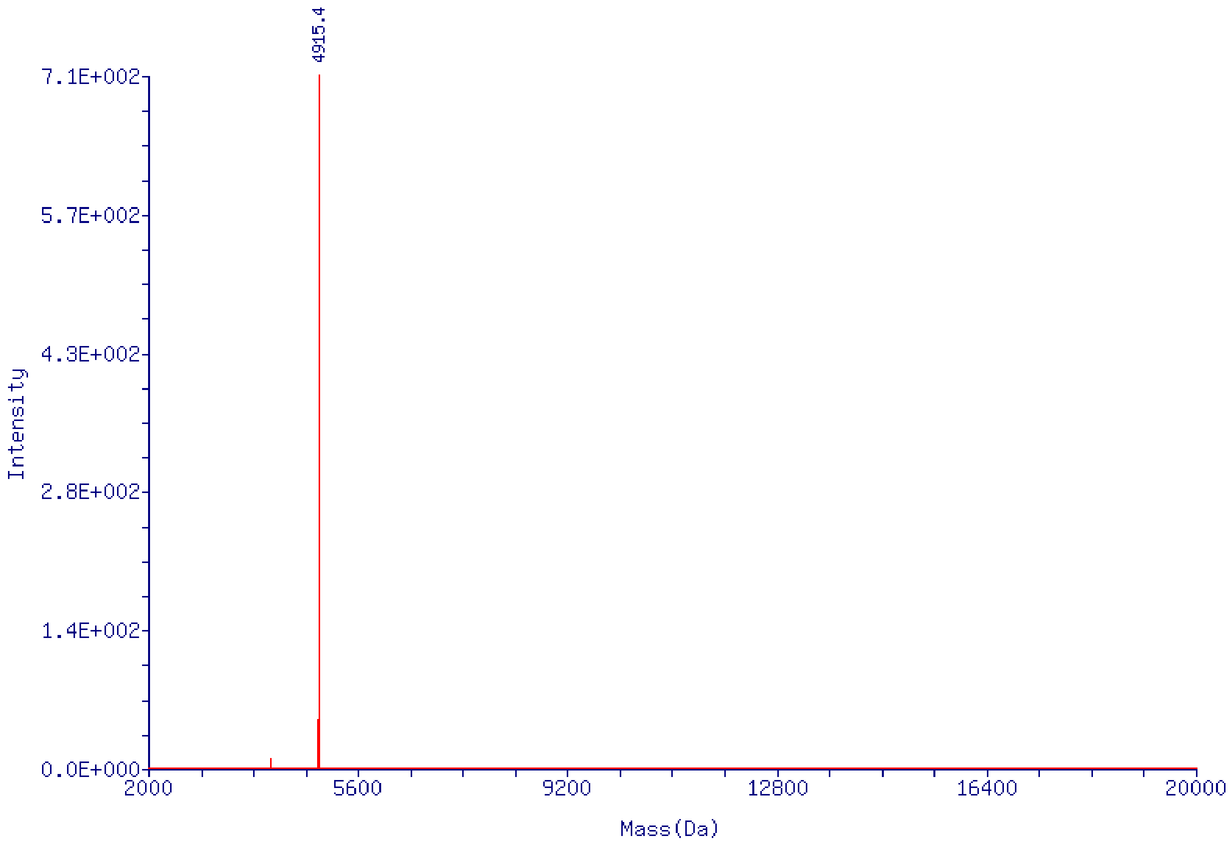

ODN24 GAAGACCG**X**GGCGTCC

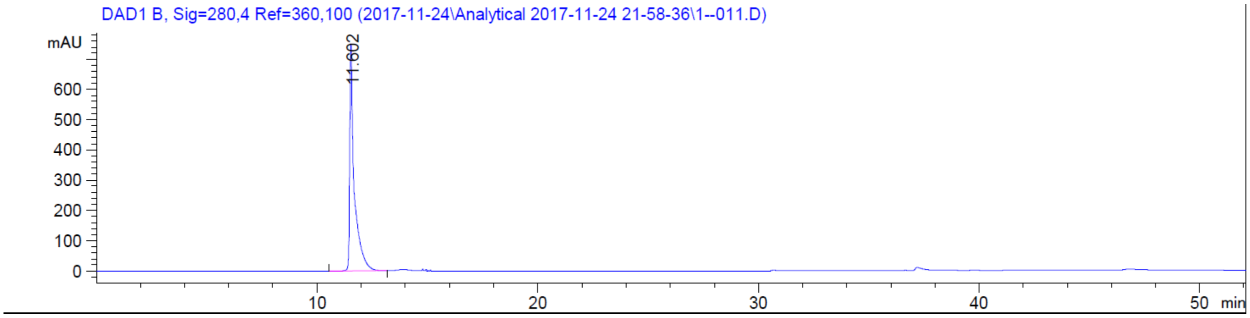

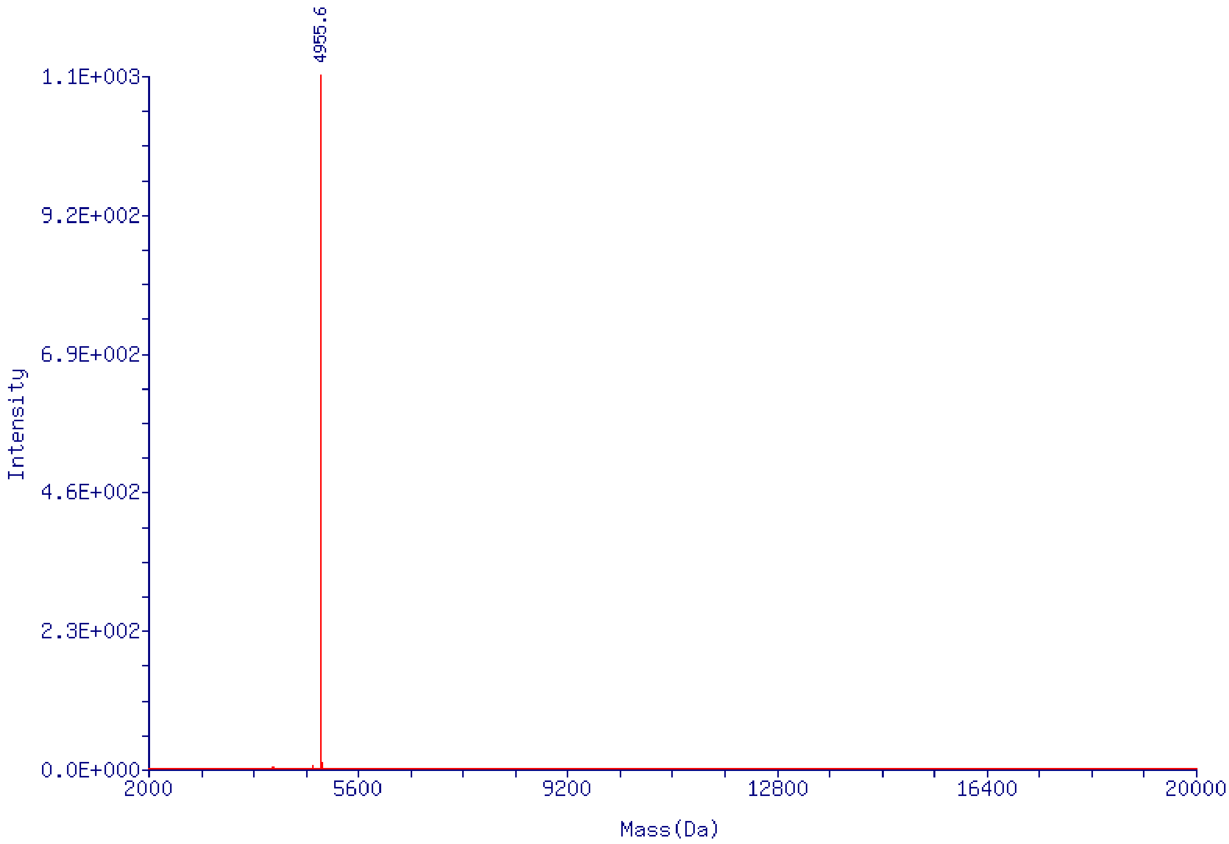

ODN25 GAAGACCG**X**TGCGTCC

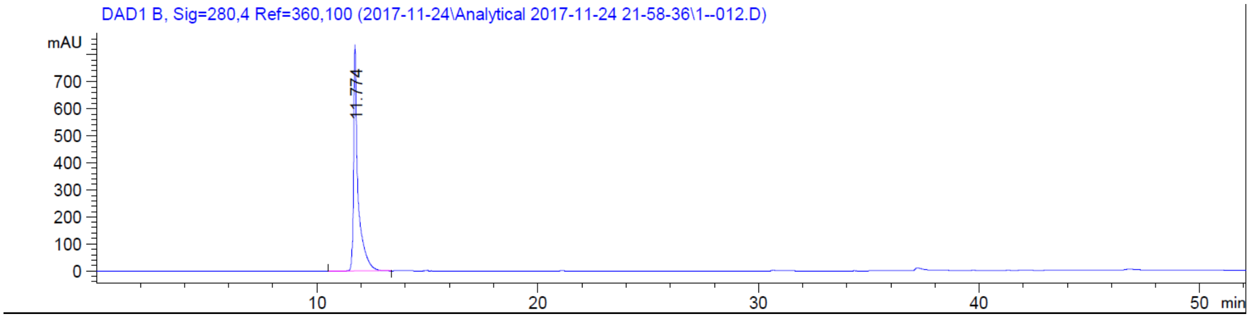

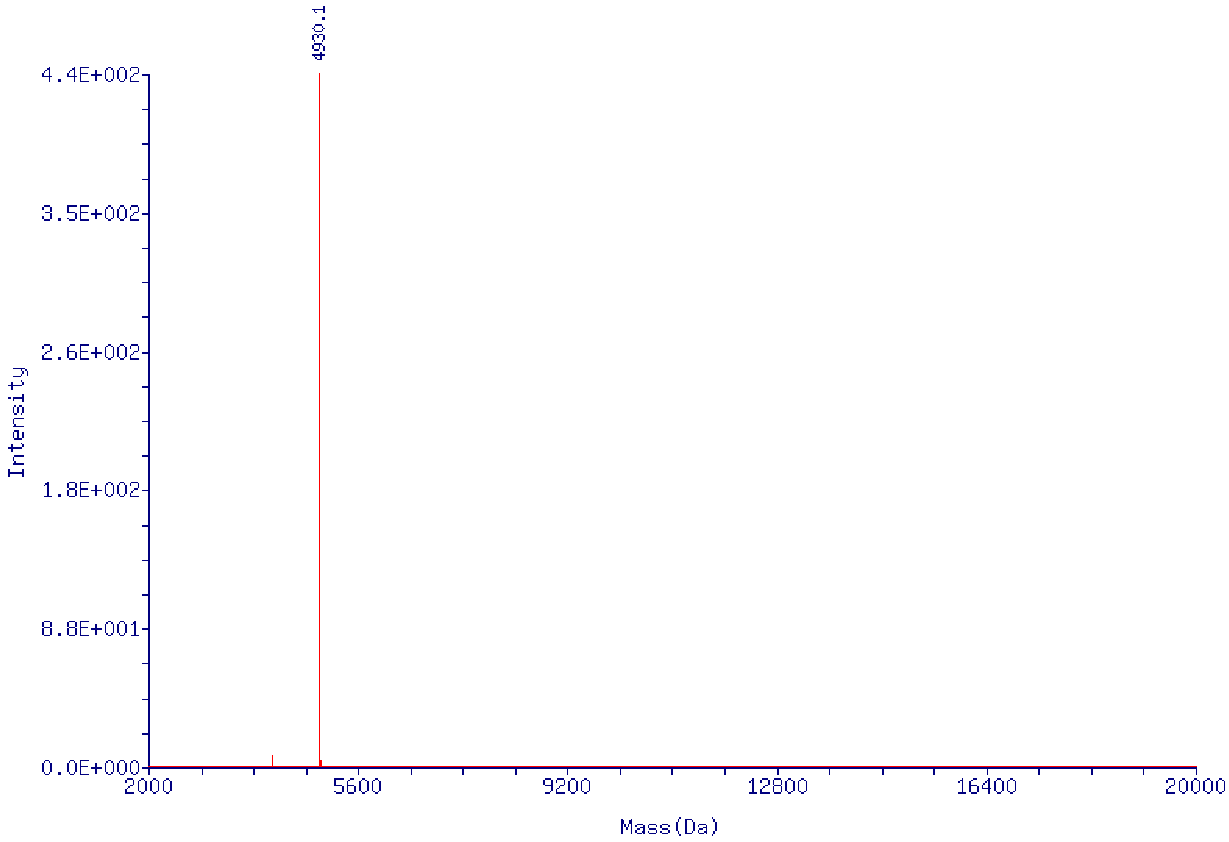

ODN26 GAAGACCT**X**AGCGTCC

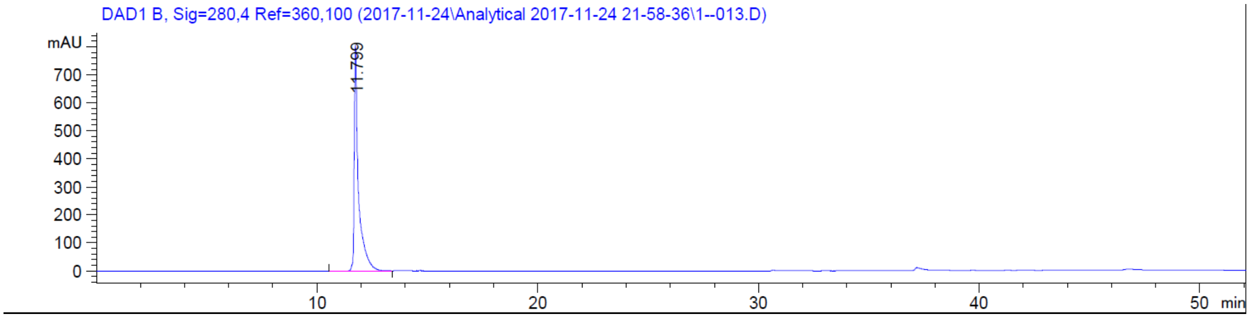

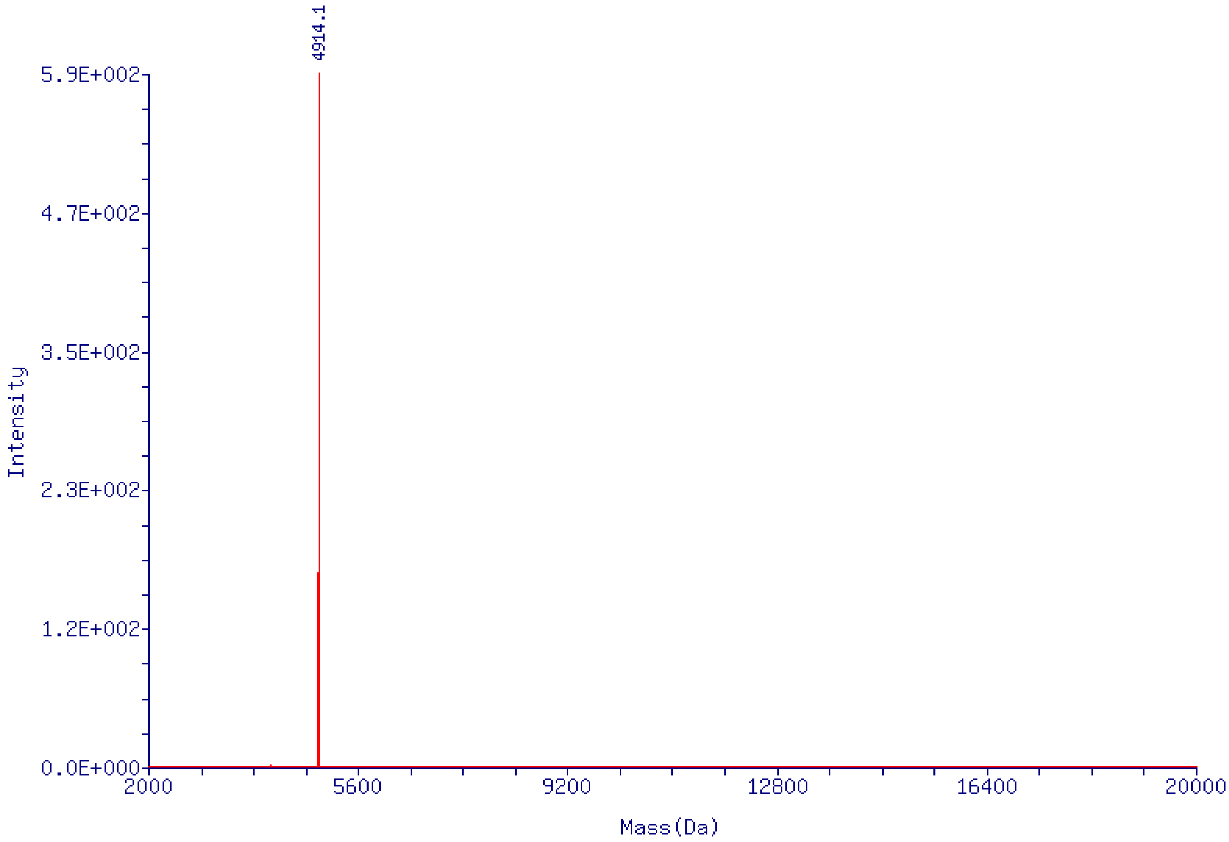

ODN27 GAAGACCT**X**CGCGTCC

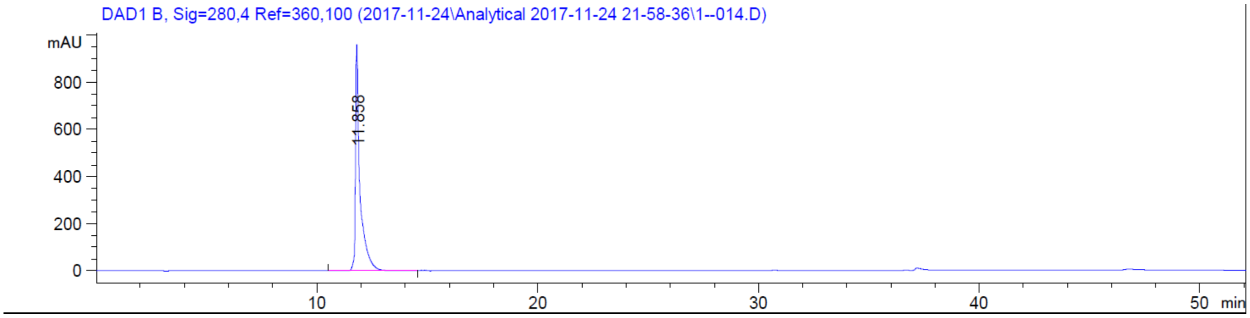

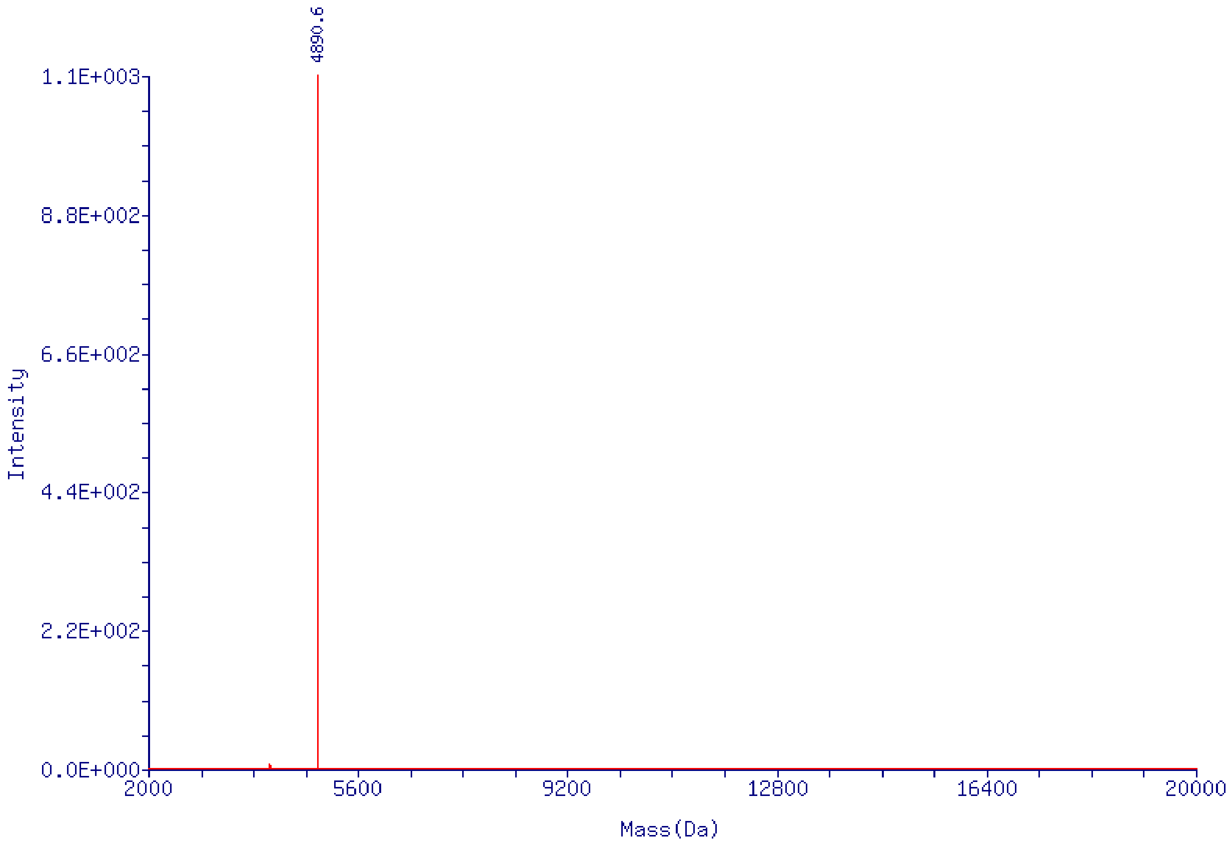

ODN28 GAAGACCT**X**GGCGTCC

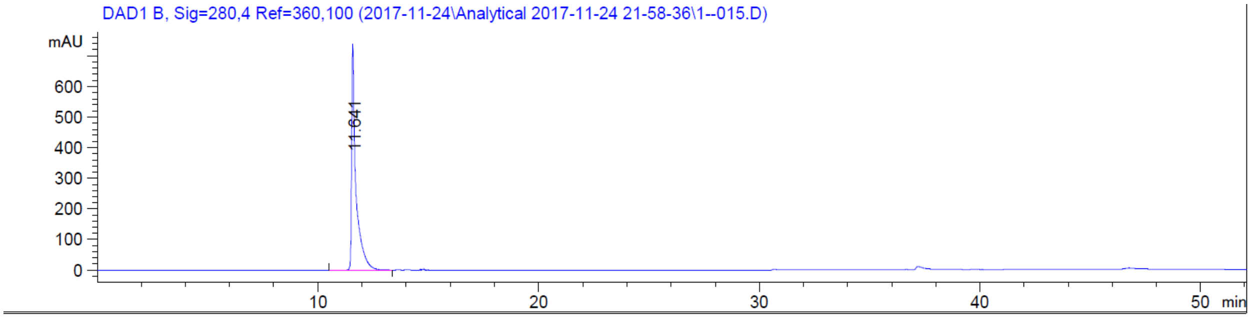

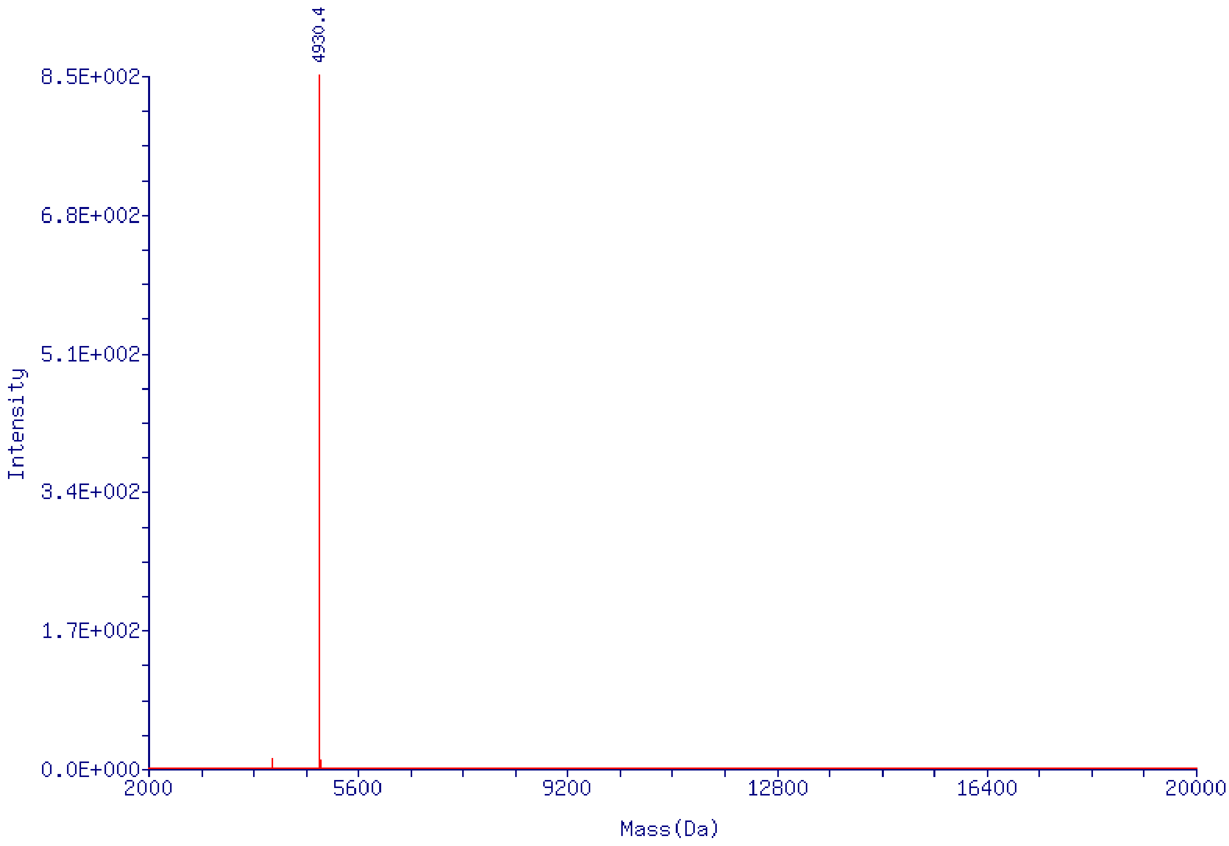

ODN29 GAAGACCT**X**TGCGTCC

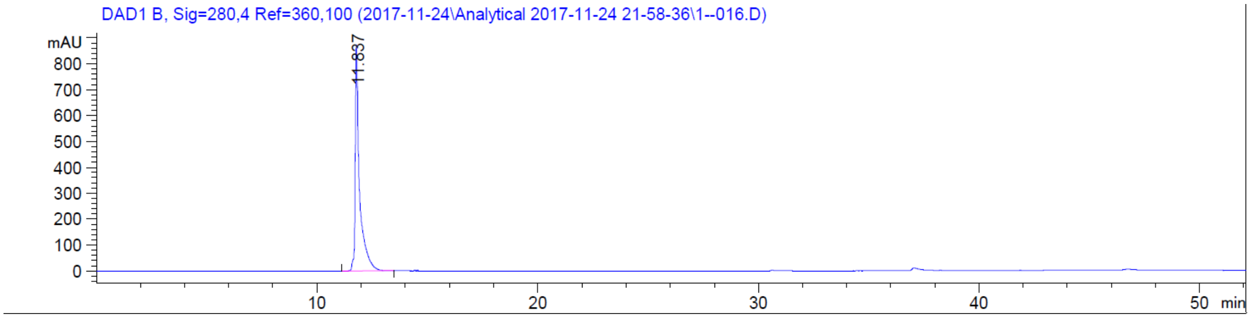

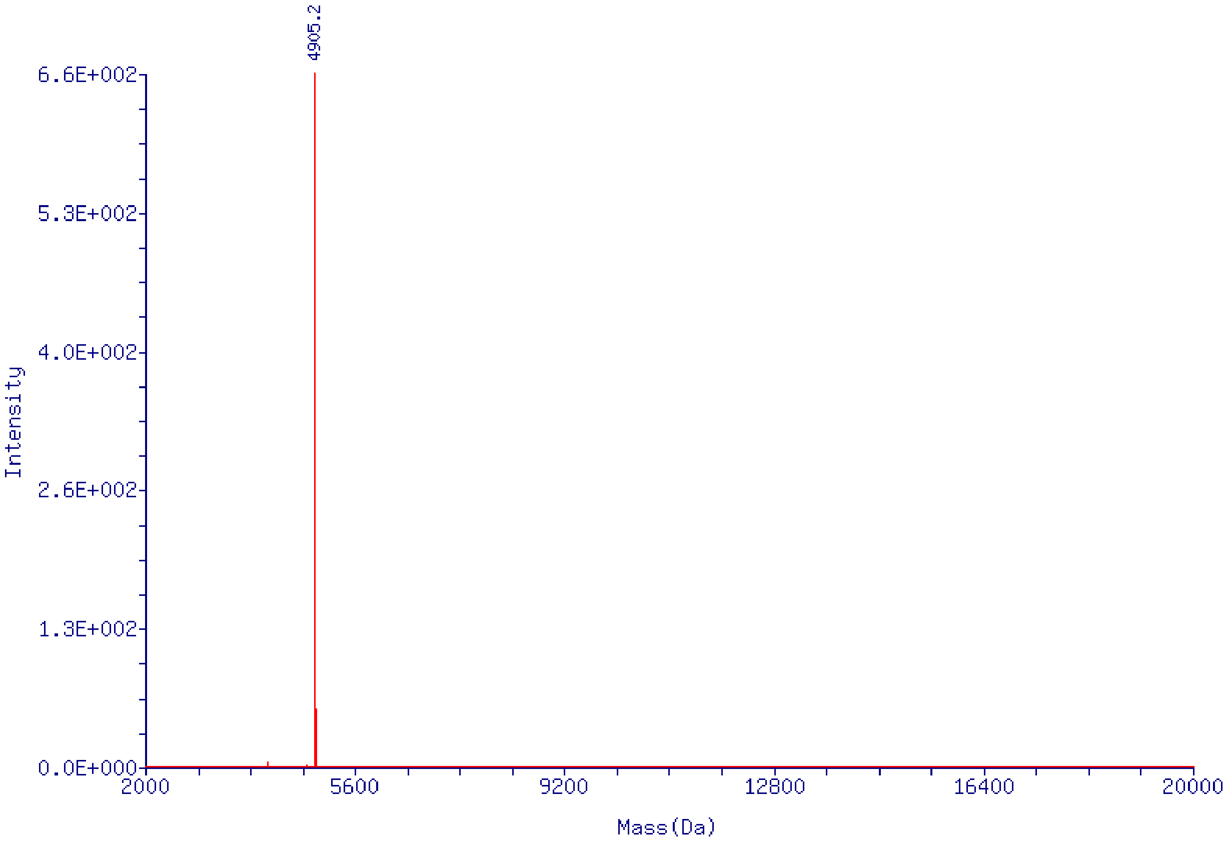

M13 genome sequences

M13mp7L2_wt

AATGCTACTACTATTAGTAGAATTGATGCCACCTTTTCAGCTCGCGCCCCAAATGAAA
ATATAGCTAAACAGGTTATTGACCATTTGCGAAATGTATCTAATGGTCAAACTAAATC
TACTCGTTCGCAGAATTGGGAATCAACTGTTACATGGAATGAAACTTCCAGACACCGT
ACTTTAGTTGCATATTTAAAACATGTTGAGCTACAGCACCAGATTCAGCAATTAAGCTC
TAAGCCATCCGCAAAAATGACCTCTTATCAAAAGGAGCAATTAAAGGTACTCTCTAAT
CCTGACCTGTTGGAGTTTGCTTCCGGTCTGGTTCGCTTTGAAGCTCGAATTAAAACGCG
ATATTTGAAGTCTTTCGGGCTTCCTCTTAATCTTTTTGATGCAATCCGCTTTGCTTCTGA
CTATAATAGTCAGGGTAAAGACCTGATTTTTGATTTATGGTCATTCTCGTTTTCTGAAC
TGTTTAAAGCATTTGAGGGGGATTCAATGAATATTTATGACGATTCCGCAGTATTGGA
CGCTATCCAGTCTAAACATTTTACTATTACCCCCTCTGGCAAAACTTCTTTTGCAAAAG
CCTCTCGCTATTTTGGTTTTTATCGTCGTCTGGTAAACGAGGGTTATGATAGTGTTGCTC
TTACTATGCCTCGTAATTCCTTTTGGCGTTATGTATCTGCATTAGTTGAATGTGGTATTC
CTAAATCTCAACTGATGAATCTTTCTACCTGTAATAATGTTGTTCCGTTAGTTCGTTTTA
TTAACGTAGATTTTTCTTCCCAACGTCCTGACTGGTATAATGAGCCAGTTCTTAAAATC
GCATAAGGTAATTCACAATGATTAAAGTTGAAATTAAACCATCTCAAGCCCAATTTAC
TACTCGTTCTGGTGTTTCTCGTCAGGGCAAGCCTTATTCACTGAATGAGCAGCTTTGTT
ACGTTGATTTGGGTAATGAATATCCGGTTCTTGTCAAGATTACTCTTGATGAAGGTCAG
CCAGCCTATGCGCCTGGTCTGTACACCGTTCATCTGTCCTCTTTCAAAGTTGGTCAGTT
CGGTTCCCTTATGATTGACCGTCTGCGCCTCGTTCCGGCTAAGTAACATGGAGCAGGTC
GCGGATTTCGACACAATTTATCAGGCGATGATACAAATCTCCGTTGTACTTTGTTTCGC
GCTTGGTATAATCGCTGGGGGTCAAAGATGAGTGTTTTAGTGTATTCTTTCGCCTCTTT
CGTTTTAGGTTGGTGCCTTCGTAGTGGCATTACGTATTTTACCCGTTTAATGGAAACTT
CCTCATGAAAAAGTCTTTAGTCCTCAAAGCCTCTGTAGCCGTTGCTACCCTCGTTCCGA
TGCTGTCTTTCGCTGCTGAGGGTGACGATCCCGCAAAAGCGGCCTTTAACTCCCTGCAA
GCCTCAGCGACCGAATATATCGGTTATGCGTGGGCGATGGTTGTTGTCATTGTCGGCG
CAACTATCGGTATCAAGCTGTTTAAGAAATTCACCTCGAAAGCAAGCTGATAAACCGA
TACAATTAAAGGCTCCTTTTGGAGCCTTTTTTTTTGGAGATTTTCAACGTGAAAAAATT
ATTATTCGCAATTCCTTTAGTTGTTCCTTTCTATTCTCACTCCGCTGAAACTGTTGAAAG
TTGTTTAGCAAAACCCCATACAGAAAATTCATTTACTAACGTCTGGAAAGACGACAAA
ACTTTAGATCGTTACGCTAACTATGAGGGTTGTCTGTGGAATGCTACAGGCGTTGTAGT
TTGTACTGGTGACGAAACTCAGTGTTACGGTACATGGGTTCCTATTGGGCTTGCTATCC
CTGAAAATGAGGGTGGTGGCTCTGAGGGTGGCGGTTCTGAGGGTGGCGGTTCTGAGGG
TGGCGGTACTAAACCTCCTGAGTACGGTGATACACCTATTCCGGGCTATACTTATATCA
ACCCTCTCGACGGCACTTATCCGCCTGGTACTGAGCAAAACCCCGCTAATCCTAATCCT
TCTCTTGAGGAGTCTCAGCCTCTTAATACTTTCATGTTTCAGAATAATAGGTTCCGAAA
TAGGCAGGGGGCATTAACTGTTTATACGGGCACTGTTACTCAAGGCACTGACCCCGTT
AAAACTTATTACCAGTACACTCCTGTATCATCAAAAGCCATGTATGACGCTTACTGGA

ACGGTAAATTCAGAGACTGCGCTTTCCATTCTGGCTTTAATGAAGATCCATTCGTTTGT
GAATATCAAGGCCAATCGTCTGACCTGCCTCAACCTCCTGTCAATGCTGGCGGCGGCT
CTGGTGGTGGTTCTGGTGGCGGCTCTGAGGGTGGTGGCTCTGAGGGTGGCGGTTCTGA
GGGTGGCGGCTCTGAGGGAGGCGGTTCCGGTGGTGGCTCTGGTTCCGGTGATTTTGAT
TATGAAAAGATGGCAAACGCTAATAAGGGGGCTATGACCGAAAATGCCGATGAAAAC
GCGCTACAGTCTGACGCTAAAGGCAAACTTGATTCTGTCGCTACTGATTACGGTGCTG
CTATCGATGGTTTCATTGGTGACGTTTCCGGCCTTGCTAATGGTAATGGTGCTACTGGT
GATTTTGCTGGCTCTAATTCCCAAATGGCTCAAGTCGGTGACGGTGATAATTCACCTTT
AATGAATAATTTCCGTCAATATTTACCTTCCCTCCCTCAATCGGTTGAATGTCGCCCTTT
TGTCTTTAGCGCTGGTAAACCATATGAATTTTCTATTGATTGTGACAAAATAAACTTAT
TCCGTGGTGTCTTTGCGTTTCTTTTATATGTTGCCACCTTTATGTATGTATTTTCTACGTT
TGCTAACATACTGCGTAATAAGGAGTCTTAATCATGCCAGTTCTTTTGGGTATTCCGTT
ATTATTGCGTTTCCTCGGTTTCCTTCTGGTAACTTTGTTCGGCTATCTGCTTACTTTTCTT
AAAAAGGGCTTCGGTAAGATAGCTATTGCTATTTCATTGTTTCTTGCTCTTATTATTGG
GCTTAACTCAATTCTTGTGGGTTATCTCTCTGATATTAGCGCTCAATTACCCTCTGACTT
TGTTCAGGGTGTTCAGTTAATTCTCCCGTCTAATGCGCTTCCCTGTTTTTATGTTATTCT
CTCTGTAAAGGCTGCTATTTTCATTTTTGACGTTAAACAAAAAATCGTTTCTTATTTGG
ATTGGGATAAATAATATGGCTGTTTATTTTGTAACTGGCAAATTAGGCTCTGGAAAGA
CGCTCGTTAGCGTTGGTAAGATTCAGGATAAAATTGTAGCTGGGTGCAAAATAGCAAC
TAATCTTGATTTAAGGCTTCAAAACCTCCCGCAAGTCGGGAGGTTCGCTAAAACGCCT
CGCGTTCTTAGAATACCGGATAAGCCTTCTATATCTGATTTGCTTGCTATTGGGCGCGG
TAATGATTCCTACGATGAAAATAAAAACGGCTTGCTTGTTCTCGATGAGTGCGGTACTT
GGTTTAATACCCGTTCTTGGAATGATAAGGAAAGACAGCCGATTATTGATTGGTTTCTA
CATGCTCGTAAATTAGGATGGGATATTATTTTTCTTGTTCAGGACTTATCTATTGTTGAT
AAACAGGCGCGTTCTGCATTAGCTGAACATGTTGTTTATTGTCGTCGTCTGGACAGAAT
TACTTTACCTTTTGTCGGTACTTTATATTCTCTTATTACTGGCTCGAAAATGCCTCTGCC
TAAATTACATGTTGGCGTTGTTAAATATGGCGATTCTCAATTAAGCCCTACTGTTGAGC
GTTGGCTTTATACTGGTAAGAATTTGTATAACGCATATGATACTAAACAGGCTTTTTCT
AGTAATTATGATTCCGGTGTTTATTCTTATTTAACGCCTTATTTATCACACGGTCGGTAT
TTCAAACCATTAAATTTAGGTCAGAAGATGAAATTAACTAAAATATATTTGAAAAAGT
TTTCTCGCGTTCTTTGTCTTGCGATTGGATTTGCATCAGCATTTACATATAGTTATATAA
CCCAACCTAAGCCGGAGGTTAAAAAGGTAGTCTCTCAGACCTATGATTTTGATAAATT
CACTATTGACTCTTCTCAGCGTCTTAATCTAAGCTATCGCTATGTTTTCAAGGATTCTA
AGGGAAAATTAATTAATAGCGACGATTTACAGAAGCAAGGTTATTCACTCACATATAT
TGATTTATGTACTGTTTCCATTAAAAAAGGTAATTCAAATGAAATTGTTAAATGTAATT
AATTTTGTTTTCTTGATGTTTGTTTCATCATCTTCTTTTGCTCAGGTAATTGAAATGAAT
AATTCGCCTCTGCGCGATTTTGTAACTTGGTATTCAAAGCAATCAGGCGAATCCGTTAT
TGTTTCTCCCGATGTAAAGGTACTGTTACTGTATATTCATCTGACGTTAAACCTGAAA
ATCTACGCAATTTCTTTATTTCTGTTTTACGTGCTAATAATTTTGATATGGTTGGTTCAA
TTCCTTCCATAATTCAGAAGTATAATCCAAACAATCAGGATTATATTGATGAATTGCCA

TCATCTGATAATCAGGAATATGATGATAATTCCGCTCCTTCTGGTGGTTTCTTTGTTCC
GCAAAATGATAATGTTACTCAAACTTTTAAAATTAATAACGTTCGGGCAAAGGATTTA
ATACGAGTTGTCGAATTGTTTGTAAAGTCTAATACTTCTAAATCCTCAAATGTATTATC
TATTGACGGCTCTAATCTATTAGTTGTTAGTGCACCTAAAGATATTTTAGATAACCTTC
CTCAATTCCTTTCTACTGTTGATTTGCCAACTGACCAGATATTGATTGAGGGTTTGATA
TTTGAGGTTCAGCAAGGTGATGCTTTAGATTTTTCATTTGCTGCTGGCTCTCAGCGTGG
CACTGTTGCAGGCGGTGTTAATACTGACCGCCTCACCTCTGTTTTATCTTCTGCTGGTG
GTTCGTTCGGTATTTTTAATGGCGATGTTTTAGGGCTATCAGTTCGCGCATTAAAGACT
AATAGCCATTCAAAAATATTGTCTGTGCCACGTATTCTTACGCTTTCAGGTCAGAAGGG
TTCTATCTCTGTTGGCCAGAATGTCCCTTTTATTACTGGTCGTGTGACTGGTGAATCTGC
CAATGTAAATAATCCATTTCAGACGATTGAGCGTCAAAATGTAGGTATTTCCATGAGC
GTTTTTCCTGTTGCAATGGCTGGCGGTAATATTGTTCTGGATATTACCAGCAAGGCCGA
TAGTTTGAGTTCTTCTACTCAGGCAAGTGATGTTATTACTAATCAAAGAAGTATTGCTA
CAACGGTTAATTTGCGTGATGGACAGACTCTTTTACTCGGTGGCCTCACTGATTATAAA
AACACTTCTCAAGATTCTGGCGTACCGTTCCTGTCTAAAATCCCTTTAATCGGCCTCCT
GTTTAGCTCCCGCTCTGATTCCAACGAGGAAAGCACGTTATACGTGCTCGTCAAAGCA
ACCATAGTACGCGCCCTGTAGCGGCGCATTAAGCGCGGCGGGTGTGGTGGTTACGCGC
AGCGTGACCGCTACACTTGCCAGCGCCCTAGCGCCCGCTCCTTTCGCTTTCTTCCCTTC
CTTTCTCGCCACGTTCGCCGGCTTTCCCCGTCAAGCTCTAAATCGGGGGCTCCCTTTAG
GGTTCCGATTTAGTGCTTTACGGCACCTCGACCCCAAAAAACTTGATTTGGGTGATGGT
TCACGTAGTGGGCCATCGCCCTGATAGACGGTTTTTCGCCCTTTGACGTTGGAGTCCAC
GTTCTTTAATAGTGGACTCTTGTTCCAAACTGGAACAACACTCAACCCTATCTCGGGCT
ATTCTTTTGATTTATAAGGGATTTTGCCGATTTCGGAACCACCATCAAACAGGATTTTC
GCCTGCTGGGGCAAACCAGCGTGGACCGCTTGCTGCAACTCTCTCAGGGCCAGGCGGT
GAAGGGCAATCAGCTGTTGCCCGTCTCGCTGGTGAAAAGAAAAACCACCCTGGCGCCC
AATACGCAAACCGCCTCTCCCCGCGCGTTGGCCGATTCATTAATGCAGCTGGCACGAC
AGGTTTCCCGACTGGAAAGCGGGCAGTGAGCGCAACGCAATTAATGTGAGTTAGCTCA
CTCATTAGGCACCCCAGGCTTTACACTTTATGCTTCCGGCTCGTATGTTGTGTGGAATT
GTGAGCGGATAACAATTTCACACAGGAAACAGCTATGACCATGATTCAGTGAATTCAC
TGGCCGTCGTTTTACAACGTCGTGACTGGGAAAACCCTGGCGTTACCCAACTTAATCG
CCTTGCAGCACATCCCCCTTTCGCCAGCTGGCGTAATAGCGAAGAGGCCCGCACCGAT
CGCCCTTCCCAACAGTTGCGCAGCCTGAATGGCGAATGGCGCTTTGCCTGGTTTCCGGC
ACCAGAAGCGGTGCCGGAAAGCTGGCTGGAGTGCGATCTTCCTGAGGCCGATACGGTC
GTCGTCCCCTCAAACTGGCAGATGCACGGTTACGATGCGCCCATCTACACCAACGTAA
CCTATCCCATTACGGTCAATCCGCCGTTTGTTCCCACGGAGAATCCGACGGGTTGTTAC
TCGCTCACATTTAATGTTGATGAAAGCTGGCTACAGGAAGGCCAGACGCGAATTATTT
TTGATGGCGTTCCTATTGGTTAAAAAATGAGCTGATTTAACAAAAATTTAACGCGAAT
TTTAACAAAATATTAACGTTTACAATTTAAATATTTGCTTATACAATCTTCCTGTTTTTG
GGGCTTTTCTGATTATCAACCGGGGTACATATGATTGACATGCTAGTTTTACGATTACC
GTTCATCGATTCTCTTGTTTGCTCCAGACTCTCAGGCAATGACCTGATAGCCTTTGTAG

ATCTCTCAAAAATAGCTACCCTCTCCGGCATTAATTTATCAGCTAGAACGGTTGAATAT
CATATTGATGGTGATTTGACTGTCTCCGGCCTTTCTCACCCTTTTGAATCTTTACCTACA
CATTACTCAGGCATTGCATTTAAAATATATGAGGGTTCTAAAAATTTTTATCCTTGCGT
TGAAATAAAGGCTTCTCCCGCAAAAGTATTACAGGGTCATAATGTTTTTGGTACAACC
GATTTAGCTTTATGCTCTGAGGCTTTATTGCTTAATTTTGCTAATTCTTTGCCTTGCCTG
TATGATTTATTGGATGTT

M13mp7L2_plus_34mer
AATGCTACTACTATTAGTAGAATTGATGCCACCTTTTCAGCTCGCGCCCCAAATGAAA
ATATAGCTAAACAGGTTATTGACCATTTGCGAAATGTATCTAATGGTCAAACTAAATC
TACTCGTTCGCAGAATTGGGAATCAACTGTTACATGGAATGAAACTTCCAGACACCGT
ACTTTAGTTGCATATTTAAAACATGTTGAGCTACAGCACCAGATTCAGCAATTAAGCTC
TAAGCCATCCGCAAAAATGACCTCTTATCAAAAGGAGCAATTAAAGGTACTCTCTAAT
CCTGACCTGTTGGAGTTTGCTTCCGGTCTGGTTCGCTTTGAAGCTCGAATTAAAACGCG
ATATTTGAAGTCTTTCGGGCTTCCTCTTAATCTTTTTGATGCAATCCGCTTTGCTTCTGA
CTATAATAGTCAGGGTAAAGACCTGATTTTTGATTTATGGTCATTCTCGTTTTCTGAAC
TGTTTAAAGCATTTGAGGGGGATTCAATGAATATTTATGACGATTCCGCAGTATTGGA
CGCTATCCAGTCTAAACATTTTACTATTACCCCCTCTGGCAAAACTTCTTTTGCAAAAG
CCTCTCGCTATTTTGGTTTTTATCGTCGTCTGGTAAACGAGGGTTATGATAGTGTTGCTC
TTACTATGCCTCGTAATTCCTTTTGGCGTTATGTATCTGCATTAGTTGAATGTGGTATTC
CTAAATCTCAACTGATGAATCTTTCTACCTGTAATAATGTTGTTCCGTTAGTTCGTTTTA
TTAACGTAGATTTTTCTTCCCAACGTCCTGACTGGTATAATGAGCCAGTTCTTAAAATC
GCATAAGGTAATTCACAATGATTAAAGTTGAAATTAAACCATCTCAAGCCCAATTTAC
TACTCGTTCTGGTGTTTCTCGTCAGGGCAAGCCTTATTCACTGAATGAGCAGCTTTGTT
ACGTTGATTTGGGTAATGAATATCCGGTTCTTGTCAAGATTACTCTTGATGAAGGTCAG
CCAGCCTATGCGCCTGGTCTGTACACCGTTCATCTGTCCTCTTTCAAAGTTGGTCAGTT
CGGTTCCCTTATGATTGACCGTCTGCGCCTCGTTCCGGCTAAGTAACATGGAGCAGGTC
GCGGATTTCGACACAATTTATCAGGCGATGATACAAATCTCCGTTGTACTTTGTTTCGC
GCTTGGTATAATCGCTGGGGGTCAAAGATGAGTGTTTTAGTGTATTCTTTCGCCTCTTT
CGTTTTAGGTTGGTGCCTTCGTAGTGGCATTACGTATTTTACCCGTTTAATGGAAACTT
CCTCATGAAAAAGTCTTTAGTCCTCAAAGCCTCTGTAGCCGTTGCTACCCTCGTTCCGA
TGCTGTCTTTCGCTGCTGAGGGTGACGATCCCGCAAAAGCGGCCTTTAACTCCCTGCAA
GCCTCAGCGACCGAATATATCGGTTATGCGTGGGCGATGGTTGTTGTCATTGTCGGCG
CAACTATCGGTATCAAGCTGTTTAAGAAATTCACCTCGAAAGCAAGCTGATAAACCGA
TACAATTAAAGGCTCCTTTTGGAGCCTTTTTTTTTGGAGATTTTCAACGTGAAAAAATT
ATTATTCGCAATTCCTTTAGTTGTTCCTTTCTATTCTCACTCCGCTGAAACTGTTGAAAG
TTGTTTAGCAAAACCCCATACAGAAAATTCATTTACTAACGTCTGGAAAGACGACAAA
ACTTTAGATCGTTACGCTAACTATGAGGGTTGTCTGTGGAATGCTACAGGCGTTGTAGT
TTGTACTGGTGACGAAACTCAGTGTTACGGTACATGGGTTCCTATTGGGCTTGCTATCC
CTGAAAATGAGGGTGGTGGCTCTGAGGGTGGCGGTTCTGAGGGTGGCGGTTCTGAGGG

TGGCGGTACTAAACCTCCTGAGTACGGTGATACACCTATTCCGGGCTATACTTATATCA
ACCCTCTCGACGGCACTTATCCGCCTGGTACTGAGCAAAACCCCGCTAATCCTAATCCT
TCTCTTGAGGAGTCTCAGCCTCTTAATACTTTCATGTTTCAGAATAATAGGTTCCGAAA
TAGGCAGGGGGCATTAACTGTTTATACGGGCACTGTTACTCAAGGCACTGACCCCGTT
AAAACTTATTACCAGTACACTCCTGTATCATCAAAAGCCATGTATGACGCTTACTGGA
ACGGTAAATTCAGAGACTGCGCTTTCCATTCTGGCTTTAATGAAGATCCATTCGTTTGT
GAATATCAAGGCCAATCGTCTGACCTGCCTCAACCTCCTGTCAATGCTGGCGGCGGCT
CTGGTGGTGGTTCTGGTGGCGGCTCTGAGGGTGGTGGCTCTGAGGGTGGCGGTTCTGA
GGGTGGCGGCTCTGAGGGAGGCGGTTCCGGTGGTGGCTCTGGTTCCGGTGATTTTGAT
TATGAAAAGATGGCAAACGCTAATAAGGGGGCTATGACCGAAAATGCCGATGAAAAC
GCGCTACAGTCTGACGCTAAAGGCAAACTTGATTCTGTCGCTACTGATTACGGTGCTG
CTATCGATGGTTTCATTGGTGACGTTTCCGGCCTTGCTAATGGTAATGGTGCTACTGGT
GATTTTGCTGGCTCTAATTCCCAAATGGCTCAAGTCGGTGACGGTGATAATTCACCTTT
AATGAATAATTTCCGTCAATATTTACCTTCCCTCCCTCAATCGGTTGAATGTCGCCCTTT
TGTCTTTAGCGCTGGTAAACCATATGAATTTTCTATTGATTGTGACAAAATAAACTTAT
TCCGTGGTGTCTTTGCGTTTCTTTTATATGTTGCCACCTTTATGTATGTATTTTCTACGTT
TGCTAACATACTGCGTAATAAGGAGTCTTAATCATGCCAGTTCTTTTGGGTATTCCGTT
ATTATTGCGTTTCCTCGGTTTCCTTCTGGTAACTTTGTTCGGCTATCTGCTTACTTTTCTT
AAAAAGGGCTTCGGTAAGATAGCTATTGCTATTTCATTGTTTCTTGCTCTTATTATTGG
GCTTAACTCAATTCTTGTGGGTTATCTCTCTGATATTAGCGCTCAATTACCCTCTGACTT
TGTTCAGGGTGTTCAGTTAATTCTCCCGTCTAATGCGCTTCCCTGTTTTTATGTTATTCT
CTCTGTAAAGGCTGCTATTTTCATTTTTGACGTTAAACAAAAAATCGTTTCTTATTTGG
ATTGGGATAAATAATATGGCTGTTTATTTTGTAACTGGCAAATTAGGCTCTGGAAAGA
CGCTCGTTAGCGTTGGTAAGATTCAGGATAAAATTGTAGCTGGGTGCAAAATAGCAAC
TAATCTTGATTTAAGGCTTCAAAACCTCCCGCAAGTCGGGAGGTTCGCTAAAACGCCT
CGCGTTCTTAGAATACCGGATAAGCCTTCTATATCTGATTTGCTTGCTATTGGGCGCGG
TAATGATTCCTACGATGAAAATAAAAACGGCTTGCTTGTTCTCGATGAGTGCGGTACTT
GGTTTAATACCCGTTCTTGGAATGATAAGGAAAGACAGCCGATTATTGATTGGTTTCTA
CATGCTCGTAAATTAGGATGGGATATTATTTTTCTTGTTCAGGACTTATCTATTGTTGAT
AAACAGGCGCGTTCTGCATTAGCTGAACATGTTGTTTATTGTCGTCGTCTGGACAGAAT
TACTTTACCTTTTGTCGGTACTTTATATTCTCTTATTACTGGCTCGAAAATGCCTCTGCC
TAAATTACATGTTGGCGTTGTTAAATATGGCGATTCTCAATTAAGCCCTACTGTTGAGC
GTTGGCTTTATACTGGTAAGAATTTGTATAACGCATATGATACTAAACAGGCTTTTTCT
AGTAATTATGATTCCGGTGTTTATTCTTATTTAACGCCTTATTTATCACACGGTCGGTAT
TTCAAACCATTAAATTTAGGTCAGAAGATGAAATTAACTAAAATATATTTGAAAAAGT
TTTCTCGCGTTCTTTGTCTTGCGATTGGATTTGCATCAGCATTTACATATAGTTATATAA
CCCAACCTAAGCCGGAGGTTAAAAAGGTAGTCTCTCAGACCTATGATTTTGATAAATT
CACTATTGACTCTTCTCAGCGTCTTAATCTAAGCTATCGCTATGTTTTCAAGGATTCTA
AGGGAAAATTAATTAATAGCGACGATTTACAGAAGCAAGGTTATTCACTCACATATAT
TGATTTATGTACTGTTTCCATTAAAAAAGGTAATTCAAATGAAATTGTTAAATGTAATT

AATTTTGTTTTCTTGATGTTTGTTTCATCATCTTCTTTTGCTCAGGTAATTGAAATGAAT
AATTCGCCTCTGCGCGATTTTGTAACTTGGTATTCAAAGCAATCAGGCGAATCCGTTAT
TGTTTCTCCCGATGTAAAAGGTACTGTTACTGTATATTCATCTGACGTTAAACCTGAAA
ATCTACGCAATTTCTTTATTTCTGTTTTACGTGCTAATAATTTTGATATGGTTGGTTCAA
TTCCTTCCATAATTCAGAAGTATAATCCAAACAATCAGGATTATATTGATGAATTGCCA
TCATCTGATAATCAGGAATATGATGATAATTCCGCTCCTTCTGGTGGTTTCTTTGTTCC
GCAAAATGATAATGTTACTCAAACTTTTAAAATTAATAACGTTCGGGCAAAGGATTTA
ATACGAGTTGTCGAATTGTTTGTAAAGTCTAATACTTCTAAATCCTCAAATGTATTATC
TATTGACGGCTCTAATCTATTAGTTGTTAGTGCACCTAAAGATATTTTAGATAACCTTC
CTCAATTCCTTTCTACTGTTGATTTGCCAACTGACCAGATATTGATTGAGGGTTTGATA
TTTGAGGTTCAGCAAGGTGATGCTTTAGATTTTTCATTTGCTGCTGGCTCTCAGCGTGG
CACTGTTGCAGGCGGTGTTAATACTGACCGCCTCACCTCTGTTTTATCTTCTGCTGGTG
GTTCGTTCGGTATTTTTAATGGCGATGTTTTAGGGCTATCAGTTCGCGCATTAAAGACT
AATAGCCATTCAAAAATATTGTCTGTGCCACGTATTCTTACGCTTTCAGGTCAGAAGGG
TTCTATCTCTGTTGGCCAGAATGTCCCTTTTATTACTGGTCGTGTGACTGGTGAATCTGC
CAATGTAAATAATCCATTTCAGACGATTGAGCGTCAAAATGTAGGTATTTCCATGAGC
GTTTTTCCTGTTGCAATGGCTGGCGGTAATATTGTTCTGGATATTACCAGCAAGGCCGA
TAGTTTGAGTTCTTCTACTCAGGCAAGTGATGTTATTACTAATCAAAGAAGTATTGCTA
CAACGGTTAATTTGCGTGATGGACAGACTCTTTTACTCGGTGGCCTCACTGATTATAAA
AACACTTCTCAAGATTCTGGCGTACCGTTCCTGTCTAAAATCCCTTTAATCGGCCTCCT
GTTTAGCTCCCGCTCTGATTCCAACGAGGAAAGCACGTTATACGTGCTCGTCAAAGCA
ACCATAGTACGCGCCCTGTAGCGGCGCATTAAGCGCGGCGGGTGTGGTGGTTACGCGC
AGCGTGACCGCTACACTTGCCAGCGCCCTAGCGCCCGCTCCTTTCGCTTTCTTCCCTTC
CTTTCTCGCCACGTTCGCCGGCTTTCCCCGTCAAGCTCTAAATCGGGGGCTCCCTTTAG
GGTTCCGATTTAGTGCTTTACGGCACCTCGACCCCAAAAAACTTGATTTGGGTGATGGT
TCACGTAGTGGGCCATCGCCCTGATAGACGGTTTTTCGCCCTTTGACGTTGGAGTCCAC
GTTCTTTAATAGTGGACTCTTGTTCCAAACTGGAACAACACTCAACCCTATCTCGGGCT
ATTCTTTTGATTTATAAGGGATTTTGCCGATTTCGGAACCACCATCAAACAGGATTTTC
GCCTGCTGGGGCAAACCAGCGTGGACCGCTTGCTGCAACTCTCTCAGGGCCAGGCGGT
GAAGGGCAATCAGCTGTTGCCCGTCTCGCTGGTGAAAAGAAAAACCACCCTGGCGCCC
AATACGCAAACCGCCTCTCCCCGCGCGTTGGCCGATTCATTAATGCAGCTGGCACGAC
AGGTTTCCCGACTGGAAAGCGGGCAGTGAGCGCAACGCAATTAATGTGAGTTAGCTCA
CTCATTAGGCACCCCAGGCTTTACACTTTATGCTTCCGGCTCGTATGTTGTGTGGAATT
GTGAGCGGATAACAATTTCACACAGGAAACAGCTATGACCATGATTCAGTGCACGGTT
AGTGCTCTGACGAAGACCTAGGCGTCCAATTCACTGGCCGTCGTTTTACAACGTCGTG
ACTGGGAAAACCCTGGCGTTACCCAACTTAATCGCCTTGCAGCACATCCCCCTTTCGCC
AGCTGGCGTAATAGCGAAGAGGCCCGCACCGATCGCCCTTCCCAACAGTTGCGCAGCC
TGAATGGCGAATGGCGCTTTGCCTGGTTTCCGGCACCAGAAGCGGTGCCGGAAAGCTG
GCTGGAGTGCGATCTTCCTGAGGCCGATACGGTCGTCGTCCCCTCAAACTGGCAGATG
CACGGTTACGATGCGCCCATCTACACCAACGTAACCTATCCCATTACGGTCAATCCGC

CGTTTGTTCCCACGGAGAATCCGACGGGTTGTTACTCGCTCACATTTAATGTTGATGAA
AGCTGGCTACAGGAAGGCCAGACGCGAATTATTTTTGATGGCGTTCCTATTGGTTAAA
AAATGAGCTGATTTAACAAAAATTTAACGCGAATTTTAACAAAATATTAACGTTTACA
ATTTAAATATTTGCTTATACAATCTTCCTGTTTTTGGGGCTTTTCTGATTATCAACCGGG
GTACATATGATTGACATGCTAGTTTTACGATTACCGTTCATCGATTCTCTTGTTTGCTCC
AGACTCTCAGGCAATGACCTGATAGCCTTTGTAGATCTCTCAAAAATAGCTACCCTCTC
CGGCATTAATTTATCAGCTAGAACGGTTGAATATCATATTGATGGTGATTTGACTGTCT
CCGGCCTTTCTCACCCTTTTGAATCTTTACCTACACATTACTCAGGCATTGCATTTAAA
ATATATGAGGGTTCTAAAAATTTTTATCCTTGCGTTGAAATAAAGGCTTCTCCCGCAAA
AGTATTACAGGGTCATAATGTTTTTGGTACAACCGATTTAGCTTTATGCTCTGAGGCTT
TATTGCTTAATTTTGCTAATTCTTTGCCTTGCCTGTATGATTTATTGGATGTT

References

1. Chatgilialoglu, C., Navacchia, M.L. and Postigo, A. (2006) A facile one-pot synthesis of 8-oxo-7,8-dihydro-(2 '-deoxy)adenosine in water. *Tetrahedron Lett*, **47**, 711-714.
2. Møller, C. and Plesset, M.S. (1934) Note on an Approximation Treatment for Many-Electron Systems. *Phys Rev*, **46**, 618-622.
3. Barone, V. and Cossi, M. (1998) Quantum calculation of molecular energies and energy gradients in solution by a conductor solvent model. *J Phys Chem A*, **102**, 1995-2001.
4. Singh, U.C. and Kollman, P.A. (1984) An Approach to Computing Electrostatic Charges for Molecules. *J Comput Chem*, **5**, 129-145.
5. Bayly, C.I., Cieplak, P., Cornell, W.D. and Kollman, P.A. (1993) A Well-Behaved Electrostatic Potential Based Method Using Charge Restraints for Deriving Atomic Charges - the Resp Model. *J Phys Chem-Us*, **97**, 10269-10280.
6 Frisch, M. J., et al. Gaussian 09, Revision A.1, Gaussian, Inc., allingford CT, 2009.
7 Case, D.A., et al. AMBER 2018, University of California, San Francisco, 2018.
8. Izadi, S. and Onufriev, A.V. (2016) Accuracy limit of rigid 3-point water models. *J Chem Phys*, **145**, 074501.
9. Zgarbova, M., Luque, F.J., Sponer, J., Cheatham, T.E., Otyepka, M. and Jurecka, P. (2013) Toward Improved Description of DNA Backbone: Revisiting Epsilon and Zeta Torsion Force Field Parameters. *J Chem Theory Comput*, **9**, 2339-2354.
10. Zgarbova, M., Sponer, J., Otyepka, M., Cheatham, T.E., 3rd, Galindo-Murillo, R. and Jurecka, P. (2015) Refinement of the Sugar-Phosphate Backbone Torsion Beta for AMBER Force Fields Improves the Description of Z- and B-DNA. *J Chem Theory Comput*, **11**, 5723-5736.
11. Wang, J., Wolf, R.M., Caldwell, J.W., Kollman, P.A. and Case, D.A. (2004) Development and testing of a general amber force field. *J Comput Chem*, **25**, 1157-1174.
12. Ryckaert, J.P, Ciccotti, G. and Berendsen H.J.C. (1997) Numerical integration of the cartesian equations of motion of a system with constraints: molecular dynamics of *n*-alkanes. *J Comput Phys*, **23**, 327-341.
13 Darden, T., York, D., and Pedersen, L. (1993) Particle mesh Ewald: An $N \cdot \log(N)$ method for Ewald sums in large systems. *J Chem Phys*, **98**, 10089.
14. Onufriev, A., Bashford, D. and Case, D.A. (2000) Modification of the generalized Born model suitable for macromolecules. *J Phys Chem B*, **104**, 3712-3720.

15. Humphrey, W., Dalke, A. and Schulten, K. (1996) VMD: visual molecular dynamics. *J Mol Graph*, **14**, 33-38, 27-38.
16. Ivani, I., Dans, P.D., Noy, A., Perez, A., Faustino, I., Hospital, A., Walther, J., Andrio, P., Goni, R., Balaceanu, A. *et al.* (2016) Parmbsc1: a refined force field for DNA simulations. *Nat Methods*, **13**, 55-58.
17. Soliva, R., Monaco, V., Gomez-Pinto, I., Meeuwenoord, N.J., Marel, G.A., Boom, J.H., Gonzalez, C. and Orozco, M. (2001) Solution structure of a DNA duplex with a chiral alkyl phosphonate moiety. *Nucleic Acids Res*, **29**, 2973-2985.
18. Lu, X.J. and Olson, W.K. (2008) 3DNA: a versatile, integrated software system for the analysis, rebuilding and visualization of three-dimensional nucleic-acid structures. *Nat Protoc*, **3**, 1213-1227.
19. Koradi, R., Billeter, M. and Wuthrich, K. (1996) MOLMOL: a program for display and analysis of macromolecular structures. *J Mol Graph*, **14**, 51-55, 29-32.
20. LeClerc, J.E., Borden, A. and Lawrence, C.W. (1991) The thymine-thymine pyrimidine-pyrimidone(6-4) ultraviolet light photoproduct is highly mutagenic and specifically induces 3' thymine-to-cytosine transitions in Escherichia coli. *Proc Natl Acad Sci U S A*, **88**, 9685-9689.
21. Gibbs, P.E.M. and Lawrence, C.W. (1993) U-U and T-T Cyclobutane Dimers Have Different Mutational Properties. *Nucleic Acids Research*, **21**, 4059-4065.
22. Hanahan, D. (1983) Studies on transformation of Escherichia coli with plasmids. *J Mol Biol*, **166**, 557-580.
23. Ronayne, E.A., Wan, Y.C., Boudreau, B.A., Landick, R. and Cox, M.M. (2016) P1 Ref Endonuclease: A Molecular Mechanism for Phage-Enhanced Antibiotic Lethality. *PLoS Genet*, **12**, e1005797.
24. Delaney, J.C. and Essigmann, J.M. (2006) Assays for determining lesion bypass efficiency and mutagenicity of site-specific DNA lesions in vivo. *Methods Enzymol*, **408**, 1-15.
25. Chang, S.C., Fedeles, B.I., Wu, J., Delaney, J.C., Li, D., Zhao, L., Christov, P.P., Yau, E., Singh, V., Jost, M. *et al.* (2015) Next-generation sequencing reveals the biological significance of the N(2),3-ethenoguanine lesion in vivo. *Nucleic Acids Res*, **43**, 5489-5500.
26 *Babraham Bioinformatics—FastQC A Quality Control tool for High Throughput Sequence Data*. (n.d.). Retrieved September 25, 2020, from https://www.bioinformatics.babraham.ac.uk/projects/fastqc/
27. Bolger, A.M., Lohse, M. and Usadel, B. (2014) Trimmomatic: a flexible trimmer for Illumina sequence data. *Bioinformatics*, **30**, 2114-2120.
28. Zhang, J., Kobert, K., Flouri, T. and Stamatakis, A. (2014) PEAR: a fast and accurate Illumina Paired-End reAd mergeR. *Bioinformatics*, **30**, 614-620.
29. Langmead, B. and Salzberg, S.L. (2012) Fast gapped-read alignment with Bowtie 2. *Nat Methods*, **9**, 357-359.
30. Li, H. (2011) A statistical framework for SNP calling, mutation discovery, association mapping and population genetical parameter estimation from sequencing data. *Bioinformatics*, **27**, 2987-2993.
31 Wickham, H. *ggplot2: Elegant Graphics for Data Analysis*. Springer-Verlag, **2009**.
